# GPR3 Ligands Discovered through Combined Virtual and Conformational Biosensor-Based Screening

**DOI:** 10.1021/jacs.6c06780

**Published:** 2026-05-07

**Authors:** Hannes Schihada, Aida Shahraki, Ainoleena Turku-Metsänen, Maximilian Rath, Lukas Wirth, Katarina Nemec, Hrisowalantu Tselepli, Laura Heitzer, Bernadette Vallaster, Mariam Fadel, Gunnar Schulte, Daniel Hilger, Steffen Pockes, Martin J. Lohse, Peter Kolb

**Affiliations:** † 9377 Philipps-Universität Marburg, Institute of Pharmaceutical Chemistry, Marbacher Weg 8, 35032 Marburg, Germany; ‡ Section of Receptor Biology & Signaling, Department of Physiology and Pharmacology, 27106 Karolinska Institutet, 17177 Stockholm, Sweden; § Institute of Pharmacy, University of Regensburg, Universitätsstraße 31, 93053 Regensburg, Germany; ∥ 28341 Max Delbrück Center for Molecular Medicine in the Helmholtz Association, 13125 Berlin, Germany; ⊥ ISAR Bioscience Institute, Planegg, 82152 Munich, Germany; # TU München Klinikum Rechts der Isar, 81675 Munich, Germany; ∇ Rudolf-Boehm-Institute for Pharmacology and Toxicology, Faculty of Medicine, Leipzig University, Härtelstrasse 16-18, 04107 Leipzig, Germany

## Abstract

GPR3 belongs to the protein superfamily of G protein-coupled receptors (GPCRs) and plays a central role in both benign and malignant physiological processes, such as energy expenditure in adipocytes and Alzheimer’s disease pathology, respectively. Despite the therapeutic potential of both receptor agonists and inverse agonists, GPR3 so far has lacked drug-like ligands and innovative screening technologies, hindering effective drug discovery efforts targeting this receptor. To overcome the limitations of conventional ligand screening techniques based on cAMP accumulation or β-arrestin recruitment, we developed a conformational GPR3 biosensor to monitor receptor activity in living cells with high-throughput screening (HTS)-compatible sensitivity and robustness. Combined with virtual compound screening against homology models of GPR3 and classical medicinal chemistry, this biosensor enabled us to identify new ligands, one of which (compound **33**) modulates GPR3-dependent G_s_ activity with an average potency in the nanomolar range. Our study not only presents novel GPR3 ligands for future optimization efforts and paves the way for even further expansion of the GPR3 ligand repertoire, but our sensor approach also provides a blueprint for targeting other therapeutically attractive yet challenging orphan GPCRs.

## Graphical abstract

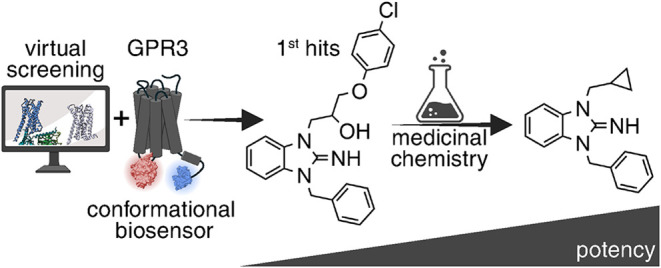

## Introduction

Members of the protein superfamily of G protein-coupled receptors (GPCRs) are targeted by more than 30% of FDA-approved drugs. However, more than two-thirds of all nonolfactory GPCRs remain untapped for disease therapy, including many orphan GPCRs—receptors with yet unknown endogenous ligands. While one of the class A orphan GPCRs, GPR3, has only recently been deorphanized,^1–3^ it still lacks drug-like ligands; moreover, innovative technologies to better study this receptor are just beginning to emerge.^4^

GPR3, together with GPR6 and GPR12, is phylogenetically related to receptors that bind sphingosine-1-phosphate (S1P), lysophosphatidic acid (LPA), cannabinoids and proopiomelanocortin-derived peptides. GPR3 activity is involved in both benign and malignant physiological processes. In the central nervous system (CNS), GPR3 mediates neurite outgrowth and neuronal cell survival^5,6^ but has also been implicated in Alzheimer’s disease.^7–10^ In the periphery, GPR3 regulates oocyte maturation and drives thermogenic programs in adipocytes.^11^ These examples demonstrate that both agonists and inverse agonists of GPR3 may be of therapeutic value for various pathologies. Although researchers have been trying to discover molecules that target GPR3 for more than two decades, only a limited set of ligands that were validated in independent laboratories is currently available (Chart 1). While the activity of S1P and cannabidiol (CBD) on GPR3 remains questionable,^12–16^ AF64394 and its structural analogs have demonstrated their value as GPR3-specific inverse agonists.^4,17,18^ In addition, diphenyleneiodonium chloride (DPI) is a proven synthetic agonist of GPR3 and a DPI derivative promotes GPR3-dependent cAMP production with submicromolar potency.^12,19^ Furthermore, the recent biochemical and structural studies revealed that endogenous long-chain lipids and fatty acids stabilize GPR3 in an active conformation.^1,2^

**Chart 1:**
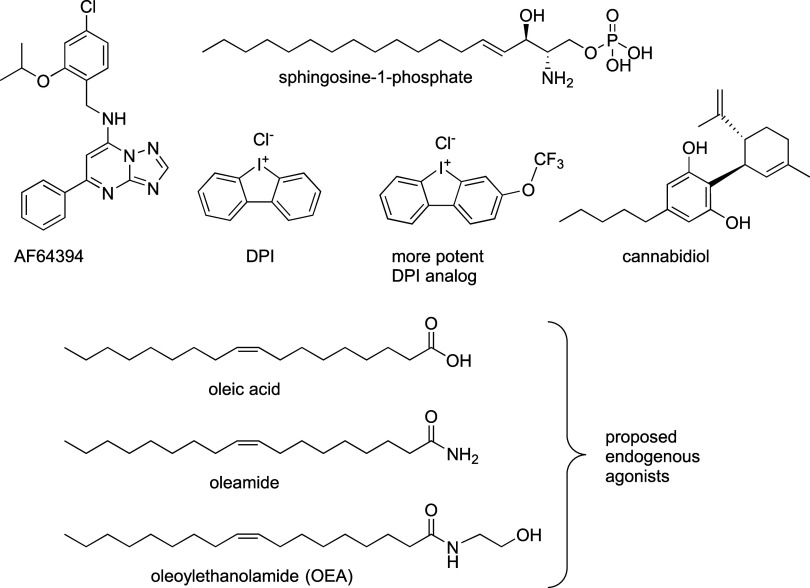
Structures of Proposed GPR3 Ligands

The limited number of success stories illustrates that today’s GPR3-targeted drug discovery is hampered by a poor understanding of the role of this receptor in cellular signaling and by a limited panel of assays that reveal GPR3 activity in living cells. This is emphasized by the fact that the few GPR3 ligands currently validated were discovered using one of only two available assay principles—cAMP accumulation or β-arrestin recruitment.^12,17,18^ Both assays detect selected cellular functions of GPR3 but may not represent the full repertoire of GPR3-mediated events in cells. These readouts are therefore limited in detecting GPR3-targeting compounds and we reasoned that an innovative sensing approach is needed to facilitate tailored GPR3 ligand screening with higher success rates. We aimed to develop a sensor that detects compound-induced changes in GPR3 activity in a pathway-independent manner in a medium- to high-throughput screening (HTS) assay format. The latest generation of conformational GPCR sensors, based on bioluminescence resonance energy transfer (BRET) between NanoLuciferase (Nluc)^20^ and HaloTag,^21^ fulfills these criteria.^22–24^

Here, we present the generation of the first conformational biosensor for an orphan GPCR with HTS-compatible sensitivity and robustness. We further demonstrate how this optical tool enabled us to identify new GPR3 ligands by combining virtual compound screening against 3D receptor models with a classical medicinal chemistry approach. Our most potent new GPR3 ligand is an inverse agonist that induces conformational changes in GPR3 with low micromolar potency and reduced basal G_s_ activity downstream of GPR3 with an average potency in the nanomolar range.

## Results and Discussion

### Design and Validation of a Conformational GPR3 Sensor

To develop a conformational GPR3 biosensor that can be used in a microtiter well plate format, we employed the intramolecular BRET design previously validated for several GPCRs.^22–24^ The BRET donor NanoLuc was fused to the receptor’s full-length C-terminus and the self-labeling protein tag, HaloTag, was inserted into GPR3′s third intracellular loop between amino acids Arg231 and His232 (Figure 1a; sequence in Figure S1). This GPR3-HaloTag/Nluc fusion construct even showed enhanced surface expression levels compared to wildtype GPR3, confirmed by whole-cell ELISA using the N-terminal HA-Tag of these GPR3 constructs (Figure 1b). Upon expression of GPR3-HaloTag/Nluc in human embryonic kidney 293A cells (HEK293A), fluorescence labeling with the HaloTag NanoBRET 618 ligand and addition of Nluc substrate furimazine, BRET in the receptor’s basal conformation was indicated in the luminescence emission spectrum by the characteristic acceptor peak around 620 nm (Figure 1c). In addition, treatment with the GPR3 inverse agonist AF64394 resulted in a time- and ligand-concentration-dependent increase in BRET (Figure 1d,e). The EC_50_ value of 161 nM is similar to the affinity of AF64394 for N-terminally Nluc-fused GPR3 ^4^ and to its potency in a GPR3 wildtype-dependent cAMP assay,^17^ demonstrating the functionality of this conformational GPR3 biosensor. Additionally, experiments with a GPR3-HaloTag/Nluc mutant with impaired AF64394 binding^4^ (Figure 1e) and with HaloTag/Nluc-based biosensors of the α_2A_- and β_2_-adrenergic receptors (β_2_AR)^22^ confirmed the specificity of the AF64394-induced GPR3 biosensor response (Figure S2). In contrast to the inverse agonist AF64394, the endogenous agonists OEA and oleamide induced concentration-dependent **de**creases in BRET (Figure 1f), confirming that the biosensor reports ligand efficacy. To assess the suitability of this new sensor for medium- to high-throughput ligand screening, we measured its Z′-factor^25^ in four independent experiments, confirming the high sensitivity (mean ± SEM Z′-factor = 0.78 ± 0.02) and low interday variability (coefficient of variation = 6.3%) (Figures 1g and S3). Ultimately, we confirmed the signaling capacity of the GPR3 sensor using a cAMP biosensor^26^ (Figure 1h). These experiments revealed elevated basal levels of cAMP when GPR3-HaloTag/Nluc was coexpressed (Figure 1i) and a concentration-dependent reduction of cAMP by AF64394 (Figure 1j), demonstrating that the GPR3 conformational biosensor has wildtype-like signaling capacity.

**Figure 1:**
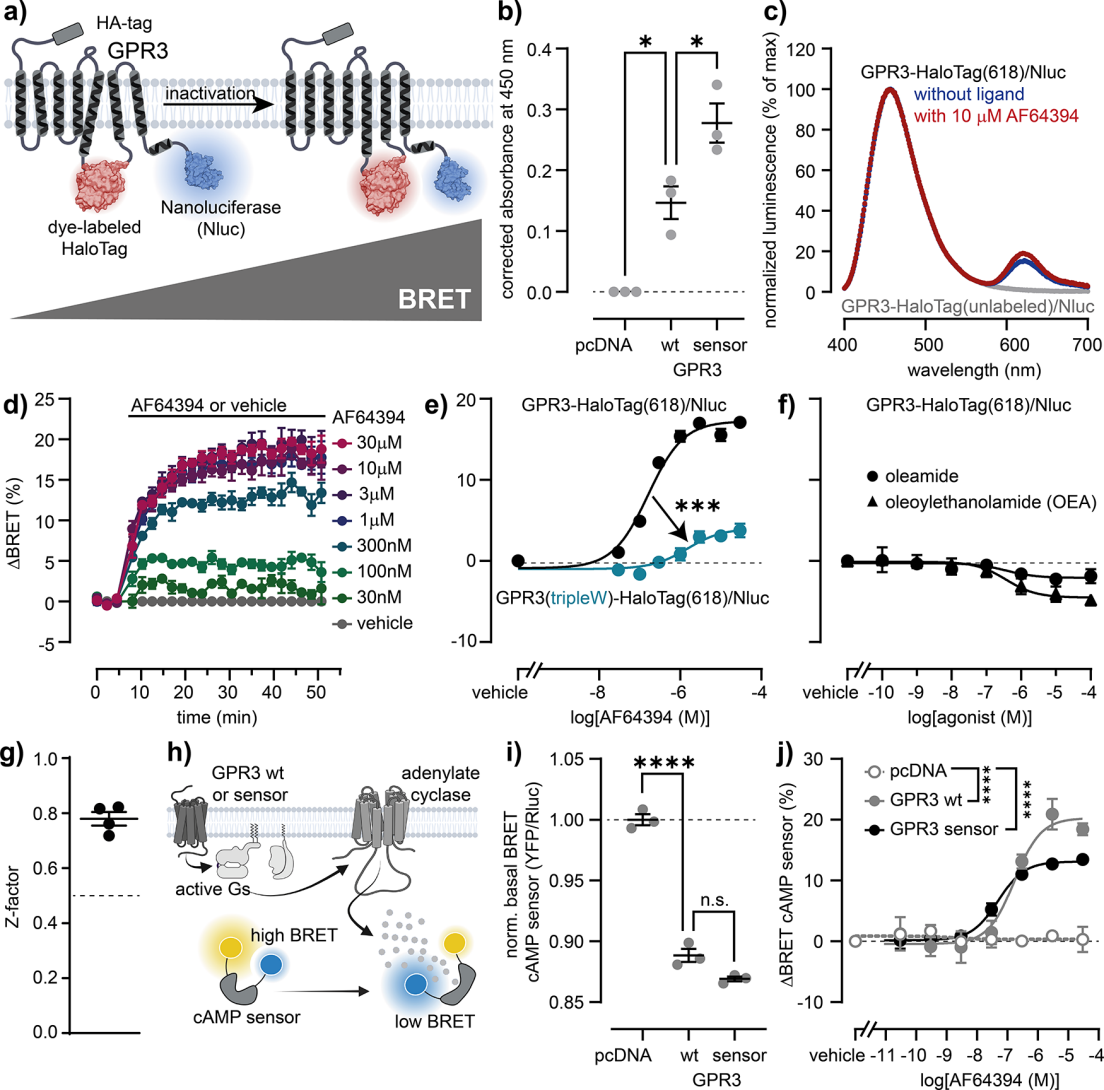
Development of a conformational GPR3 biosensor. (a) Design and principle of the conformational GPR3 biosensor. (b) Surface expression of wildtype GPR3 and the GPR3 biosensor. (c) Luminescence spectra of the HaloTag labeled and unlabeled GPR3 sensor. (d) AF64394-induced ΔBRET time course of the GPR3 biosensor. (e, f) Concentration–response curves of AF64394 (e) or OEA and oleamide (f) obtained with the GPR3 sensor or a mutant variant. (g) Z-factor of the GPR3 biosensor. (h) Scheme of the cAMP assay to assess the signaling capacity of the GPR3 biosensor. (i) cAMP sensor BRET ratio upon coexpression of GPR3 sensor or wildtype. (j) Concentration–response curves of AF64394 obtained with the cAMP biosensor in cells cotransfected with pcDNA, GPR3 wildtype or GPR3 sensor. All experiments were conducted in transiently (b–e, h), (i) or stably (f) expressing HEK293A cells. Data show the mean ± SEM of three to five independent experiments. Statistical significance in (b) and (h) was tested using One-Way ANOVA followed by Tukey′s multiple comparison. Statistical difference of logEC_50_ values in (e) and between the top plateaus in (i) was tested using the extra-sum-of-squares F-test. *: *p* < 0.05; ***: *p* = 0.0002; ****: *p* < 0.0001.

### Virtual Screening for New GPR3 Ligands

To demonstrate the value of this signaling pathway-independent readout of receptor activity for ligand discovery campaigns, we used the conformational biosensor to screen for GPR3 activity modulating compounds. To preselect the compounds to be tested *in vitro*, we conducted a structure-based *in silico* screening using molecular docking calculations.^27^ At the time this study started, no experimental structures of GPR3 or the related receptors GPR6 and GPR12 were available. Hence, we constructed active- and inactive-state GPR3 homology models based on experimental structures of the phylogenetically related cannabinoid receptor 1 (PDB IDs 6N4B and 5TGZ, respectively) (Figure S4a–c). In exploratory docking calculations against the orthosteric pockets of both GPR3 models to assess their utility in a prospective screening, AF64394 and two structural analogs were ranked favorably compared to a randomly selected subset of our virtual compound library (about 700 molecules), indicating that the models were indeed able to recognize these GPR3 ligands (Figure S4d,e). While encouraging, a recent experimental structure of GPR3 in complex with AF64394 shows that this ligand does not bind to the orthosteric pocket^31^, hence we would not have included this step had the project been started today. A library of around 70,000 readily available compounds from the Chemical Biology Consortium Sweden (CBCS) was then docked to each of these structures (about 110,000 entries at pH 7 ± 2). The docking poses of the top 100 (based on the docking score) molecules were visually inspected for each of the docking calculations to the active- and inactive-state GPR3 models. Additionally, the poses of 120 molecules from a consensus list (between the top 1000 molecules of either docking calculation) were evaluated. After this visual inspection followed a clustering of compounds based on 2D similarity to obtain a high structural diversity of compounds to be tested. Finally, 31 and 20 molecules were obtained from the lists resulting from docking to both GPR3 models and 42 compounds were obtained from the consensus list for *in vitro* testing.

### *In Vitro* Testing of Virtual Hits

Initially, all 93 molecules were tested for activity at the GPR3 biosensor at a concentration of 1 μM (Figure 2a). Only three compounds induced a BRET response exceeding the threshold of the mean vehicle response ± 3-fold standard deviation and were subsequently applied at serial dilutions to cells expressing either GPR3- or β_2_AR-HaloTag/Nluc^22^ (Figure S5a–c). All three compounds induced GPR3-specific conformational changes. We hence searched for commercially available derivatives of these three molecules and obtained 14 additional compounds. Among these analogs, two more compounds induced GPR3-specific conformational changes (Figure S5d–f). Of the five compounds that emerged from the virtual screen, three–hereafter referred to as virtual hit 1–3, VH1/2/3–were selected for in-house chemical synthesis (compounds **52**, **93**, and **115**; cf. Scheme 1, Figure S6), providing compounds of ≥99% purity for hit validation (NMR spectra: Figures S7–S142; HPLC purity: Figures S143–S218). For VH1, the racemic mix, rac-VH1/**52**, was synthesized and used for testing. Compounds **52**, VH2/**93**, and VH3/**115** (Figure S6) were then validated with the GPR3 biosensor including β_2_AR-HaloTag/Nluc as a negative control. All three compounds induced concentration-dependent and GPR3-specific conformational changes with potencies ranging from 25 (rac-VH1) to 85 μM (VH3) (Figure 2b–d).

**Figure 2:**
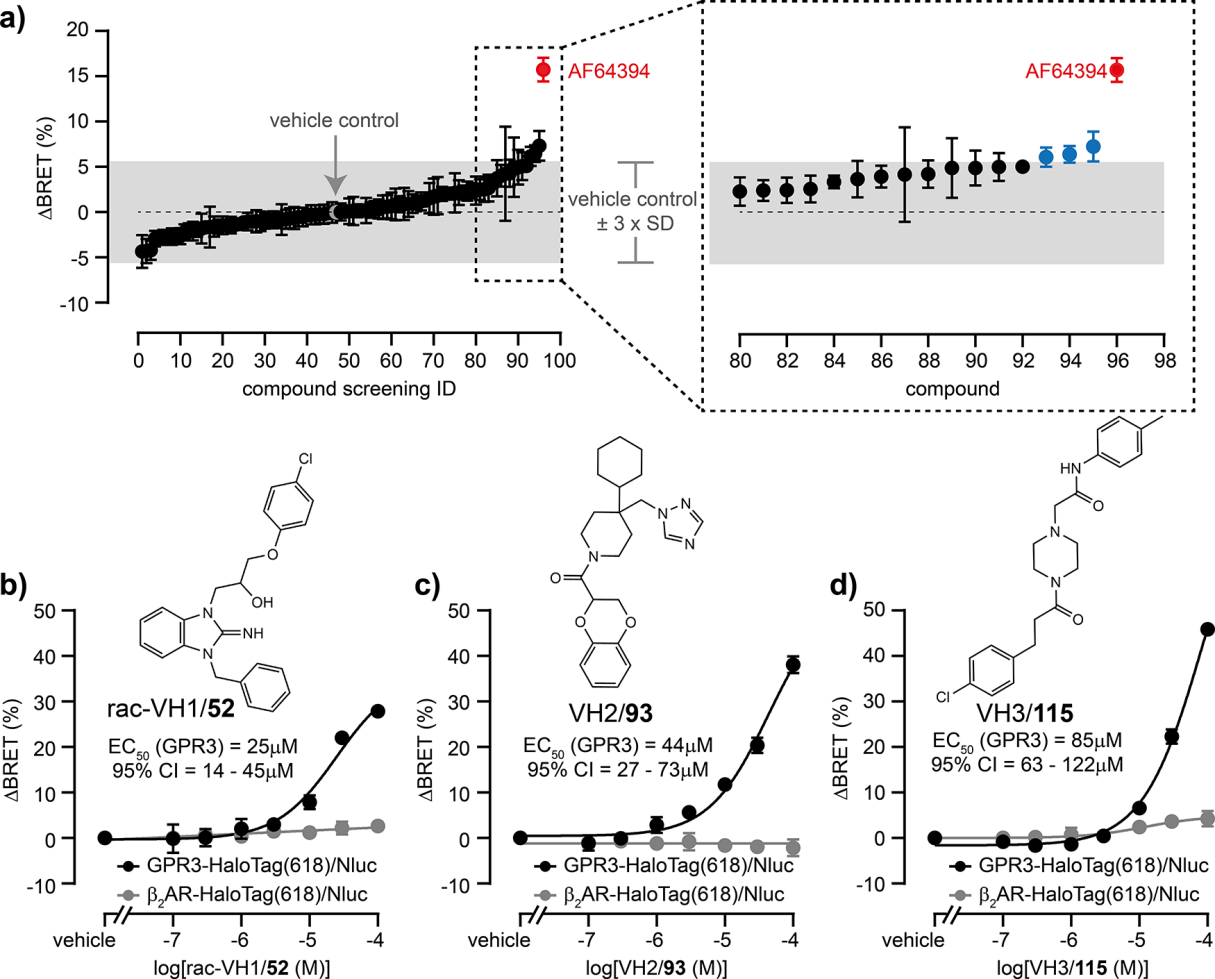
*In vitro* testing of virtual screening hits. (a) BRET changes of GPR3-HaloTag(618)/Nluc induced by 93 test compounds (1 μM), vehicle control (gray) or 10 μM AF64394 (red, positive control). The gray shaded area indicates negative control ± 3-fold SD. Blue data points represent the screening hits VH1, VH2 and VH3. (b–d) Concentration response curves of in-house synthesized rac-VH1/**52**, VH2/**93** and VH3/**115** obtained with the GPR3- or β_2_AR-HaloTag­(618)/Nluc sensor. Data show mean ± SEM of two (a) or three (b–d) independent experiments conducted in HEK293A cells stably expressing the biosensors.

**Scheme 1:**
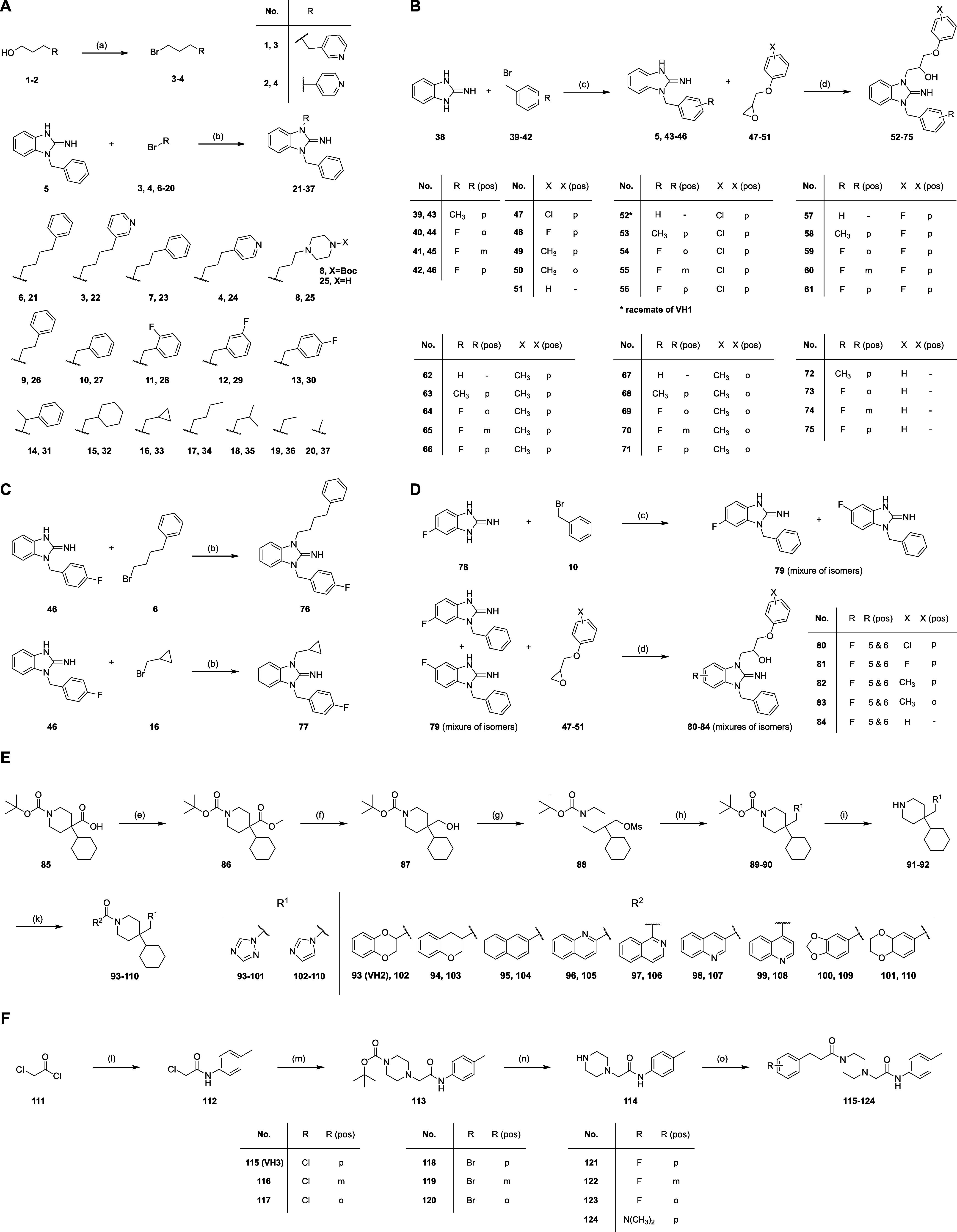
Synthesis of VH1-3 Analogs 21–37 (A), 52–77 (B, C), 80–84 (D), 93–110 (E), 115–124 (F)^a^

a Reagents and conditions: (a) *N*-bromosuccinimide (1.2 equiv), PPh_3_ (1.2 equiv), DCM, 4 h, 0 °C; (b) butan-2-one, 48 h, 90 °C; (c) 20 M NaOH, acetone, 2 h, 60 °C; (d) EtOH, 48 h, 100 °C; (e) CH_3_I (1.2 eq), K_2_CO_3_ (3.0 equiv), DMF, overnight, rt; (f) LiAlH_4_ (2.0 equiv), TFA, 4 d, 0 °C to rt; (g) methanesulfonyl chloride (2.0 equiv), TEA (2.0 equiv), DCM, overnight, 0 °C to rt; (h) sodium salt (3 equiv), DMF, overnight, 110 °C; (i) 20% TFA in DCM, overnight, 0 °C to rt; (k) bicyclic carboxylic acid (2.0 eq), DIPEA (3 equiv), HATU (1.1 eq), DMF, overnight, rt; (l) 4-methylaniline (1 equiv), TEA (2 equiv), DCM, 4–6 h, 0 °C to rt; (m) *tert*-butyl-piperazine-1-carboxylate (3 equiv), K_2_CO_3_ (5.0 equiv), CH_3_CN, 5 h, 80 °C; (n) 50% TFA in DCM, 3 h, rt; (o) 3-phenylpropanoic acid derivative (1 eq), DIPEA (2–3 equiv), HATU (1.2–1.5 eq), TEA (2 eq), DCM, 12 h, −15 °C.

With the first GPR3 ligands identified by using a conformational readout in our hands, we next wanted to understand whether these compounds could have been detected in a cAMP-based assay, which has been used extensively in the past to screen for GPR3 ligands. We therefore tested rac-VH1/**52**, VH2/**93** and VH3/**115** in cells expressing a cAMP biosensor. Interestingly, only rac-VH1 and–to a much lesser extent–VH2 induced GPR3-dependent changes in cAMP concentrations, demonstrating that a cAMP-based screen would likely have incorrectly classified VH3 as inactive (Figure 3).

**Figure 3:**
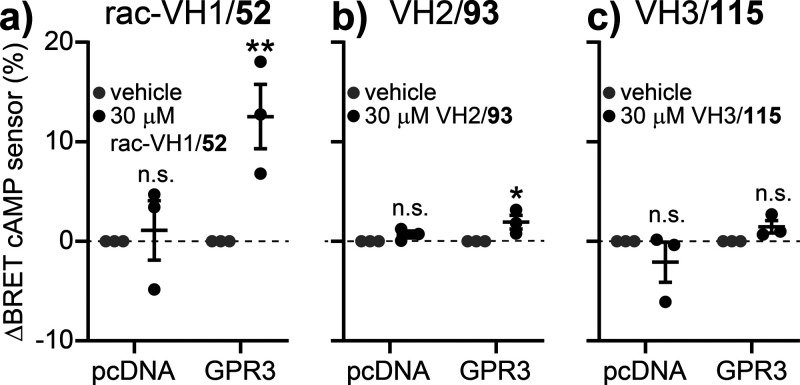
Effect of rac-VH1/**52**, VH2/**93** and VH3/**115** on GPR3-dependent cAMP production. (a–c) Vehicle-corrected BRET changes induced by rac-VH1/**52** (a), VH2/**93** (b) or VH3/**115** (c) in HEK293A cells transiently transfected with a cAMP BRET sensor along with pcDNA or GPR3. Data show mean ± SEM of three independent experiments. Statistical significance was tested using Two-Way ANOVA followed by Sidak multiple comparison (*: *p* < 0.5; **: *p* < 0.01).

Unfortunately, the GPR3-HaloTag/Nluc responses observed with 10 and 100 μM compound treatment suggested that none of the 18 further VH1/2/3-related molecules (c.f. SI for selection) were significantly more potent than the template molecules (Figures S219–S221). Hence, we decided to take this information and used classical medicinal chemistry strategies to derivatize VH1/VH2/VH3 in smaller increments and obtain analogs more similar to the templates (Figure S222). In total, we synthesized 48 different analogs of VH1 (**21–37**, **52–77**, and **80–84**; including rac-VH1/**52**), 17 VH2 analogs (**93–110**) and 9 VH3 analogs (**115–124**) (cf. Scheme 1) with modifications in different regions of the molecule. Details regarding the synthesis and analytical validation of these molecules can be found in the Supporting Information (NMR spectra: Figures S7–S142; HPLC purity: Figures S143–S218) and Scheme 1. All 74 rac-VH1/VH2/VH3 analogs were first tested at two different concentrations, 10 μM and 100 μM, with GPR3-HaloTag(618)/Nluc and the observed BRET changes were compared to those obtained with VH1/2/3 in the same biological replicates (Figures 4a and S223). From these results, promising analogs were selected for full concentration–response experiments at GPR3-HaloTag(618)/Nluc. Three interesting VH1 derivatives emerged from this analysis: While compounds **56** and **80** showed only small left-shifts of the concentration–response curves (*i*.*e*., higher potency) compared to the template molecule rac-VH1/**52** (Figure 4b,c), **33** showed a more pronounced, approximately 6-fold, improvement in ligand potency (Figure 4d) and selectivity for GPR3 over β_2_AR (Figure 4e). Structurally, **33** shares very little similarity with the known GPR3 inverse agonist AF64394 (atom pair Tanimoto = 0.22; maximum common substructure Tanimoto = 0.20 using PubChem fingerprints as implemented on chemminetools.ucr.edu^28^), underscoring the novelty of this compound as a GPR3 ligand.

**Figure 4:**
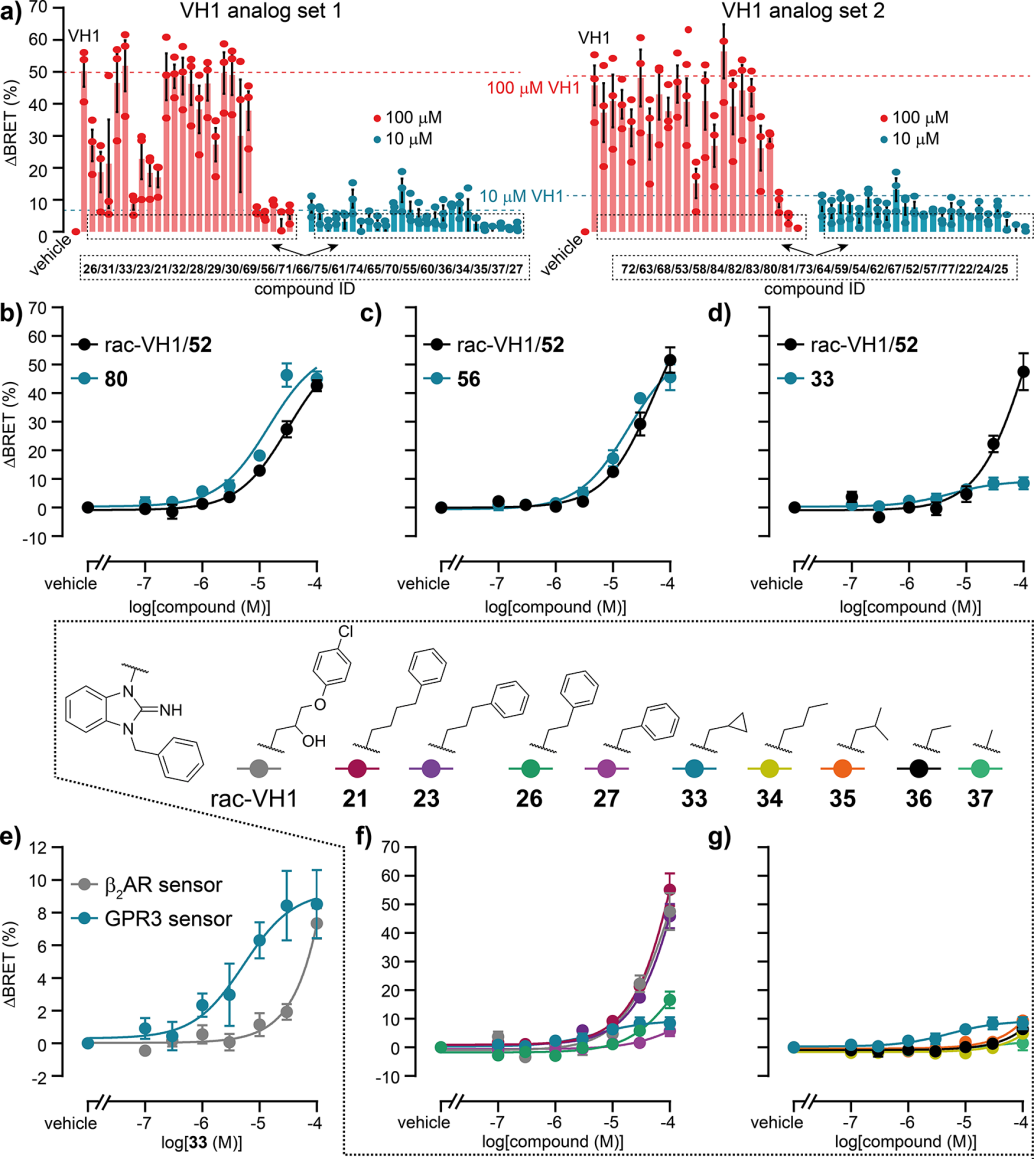
*In vitro* testing of VH1 analogs. (a) BRET changes of GPR3-HaloTag(618)/Nluc induced by vehicle control, rac-VH1/**52** and 49 chemical analogs. (b–d) Concentration response curves of VH1 analogs **80** (b), **56** (c) and **33** (d) obtained with the GPR3-HaloTag(618)/Nluc sensor. (e) Comparison of the **33** response at GPR3- vs β_2_AR-HaloTag­(618)/Nluc sensor. (f, g) Concentration response curves of rac-VH1/**52** and its analogs **21**, **23**, **26**, **27**, **33**, and **34**–**37** obtained with the GPR3-HaloTag(618)/Nluc sensor. Data show mean ± SEM of three independent experiments conducted in HEK293A cells stably expressing the indicated biosensor.

The EC_50_ value of **33** at the GPR3 conformational biosensor was 5 μM (95% CI: 1–19 μM). Strikingly, **33** showed a reduced amplitude in the GPR3 conformational change readout (which was still robustly reproducible in all of our independent experiments; Figure S224), indicating that the modification from rac-VH1/**52** to **33** caused a loss in inverse agonist efficacy while enhancing ligand affinity. To obtain deeper insights into the structure–activity relationship of rac-VH1/**52** and **33**, we further collected concentration–response data for other VH1 derivatives of this analog series (Figure 4f,g). Our analysis revealed that the hydroxy and ether-groups, as well as the *para* chlorine substituent are dispensable for VH1 action because both **21** and **23** showed similar efficacy as rac-VH1/**52**. In addition, the reduced BRET response obtained with **26** and **27** indicates that the loss in inverse agonist activity for **33** could be due to the shortening of the linker between the central heterocyclic system and the “upper” aromatic ring (Figure 4f). This effect of side chain truncation was, however, strictly dependent on the cyclopropyl substituent, because none of the other truncated, saturated VH1 analogs (**34**–**37**) exhibited the low micromolar potency observed with **33** (Figure 4g).

To develop a hypothesis for the cause of the significant loss in efficacy from VH1 to **33**, we computationally predicted the binding poses of both molecules in GPR3 (Figure 5a). Our docking suggests that the *para*-substituted phenyl ring of VH1 and its linker extend toward the extracellular space without making any contacts with the receptor. In contrast, the cyclopropyl ring of **33** is still buried in the receptor’s transmembrane core. Additionally, further energy minimization of the GPR3 ligands revealed that compound **33**, but not VH1, can adopt an energetically favorable binding pose that is oriented closer toward transmembrane helix 2 (Figure S225). Notably, these computationally predicted poses of VH1 and **33** were supported by experimental data with mutant variants of the GPR3 showing wildtype-like surface expression levels.^4^ While the insertion of bulky tryptophan residues in extracellular loop 2 right-shifted the rac-VH1/**52** concentration–response at the GPR3 biosensor (Figure 5b), **33** was not affected by these modifications (Figure 5c). We also challenged this binding model of **33** with another GPR3 biosensor variant which harbors the T^3.37^A point mutation in transmembrane helix 3. According to an alternative predicted pose of **33** obtained with Boltz-2 (c.f., SI – Materials and Methods section), this mutation should hamper **33** binding to GPR3. However, our experiments with wildtype and mutant GPR3 biosensor showed no such effect on **33** activity (Figure S226). Taken together, these results support the binding pose depicted in Figure 5 and suggest that **33** binds to GPR3 in a different manner compared to VH1, which may relate to the mechanistic basis for the apparent reduction in inverse agonist efficacy from VH1 to **33**.

**Figure 5:**
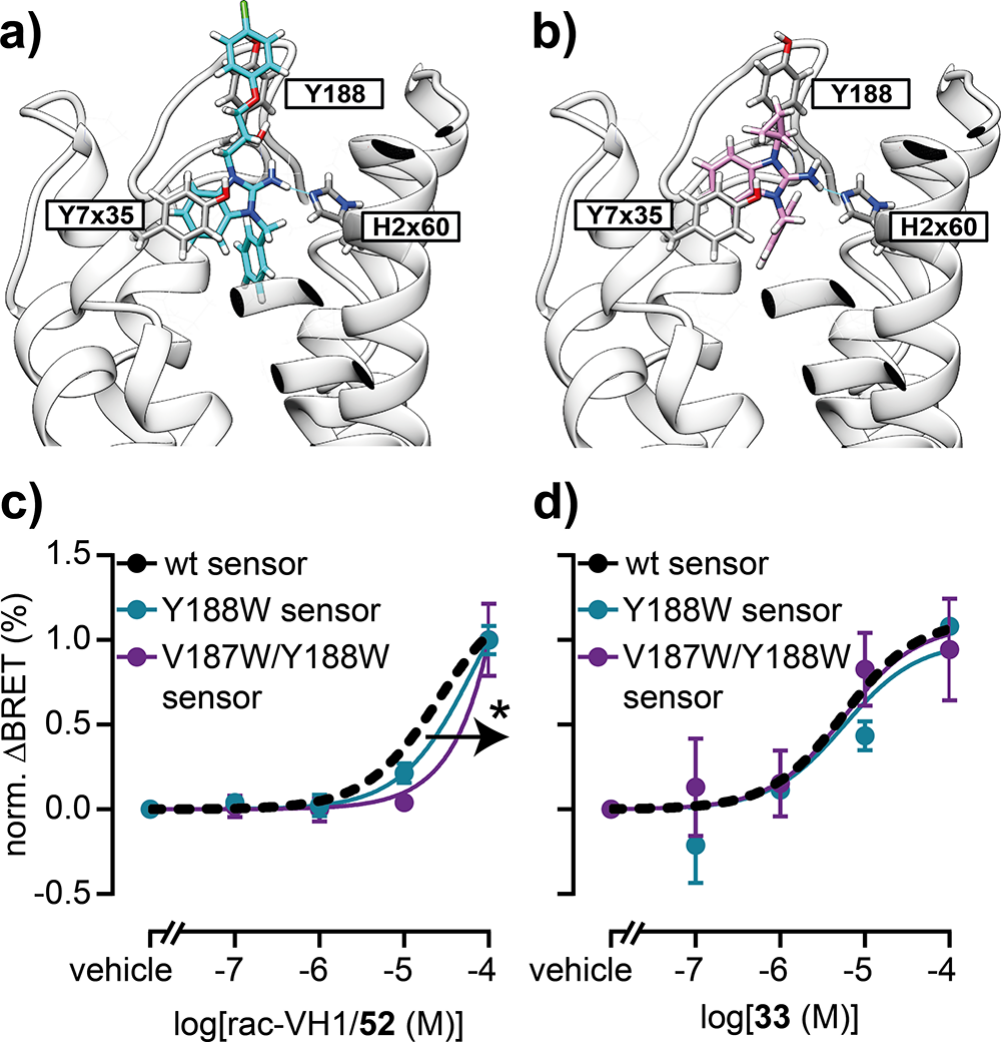
Proposed binding poses of VH1 and **33**. (a, b) Computationally predicted binding poses of VH1 (a) and **33** (b) in GPR3 (model based on PDB 8X2K). (c, d) Concentration response curves of rac-VH1/**52** (c) and **33** (d) obtained with wildtype and mutant GPR3 conformational sensors. Data show the mean ± SEM of three independent experiments conducted in transiently transfected HEK293A cells. Statistical difference of the pEC_50_ values was tested using extra-sum-of-squares F-test (*p < 0.05).

### Pharmacological Characterization of **33**

To further delineate the pharmacological properties of **33**, we evaluated its activity in signaling assays downstream of GPR3. When **33** was applied to HEK293A cells expressing our G_s_ dissociation biosensor, Gs-CASE,^29^ a concentration- and GPR3-dependent increase in BRET was observed with an EC_50_ value of 250 nM (95% CI: 13 nM–3 μM) (Figure 6a,b). These data confirm that **33** is an inverse agonist of GPR3. We also investigated whether **33** blocks the activity of previously validated GPR3 ligands–the agonist DPI and the inverse agonist AF64394 (Chart 1)–in a cAMP assay.^30^ Intriguingly, **33** had no effect on the effect of AF64394 (Figure 6c), which is consistent with a recent experimental GPR3 structure showing nonorthosteric binding of AF64394 ^31^ but inhibited DPI-induced cAMP generation (Figure 6d). These data support our previous observation that DPI and AF64394 engage distinct sites in GPR3 ^4^ and suggest that **33** competes with DPI for GPR3 binding. Additionally, we observed reduced potency of OEA, but not oleamide, in the GPR3 conformation assay following preincubation with **33** (Figure S227). The differential effect between OEA and oleamide may reflect differences in their conformational and/or positional flexibility within the GPR3 binding pocket. This difference is supported by MD simulations of oleamide in GPR3 ^1^ and by a comparison of oleic acid binding poses in the highly similar pockets of GPR3 and GPR6.^2,32^

**Figure 6:**
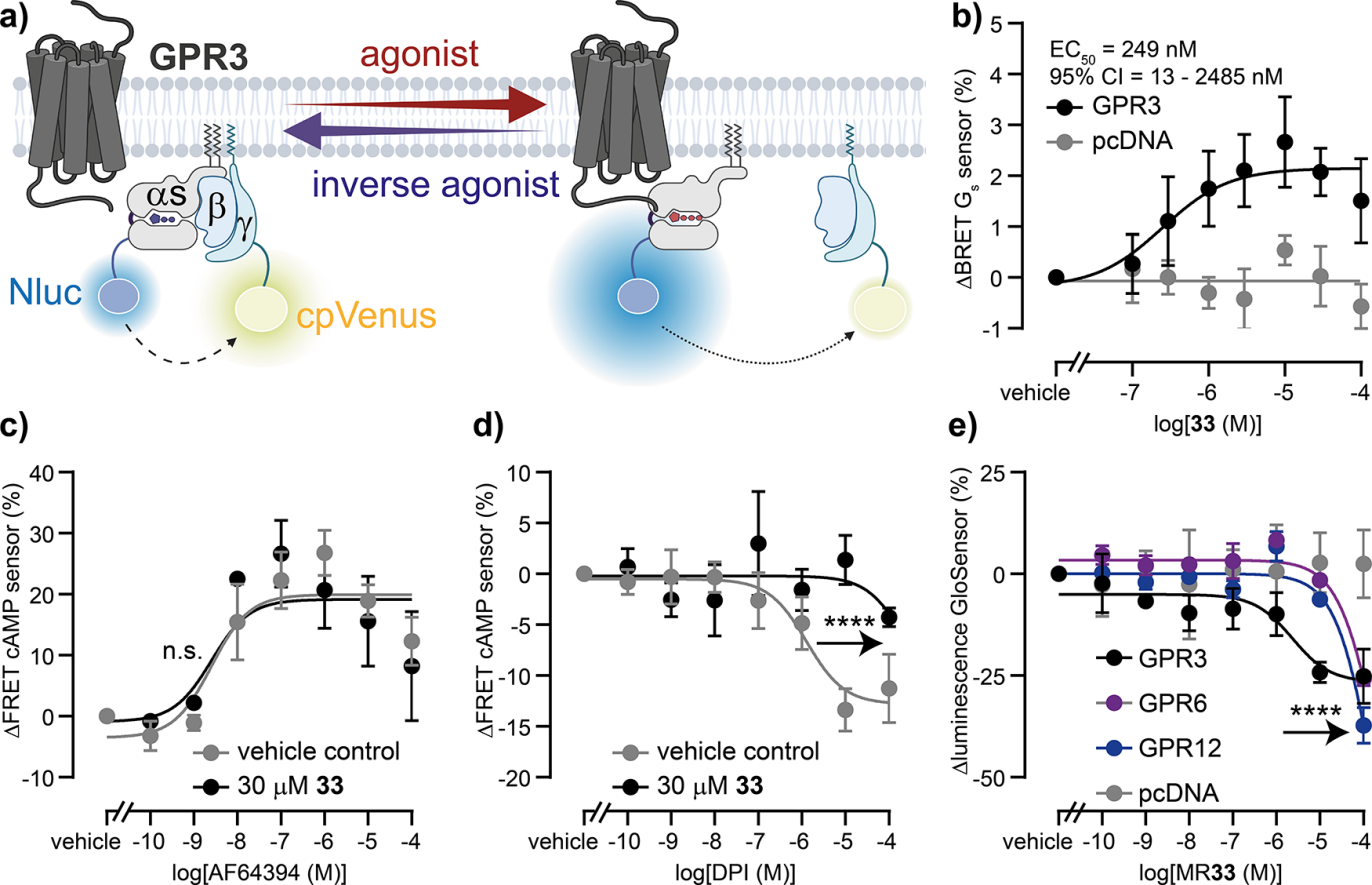
Pharmacological characterization of compound **33**. (a) Schematic of a live-cell G_s_ heterotrimer dissociation/reassociation assay to assess GPR3-dependent G_s_ activity. (b) Concentration response curves of **33** obtained with the G_s_ dissociation sensor in cells cotransfected with GPR3 or empty vector control (pcDNA). (c, d) Concentration response curves of AF64394 (c) and DPI (d) obtained with a cAMP biosensor in cells coexpressing GPR3 and pretreated with 30 μM **33** or vehicle control. (e) Concentration response curves of **33** obtained with the cAMP GloSensor in cells cotransfected with GPR3, GPR6, GPR12 or empty vector control (pcDNA). Data show mean ± SEM of three (c–e) or four (b) independent experiments conducted in HEK293A cells transiently expressing the indicated proteins.

Next, we used the classical cAMP GloSensor technology to assess the target selectivity profile of **33**. Addition of **33** to the constitutively G_s_-coupled receptors GPR6 and GPR12–the closest phylogenetic relatives of GPR3–revealed no receptor-specific changes in cAMP levels below 100 μM **33**, while a concentration-dependent decrease in cAMP was observed in GPR3-expressing cells with an EC_50_ value of 2 μM (95% CI: 0.2–17 μM; Figure 6e). In addition, we tested the effects of **33** on cAMP changes mediated by agonist-stimulated GPCRs phylogenetically close to GPR3 and coupling to G_s_ or G_i/o_ proteins. Preincubation with neither 100 μM nor 10 μM **33** significantly altered the agonist-induced cAMP change mediated by the adenosine A_2B_ receptor (A_2B_R), the cannabinoid receptor 1 (CB_1_R), the S1P receptor 1 (S1PR1) or the LPA receptor 1 (LPAR1) (Figure S228). In summary, these data imply that **33** is selective for GPR3 among the seven receptors included in this selectivity profiling.

### Physicochemical Properties of **33**

Finally, we assessed the physicochemical properties of **33** using the SwissADME Swiss Drug Design online tool.^33^ This analysis indicated that **33**, with a molecular weight of only 277 g/mol, a calculated Log-P of 2.9, no Lipiniski rule violations, zero PAINS alerts, high leadlikeness and high synthetic accessibility (2.4 on a scale ranging from 1–10/highly accessible-not accessible), provides ample room for synthetic expansions and modifications in future campaigns aiming for more potent and efficacious **33** analogs.

## Conclusions

GPR3 is a class A GPCR that holds great potential for the development of treatments against severe human diseases. However, the therapeutic exploitation of this target is hampered by a very limited number of available GPR3 ligands. To fill this gap, we have developed an analytical tool that enables the discovery and characterization of new GPR3 ligands. Our biosensor detects ligand-induced conformational changes in GPR3 and allowed us to identify and optimize new ligands by combining this advanced analytical tool with virtual compound screening and classical medicinal chemistry. Our virtual screening approach based on compound docking to 3D models of GPR3 revealed three chemically novel inverse agonists of GPR3, VH1–3. Subsequent synthesis of more than 70 VH1/2/3 analogs allowed us to further improve the potency of VH1 and identify one of its chemical analogs, **33**, as a GPR3 ligand with low micromolar potency in the conformational readout (EC_50_ = 5 μM) and submicromolar potency in a signaling-based G protein reassociation assay (EC_50_ = 249 nM). Additionally, mutagenesis studies and competition experiments with two known GPR3 ligands provided insights into the binding mode of **33** at GPR3. In the future, **33** may, due to its favorable chemical characteristics, serve as a useful lead structure for the development of advanced GPR3 inverse agonists that could aid in the treatment of severe, GPR3-dependent diseases such as Alzheimer’s disease. Its low molecular weight, balanced physicochemical properties and high synthetic accessibility allows for extensive chemical derivatization toward further advanced GPR3 inverse agonists.

Collectively, our results demonstrate the power of structure-based ligand discovery pipelines including readily applicable conformational GPCR biosensors. Using such tools, the modulation of receptor activity can be detected in a downstream signaling pathway-independent–and therefore unbiased–manner, reducing the risk of false screening results. This advantage is particularly relevant for poorly studied targets, *e*.*g*., orphan GPCRs with often unknown signaling patterns, as exemplified by the discovery of VH3/**115**. VH3/**115** modulates the conformation of GPR3 but not GPR3-dependent cAMP production. This provides evidence for an unproductive GPR3 conformation that is stabilized by VH3 or GPR3 signaling via G_s_- and cAMP-independent pathways, possibilities that require further investigation.

## Supplementary Material





## Notes

Source information and SMILES for all compounds (XLSX)
Materials and methods section, amino acid sequence of the GPR3 biosensor, supporting experimental data, GPR3 homology model snapshots and validation data, and chemical synthesis information supplemented with the analytical data of new compounds (PDF)

## ◆

Orion Pharma R&D, Orionintie 1A, Espoo 02101, Finland

## ¶

St. Jude Children’s Research Hospital, Memphis, Tennessee 38105, United States

## Abbreviations

β_2_AR: β_2_-adrenergic receptors
BRET: bioluminescence resonance energy transfer
CBD: cannabidiol
CNS: central nervous system
DPI: diphenyleneiodonium chloride
FRET: fluorescence resonance energy transfer
GPCR: G protein-coupled receptor
HEK293: human embryonic kidney
LPA: lysophosphatidic acid
Nluc: Nanoluciferase
S1P: sphingosine-1-phosphate (S1P)
