# Supplementary material for: GPR3 Ligands Discovered through Combined Virtual and Conformational Biosensor-Based Screening
Source: J Am Chem Soc. 2026 May 7;148(19):20285–95. doi: 10.1021/jacs.6c06780 (PMC13195669; doi:10.1021/jacs.6c06780)
Supplement: Supplementary file 2 [file ja6c06780_si_002.pdf]

Supporting information for
GPR3 ligands discovered through combined virtual and conformational biosensor-based screening

Hannes Schihada*[a], Aida Shahraki[a] ‡, Ainoleena Turku-Metsänen[b] ‡,§, Maximilian Rath[c], Lukas Wirth[c], Katarina Nemec[d] ¶, Hrisowalantu Tselepli[c], Laura Heitzer[c], Bernadette Vallaster[c], Mariam Fadel[a], Gunnar Schulte[b], Daniel Hilger[a], Steffen Pockes[c], Martin J. Lohse*[e, f], and Peter Kolb*[a]

[a] Philipps-Universität Marburg, Institute of Pharmaceutical Chemistry, Marbacher Weg 8, 35032 Marburg, Germany

[b] Section of Receptor Biology & Signaling, Department of Physiology and Pharmacology, Karolinska Institutet, 171 77 Stockholm, Sweden

[c] Institute of Pharmacy, University of Regensburg, Universitätsstraße 31, 93053 Regensburg, Germany

[d] Max Delbrück Center for Molecular Medicine in the Helmholtz Association, 13125 Berlin, Germany

[e] ISAR Bioscience Institute, 82152 Planegg/Munich, Germany and TU München Klinikum Rechts der Isar, 81675 Munich

[f] Rudolf-Boehm-Institute for Pharmacology and Toxicology, Faculty of Medicine, Leipzig University, Härtelstrasse 16-18, 04107 Leipzig, Germany.

‡These authors contributed equally.
§ A.T. Present address: Orion Pharma R&D, Orionintie 1A, Espoo, 02101, Finland
¶ K.N. Present address: St. Jude Children's Research Hospital, Memphis, TN, 38105, United States

* corresponding authors; hannes.schihada@uni-marburg.de; martin.lohse@isarbioscience.de; peter.kolb@uni-marburg.de

Materials and Methods

Homology modelling and model selection

To select templates for homology modelling, we conducted an NCBI BLAST [1] search on Protein Data Bank proteins (as of October 2019). Two template proteins with three 3D structures were highlighted by the search: Sphingosine 1-phosphate receptor 1 (PDB ID: 3V2Y) and cannabinoid receptor 1 (PDB IDs: 5TGZ and 6N4B). We built 20 homology models based on each template using MODELLER[2], and prepared them with the Protein Preparation tool of Schrödinger Maestro (release: January 2019, Schrödinger, LLC, New York, NY) for further calculations. The models were examined further by docking the reference ligand AF64394 to their orthosteric binding sites using three docking grids: one for the active receptor conformations and two for the inactive ones. The two former grids were located close to the extracellular region of the receptor, including e.g. amino acid H2.60, whereas the latter grid was deeper in the binding cavity close to e.g. amino acid W6.48. For each model, ten ligand poses were produced. Schrödinger's Glide was utilized for the molecular docking and LigPrep was used for ligand preparation.

The docking poses were clustered based on the receptor-ligand interaction fingerprints (Schrödinger Canvas; release: January 2019, Schrödinger, LLC, New York, NY) and subsequently, the representative models were visually examined. Our in-house point mutation data showed that amino acid F3.36 plays a role in AF64394 binding and, thus, we included only those models where this interaction was present. Based on these steps, we selected two cannabinoid receptor 1-based models for the virtual screening – one model represented an inactive receptor conformation (template: 5TGZ) and another an active receptor conformation (template: 6N4B).

Structure- and ligand-based virtual screening

The selected receptor-ligand models were further optimized utilizing the InducedFit docking protocol of Schrödinger Maestro (release: January 2019, Schrödinger, LLC, New York, NY). The amino acid residues within 3 Å of the originally docked AF64394 poses were selected for the energy minimization, otherwise the standard parameters were used. The resulting binding poses were in good agreement with the original models. Next, the screening power of the optimized models was examined by docking a random subset of 1% of the screening library seeded with the known reference ligand AF64394 and two closely related derivatives utilizing Schrödinger Glide with the standard SP parameters. The docking grid was defined based on the location of the ligand in these models. All reference compounds were retrieved in the top 5% of the library by the 5TGZ-based model and in the top 10% of the library by the 6N4B-based model. Of note, only AF64394 and two structurally analogous reference molecules were reported in the literature at the time of the screening, and thus the enrichment results likely overestimate the screening power.

We screened a compound collection provided by the Chemical Biology Consortium Sweden (CBCS) consisting of approx. 70 000 compounds. The compounds were prepared using LigPrep with Epik at pH 7±2. The docking was conducted similarly to that of the enrichment study. The top-ranked 100 compounds from each receptor model and 120 compounds which were among the top-ranked 1000 compounds in *both* models, were clustered (Canvas linear fingerprints and average linkage) and visually examined leading to a list of 96 compounds.

For ligand-based virtual screening, individual conformers of approximately half a million ZINC20 in-stock molecules (prefiltered based on physicochemical properties) were aligned to 400 distinct conformers of VH1-3 using Rapid Overlay of Chemical Structures (ROCS; OpenEye Scientific[3]) based on 2D similarity, 3D shape and positioning of functional groups. 18 molecules were selected for testing after statistical analysis of the similarity and visual inspection of the most similar molecules.

Structure preparation and docking calculations of derivatives

For docking the derivative compounds of the initial hits, the 3D structure of the GPR3 (PDB ID 8X2K) was prepared using the protein preparation menu of Molecular Operating Environment (MOE) software, which is an integrated software package for drug discovery MOE version 2013.08, Chemical Computing Group ULC, 910-1010 Sherbrooke St. W., Montreal, QC H3A 2R7, Canada, 2016.).

DOCK3.7[4] was used for obtaining the poses of the final hits to the GPR3 structure. The required inputs for running docking in DOCK, including the receptor with polar hydrogens, without hydrogens and a co-crystallized ligand in pdb format were prepared in MOE. All the poses were subjected to energy minimization using the MMFF94x force field, a variant of MMFF94 (Merck Molecular force field), which is suitable for minimizing protein-ligand complexes[5] in MOE. Poses were visually inspected for the satisfaction of polar contacts, lack of intramolecular strain, absence of clashes with the receptor, etc.

To explore other potential binding modes of molecule **33**, we used Boltz-2 (https://github.com/jwohlwend/boltz), which is a co-folding method[6]. Choosing the "Protein-Ligand Complex" mode, the protein sequence of GPR3 (uniport ID: P46089) and the SMILES of compound **33** were provided as chain A and B, respectively. "Number of Samples", which corresponds to the generated poses was set to 5, and "Number of Recycles", which is the cycles of structure refinement, was set to 3. No method

conditioning and no constraints were applied. The poses were visually inspected and the most favorable one that coincides with the co-solved ligand according to the available structures was chosen.

Plasmids and molecular cloning
The H187-EPAC-FRET sensor[7] was kindly provided by K. Jalink (The Netherlands Cancer Institute, Amsterdam, The Netherlands). The BRET-based cAMP sensor CAMYEL[8] was kindly provided by Laura Humphrys (University of Regensburg, Regensburg, Germany). Plasmids encoding the conformational biosensors α_{2A}AR- and β_2AR-HaloTag/Nluc[9] and the Gs heterotrimer dissociation sensor[10] were previously described. The expression plasmid encoding N-terminally HA-tagged GPR3 (cat. no. #GPR003TN00) on a pcDNA3.1+ vector was obtained from the cDNA resource center (cdna.org). The Nluc and HaloTag inserts were amplified with overhanging restriction sites from β_{2A}AR- HaloTag/Nluc and ligated to the full-length C-terminus or inserted into icl3 of GPR3, respectively, to obtain the conformational biosensor GPR3-HaloTag/Nluc. Point mutants of GPR3-HaloTag/Nluc were generated using the GeneArt Site-directed Mutagenesis System (Thermo Fisher Scientific). All constructs were verified by sequencing (Eurofins genomics).

Reagents
Poly-D-lysine, polyethylenimin hydrochloride (linear; average Mn 20 000; PEI), CP 55,490, LPA, forskolin, NECA and bovine serum albumin (BSA) were obtained from Sigma Aldrich (Merck KGaA). Dulbecco's Modified Eagle's Medium (DMEM), Dulbecco's Phosphate-Buffered Saline (DPBS) and G-418 (Geneticin) were from Gibco. Diphenyleneiodonium chloride (DPI) and AF64394 were purchased from Tocris (Bio-Techne). The TMB (3, 3', 5, 5' tetramethyl benzidine) substrate reagent set was from BioLegend. The Nluc substrate furimazine and the red fluorescent HaloTag dye, HaloTag® NanoBRET® 618 Ligand, were obtained in a single kit from Promega (cat. no. N1662). D-luciferin-sodium and the S1PR1 agonist III were from MedChemExpress. The Rluc substrate coelenterazine 400a was from NanoLight Technology (cat. no. 340-10). White-wall, white-bottomed 96-well, black-wall, black bottomed and transparent 96-well microtiter plates were from Brand.

Cell culture
HEK293A cells were used for transient transfection and grown in Dulbecco's modified Eagle's medium (DMEM) supplemented with 2 mM glutamine, 10 % fetal calf serum, streptomycin (0.1 mg/mL), and penicillin (100 U/mL) at 37 °C with 5 % CO_2. For the generation of stable GPR3-HaloTag/Nluc cells, HEK293A cells grown in T75 flasks were transfected at a confluence of 40–50 % with 1 µg of DNA. To select for stably expressing cells, transfected cells were cultured with 2000 µg/mL of G-418 and maintained in fully supplemented DMEM containing 500 µg/mL G-418. Absence of mycoplasma contamination was routinely confirmed by PCR.

Transient transfection and plating
Resuspended cells (300,000 cells/mL) were transfected in suspension with a total of 1 µg DNA/mL suspension using PEI (1 mg/ml stock solution; 3 µL PEI solution per µg DNA). For cAMP experiments with FRET- or BRET-based sensors, resuspended cells were transfected with 500 ng pcDNA, wildtype GPR3 or GPR3-HaloTag/Nluc constructs along with 500 ng of the FRET- or BRET-based cAMP sensor per mL cell suspension. For cAMP experiments with GloSensor™ 22F, resuspended cells were transfected with 250 ng pcDNA or GPCR along with 500 ng of a 1:1 mix of pcDNA with the GloSensor™ 22F per mL cell suspension. Cells mixed with the transfection reagents were seeded onto poly-D-lysine–precoated, 96-well plates and grown for 48 h at 37 °C with 5 % CO_2. Stable α_{2A}AR-, β_2AR- or GPR3-HaloTag/Nluc expressing cells were seeded 24 h before the experiment at a density of 800,000 cells/mL into white 96-well plates. To monitor changes in BRET in with conformational GPCR sensors, cells were supplemented with 500 nM of the HaloTag dye HaloTag® NanoBRET® 618 Ligand the day before the experiment. White plates were used for BRET and luminescence measurements, black plates for FRET experiments and transparent plates for absorbance-based quantification of receptor surface expression levels.

Recording of Nluc-GPR3 luminescence spectrum
HEK293A cells stably expressing GPR3-HaloTag/Nluc were seeded as described above. Luminescence emission was recorded between 400 and 700 nm with 5 nm resolution in Hank's balanced salt solution (HBSS) upon addition of 1:1000 furimazine dilution. All experiments were conducted using a CLARIOstar plate reader (BMG, Ortenberg, Germany) and spectra were normalized to the *donor emission peak.*

Quantification of receptor surface expression levels
Transfected cells grown in transparent 96-well plates were washed once with 200 µl of HBSS 48 h after transfection and incubated with a 1/1000 stock solution of the primary HA-tag antibody (Cell Signaling; cat.# 3724) dissolved in ELISA buffer (1% w/m bovine serum albumin in Ca^{2+}-supplemented DPBS) for 1h at 4°C. Subsequently, cells were washed five times with ice-cold washing buffer (0.5% w/m bovine serum albumin in Ca^{2+}-supplemented DPBS) and then incubated with 1/2500 stock solution of the HRP-labeled secondary antibody (Cell Signaling; cat.# 7074= for 1h at 4°C. Following five more washing steps, 50 µl of the TMB substrate solution was added to each well and incubated at room temperature in the dark for 30 minutes. After the addition of 2M HCl, the absorbance was read at 450 nm using the CLARIOstar plate reader.

BRET-based GPCR conformational sensor experiments

Cells expressing the conformational GPCR biosensors were grown for 24 h (stable cells) or 48 h (transiently transfected cells) in white 96-well plates and washed with 100 µl of HBSS. After washing, cells were incubated for 2 minutes with a 1/1000 solution of the furimazine stock solution (Promega; cat. no. N1662). Three baseline BRET reads were recorded and averaged prior to the addition of serial dilutions of ligands or vehicle control and subsequent BRET reads. All experiments were conducted at 37 °C. BRET donor emission was quantified with a 470/80 nm monochromator. BRET acceptor emission was quantified using a 660/100 nm monochromator. Experiments were carried out using a CLARIOstar plate reader.

FRET- and BRET-based measurements of cAMP levels and G_s dissociation/reassociation

Transfected cells grown in black 96-well plates were washed twice with 100 µl of HBSS 48 h after transfection and incubated with HBSS. For G_s dissociation BRET measurements, HBSS was supplemented with a 1/1000 solution of the furimazine stock solution. For BRET-based cAMP measurements, HBSS was supplemented with a 5 µM coelenterazine 400a. Three baseline FRET/BRET reads were recorded and averaged prior to the addition of serial dilutions of ligands or vehicle control and subsequent FRET/BRET reads. All experiments were conducted at 37 °C. For FRET, cells were excited using a 430/10 nm filter combined with 458 nm and 504 nm longpass filters to separate excitation from donor and acceptor emission light. FRET donor emission was quantified with a 480/10 nm filter. FRET acceptor emission was quantified using a 530/10 nm filter. For BRET measurements, donor emission was quantified with a 400/80 nm monochromator. BRET acceptor emission was quantified using a 525/70 nm monochromator. Experiments were carried out using a CLARIOstar plate reader.

Luminescence measurement using the cAMP GloSensor™ 22F

Cells co-expressing the cAMP GloSensor™ and the receptor of interest were grown for 48 h in white 96-well plates and washed with 100 µl of HBSS. After washing, cells were incubated for 20 minutes with 1 mM D-luciferin-sodium. For experiments with $A_{2B}R$, CB_1R, S1PR1 and LPAR1, 10 or 100 µM **33** was added with (CB_1R, S1PR1 and LPAR1) or without ($A_{2B}R$) 10 µM forskolin five minutes prior to the start of the experiment. Three baseline luminescence reads were recorded and averaged prior to the addition of **33** (to pcDNA-, GPR3-, GPR6- and GPR12-transfected cells), GPCR agonist (to $A_{2B}R$-, CB_1R-, S1PR1- and LPAR1-transfected cells) or vehicle control, and subsequent luminescence reads. All experiments were conducted at 37 °C. Luminescence emission was quantified using a 580/80 nm monochromator. Experiments were carried out using a CLARIOstar plate reader.

Data analysis

FRET and BRET ratios were defined as acceptor emission/donor emission. The first ratio of a time-course experiments was defined as $Ratio_{basal}$. To quantify ligand-induced changes ΔFRET and ΔBRET were calculated for each well as a percent over basal ([($Ratio_{stim}$ − $Ratio_{basal}$)/$Ratio_{basal}$] × 100). Subsequently, the average ΔFRET/BRET of vehicle control was subtracted. Data from FRET/BRET experiments were fitted using a three-parameter fit. *Z*-factors expressing the high-throughput suitability were calculated with the following equation:

$$Z - factor = \frac{(3 * \sigma s + 3 * \sigma c)}{(\mu s - \mu c)}$$

where σs and σc are the standard deviations of ΔFRET or ΔBRET. μs and μc express the mean of ΔBRET values of AF64394- and vehicle-treated wells, respectively. Data shown are mean values ± SEM of N independent experiments. Data were analyzed using Prism 5.0 software (GraphPad, San Diego, CA, USA).

General chemical procedures

Commercially available chemicals and solvents were purchased from standard commercial suppliers (Merck (Darmstadt, Germany), Sigma-Aldrich (Munich, Germany), Acros Organics (Geel, Belgium), Alfa Aesar (Karlsruhe, Germany), abcr (Karlsruhe, Germany), BLD Pharmatech (Reinbek, Germany) or TCI Europe (Zwijndrecht, Belgium) and were used as received. Deuterated solvents for nuclear magnetic resonance (^{1}H NMR and ^{13}C NMR) spectra were purchased from Deutero GmbH (Kastellaun, Germany). All reactions carried out with dry solvents were accomplished in dry flasks under nitrogen or argon atmosphere. For the preparation of buffers, HPLC eluents, and stock solutions millipore water was used. Column chromatography was accomplished using Merck silica gel Geduran 60 (0.063-0.200 mm). Flash chromatography was performed using an Advion Interchim puriFlash® XS 520 Plus purification system (Advion, Ithaca, NY; Interchim, Montluçon, France) with SI-HP 30 µm and SI-SDT-50 µm puriFlash columns from Interchim (Montlucon, France). The reactions were monitored by thin layer chromatography (TLC) on Merck silica gel 60 F254 aluminium sheets and spots were visualized under UV light at 254 nm, by potassium permanganate or ninhydrin staining. Lyophilization was done with a Labogene CoolSafe 100-9 Pro equipped with a Welch CRV Pro 2 vacuum pump (Labogene, Allerød, Denmark). Nuclear magnetic resonance (^{1}H NMR and ^{13}C NMR) spectra were recorded on a Bruker (Karlsruhe, Germany) Avance 300 (^{1}H: 300 MHz, ^{13}C: 75 MHz), 400 (^{1}H: 400 MHz, ^{13}C: 101 MHz), 500 (^{1}H: 500 MHz, ^{13}C: 126 MHz) or 600 (^{1}H: 600 MHz, ^{13}C: 151 MHz) spectrometer using perdeuterated solvents. The chemical shift δ is given in parts per million (ppm). Multiplicities were specified with the following abbreviations: s (singlet), d (doublet), t (triplet), q (quartet), and m (multiplet) as well as combinations thereof. ^{13}C NMR-Peaks were determined by DEPT

135 and DEPT 90 (distortionless enhancement by polarization transfer). NMR spectra were processed with MestReNova 11.0 (Mestrelab Research, Compostela, Spain). High-resolution mass spectrometry (HRMS) was performed on an Agilent 6540 UHD Accurate-Mass Q-TOF LC/MS system (Agilent Technologies, Santa Clara, CA) using an ESI source. A preparative HPLC system from Thermo Fisher Scientific consisting of an UltiMate 3000 HPG-3200BX PUMP and a VWD-3400RS UV/Vis-detector is used for compound purification (Thermo Fisher Scientific, Waltham, MA). A Phenomenex Gemini 5 µm NX-C18 column (110 Å, 250 x 21.2 mm, Phenomenex Ltd., Aschaffenburg, Germany) served as stationary phase. As mobile phase, 0.1 % TFA in millipore water and acetonitrile (MeCN) were used. The temperature was 25 °C, the flow rate 20 mL/min and UV detection was performed at 220 nm. Analytical purity control is performed on a Vanquish Core HPLC system from Thermo Fisher Scientific equipped with a Binary Pump C, a Split Sampler CT, Column Compartment C, a Diode Array Detector CG and an ISQ EC Single Quadrupole Mass Spectrometer (Thermo Fisher Scientific, Waltham, MA). The column was a Gemini 5 µm NX-C18 column (110 Å, 250 x 4.6 mm) or Gemini 3 µm NX-C18 column (110 Å, 150 x 3 mm) (Phenomenex Ltd., Aschaffenburg, Germany) tempered at 30 °C. As mobile phase, mixtures of MeCN and aqueous TFA (Gemini 5 µm: linear gradient: MeCN/TFA (0.05 %) (v/v) 0 min: 10:95, 25-35 min: 95:5, 36-45 min: 10:90; flow rate = 1.00 mL/min, t_0 = 2.90 min) or mixtures of MeCN and aqueous formic acid (Gemini 3 µm: linear gradient: 0.025 % formic acid in MeCN /formic acid (0.05 %) (v/v) 0 min: 10:95, 15-21 min: 95:5, 21.5-27 min: 10:90; flow rate = 0.50 mL/min, t_0 = 1.51 min) were used. Capacity factors were calculated according to $k = (t_R - t_0)/t_0$. Detection was performed at 220 nm. Furthermore, a filtration of the stock solutions with PTFE filters (25 or 15 mm, 0.2 µm, Phenomenex Ltd., Aschaffenburg, Germany) was carried out before testing. Compound purities determined by HPLC were calculated as the peak area of the analyzed compound in % relative to the total peak area (UV detection at 220 nm). The NMR spectra (Figure S11-S146) and HPLC purity (Figure S147-S222) of final compounds are displayed in the SI.

Synthesis and analytical data

*General procedure for the synthesis of **3-4***

1-2 (1 eq) were dissolved in DCM at 0 °C and triphenylphosphine (1.2 eq) was added to the solution. Subsequently, *N*-bromosuccinimide (1.2 eq) was added dropwise over 15 minutes. The reaction mixture was stirred at 0 °C for 4 hours. After completion, the crude product was purified by flash column chromatography, yielding **3-4**.

3-(4-Bromobutyl)pyridine (3)

The title compound was synthesized according to the general procedure with **1** (0.36 mL, 2.6 mmol, 1 eq), triphenylphosphine (833 mg, 3.2 mmol, 1.2 eq) and *N*-bromosuccinimide (565 mg, 3.2 mmol, 1.2 eq), yielding an orange oil (375 mg, 66 %). ^{1}H NMR (400 MHz, CD_3OD) δ 8.78 – 8.67 (m, 1H), 8.48 (d, *J* = 8.2 Hz, 2H), 8.03 – 7.94 (m, 1H), 3.53 (t, *J* = 6.4 Hz, 2H), 2.92 (t, *J* = 7.5 Hz, 2H), 2.00 – 1.85 (m, 4H). HRMS (ESI-MS): m/z [M+H$^+$] calculated for $C_9H_{13}BrN^+$: 214.0226, found 214.0226. $C_9H_{12}BrN$ (213.02).

4-(3-Bromopropyl)pyridine (4)

The title compound was synthesized according to the general procedure with **2** (0.38 mL, 2.9 mmol, 1 eq), triphenylphosphine (918 mg, 3.5 mmol, 1.2 eq) and *N*-bromosuccinimide (623 mg, 3.5 mmol, 1.2 eq), yielding an orange oil (918 mg, 54 %). ^{1}H NMR (400 MHz, CD_3OD) δ 8.70 (s, 2H), 7.89 (d, *J* = 5.8 Hz, 2H), 3.53 (t, *J* = 6.4 Hz, 2H), 3.10 (t, *J* = 7.6 Hz, 2H), 2.35 – 2.26 (m, 2H). HRMS (ESI-MS): m/z [M+H$^+$] calculated for $C_8H_{11}BrN^+$: 200.0069, found 200.0071. $C_8H_{10}BrN$ (199.00).

*General procedure for the synthesis of **21-37***

5 was dissolved in butan-2-one at room temperature, followed by addition of the corresponding bromide **3, 4,** or **6-20** (1.5-6 eq). The reaction was heated to 90 °C and stirred for 48 h. The crude product was purified by HPLC, yielding **21-37**.

1-Benzyl-3-(4-phenylbutyl)-1,3-dihydro-2*H*-benzo[d]imidazol-2-imine hydrotrifluoroacetate (21)

The title compound was synthesized according to the general procedure with **5** (70 mg, 0.31 mmol, 1 eq) and **6** (221 µL, 1.3 mmol, 4 eq), yielding a hygroscopic white solid (46 mg, 30 %): RP-HPLC: 97 %, (t_R = 14.94, *k* = 4.15). ^{1}H NMR (300 MHz, CD_3OD) δ 7.52 – 7.43 (m, 1H), 7.41 – 7.03 (m, 13H), 5.43 (s, 2H), 4.23 (t, *J* = 7.2 Hz, 2H), 2.65 (t, *J* = 7.4 Hz, 2H), 1.93 – 1.78 (m, 2H), 1.77 – 1.64 (m, 2H). ^{13}C NMR (75 MHz, CD_3OD) δ 149.69, 141.52, 133.88, 129.75, 129.67, 128.78, 128.04, 128.01, 127.99, 126.42, 125.58, 124.04, 123.90, 110.48, 110.20, 45.81, 42.78, 34.90, 27.95, 27.02. HRMS (ESI-MS): m/z [M+H$^+$] calculated for $C_{24}H_{26}N_3^+$: 356.2121, found 356.2127. $C_{24}H_{25}N_3$ x $C_2HF_3O_2$ (469.20).

1-Benzyl-3-(4-(pyridin-3-yl)butyl)-1,3-dihydro-2*H*-benzo[d]imidazol-2-imine dihydrotrifluoroacetate (22)

The title compound was synthesized according to the general procedure with **5** (30 mg, 0.13 mmol, 1 eq) and **3** (115 mg, 0.54 mmol, 4 eq), yielding a hygroscopic yellow solid (10 mg, 13 %): RP-HPLC: 97 %, (t_R = 8.82, *k* = 2.04). ^{1}H NMR (400 MHz, CD_3OD) δ 8.72 – 8.59 (m, 2H), 8.38 – 8.27 (m, 1H), 7.92 – 7.81 (m, 1H), 7.60 – 7.55 (m, 1H), 7.47 – 7.21 (m, 8H), 5.47 (s, 2H), 4.30 (t, *J* = 7.2 Hz, 2H), 2.93 (t, *J* = 7.6 Hz, 2H), 2.00 – 1.92 (m, 2H), 1.88 – 1.79 (m, 2H). ^{13}C NMR (101 MHz, CD_3OD) δ 149.78, 144.44, 142.61, 140.94, 140.89, 133.85, 129.78, 128.81, 128.11, 126.45, 126.32, 126.30, 124.13, 124.02, 110.59, 110.16, 45.88, 42.60, 31.66, 27.16, 27.08. HRMS (ESI-MS): m/z [M+H$^+$] calculated for $C_{23}H_{25}N_4^+$: 357.2074, found 357.2073. $C_{23}H_{24}N_4$ x $2C_2HF_3O_2$ (584.19).

1-Benzyl-3-(3-phenylpropyl)-1,3-dihydro-2*H*-benzo[d]imidazol-2-imine hydrotrifluoroacetate (23)

The title compound was synthesized according to the general procedure with **5** (70 mg, 0.31 mmol, 1 eq) and **7** (191 µL, 1.3 mmol, 4 eq), yielding a hygroscopic white solid (31 mg, 22 %): RP-HPLC: > 99 %, (t_R = 14.37, *k* = 3.96). ^{1}H NMR (300 MHz, CD_3OD) δ 7.44 – 7.11 (m, 14H), 5.39 (s, 2H), 4.27 (t, *J* = 9.8 Hz, 2H), 2.75 (t, *J* = 10.2 Hz, 2H), 2.26 – 2.09 (m, 2H). ^{13}C NMR (75 MHz, CD_3OD) δ 149.69, 140.32, 133.83, 129.75, 129.69, 128.77, 128.11, 128.04, 127.81, 126.42, 125.87, 124.00, 123.88, 110.46, 110.11, 45.82, 42.62, 32.16, 28.83. HRMS (ESI-MS): m/z [M+H$^+$] calculated for $C_{23}H_{24}N_3^+$: 342.1965, found 342.1969. $C_{23}H_{23}N_3$ x $C_2HF_3O_2$ (455.18).

1-Benzyl-3-(3-(pyridin-4-yl)propyl)-1,3-dihydro-2*H*-benzo[d]imidazol-2-imine dihydrotrifluoroacetate (24)

The title compound was synthesized according to the general procedure with **5** (30 mg, 0.13 mmol, 1 eq) and **4** (107 mg, 0.54 mmol, 4 eq), yielding a hygroscopic yellow solid (6 mg, 8 %): RP-HPLC: > 99 %, (t_R = 8.39, *k* = 1.89). ^{1}H NMR (400 MHz, CD_3OD) δ 8.57 – 8.51 (m, 2H), 7.74 – 7.67 (m, 2H), 7.52 – 7.46 (m, 1H), 7.39 – 7.13 (m, 8H), 5.35 (s, 2H), 4.27 (t, *J* = 7.5 Hz, 2H), 2.96 (t, *J* = 8.2 Hz, 2H), 2.24 – 2.13 (m, 2H).. HRMS (ESI-MS): m/z [M+H$^+$] calculated for $C_{22}H_{23}N_4^+$: 343.1917, found 343.1915, m/z [M+2H^{2+}] calculated for $C_{22}H_{24}N_4^{2+}$: 172.0995, found 172.0995. $C_{22}H_{22}N_4$ x $2C_2HF_3O_2$ (570.17).

1-Benzyl-3-(3-(piperazin-1-yl)propyl)-1,3-dihydro-2*H*-benzo[d]imidazol-2-imine trihydrotrifluoroacetate (25)

The title compound was synthesized according to the general procedure with **5** (50 mg, 0.22 mmol, 1 eq) and **8** (206 mg, 0.67 mmol, 3 eq). The crude product was subsequently dissolved in a 5:1 mixture of dichloromethane and trifluoroacetic acid and was stirred for 2 hours to remove the Boc protecting group, yielding a beige solid (45 mg, 29 %): RP-HPLC: > 99 %, (t_R = 7.22, *k* = 1.49). ^{1}H NMR (400 MHz, CD_3OD) δ 7.66 – 7.28 (m, 9H), 5.49 (s, 2H), 4.37 (t, *J* = 7.2 Hz, 2H), 3.46 (t, *J* = 5.3 Hz, 4H), 3.08 (t, *J* = 7.5 Hz, 2H), 2.33 – 2.21 (m, 2H). ^{13}C NMR (101 MHz, CD_3OD) δ 149.95, 133.82, 129.80, 129.79, 128.81, 128.10, 126.56, 124.17, 124.08, 110.61, 110.14, 53.47, 48.69, 46.00, 41.63, 40.20, 22.95. HRMS (ESI-MS): m/z [M+H$^+$] calculated for $C_{21}H_{28}N_5^+$: 350.2339, found 350.2343, m/z [M+2H^{2+}] calculated for $C_{21}H_{29}N_5^{2+}$: 175.6206, found 175.6207. $C_{21}H_{27}N_5$ x $C_2HF_3O_2$ (691.21).

1-Benzyl-3-phenethyl-1,3-dihydro-2*H*-benzo[d]imidazol-2-imine hydrotrifluoroacetate (26)

The title compound was synthesized according to the general procedure with **5** (25 mg, 0.11 mmol, 1 eq) and **9** (30 µL, 0.22 mmol, 2 eq), yielding a hygroscopic white solid (3 mg, 6 %): RP-HPLC: 98 %, (t_R = 13.40, *k* = 3.62). ^{1}H NMR (300 MHz, CD_3OD) δ 7.47 – 7.22 (m, 7H), 7.22 – 7.03 (m, 7H), 5.36 (s, 2H), 4.50 (t, *J* = 6.7 Hz, 2H), 3.17 (t, *J* = 6.7 Hz, 2H). HRMS (ESI-MS): m/z [M+H$^+$] calculated for $C_{22}H_{22}N_3^+$: 328.1808, found 328.1812. $C_{22}H_{21}N_3$ x $C_2HF_3O_2$ (441.16).

1,3-Dibenzyl-1,3-dihydro-2*H*-benzo[d]imidazol-2-imine hydrotrifluoroacetate (27)

The title compound was synthesized according to the general procedure with **5** (20 mg, 0.09 mmol, 1 eq) and **10** (64 µL, 0.54 mmol, 6 eq), yielding a hygroscopic white solid (27 mg, 70 %): RP-HPLC: 98 %, (t_R = 12.89, *k* = 3.44). ^{1}H NMR (400 MHz, CD_3OD) δ 7.32 – 7.13 (m, 14H), 5.40 (s, 4H). ^{13}C NMR (101 MHz, CD_3OD) δ 150.30, 133.85, 129.86, 128.85, 128.12, 126.47, 124.17, 110.64, 46.03. HRMS (ESI-MS): m/z [M+H$^+$] calculated for $C_{21}H_{20}N_3^+$: 314.1652, found 314.1655. $C_{21}H_{19}N_3$ x $C_2HF_3O_2$ (427.15).

1-Benzyl-3-(2-fluorobenzyl)-1,3-dihydro-2*H*-benzo[d]imidazol-2-imine hydrotrifluoroacetate (28)

The title compound was synthesized according to the general procedure with **5** (30 mg, 0.13 mmol, 1 eq) and **11** (24.3 µL, 0.20 mmol, 1.5 eq), yielding a hygroscopic beige solid (33 mg, 54 %): RP-HPLC: > 99 %, (t_R = 13.12, *k* = 3.52). ^{1}H NMR (400 MHz, CD_3OD) δ 7.47 – 7.26 (m, 11H), 7.26 – 7.18 (m, 2H), 5.59 (s, 2H), 5.53 (s, 2H). ^{13}C NMR (101 MHz, CD_3OD) δ 160.75 (d, *J* = 246.2 Hz), 150.32, 133.77, 130.52 (d, *J* = 8.2 Hz), 129.71 (d, *J* = 24.3 Hz), 128.82, 128.66 (d, *J* = 3.5 Hz), 128.10, 126.40, 124.61 (d, *J* = 3.9 Hz), 124.19, 124.12, 120.79 (d, *J* = 14.3 Hz), 115.70, 115.49, 110.62, 110.48, 46.01, 41.33 (d, *J* = 4.3 Hz). HRMS (ESI-MS): m/z [M+H$^+$] calculated for $C_{21}H_{19}FN_3^+$: 332.1558, found 332.1563. $C_{21}H_{18}FN_3$ x $C_4H_2F_6O_4$ (445.14).

1-Benzyl-3-(3-fluorobenzyl)-1,3-dihydro-2*H*-benzo[d]imidazol-2-imine hydrotrifluoroacetate (29)

The title compound was synthesized according to the general procedure with **5** (50 mg, 0.22 mmol, 1 eq) and **12** (41 µL, 0.34 mmol, 1.5 eq), yielding a hygroscopic beige solid (71 mg, 71 %): RP-HPLC: > 99 %, (t_R = 13.25, k = 3.57). ^{1}H NMR (400 MHz, CD_3OD) δ 7.48 – 7.27 (m, 10H), 7.15 – 7.03 (m, 3H), 5.56 (s, 2H), 5.54 (s, 2H). ^{13}C NMR (101 MHz, CD_3OD) δ 163.15 (d, J = 246.2 Hz), 150.33, 136.61 (d, J = 7.4 Hz), 133.80, 130.78 (d, J = 8.7 Hz), 129.87, 129.75, 128.84, 128.12, 126.48, 124.28, 124.25, 122.17 (d, J = 3.0 Hz), 114.87 (d, J = 21.2 Hz), 113.41 (d, J = 23.0 Hz), 110.72, 110.48, 46.10, 45.48 (d, J = 2.0 Hz). HRMS (ESI-MS): m/z [M+H$^+$] calculated for $C_{21}H_{19}FN_3^+$: 332.1558, found 332.1561. $C_{21}H_{18}FN_3$ x $C_2HF_3O_2$ (445.14).

1-Benzyl-3-(4-fluorobenzyl)-1,3-dihydro-2*H*-benzo[d]imidazol-2-imine hydrotrifluoroacetate (30)

The title compound was synthesized according to the general procedure with **5** (50 mg, 0.22 mmol, 1 eq) and **13** (42 µL, 0.34 mmol, 1.5 eq), yielding a hygroscopic beige solid (70 mg, 70 %): RP-HPLC: > 99 %, (t_R = 13.31, k = 3.59). ^{1}H NMR (400 MHz, CD_3OD) δ 7.54 – 7.24 (m, 11H), 7.19 – 7.08 (m, 2H), 5.53 (s, 2H), 5.52 (s, 2H). ^{13}C NMR (101 MHz, CD_3OD) δ 162.65 (d, J = 246.2 Hz), 150.22, 133.79, 129.89 (d, J = 4.3 Hz), 129.87, 129.73, 128.84, 128.67 (d, J = 8.2 Hz), 128.12, 126.48, 126.48, 124.21, 115.59 (d, J = 22.1 Hz), 110.67, 110.57, 46.07, 45.41. HRMS (ESI-MS): m/z [M+H$^+$] calculated for $C_{21}H_{19}FN_3^+$: 332.1558, found 332.1561. $C_{21}H_{18}FN_3$ x $C_2HF_3O_2$ (445.14).

1-Benzyl-3-(1-phenylethyl)-1,3-dihydro-2*H*-benzo[d]imidazol-2-imine hydrotrifluoroacetate (31)

The title compound was synthesized according to the general procedure with **5** (25 mg, 0.11 mmol, 1 eq) and **14** (31 µL, 0.22 mmol, 2 eq), yielding a hygroscopic white solid (4 mg, 8 %): RP-HPLC: 97 %, (t_R = 13.53, k = 3.67). ^{1}H NMR (300 MHz, CD_3OD) δ 7.56 – 6.89 (m, 14H), 6.01 (q, J = 7.0 Hz, 1H), 5.50 (s, 2H), 2.05 (d, J = 7.1 Hz, 2H). HRMS (ESI-MS): m/z [M+H$^+$] calculated for $C_{22}H_{22}N_3^+$: 328.1808, found 328.1811. $C_{22}H_{21}N_3$ x $C_2HF_3O_2$ (441.16).

1-Benzyl-3-(cyclohexylmethyl)-1,3-dihydro-2*H*-benzo[d]imidazol-2-imine hydrotrifluoroacetate (32)

The title compound was synthesized according to the general procedure with **5** (50 mg, 0.22 mmol, 1 eq) and **15** (141 µL, 1.0 mmol, 4.5 eq), yielding a hygroscopic white solid (5 mg, 5 %): RP-HPLC: 97 %, (t_R = 14.59, k = 4.03). ^{1}H NMR (400 MHz, CD_3OD) δ 7.49 – 7.44 (m, 1H), 7.33 – 7.10 (m, 8H), 5.36 (s, 2H), 3.97 (d, J = 7.7 Hz, 2H), 1.94 – 1.82 (m, 1H), 1.74 – 1.50 (m, 5H), 1.23 – 1.00 (m, 5H). ^{13}C NMR (101 MHz, CD_3OD) δ 150.04, 133.88, 130.30, 129.60, 128.81, 128.08, 126.34, 124.05, 123.93, 110.70, 110.47, 48.58, 45.83, 36.74, 29.91, 25.75, 25.30. HRMS (ESI-MS): m/z [M+H$^+$] calculated for $C_{21}H_{26}N_3^+$: 320.2121, found 320.2125. $C_{21}H_{25}N_3$ x $C_2HF_3O_2$ (433.20).

1-Benzyl-3-(cyclopropylmethyl)-1,3-dihydro-2*H*-benzo[d]imidazol-2-imine hydrotrifluoroacetate (33)

The title compound was synthesized according to the general procedure with **5** (70 mg, 0.31 mmol, 1 eq) and **16** (46 µL, 0.5 mmol, 1.5 eq), yielding a hygroscopic white solid (5 mg, 4 %): RP-HPLC: 99 %, (t_R = 12.11, k = 3.18). ^{1}H NMR (400 MHz, CD_3OD) δ 7.64 – 7.56 (m, 1H), 7.45 – 7.25 (m, 8H), 5.49 (s, 2H), 4.20 (d, J = 7.0 Hz, 2H), 1.47 – 1.32 (m, 1H), 0.72 – 0.64 (m, 2H), 0.57 – 0.52 (m, 2H). ^{13}C NMR (101 MHz, CD_3OD) δ 148.45, 132.53, 128.60, 128.41, 127.43, 126.70, 125.08, 125.04, 122.72, 122.54, 109.13, 45.59, 44.50, 7.97, 1.42. HRMS (ESI-MS): m/z [M+H$^+$] calculated for $C_{18}H_{20}N_3^+$: 278.1652, found 278.1655. $C_{18}H_{19}N_3$ x $C_2HF_3O_2$ (391.15).

1-Benzyl-3-butyl-1,3-dihydro-2*H*-benzo[d]imidazol-2-imine hydrotrifluoroacetate (34)

The title compound was synthesized according to the general procedure with **5** (40 mg, 0.18 mmol, 1 eq) and **17** (136 µL, 1.1 mmol, 6 eq), yielding a hygroscopic white solid (16 mg, 23 %): RP-HPLC: 97 %, (t_R = 12.89, k = 3.44). ^{1}H NMR (400 MHz, CD_3OD) δ 7.48 – 7.42 (m, 1H), 7.32 – 7.13 (m, 8H), 5.35 (s, 2H), 4.13 (t, J = 7.4 Hz, 2H), 1.78 – 1.68 (m, 2H), 1.41 – 1.30 (m, 2H), 0.90 (t, J = 7.4 Hz, 3H). ^{13}C NMR (101 MHz, CD_3OD) δ 149.74, 133.88, 129.85, 129.74, 128.81, 128.08, 126.39, 124.10, 123.93, 110.51, 110.23, 45.84, 42.84, 29.68, 19.44, 12.66. HRMS (ESI-MS): m/z [M+H$^+$] calculated for $C_{18}H_{22}N_3^+$: 280.1808, found 280.1811. $C_{18}H_{21}N_3$ x $C_2HF_3O_2$ (393.17).

1-Benzyl-3-isobutyl-1,3-dihydro-2*H*-benzo[d]imidazol-2-imine hydrotrifluoroacetate (35)

The title compound was synthesized according to the general procedure with **5** (40 mg, 0.18 mmol, 1 eq) and **18** (116 µL, 1.1 mmol, 6 eq), yielding a hygroscopic white solid (17 mg, 25 %): RP-HPLC: 97 %, (t_R = 12.66, k = 3.37). ^{1}H NMR (400 MHz, CD_3OD) δ 7.49 – 7.42 (m, 1H), 7.31 – 7.18 (m, 6H), 7.17 – 7.09 (m, 2H), 5.37 (s, 2H), 3.96 (d, J = 7.9 Hz, 2H), 2.27 – 2.15 (m, 1H), 0.92 (d, J = 6.7 Hz, 6H). ^{13}C NMR (101 MHz, CD_3OD) δ 150.02, 133.88, 130.18, 129.63, 128.81, 128.08, 126.36, 124.06, 123.95, 110.67, 110.49, 49.60, 45.83, 27.51, 18.44. HRMS (ESI-MS): m/z [M+H$^+$] calculated for $C_{18}H_{22}N_3^+$: 280.1808, found 280.1809. $C_{18}H_{21}N_3$ x $C_2HF_3O_2$ (393.17).

1-Benzyl-3-ethyl-1,3-dihydro-2*H*-benzo[d]imidazol-2-imine hydrotrifluoroacetate (36)

The title compound was synthesized according to the general procedure with **5** (40 mg, 0.18 mmol, 1 eq) and **19** (20 µL, 0.27 mmol, 1.5 eq), yielding a hygroscopic white solid (24 mg, 36 %): RP-HPLC: > 99 %, (t_R = 11.23, k = 2.87). ^1H NMR (400 MHz, CD_3OD) δ 7.48 – 7.43 (m, 1H), 7.33 – 7.11 (m, 8H), 5.35 (s, 2H), 4.19 (q, J = 7.2 Hz, 2H), 1.33 (t, J = 7.2 Hz, 3H). ^{13}C NMR (101 MHz, CD_3OD) δ 149.52, 133.91, 129.85, 129.42, 128.80, 128.06, 126.48, 124.10, 123.91, 110.53, 109.96, 45.87, 38.02, 11.92. HRMS (ESI-MS): m/z [M+H$^+$] calculated for $C_{16}H_{18}N_3^+$: 252.1495, found 252.1498. $C_{16}H_{17}N_3$ x $C_2HF_3O_2$ (365.14).

1-Benzyl-3-methyl-1,3-dihydro-2*H*-benzo[d]imidazol-2-imine hydrotrifluoroacetate (37)

The title compound was synthesized according to the general procedure with **5** (40 mg, 0.18 mmol, 1 eq) and **20** (17 µL, 0.27 mmol, 1.5 eq), yielding a hygroscopic white solid (15 mg, 24 %): RP-HPLC: 97 %, (t_R = 10.59, k = 2.65). ^1H NMR (400 MHz, CD_3OD) δ 7.45 – 7.40 (m, 1H), 7.31 – 7.16 (m, 8H), 5.35 (s, 2H), 3.66 (s, 3H). ^{13}C NMR (101 MHz, CD_3OD) δ 150.41, 133.90, 130.52, 129.69, 128.77, 128.05, 126.48, 124.02, 123.85, 110.38, 109.91, 45.88, 28.51. HRMS (ESI-MS): m/z [M+H$^+$] calculated for $C_{15}H_{16}N_3^+$: 238.1339, found 238.1343. $C_{15}H_{15}N_3$ x $C_2HF_3O_2$ (351.12).

*General procedure for the synthesis of **43-46***

43-46 were synthesized by dissolving **38** (1.0 g, 7.5 mmol, 1 eq) in 2.7 ml of a 20 M NaOH solution (7.2 eq) at 40 °C. After stirring the mixture for 15 min until the starting material dissolved, yielding a viscous, red solution, 40 mL of acetone were added, resulting in the deprotonated product precipitating into a beige solid. Subsequently, the corresponding bromide **39-42** (1 eq) was added to the suspension. The approach was then heated to reflux at 60 °C and stirred for 2 h until the product was dissolved. Following evaporation of acetone under reduced pressure, the residue was dispersed in 40 mL of water leading to a slight yellow dispersion and a clumpy, brown solid, which remained at the bottom of the flask. The desired, yellow produ ct was decanted and extracted using ethyl acetate (3 x 15 mL). The combined organic layers were washed with 1 M sodium hydroxide solution (3 x 15 mL) to remove residual **38**. Following solvent removal by rotary evaporation, **44-46** were used for the synthesis of **52-75** without further purification.

1-(4-Methylbenzyl)-1,3-dihydro-2*H*-benzo[d]imidazol-2-imine hydrotrifluoroacetate (43)

The title compound was synthesized according to the general procedure with **39** (1.39 g, 7.5 mmol, 1 eq) and additionally purified by HPLC, yielding a beige solid (1.11 g, 46 %). ^1H NMR (400 MHz, CD_3OD) δ 7.43 – 7.36 (m, 1H), 7.32 – 7.11 (m, 7H), 5.33 (s, 2H), 2.27 (s, 3H). ^{13}C NMR (101 MHz, CD_3OD) δ 150.64, 138.07, 130.94, 130.35, 129.34, 128.95, 126.62, 123.96, 123.21, 111.28, 110.30, 45.33, 19.73. HRMS (ESI-MS): m/z [M+H$^+$] calculated for $C_{15}H_{16}N_3^+$: 238.1339, found 238.1341. $C_{15}H_{15}N_3$ x $C_2HF_3O_2$ (351.12).

1-(2-Fluorobenzyl)-1,3-dihydro-2*H*-benzo[d]imidazol-2-imine (44)

The title compound was synthesized according to the general procedure with **40** (904 µL, 7.5 mmol, 1 eq), yielding a yellow solid (739 mg, 61 %). ^1H NMR (400 MHz, CD_3OD) δ 7.37 – 7.24 (m, 2H), 7.24 – 6.88 (m, 6H), 5.35 (s, 2H). HRMS (ESI-MS): m/z [M+H$^+$] calculated for $C_{14}H_{13}FN_3^+$: 242.1088, found 242.1088. $C_{14}H_{12}FN_3$ (241.10)

1-(3-Fluorobenzyl)-1,3-dihydro-2*H*-benzo[d]imidazol-2-imine (45)

The title compound was synthesized according to the general procedure with **41** (922 µL, 7.5 mmol, 1 eq), yielding a brown solid (1.49 g, 62 %). ^1H NMR (400 MHz, CD_3OD) δ 7.32 – 7.22 (m, 2H), 7.09 – 6.86 (m, 6H), 5.22 (s, 2H). ^{13}C NMR (101 MHz, CD_3OD) δ 163.05 (d, J = 245.4 Hz), 154.92, 141.32, 139.17 (d, J = 6.9 Hz), 133.75, 130.21 (d, J = 8.2 Hz), 122.13 (d, J = 3.0 Hz), 121.37, 119.47, 114.68, 113.97 (d, J = 21.2 Hz), 113.20 (d, J = 22.5 Hz), 107.88, 44.49 (d, J = 1.8 Hz). HRMS (ESI-MS): m/z [M+H$^+$] calculated for $C_{14}H_{13}FN_3^+$: 242.1088, found 242.1091. $C_{14}H_{12}FN_3$ (241.10).

1-(4-Fluorobenzyl)-1,3-dihydro-2*H*-benzo[d]imidazol-2-imine (46)

The title compound was synthesized according to the general procedure with **42** (936 µL, 7.5 mmol, 1 eq), yielding a yellow solid (1.53 g, 63 %). ^1H NMR (400 MHz, CD_3OD) δ 7.48 – 7.02 (m, 8H), 5.39 (s, 2H). ^{13}C NMR (101 MHz, CD_3OD) δ 162.60 (d, J = 245.8 Hz), 150.66, 130.26, 130.06 (d, J = 3.5 Hz), 128.98, 128.71 (d, J = 8.2 Hz), 124.08, 123.33, 115.52 (d, J = 21.7 Hz), 111.36, 110.20, 44.83. HRMS (ESI-MS): m/z [M+H$^+$] calculated for $C_{14}H_{13}FN_3^+$: 242.1088, found 242.1092. $C_{14}H_{12}FN_3$ (241.10).

*General procedure for the synthesis of **52-75***

5 or **43-46** were dissolved in ethanol at room temperature, followed by addition of the corresponding epoxide **47-51** (1.3-1.5 eq). The approach was heated to reflux at 100 °C and stirred for 48 h. The crude product was purified by HPLC, yielding **52-75**.

1-(3-Benzyl-2-imino-2,3-dihydro-1*H*-benzo[d]imidazol-1-yl)-3-(4-chlorophenoxy)propan-2-ol hydrotrifluoroacetate (52)

The title compound was synthesized according to the general procedure with **5** (20 mg, 0.09 mmol, 1 eq) and **47** (22 mg, 0.12 mmol, 1.3 eq), yielding a hygroscopic white solid (22 mg, 48 %): RP-HPLC: > 99 %, (t_R = 14.44, k = 3.98). ^{1}H NMR (400 MHz, CD_3OD) δ 7.61 – 7.57 (m, 1H), 7.42 – 7.26 (m, 10H), 7.00 – 6.92 (m, 2H), 5.49 (s, 2H), 4.53 – 4.42 (m, 3H), 4.15 – 4.05 (m, 2H). ^{13}C NMR (101 MHz, CD_3OD) δ 157.23, 150.98, 133.82, 130.43, 129.74, 129.05, 128.79, 128.06, 126.46, 125.82, 124.02, 123.96, 115.70, 110.62, 110.44, 69.27, 67.36, 46.19, 45.93. HRMS (ESI-MS): m/z [M+H$^+$] calculated for $C_{23}H_{23}ClN_3O_2^+$: 408.148, found 408.1473. $C_{23}H_{22}ClN_3O_2$ x $C_2HF_3O_2$ (521.13).

1-(4-Chlorophenoxy)-3-(2-imino-3-(4-methylbenzyl)-2,3-dihydro-1*H*-benzo[d]imidazol-1-yl)propan-2-ol hydrotrifluoroacetate (53)

The title compound was synthesized according to the general procedure with **43** (15 mg, 0.04 mmol, 1 eq) and **47** (10 mg, 0.06 mmol, 1.3 eq), yielding a hygroscopic white solid (9 mg, 27 %): RP-HPLC: > 99 %, (t_R = 15.08, k = 4.20). ^{1}H NMR (400 MHz, CD_3OD) δ 7.48 – 7.43 (m, 1H), 7.29 – 7.04 (m, 9H), 6.87 – 6.80 (m, 2H), 5.31 (s, 2H), 4.42 – 4.29 (m, 3H), 4.02 – 3.92 (m, 2H), 2.21 (s, 3H). ^{13}C NMR (101 MHz, CD_3OD) δ 157.22, 152.83, 138.15, 130.75, 130.41, 129.73, 129.35, 129.04, 126.51, 125.82, 123.98, 123.93, 115.70, 110.58, 110.50, 69.26, 67.35, 46.17, 45.77, 19.68. HRMS (ESI-MS): m/z [M+H$^+$] calculated for $C_{24}H_{25}ClN_3O_2^+$: 422.163, found 422.1631. $C_{24}H_{24}ClN_3O_2$ x $C_2HF_3O_2$ (535.15).

1-(4-Chlorophenoxy)-3-(3-(2-fluorobenzyl)-2-imino-2,3-dihydro-1*H*-benzo[d]imidazol-1-yl)propan-2-ol hydrotrifluoroacetate (54)

The title compound was synthesized according to the general procedure with **44** (19 mg, 0.08 mmol, 1 eq) and **47** (19 mg, 0.10 mmol, 1.3 eq), yielding a hygroscopic white solid (27 mg, 65 %): RP-HPLC: > 99 %, (t_R = 14.37, k = 3.96). ^{1}H NMR (400 MHz, CD_3OD) δ 7.61 – 7.56 (m, 1H), 7.45 – 7.15 (m, 9H), 7.01 – 6.93 (m, 2H), 5.55 (s, 2H), 4.53 – 4.41 (m, 3H), 4.16 – 4.03 (m, 2H). ^{13}C NMR (101 MHz, CD_3OD) δ 161.26 (d, J = 365.2 Hz), 157.22, 151.04, 130.39 (d, J = 6.4 Hz), 129.69 (d, J = 31.5 Hz), 129.04, 128.41 (d, J = 3.5 Hz), 125.82, 124.55 (d, J = 3.8 Hz), 124.08, 123.97, 120.82 (d, J = 14.5 Hz), 115.70, 115.61, 115.40, 110.65, 110.27, 69.23, 67.37, 46.20, 40.99 (d, J = 4.8 Hz). HRMS (ESI-MS): m/z [M+H$^+$] calculated for $C_{23}H_{22}ClFN_3O_2^+$: 426.1379, found 426.1386. $C_{23}H_{21}ClFN_3O_2$ x $C_2HF_3O_2$ (539.12).

1-(4-Chlorophenoxy)-3-(3-(3-fluorobenzyl)-2-imino-2,3-dihydro-1*H*-benzo[d]imidazol-1-yl)propan-2-ol hydrotrifluoroacetate (55)

The title compound was synthesized according to the general procedure with **45** (30 mg, 0.12 mmol, 1 eq) and **47** (35 mg, 0.19 mmol, 1.5 eq), yielding a hygroscopic white solid (33 mg, 49 %): RP-HPLC: > 99 %, (t_R = 14.45, k = 3.98). ^{1}H NMR (400 MHz, CD_3OD) δ 7.63 – 7.57 (m, 1H), 7.44 – 7.26 (m, 6H), 7.14 – 7.04 (m, 3H), 6.99 – 6.93 (m, 2H), 5.51 (s, 2H), 4.54 – 4.43 (m, 3H), 4.16 – 4.06 (m, 2H). ^{13}C NMR (101 MHz, CD_3OD) δ 163.16 (d, J = 246.2 Hz), 157.22, 151.02, 136.59 (d, J = 7.4 Hz), 130.73 (d, J = 8.2 Hz), 130.45, 129.64, 129.05, 125.81, 124.15, 124.06, 122.19 (d, J = 3.0 Hz), 115.71, 114.84 (d, J = 21.2 Hz), 113.41 (d, J = 23.0 Hz), 110.71, 110.28, 69.27, 67.35, 46.24, 45.39 (d, J = 1.9 Hz). HRMS (ESI-MS): m/z [M+H$^+$] calculated for $C_{23}H_{22}ClFN_3O_2^+$: 426.1379, found 426.1382. $C_{23}H_{21}ClFN_3O_2$ x $C_2HF_3O_2$ (539.12).

1-(4-Chlorophenoxy)-3-(3-(4-fluorobenzyl)-2-imino-2,3-dihydro-1*H*-benzo[d]imidazol-1-yl)propan-2-ol hydrotrifluoroacetate (56)

The title compound was synthesized according to the general procedure with **46** (50 mg, 0.21 mmol, 1 eq) and **47** (57 mg, 0.31 mmol, 1.5 eq), yielding a hygroscopic white solid (45 mg, 40 %): RP-HPLC: > 99 %, (t_R = 14.47, k = 3.99). ^{1}H NMR (400 MHz, CD_3OD) δ 7.50 – 7.43 (m, 1H), 7.31 – 7.13 (m, 7H), 7.03 – 6.96 (m, 2H), 6.87 – 6.80 (m, 2H), 5.34 (s, 2H), 4.41 – 4.28 (m, 3H), 4.03 – 3.92 (m, 2H). ^{13}C NMR (101 MHz, CD_3OD) δ 162.62 (d, J = 246.2 Hz), 157.20, 150.91, 130.44, 129.86 (d, J = 3.5 Hz), 129.61, 129.03, 128.64 (d, J = 8.2 Hz), 125.79, 124.06, 124.00, 115.70, 115.52 (d, J = 21.7 Hz), 110.66, 110.35, 69.25, 67.34, 46.20, 45.28. HRMS (ESI-MS): m/z [M+H$^+$] calculated for $C_{23}H_{22}ClFN_3O_2^+$: 426.1379, found 426.1382. $C_{23}H_{21}ClFN_3O_2$ x $C_2HF_3O_2$ (539.12).

1-(3-Benzyl-2-imino-2,3-dihydro-1*H*-benzo[d]imidazol-1-yl)-3-(4-fluorophenoxy)propan-2-ol hydrotrifluoroacetate (57)

The title compound was synthesized according to the general procedure with **5** (20 mg, 0.09 mmol, 1 eq) and **48** (17 µL, 0.12 mmol, 1.3 eq), yielding a hygroscopic white solid (13 mg, 29 %): RP-HPLC: 98 %, (t_R = 13.68, k = 3.71). ^{1}H NMR (400 MHz, CD_3OD) δ 7.62 – 7.56 (m, 1H), 7.42 – 7.27 (m, 8H), 7.07 – 6.93 (m, 4H), 5.49 (s, 2H), 4.54 – 4.42 (m, 3H), 4.13 – 4.04 (m, 2H). ^{13}C NMR (101 MHz, CD_3OD) δ 157.59 (d, J = 237.6 Hz), 154.72 (d, J = 2.2 Hz), 150.99, 133.82, 130.44, 129.74, 128.78, 128.05,

126.45, 124.01, 123.96, 115.51 (d, *J* = 2.6 Hz), 115.35 (d, *J* = 12.6 Hz), 110.63, 110.43, 69.60, 67.43, 46.23, 45.93. HRMS (ESI-MS): m/z [M+H$^+$] calculated for $C_{23}H_{23}FN_3O_2^+$: 392.1769, found 392.1773. $C_{23}H_{22}FN_3O_2$ x $C_2HF_3O_2$ (505.16).

1-(4-Fluorophenoxy)-3-(2-imino-3-(4-methylbenzyl)-2,3-dihydro-1*H*-benzo[d]imidazol-1-yl)propan-2-ol hydrotrifluoroacetate (58)

The title compound was synthesized according to the general procedure with **43** (15 mg, 0.04 mmol, 1 eq) and **48** (8 mg, 0.06 mmol, 1.3 eq), yielding a hygroscopic white solid (20 mg, 60 %): RP-HPLC: > 99 %, (t_R = 14.28, *k* = 3.92). ^{1}H NMR (400 MHz, CD_3OD) δ 7.49 – 7.44 (m, 1H), 7.29 – 7.17 (m, 3H), 7.14 – 7.00 (m, 4H), 6.97 – 6.78 (m, 4H), 5.31 (s, 2H), 4.41 – 4.28 (m, 3H), 4.01 – 3.91 (m, 2H), 2.21 (s, 3H). 13C NMR (101 MHz, CD_3OD) δ 158.21 (d, J = 301.1 Hz), 154.75 (d, J = 1.0 Hz), 150.93, 138.15, 130.76, 130.42, 129.35, 128.88, 126.51, 123.98, 123.93, 115.51 (d, J = 3.5 Hz), 115.35 (d, J = 11.7 Hz), 110.58, 110. 50, 69.59, 67.42, 46.20, 45.78, 19.67. HRMS (ESI-MS): m/z [M+H+] calculated for $C_{24}H_{25}FN_3O_2$+: 406.1925, found 406.1929. $C_{24}H_{24}FN_3O_2$x C2HF3O2 (519.18).

1-(3-(2-Fluorobenzyl)-2-imino-2,3-dihydro-1*H*-benzo[d]imidazol-1-yl)-3-(4-fluorophenoxy)propan-2-ol hydrotrifluoroacetate (59)

The title compound was synthesized according to the general procedure with **44** (13 mg, 0.05 mmol, 1 eq) and **48** (10 µL, 0.07 mmol, 1.3 eq), yielding a hygroscopic white solid (16 mg, 57 %): RP-HPLC: > 99 %, (t_R = 13.63, *k* = 3.70). ^{1}H NMR (400 MHz, CD_3OD) δ 7.49 – 7.45 (m, 1H), 7.33 – 7.17 (m, 4H), 7.14 – 7.03 (m, 3H), 6.96 – 6.82 (m, 4H), 5.43 (s, 2H), 4.42 – 4.29 (m, 3H), 4.02 – 3.91 (m, 2H). HRMS (ESI-MS): m/z [M+H$^+$] calculated for $C_{23}H_{22}F_2N_3O_2^+$: 410.1675, found 410.1679. $C_{23}H_{21}F_2N_3O_2$ x $C_2HF_3O_2$ (523.15).

1-(3-(3-Fluorobenzyl)-2-imino-2,3-dihydro-1*H*-benzo[d]imidazol-1-yl)-3-(4-fluorophenoxy)propan-2-ol hydrotrifluoroacetate (60)

The title compound was synthesized according to the general procedure with **45** (30 mg, 0.12 mmol, 1 eq) and **48** (28 µL, 0.19 mmol, 1.5 eq), yielding a hygroscopic white solid (27 mg, 42 %): RP-HPLC: 95 %, (t_R = 13.77, *k* = 3.75). ^{1}H NMR (400 MHz, CD_3OD) δ 7.63 – 7.57 (m, 1H), 7.43 – 7.31 (m, 4H), 7.13 – 6.94 (m, 7H), 5.51 (s, 2H), 4.56 – 4.41 (m, 3H), 4.15 – 4.03 (m, 2H). ^{13}C NMR (101 MHz, CD_3OD) δ 163.16 (d, *J* = 246.2 Hz), 157.59 (d, *J* = 237.9 Hz), 154.71 (d, *J* = 2.4 Hz), 151.03, 136.60 (d, *J* = 6.9 Hz), 130.73 (d, *J* = 8.2 Hz), 130.47, 129.65, 124.14, 124.05, 122.19 (d, *J* = 3.0 Hz), 115.52 (d, *J* = 2.2 Hz), 115.36 (d, *J* = 13.0 Hz), 114.83 (d, *J* = 21.2 Hz), 113.41 (d, *J* = 22.5 Hz), 110.72, 110.28, 69.59, 67.42, 46.28, 45.40 (d, *J* = 1.8 Hz). HRMS (ESI-MS): m/z [M+H$^+$] calculated for $C_{23}H_{22}F_2N_3O_2^+$: 410.1675, found 410.1679. $C_{23}H_{21}F_2N_3O_2$ x $C_2HF_3O_2$ (523.15).

1-(3-(4-Fluorobenzyl)-2-imino-2,3-dihydro-1*H*-benzo[d]imidazol-1-yl)-3-(4-fluorophenoxy)propan-2-ol hydrotrifluoroacetate (61)

The title compound was synthesized according to the general procedure with **46** (30 mg, 0.12 mmol, 1 eq) and **48** (28 µL, 0.19 mmol, 1.5 eq), yielding a hygroscopic white solid (18 mg, 27 %): RP-HPLC: 97 %, (t_R = 13.76, *k* = 3.74). ^{1}H NMR (400 MHz, CD_3OD) δ 7.62 – 7.57 (m, 1H), 7.43 – 7.31 (m, 5H), 7.15 – 6.94 (m, 6H), 5.47 (s, 2H), 4.53 – 4.41 (m, 3H), 4.14 – 4.03 (m, 2H). ^{13}C NMR (101 MHz, CD_3OD) δ 162.65 (d, *J* = 245.4 Hz), 157.59 (d, *J* = 237.3 Hz), 154.71 (d, *J* = 2.2 Hz), 150.94, 130.46, 129.87 (d, *J* = 3.0 Hz), 129.62, 128.65 (d, *J* = 8.2 Hz), 124.08, 124.02, 115.54 (d, *J* = 22.0 Hz), 115.52 (d, *J* = 2.6 Hz), 115.36 (d, *J* = 12.6 Hz), 110.68, 110.37, 69.58, 67.42, 46.24, 45.29. HRMS (ESI-MS): m/z [M+H$^+$] calculated for $C_{23}H_{22}F_2N_3O_2^+$: 410.1675, found 410.168. $C_{23}H_{21}F_2N_3O_2$ x $C_2HF_3O_2$ (523.15).

1-(3-Benzyl-2-imino-2,3-dihydro-1*H*-benzo[d]imidazol-1-yl)-3-(p-tolyloxy)propan-2-ol hydrotrifluoroacetate (62)

The title compound was synthesized according to the general procedure with **5** (20 mg, 0.09 mmol, 1 eq) and **49** (19 µL, 0.12 mmol, 1.3 eq), yielding a hygroscopic white solid (15 mg, 34 %): RP-HPLC: > 99 %, (t_R = 14.23, *k* = 3.91). ^{1}H NMR (400 MHz, CD_3OD) δ 7.48 – 7.43 (m, 1H), 7.31 – 7.13 (m, 8H), 7.02 – 6.93 (m, 2H), 6.78 – 6.70 (m, 2H), 5.36 (s, 2H), 4.40 – 4.29 (m, 3H), 4.00 – 3.89 (m, 2H), 2.17 (s, 3H). ^{13}C NMR (101 MHz, CD_3OD) δ 156.33, 150.00, 133.82, 130.45, 130.34, 129.73, 129.59, 128.78, 128.04, 126.44, 124.02, 123.94, 114.05, 110.64, 110.41, 68.93, 67.48, 46.30, 45.92, 19.10. HRMS (ESI-MS): m/z [M+H$^+$] calculated for $C_{24}H_{26}N_3O_2^+$: 388.202, found 388.2024. $C_{24}H_{25}N_3O_2$ x $C_2HF_3O_2$ (501.19).

1-(2-Imino-3-(4-methylbenzyl)-2,3-dihydro-1*H*-benzo[d]imidazol-1-yl)-3-(p-tolyloxy)propan-2-ol hydrotrifluoroacetate (63)

The title compound was synthesized according to the general procedure with **43** (30 mg, 0.085 mmol, 1.5 eq) and **49** (21 µL, 0.13 mmol, 1.5 eq), yielding a hygroscopic white solid (11 mg, 17 %): RP-HPLC: 95 %, (t_R = 14.76, *k* = 4.09). ^{1}H NMR (400 MHz, CD_3OD) δ 7.47 – 7.43 (m, 1H), 7.29 – 7.16 (m, 3H), 7.10 – 6.96 (m, 6H), 6.77 – 6.70 (m, 2H), 5.30 (s, 2H), 4.39 – 4.28 (m, 3H),

4.00 – 3.89 (m, 2H), 2.21 (s, 3H), 2.17 (s, 3H). HRMS (ESI-MS): m/z [M+H+] calculated for $C_{25}H_{28}N_3O_2^+$: 402.2176, found 402.2182. $C_{25}H_{27}N_3O_2$ x $C_2HF_3O_2$ (515.20).

1-(3-(2-Fluorobenzyl)-2-imino-2,3-dihydro-1*H*-benzo[d]imidazol-1-yl)-3-(p-tolyloxy)propan-2-ol hydrotrifluoro-acetate (64)

The title compound was synthesized according to the general procedure with **44** (19 mg, 0.08 mmol, 1 eq) and **49** (17 µL, 0.10 mmol, 1.3 eq), yielding a hygroscopic white solid (27 mg, 66 %): RP-HPLC: > 99 %, (t_R = 14.17, k = 3.89). ^{1}H NMR (400 MHz, CD_3OD) δ 7.61 – 7.55 (m, 1H), 7.45 – 7.08 (m, 9H), 6.90 – 6.83 (m, 2H), 5.54 (s, 2H), 4.54 – 4.41 (m, 3H), 4.13 – 4.01 (m, 2H), 2.29 (s, 3H). ^{13}C NMR (101 MHz, CD_3OD) δ 160.50 (d, J = 322.2 Hz), 156.32, 151.06, 130.40 (d, J = 11.4 Hz), 129.58 (d, J = 10.4 Hz), 129.58, 128.36 (d, J = 3.4 Hz), 125.96, 124.55 (d, J = 3.5 Hz), 124.08, 123.96, 120.83 (d, J = 14.5 Hz), 115.39, 115.29, 114.05, 110.68, 110.24, 68.90, 67.51, 46.31, 40.96 (d, J = 3.7 Hz), 19.10. HRMS (ESI-MS): m/z [M+H+] calculated for $C_{24}H_{25}FN_3O_2^+$: 406.1925, found 406.1929. $C_{24}H_{24}FN_3O_2$ x $C_2HF_3O_2$ (519.18).

1-(3-(3-fluorobenzyl)-2-imino-2,3-dihydro-1*H*-benzo[d]imidazol-1-yl)-3-(p-tolyloxy)propan-2-ol hydrotrifluoro-acetate (65)

The title compound was synthesized according to the general procedure with **45** (30 mg, 0.12 mmol, 1 eq) and **49** (31 µL, 0.19 mmol, 1.5 eq), yielding a hygroscopic white solid (37 mg, 57 %): RP-HPLC: > 99 %, (t_R = 14.18, k = 3.89). ^{1}H NMR (400 MHz, CD_3OD) δ 7.50 – 7.45 (m, 1H), 7.32 – 7.19 (m, 4H), 7.03 – 6.93 (m, 5H), 6.78 – 6.72 (m, 2H), 5.39 (s, 2H), 4.38 – 4.29 (m, 3H), 4.01 – 3.91 (m, 2H), 2.17 (s, 3H). HRMS (ESI-MS): m/z [M+H+] calculated for $C_{24}H_{25}FN_3O_2^+$: 406.1925, found 406.193. $C_{24}H_{24}FN_3O_2$ x $C_2HF_3O_2$ (519.18).

1-(3-(4-Fluorobenzyl)-2-imino-2,3-dihydro-1*H*-benzo[d]imidazol-1-yl)-3-(p-tolyloxy)propan-2-ol hydrotrifluoro-acetate (66)

The title compound was synthesized according to the general procedure with **46** (50 mg, 0.21 mmol, 1 eq) and **49** (51 µL, 0.31 mmol, 1.5 eq), yielding a hygroscopic white solid (22 mg, 21 %): RP-HPLC: > 99 %, (t_R = 14.30, k = 3.93). ^{1}H NMR (400 MHz, CD_3OD) δ 7.51 – 7.41 (m, 1H), 7.30 – 7.16 (m, 5H), 7.04 – 6.93 (m, 4H), 6.78 – 6.69 (m, 2H), 5.34 (s, 2H), 4.42 – 4.27 (m, 3H), 4.01 – 3.87 (m, 2H), 2.16 (s, 3H). HRMS (ESI-MS): m/z [M+H+] calculated for $C_{24}H_{25}FN_3O_2^+$: 406.1925, found 406.193. $C_{24}H_{24}FN_3O_2$ x $C_2HF_3O_2$ (519.18).

1-(3-Benzyl-2-imino-2,3-dihydro-1*H*-benzo[d]imidazol-1-yl)-3-(o-tolyloxy)propan-2-ol hydrotrifluoroacetate (67)

The title compound was synthesized according to the general procedure with **5** (20 mg, 0.09 mmol, 1 eq) and **50** (18 µL, 0.12 mmol, 1.3 eq), yielding a hygroscopic white solid (29 mg, 64 %): RP-HPLC: > 99 %, (t_R = 14.37, k = 3.96). ^{1}H NMR (400 MHz, CD_3OD) δ 7.44 – 7.40 (m, 1H), 7.30 – 7.15 (m, 8H), 7.07 – 7.00 (m, 2H), 6.82 – 6.74 (m, 2H), 5.37 (s, 2H), 4.43 – 4.33 (m, 3H), 4.06 – 3.96 (m, 2H), 2.16 (s, 3H). ^{13}C NMR (101 MHz, CD_3OD) δ 156.48, 151.07, 133.81, 130.47, 130.38, 129.77, 128.78, 128.05, 126.67, 126.45, 126.34, 124.02, 123.97, 120.75, 111.00, 110.49, 110.41, 69.18, 67.72, 46.50, 45.95, 14.97 . HRMS (ESI-MS): m/z [M+H+] calculated for $C_{24}H_{26}N_3O_2^+$: 388.202, found 388.2023. $C_{24}H_{25}N_3O_2$ x $C_2HF_3O_2$ (501.19).

1-(2-Imino-3-(4-methylbenzyl)-2,3-dihydro-1*H*-benzo[d]imidazol-1-yl)-3-(o-tolyloxy)propan-2-ol hydrotrifluoro-acetate (68)

The title compound was synthesized according to the general procedure with **43** (30 mg, 0.09 mmol, 1.5 eq) and **50** (20 µL, 0.13 mmol, 1.5 eq), yielding a hygroscopic white solid (15 mg, 23 %): RP-HPLC: > 99 %, (t_R = 14.95, k = 4.16). ^{1}H NMR (400 MHz, CD_3OD) δ 7.55 – 7.52 (m, 1H), 7.41 – 7.31 (m, 3H), 7.21 – 7.13 (m, 6H), 6.93 – 6.86 (m, 2H), 5.44 (s, 2H), 4.50 (s, 3H), 4.17 – 4.09 (m, 2H), 2.33 (s, 3H), 2.27 (s, 3H). HRMS (ESI-MS): m/z [M+H+] calculated for $C_{25}H_{28}N_3O_2^+$: 402.2176, found 402.218. $C_{25}H_{27}N_3O_2$ x $C_2HF_3O_2$ (515.20).

1-(3-(2-Fluorobenzyl)-2-imino-2,3-dihydro-1*H*-benzo[d]imidazol-1-yl)-3-(o-tolyloxy)propan-2-ol hydrotrifluoro-acetate (69)

The title compound was synthesized according to the general procedure with **44** (22 mg, 0.091 mmol, 1 eq) and **50** (21 µL, 0.14 mmol, 1.5 eq), yielding a hygroscopic white solid (20 mg, 43 %): RP-HPLC: > 99 %, (t_R = 14.20, k = 3.90). ^{1}H NMR (400 MHz, CD_3OD) δ 7.45 – 7.39 (m, 1H), 7.33 – 7.00 (m, 9H), 6.82 – 6.73 (m, 2H), 5.43 (s, 2H), 4.45 – 4.32 (m, 3H), 4.07 – 3.95 (m, 2H), 2.16 (s, 3H).. HRMS (ESI-MS): m/z [M+H+] calculated for $C_{24}H_{25}FN_3O_2^+$: 406.1925, found 406.193. $C_{24}H_{24}FN_3O_2$ x $C_2HF_3O_2$ (519.18).

1-(3-(3-Fluorobenzyl)-2-imino-2,3-dihydro-1*H*-benzo[d]imidazol-1-yl)-3-(o-tolyloxy)propan-2-ol hydrotrifluoro-acetate (70)

The title compound was synthesized according to the general procedure with **45** (30 mg, 0.12 mmol, 1 eq) and **50** (28 µL, 0.19 mmol, 1.5 eq), yielding a hygroscopic white solid (34 mg, 52 %): RP-HPLC: > 99 %, (t_R = 14.38, k = 3.96). ^{1}H NMR (400 MHz, CD_3OD) δ 7.46 – 7.40 (m, 1H), 7.31 – 7.19 (m, 4H), 7.08 – 6.93 (m, 5H), 6.82 – 6.74 (m, 2H), 5.39 (s, 2H), 4.44 – 4.34 (m, 3H), 4.07 – 3.96 (m, 2H), 2.16 (s, 3H). ^{13}C NMR (101 MHz, CD_3OD) δ 163.17 (d, J = 246.2 Hz), 156.48, 151.11, 136.59 (d, J = 6.9 Hz), 130.72 (d, J = 8.2 Hz), 130.49, 130.38, 129.67, 126.67, 126.33, 124.14, 124.07, 122.19 (d, J = 3.0 Hz), 120.75, 114.83 (d, J = 21.2 Hz), 113.43 (d, J = 23.0 Hz), 111.00, 110.50, 110.34, 69.15, 67.71, 46.55, 45.42 (d, J = 2.0 Hz), 14.99. HRMS (ESI-MS): m/z [M+H$^+$] calculated for $C_{24}H_{25}FN_3O_2^+$: 406.1925, found 406.1928. $C_{24}H_{24}FN_3O_2$ x $C_2HF_3O_2$ (519.18).

1-(3-(4-Fluorobenzyl)-2-imino-2,3-dihydro-1*H*-benzo[d]imidazol-1-yl)-3-(o-tolyloxy)propan-2-ol hydrotrifluoro-acetate (71)

The title compound was synthesized according to the general procedure with **46** (50 mg, 0.21 mmol, 1 eq) and **50** (47 µL, 0.31 mmol, 1.5 eq), yielding a hygroscopic white solid (23 mg, 21 %): RP-HPLC: > 99 %, (t_R = 14.42, k = 3.97). ^{1}H NMR (400 MHz, CD_3OD) δ 7.57 – 7.52 (m, 1H), 7.44 – 7.30 (m, 5H), 7.20 – 7.07 (m, 4H), 6.94 – 6.86 (m, 2H), 5.47 (s, 2H), 4.55 – 4.46 (m, 3H), 4.18 – 4.08 (m, 2H), 2.27 (s, 3H). ^{13}C NMR (101 MHz, CD_3OD) δ 162.62 (d, J = 245.8 Hz), 156.46, 150.99, 130.48, 130.37, 129.85 (d, J = 3.0 Hz), 129.64, 128.62 (d, J = 8.7 Hz), 126.66, 126.32, 124.06, 124.01, 120.75, 115.51 (d, J = 21.7 Hz), 111.01, 110.45, 110.41, 69.18, 67.68, 46.51, 45.29, 14.96. HRMS (ESI-MS): m/z [M+H$^+$] calculated for $C_{24}H_{25}FN_3O_2^+$: 406.1925, found 406.1931. $C_{24}H_{24}FN_3O_2$ x $C_2HF_3O_2$ (519.18).

1-(2-Imino-3-(4-methylbenzyl)-2,3-dihydro-1*H*-benzo[d]imidazol-1-yl)-3-phenoxypropan-2-ol hydrotrifluoroace-tate (72)

The title compound was synthesized according to the general procedure with **43** (15 mg, 0.04 mmol, 1 eq) and **51** (8 µL, 0.06 mmol, 1.3 eq), yielding a hygroscopic white solid (4 mg, 13 %): RP-HPLC: > 99 %, (t_R = 14.16, k = 3.88). ^{1}H NMR (400 MHz, CD_3OD) δ 7.60 – 7.56 (m, 1H), 7.41 – 7.28 (m, 5H), 7.22 – 7.15 (m, 4H), 7.01 – 6.95 (m, 3H), 5.43 (s, 2H), 4.51 – 4.43 (m, 3H), 4.15 – 4.05 (m, 2H), 2.33 (s, 3H). ^{13}C NMR (101 MHz, CD_3OD) δ 158.43, 150.94, 138.14, 130.43, 129.73, 129.35, 129.21, 127.62, 126.50, 123.98, 123.92, 120.97, 114.18, 110.59, 110.49, 68.81, 67.45, 46.26, 45.77, 19.67 . HRMS (ESI-MS): m/z [M+H$^+$] calculated for $C_{24}H_{26}N_3O_2^+$: 388.202, found 388.2013. $C_{24}H_{25}N_3O_2$ x $C_2HF_3O_2$ (501.19).

1-(3-(2-Fluorobenzyl)-2-imino-2,3-dihydro-1*H*-benzo[d]imidazol-1-yl)-3-phenoxypropan-2-ol hydrotrifluoroace-tate (73)

The title compound was synthesized according to the general procedure with **44** (19 mg, 0.08 mmol, 1 eq) and **51** (14 µL, 0.10 mmol, 1.3 eq), yielding a hygroscopic white solid (24 mg, 60 %): RP-HPLC: 96 %, (t_R = 13.45, k = 3.64). ^{1}H NMR (400 MHz, CD_3OD) δ 7.62 – 7.55 (m, 1H), 7.46 – 7.14 (m, 9H), 7.03 – 6.91 (m, 3H), 5.55 (s, 2H), 4.56 – 4.42 (m, 3H), 4.18 – 4.04 (m, 2H). HRMS (ESI-MS): m/z [M+H$^+$] calculated for $C_{23}H_{23}FN_3O_2^+$: 392.1769, found 392.1773. $C_{23}H_{22}FN_3O_2$ x $C_2HF_3O_2$ (505.16).

1-(3-(3-Fluorobenzyl)-2-imino-2,3-dihydro-1*H*-benzo[d]imidazol-1-yl)-3-phenoxypropan-2-ol hydrotrifluoroace-tate (74)

The title compound was synthesized according to the general procedure with **45** (30 mg, 0.12 mmol, 1 eq) and **51** (25 µL, 0.19 mmol, 1.5 eq), yielding a hygroscopic white solid (5 mg, 9 %): RP-HPLC: 95 %, (t_R = 13.67, k = 3.71). ^{1}H NMR (400 MHz, CD_3OD) δ 7.50 – 7.46 (m, 1H), 7.32 – 7.15 (m, 6H), 7.02 – 6.82 (m, 6H), 5.38 (s, 2H), 4.41 – 4.31 (m, 3H), 4.05 – 3.94 (m, 2H). HRMS (ESI-MS): m/z [M+H$^+$] calculated for $C_{23}H_{23}FN_3O_2^+$: 392.1769, found 392.1773. $C_{23}H_{22}FN_3O_2$ x $C_2HF_3O_2$ (505.16).

1-(3-(4-Fluorobenzyl)-2-imino-2,3-dihydro-1H-benzo[d]imidazol-1-yl)-3-phenoxypropan-2-ol hydrotrifluoroace-tate (75)

The title compound was synthesized according to the general procedure with **46** (30 mg, 0.12 mmol, 1 eq) and **51** (25.3 µL, 0.19 mmol, 1.5 eq), yielding a hygroscopic white solid (30.0 mg, 48 %): RP-HPLC: > 99 %, (t_R = 13.59, k = 3.69). ^{1}H NMR (400 MHz, CD_3OD) δ 7.62 – 7.55 (m, 1H), 7.43 – 7.25 (m, 7H), 7.19 – 7.06 (m, 2H), 7.03 – 6.93 (m, 3H), 5.47 (s, 2H), 4.55 – 4.42 (m, 3H), 4.18 – 4.04 (m, 2H). ^{13}C NMR (101 MHz, CD_3OD) δ 162.61 (d, J = 246.2 Hz), 158.41, 150.93, 130.46, 129.86 (d, J = 3.5 Hz), 129.61, 129.20, 128.62 (d, J = 8.2 Hz), 124.06, 123.98, 120.95, 115.52 (d, J = 21.7 Hz), 114.19, 110.67, 110.34, 68.80, 67.44, 46.29, 45.28. HRMS (ESI-MS): m/z [M+H$^+$] calculated for $C_{23}H_{23}FN_3O_2^+$: 392.1769, found 392.1774. $C_{23}H_{22}FN_3O_2$ x $C_2HF_3O_2$ (505.16).

General procedure for the synthesis of ***76-77***

46 was dissolved in butan-2-one at room temperature, followed by addition of the corresponding bromide (**6** or **16**, 6 eq). The approach was heated to 90 °C and stirred for 48 h. The crude product was purified by HPLC, yielding ***76-77.***

1-(4-Fluorobenzyl)-3-(4-phenylbutyl)-1,3-dihydro-2*H*-benzo[d]imidazol-2-imine hydrotrifluoroacetate (76)

The title compound was synthesized according to the general procedure with **46** (40 mg, 0.17 mmol, 1 eq) and **6** (175 µL, 1.0 mmol, 6 eq), yielding a hygroscopic white solid (21 mg, 27 %): RP-HPLC: 99 %, (t_R = 14.89, *k* = 4.13). ^1H NMR (400 MHz, CD_3OD) δ 7.42 – 7.37 (m, 1H), 7.33 – 6.93 (m, 12H), 5.32 (s, 2H), 4.14 (t, *J* = 7.3 Hz, 2H), 2.56 (t, *J* = 7.5 Hz, 2H), 1.82 – 1.70 (m, 2H), 1.66 – 1.57 (m, 2H). ^{13}C NMR (101 MHz, CD_3OD) δ 162.39 (d, *J* = 197.8 Hz), 149.67, 141.47, 129.93 (d, *J* = 2.3 Hz), 129.80, 129.59, 128.60 (d, *J* = 8.7 Hz), 128.03, 128.00, 125.62, 124.16, 124.00, 115.56 (d, *J* = 22.1 Hz), 110.45, 110.29, 45.19, 42.84, 34.89, 27.95, 27.00. HRMS (ESI-MS): m/z [M+H$^+$] calculated for $C_{24}H_{25}FN_3^+$: 374.2027, found 374.2033. $C_{24}H_{24}FN_3$ x $C_2HF_3O_2$ (487.19).

1-(Cyclopropylmethyl)-3-(4-fluorobenzyl)-1,3-dihydro-2*H*-benzo[d]imidazol-2-imine hydrotrifluoroacetate (77)

The title compound was synthesized according to the general procedure with **46** (40 mg, 0.17 mmol, 1 eq) and **16** (97 µL, 1.0 mmol, 6 eq), yielding a hygroscopic white solid (16 mg, 23 %): RP-HPLC: 97 %, (t_R = 12.40, *k* = 3.28). ^1H NMR (400 MHz, CD_3OD) δ 7.52 – 7.44 (m, 1H), 7.32 – 7.17 (m, 5H), 7.07 – 6.96 (m, 2H), 5.35 (s, 2H), 4.07 (d, *J* = 7.1 Hz, 2H), 1.34 – 1.20 (m, 1H), 0.60 – 0.49 (m, 2H), 0.47 – 0.38 (m, 2H). ^{13}C NMR (101 MHz, CD_3OD) δ 161.27 (d, *J* = 246.2 Hz), 148.36, 128.61, 128.55 (d, *J* = 3.2 Hz), 128.27, 127.24 (d, *J* = 8.7 Hz), 122.81, 122.62, 114.21 (d, *J* = 22.1 Hz), 109.19, 109.06, 43.86, 7.94, 1.42. HRMS (ESI-MS): m/z [M+H$^+$] calculated for $C_{18}H_{19}FN_3^+$: 296.1558, found 296.1565. $C_{18}H_{18}FN_3$ x $C_2HF_3O_2$ (409.14).

1-Benzyl-6-fluoro-1,3-dihydro-2*H*-benzo[d]imidazol-2-imine & 1-Benzyl-5-fluoro-1,3-dihydro-2*H*-benzo[d]imidazol-2-imine (79)

The title compounds were synthesized by dissolving **78** (120 mg, 0.79 mmol, 1 eq) in 286 µL of a 20 M NaOH solution (7.2 eq) at 40 °C. After stirring the mixture for 15 min until the starting material dissolved, yielding a viscous, red solution, 10 mL of acetone were added, resulting in the deprotonated product precipitating into a beige solid. Subsequently, **10** was added to the suspension. The approach was then heated to reflux at 60 °C and stirred for 2 h until the product was dissolved. Following evaporation of acetone under reduced pressure, the residue was dispersed in 10 mL of water leading to a yellow dispersion and a clumpy, brown solid, which remained at the bottom of the flask. The desired, yellow product was decanted and extracted using ethyl acetate (3 x 15 mL). The combined organic layers were washed with 1 M sodium hydroxide solution (3 x 15 mL) to remove residual **78.** Following solvent removal by rotary evaporation, **79** was used for the synthesis of **80-84** without further purification, yielding a brown solid containing both isomers of **79** (63 mg, 64%). ^1H NMR (400 MHz, CD_3OD, mixture of isomers) δ 7.37 – 7.15 (m, 6H), 7.00 – 6.93 (m, 1H), 6.85 – 6.65 (m, 2H), 5.26 (d, *J* = 2.3 Hz, 2H). HRMS (ESI-MS): m/z [M+H$^+$] calculated for $C_{14}H_{13}FN_3^+$: 242.1088, found 242.1088. $C_{14}H_{12}FN_3$ x HBr (321.03).

General procedure for the synthesis of ***80-84***

79 was dissolved in ethanol at room temperature, followed by addition of the corresponding epoxide **47-51** (1.2-1.4 eq). The approach was heated to reflux at 100 °C and stirred for 48 h. The crude product was purified by HPLC, yielding **80-84**, each as mixtures of isomers.

1-(3-Benzyl-5-fluoro-2-imino-2,3-dihydro-1*H*-benzo[d]imidazol-1-yl)-3-(4-chlorophenoxy)propan-2-ol hydrotrifluoroacetate & 1-(3-benzyl-6-fluoro-2-imino-2,3-dihydro-1*H*-benzo[d]imidazol-1-yl)-3-(4-chlorophenoxy)propan-2-ol hydrotrifluoroacetate (80)

The title compound (mixture of isomers) was synthesized according to the general procedure with **79** (15 mg, 0.06 mmol, 1 eq) and **47** (15 mg, 0.08 mmol, 1.3 eq), yielding a hygroscopic beige solid (13 mg, 39 %): RP-HPLC: > 99 %, (t_R = 14.63, *k* = 4.04). ^1H NMR (400 MHz, CD_3OD, mixture of isomers) δ 7.49 – 7.10 (m, 9H), 7.03 – 6.93 (m, 1H), 6.87 – 6.81 (m, 2H), 5.35 (d, *J* = 2.4 Hz, 2H), 4.41 – 4.28 (m, 3H), 4.03 – 3.93 (m, 2H). HRMS (ESI-MS): m/z [M+H$^+$] calculated for $C_{23}H_{22}ClFN_3O_2^+$: 426.1379, found 426.1385. $C_{23}H_{21}ClFN_3O_2$ x $C_2HF_3O_2$ (539.12).

1-(3-Benzyl-5-fluoro-2-imino-2,3-dihydro-1*H*-benzo[d]imidazol-1-yl)-3-(4-fluorophenoxy)propan-2-ol hydrotrifluoroacetate & 1-(3-Benzyl-6-fluoro-2-imino-2,3-dihydro-1*H*-benzo[d]imidazol-1-yl)-3-(4-fluorophenoxy)propan-2-ol hydrotrifluoroacetate (81)

The title compound (mixture of isomers) was synthesized according to the general procedure with **79** (10 mg, 0.04 mmol, 1 eq) and **48** (8 µL, 0.05 mmol, 1.3 eq), yielding a hygroscopic beige solid (9 mg, 43 %): RP-HPLC: 96 %, (t_R = 13.79, *k* = 3.75). ^1H NMR (400 MHz, CD_3OD, mixture of isomers) δ 7.61 – 7.22 (m, 7H), 7.16 – 6.93 (m, 5H), 5.47 (d, *J* = 2.4 Hz, 2H), 4.54 – 4.39 (m, 3H), 4.14 – 4.02 (m, 2H). HRMS (ESI-MS): m/z [M+H$^+$] calculated for $C_{23}H_{22}F_2N_3O_2^+$: 410.1675, found 410.1678. $C_{23}H_{21}F_2N_3O_2$ x $C_2HF_3O_2$ (523.15).

1-(3-Benzyl-5-fluoro-2-imino-2,3-dihydro-1*H*-benzo[d]imidazol-1-yl)-3-(p-tolyloxy)propan-2-ol hydrotrifluoroacetate & 1-(3-Benzyl-6-fluoro-2-imino-2,3-dihydro-1*H*-benzo[d]imidazol-1-yl)-3-(p-tolyloxy)propan-2-ol hydrotrifluoroacetate (82)

The title compound (mixture of isomers) was synthesized according to the general procedure with **79** (13 mg, 0.05 mmol, 1 eq) and **49** (12 µL, 0.08 mmol, 1.4 eq), yielding a hygroscopic beige solid (8 mg, 29 %): RP-HPLC: > 99 %, (t_R = 14.31, *k* = 3.93). ^1H NMR (400 MHz, CD_3OD, mixture of isomers) δ 7.48 – 7.09 (m, 4H), 7.04 – 6.91 (m, 7H), 6.78 – 6.69 (m, 2H), 5.34 (d, *J* = 2.3 Hz, 2H), 4.41 – 4.27 (m, 3H), 4.01 – 3.89 (m, 2H), 2.17 (s, 3H). HRMS (ESI-MS): m/z [M+H$^+$] calculated for $C_{24}H_{25}FN_3O_2^+$: 406.1925, found 406.1932. $C_{24}H_{24}FN_3O_2$ x $C_2HF_3O_2$ (519.18).

1-(3-Benzyl-5-fluoro-2-imino-2,3-dihydro-1*H*-benzo[d]imidazol-1-yl)-3-(o-tolyloxy)propan-2-ol hydrotrifluoroacetate & 1-(3-benzyl-6-fluoro-2-imino-2,3-dihydro-1*H*-benzo[d]imidazol-1-yl)-3-(o-tolyloxy)propan-2-ol hydrotrifluoroacetate (83)

The title compound (mixture of isomers) was synthesized according to the procedure with **79** (10 mg, 0.04 mmol, 1 eq) and **50** (8 µL, 0.05 mmol, 1.3 eq), yielding a hygroscopic beige solid (4 mg, 16 %): RP-HPLC: 98 %, (t_R = 14.44, *k* = 3.97). ^1H NMR (400 MHz, CD_3OD, mixture of isomers) δ 7.44 – 6.94 (m, 10H), 6.84 – 6.74 (m, 2H), 5.35 (d, *J* = 2.2 Hz, 2H), 4.44 – 4.31 (m, 3H), 4.06 – 3.96 (m, 2H), 2.15 (s, 3H). HRMS (ESI-MS): m/z [M+H$^+$] calculated for $C_{24}H_{25}FN_3O_2^+$: 406.1925, found 406.1928. $C_{24}H_{24}FN_3O_2$ x $C_2HF_3O_2$ (519.18).

1-(3-Benzyl-5-fluoro-2-imino-2,3-dihydro-1*H*-benzo[d]imidazol-1-yl)-3-phenoxypropan-2-ol hydrotrifluoroacetate & 1-(3-Benzyl-6-fluoro-2-imino-2,3-dihydro-1*H*-benzo[d]imidazol-1-yl)-3-phenoxypropan-2-ol hydrotrifluoroacetate (84)

The title compound (mixture of isomers) was synthesized according to the general procedure with **79** (13 mg, 0.05 mmol, 1 eq) and **51** (9 µL, 0.07 mmol, 1.2 eq), yielding a hygroscopic beige solid (6 mg, 23 %): RP-HPLC: 99 %, (t_R = 13.76, *k* = 3.74). ^1H NMR (400 MHz, CD_3OD, mixture of isomers) δ 7.49 – 7.10 (m, 9H), 7.02 – 6.83 (m, 4H), 5.34 (d, *J* = 2.4 Hz, 2H), 4.43 – 4.27 (m, 3H), 4.05 – 3.93 (m, 2H). HRMS (ESI-MS): m/z [M+H$^+$] calculated for $C_{23}H_{23}FN_3O_2^+$: 392.1769, found 392.1771. $C_{23}H_{22}FN_3O_2$ x $C_2HF_3O_2$ (505.16).

1-(*tert*-Butyl) 4-methyl 4-cyclohexylpiperidine-1,4-dicarboxylate (86)

85 (72 mg, 0.23 mmol, 1.0 eq) was dissolved in DMF (30 mL) and treated with K_2CO_3 (106 mg, 0.76 mmol, 3.0 eq). After the addition of methyl iodide (0.02 mL, 0.32 mmol, 1.2 eq), the reaction mixture was stirred at room temperature overnight. Upon completion, the mixture was quenched with saturated NaCl solution and extracted with EtOAc (3 × 50 mL). The combined organic phases were washed with 1 M HCl (2 × 50 mL), dried over Na_2SO_4 and concentrated under reduced pressure. The crude product was purified by preparative HPLC yielding the title compound as a yellow solid (67 mg, 90 %).^1H-NMR (400 MHz, $CDCl_3$): δ 3.96 (s, 2H), 3.68 (s, 3H), 2.68 (s, 2H), 2.05 (dd, J = 13.6, 2.8 Hz, 2H), 1.81 – 1.70 (m, 2H), 1.69 – 1.57 (m, 3H), 1.42 (s, 9H), 1.33 (m, J = 12.1, 3.1 Hz, 3H), 1.23 – 1.02 (m, 3H), 0.92 (m, 2H). ^{13}C-NMR (101 MHz, $CDCl_3$), δ 175.48, 154.86, 79.31, 77.23, 51.40, 49.64, 46.54, 30.84, 28.44, 27.70, 26.84, 26.41. HRMS (ESI-MS): m/z [M+Na]$^+$ calculated for $C_{18}H_{31}NO_4Na^+$: 348.2145, found: 348.2150. $C_{18}H_{31}NO_4$ (325.45).

***tert*-Butyl 4-cyclohexyl-4-(hydroxymethyl)piperidine-1-carboxylate (87)**

To a solution of **86** (533 mg, 1.64 mmol, 1.0 eq) in THF (50 mL), $LiAlH_4$ (125 mg, 3.28 mmol, 2.0 eq) was added portion wise at 0 °C. After complete addition, the ice bath was removed and the reaction mixture was stirred at room temperature for 4 days. The reaction was quenched by adding water (2 mL), followed by filtration over Celite. The filtrate was concentrated under reduced pressure and the aqueous phase was extracted with EtOAc (3 × 50 mL). The combined organic layers were dried over $MgSO_4$ and concentrated under reduced pressure. The crude product was purified by preparative HPLC yielding the title compound as a colorless oil (234 mg, 48 %).^1H-NMR (400 MHz, $CDCl_3$): δ 3.59 (s, 2H), 3.52 (dt, J = 13.3, 5.2 Hz, 2H), 3.20 (ddd, J = 13.3, 8.9, 4.1 Hz, 2H), 2.03 (d, J = 4.3 Hz, 2H), 1.83 – 1.69 (m, 4H), 1.68 – 1.62 (m, 1H), 1.54 – 1.46 (m, 2H), 1.44 (s, 9H), 1.42 – 1.33 (m, 2H), 1.22 – 0.98 (m, 5H). ^{13}C-NMR (101 MHz, $CDCl_3$): δ 155.15, 79.37, 64.26, 42.54, 39.50, 37.64, 29.04, 28.48, 27.36, 27.17, 26.73. HRMS (ESI-MS): m/z [M+H]$^+$ calculated for $C_{17}H_{32}NO_3^+$: 298.2377, found: 298.2374. $C_{17}H_{31}NO_3$ (297.44).

***tert*-Butyl 4-cyclohexyl-4-(((methylsulfonyl)oxy)methyl)piperidine-1-carboxylate (88)**

A solution of **87** (94 mg, 0.32 mmol, 1.0 eq) in DCM (35 mL) was cooled to 0 °C, and TEA (89 µL, 0.64 mmol, 2.0 eq) was added. After 5 minutes, methanesulfonyl chloride (50 µL, 0.64 mmol, 2.0 eq) was added dropwise. Upon completion of the addition, the ice bath was removed, and the reaction mixture was stirred overnight at room temperature. The reaction was quenched by the addition of saturated NH_4Cl solution. The product was isolated by extraction with DCM (3 × 50 mL), and the combined

organic layers were dried over $MgSO_4$ and concentrated under reduced pressure. The crude product was purified by preparative HPLC, affording the desired compound as a white solid (91 mg, 77 %). ^{1}H-NMR (400 MHz, $CDCl_3$): δ 4.16 (s, 2H), 3.58 – 3.49 (m, 2H), 3.25 (ddd, J = 13.4, 8.9, 3.8 Hz, 2H), 3.01 (s, 3H), 1.84 – 1.48 (m, 10H), 1.44 (s, 9H), 1.26 – 0.97 (m, 5H). ^{13}C-NMR (101 MHz, $CDCl_3$): δ 154.93, 79.56, 70.87, 42.46, 38.97, 37.29, 37.04, 29.20, 28.45, 27.17, 26.93, 26.53. HRMS (ESI-MS): m/z $[M+Na]^+$ calculated for $C_{18}H_{33}NO_5SNa^+$: 398.1972, found: 398.1972. $C_{18}H_{33}NO_5S$ (375.52).

*General procedure for the synthesis of **89** and **90***

A solution of **88** (1.0 eq) in DMF was treated with the corresponding sodium salt (3.0 eq). The reaction mixture was stirred and heated at 110 °C overnight. Upon completion, the solvent was removed under reduced pressure. The crude residue was purified by preparative HPLC, yielding the desired product.

tert-Butyl 4-((1_H_-1,2,4-triazol-1-yl)methyl)-4-cyclohexylpiperidine-1-carboxylate hydrotrifluoroacetate (89)

To a solution of **88** (101 mg, 0.27 mmol) in DMF (25 mL) sodium 1,2,4-triazol-1-ide (74 mg, 0.81 mmol) was added. The reaction mixture was implemented according to the general procedure, yielding a colorless oil (70 mg, 56 %). ^{1}H-NMR (400 MHz, $CDCl_3$): δ 8.17 (s, 1H), 7.97 (s, 1H), 4.19 (s, 2H), 3.45 – 3.40 (m, 4H), 1.83 – 1.62 (m, 7H), 1.41 (s, 9H), 1.37 (d, J = 2.9 Hz, 1H), 1.32 – 1.25 (m, 2H), 1.23 – 1.04 (m, 3H), 0.94 (qd, J = 12.1, 2.8 Hz, 2H). ^{13}C-NMR (101 MHz, $CDCl_3$): δ 155.00, 149.99, 143.82, 79.71, 77.27, 52.86, 40.20, 39.06, 37.87, 29.58, 28.38, 26.98, 26.30. HRMS (ESI-MS): m/z $[M+H]^+$ calculated for $C_{19}H_{33}N_4O_2^+$: 349.2598, found: 349.2598. $C_{19}H_{32}N_4O_2 \times C_2HF_3O_2$ (462.51).

tert-Butyl 4-((1_H_-imidazol-1-yl)methyl)-4-cyclohexylpiperidine-1-carboxylate hydrotrifluoroacetate (90)

To a solution of **88** (260 mg, 0.69 mmol) in DMF (20 mL) sodium imidazolide (189 mg, 2.07 mmol) was added. The reaction mixture was implemented according to the general procedure, yielding a white solid (63 mg, 20 %). ^{1}H-NMR (600 MHz, $CDCl_3$): δ 8.51 (s, 1H), 7.38 (s, 1H), 6.99 (s, 1H), 4.07 (s, 2H), 3.66 – 3.58 (m, 2H), 3.30 – 3.22 (m, 2H), 1.88 (d, *J* = 13.2 Hz, 2H), 1.74 (d, *J* = 7.0 Hz, 4H), 1.63 (t, *J* = 11.9 Hz, 1H), 1.43 (s, 9H), 1.28 – 1.09 (m, 6H), 1.00 (qd, *J* = 12.4, 3.2 Hz, 2H). ^{13}C-NMR (151 MHz, $CDCl_3$): δ 154.80, 136.43, 122.39, 121.23, 80.10, 53.60, 38.53, 37.73, 29.39, 28.35, 26.89, 26.31, 26.25. HRMS (ESI-MS): m/z $[M+H]^+$ calculated for $C_{20}H_{34}N_3O_2^+$: 348.2649, found: 348.2649. $C_{20}H_{33}N_3O_2 \times C_2HF_3O_2$ (461.52).

*General procedure for the synthesis of **91** and **92***

To a solution of **89** or **90** in 10 mL DCM, 2 mL TFA was added dropwise at 0 °C under stirring. The reaction mixture was allowed to warm to room temperature and stirred overnight. After completion, the solvent and excess of TFA were removed under reduced pressure. The resulting orange oil was used without further purification.

4-((1_H_-1,2,4-Triazol-1-yl)methyl)-4-cyclohexylpiperidine dihydrotrifluoroacetate (91)

The title compound was synthesized with **89** (69 mg, 0.20 mmol) according to the general procedure, yielding an orange oil that was used without further purification. ^{1}H-NMR (400 MHz, DMSO-*d*$_6$): δ 8.55 (s, 1H), 8.40 (s, 2H), 8.01 (s, 1H), 4.28 (s, 2H), 3.20 – 3.04 (m, 5H), 1.80 – 1.65 (m, 7H), 1.45 (m, 2H), 1.30 (t, J = 12.0 Hz, 1H), 1.20 – 1.03 (m, 3H), 0.98 – 0.86 (m, 2H). ^{13}C-NMR (101 MHz, DMSO-*d*$_6$): δ 163.70, 147.98, 77.23, 53.04, 39.55, 37.11, 31.98, 29.69, 26.72, 26.14. HRMS (ESI-MS): m/z $[M+H]^+$ calculated for $C_{14}H_{25}N_4^+$: 249.2074, found: 249.2074. $C_{14}H_{24}N_4 \times C_4H_2F_6O_4$ (476.41).

4-((1_H_-Imidazol-1-yl)methyl)-4-cyclohexylpiperidine dihydrotrifluoroacetate (92)

The title compound was synthesized with **90** (63 mg, 0.14 mmol) according to the general procedure, yielding an orange oil that was used without further purification. ^{1}H-NMR (400 MHz, $CDCl_3$): δ 8.71 (s, 1H), 8.45 (s, 1H), 7.35 (s, 1H), 7.21 (s, 1H), 4.18 (s, 2H), 3.32 – 3.10 (m, 4H), 1.97 (d, *J* = 15.4 Hz, 2H), 1.82 (d, *J* = 13.2 Hz, 2H), 1.72 – 1.61 (m, 4H), 1.46 (td, *J* = 10.5, 5.3 Hz, 2H), 1.29 – 0.95 (m, 6H). ^{13}C-NMR (101 MHz, $CDCl_3$): δ 164.40, 136.19, 123.49, 120.24, 53.48, 39.55, 38.15, 37.52, 32.25, 26.61, 26.03, 25.98, 25.88. HRMS (ESI-MS): m/z $[M+H]^+$ calculated for $C_{15}H_{26}N_3^+$: 248.2121, found: 248.2122. $C_{15}H_{25}N_3 \times C_4H_2F_6O_4$ (475.43).

*General procedure for the synthesis of **93-110***

A solution of **91** or **92** (1.0 eq) and the respective bicyclic carboxylic acid (2.0 eq) in DMF was treated with DIPEA (3.0 eq). After stirring for 5 minutes, HATU (1.1 eq) was added, and the reaction mixture was stirred overnight at room temperature. Following preparative purification, the product was obtained as an oil or solid.

(4-((1_H_-1,2,4-Triazol-1-yl)methyl)-4-cyclohexylpiperidin-1-yl)(2,3-dihydrobenzo [b][1,4] dioxin-2-yl)methanone hydrotrifluoroacetate (93)

The title compound was synthesized with **91** (38 mg, 0,08 mmol), 2,3-dihydrobenzo[*b*][1,4]dioxine-2-carboxylic acid (28 mg, 0.16 mmol), DIPEA (42 µL, 0,24 mmol) and HATU (34 mg, 0.09 mmol) according to the general procedure, yielding an opalescent oil (16 mg, 40 %). RP-HPLC: 98 %, (t_R = 18.89 min, *k* = 5.51). ^{1}H-NMR (400 MHz, $CDCl_3$): δ 8.64 (d, J = 10.3 Hz, 1H), 8.17 (s, 1H), 6.92 – 6.82 (m, 4H), 4.83 (dd, J = 7.9, 2.5 Hz, 1H), 4.45 (m, 1H), 4.35 – 4.24 (m, 3H), 3.85 – 3.55 (m, 4H), 1.94 – 1.69 (m, 7H), 1.57 – 1.34 (m, 3H), 1.29 – 1.11 (m, 3H), 1.01 (tt, J = 12.7, 6.0 Hz, 2H). ^{13}C-NMR (101 MHz, $CDCl_3$): δ 147.85, 143.43, 142.29, 122.37, 121.70, 121.59, 117.47, 117.21, 77.23, 70.66, 65.02, 53.23, 38.37, 37.77, 30.32, 29.25, 26.90, 26.42, 26.27. HRMS (ESI-MS): m/z [M+H]$^+$ calculated for $C_{23}H_{31}N_4O_3^+$: 411.2391, found: 411.2392. $C_{23}H_{30}N_4O_3$ × $C_2HF_3O_2$ (524.54).

(4-((1*H*-1,2,4-Triazol-1-yl)methyl)-4-cyclohexylpiperidin-1-yl)(chroman-3-yl-methanone hydrotrifluoroacetate (94)

The title compound was synthesized with **91** (21 mg, 0,04 mmol), chromane-3-carboxylic acid (14 mg, 0.08 mmol), DIPEA (21 µL, 0,12 mmol) and HATU (19 mg, 0.05 mmol) according to the general procedure, yielding an opalescent oil (6 mg, 28 %). RP-HPLC: 97 %, (t_R = 19.36 min, *k* = 5.68). ^{1}H-NMR (400 MHz, $CDCl_3$): δ 8.22 (s, 1H), 8.03 (s, 1H), 7.15 – 7.04 (m, 2H), 6.91 – 6.80 (m, 2H), 4.33 (dd, J = 12.1, 3.1 Hz, 1H), 4.27 (d, J = 2.6 Hz, 2H), 4.04 (td, J = 10.1, 9.6, 4.8 Hz, 1H), 3.63 (t, J = 5.8 Hz, 3H), 3.19 – 3.07 (m, 2H), 2.85 – 2.72 (m, 1H), 1.90 – 1.67 (m, 7H), 1.53 – 1.34 (m, 3H), 1.18 (m, 4H), 1.01 (m, J = 12.2 Hz, 2H). ^{13}C-NMR (101 MHz, $CDCl_3$): δ 170.88, 150.13, 129.69, 127.61, 120.66, 116.59, 77.23, 67.34, 52.65, 41.15, 40.76, 38.25, 37.28, 35.58, 30.69, 29.45, 28.90, 26.95, 26.40, 26.33. HRMS (ESI-MS): m/z [M+H]$^+$ calculated for $C_{24}H_{33}N_4O_2^+$: 409.2598, found: 409.2606. $C_{24}H_{32}N_4O_2$ × $C_2HF_3O_2$ (522.57).

(4-((1*H*-1,2,4-Triazol-1-yl)methyl)-4-cyclohexylpiperidin-1-yl)(naphthalen-2-yl)-methanone hydrotrifluoroacetate (95)

The title compound was synthesized with **91** (21 mg, 0,04 mmol), 2-naphthoic acid (14 mg, 0.09 mmol), DIPEA (21 µL, 0,12 mmol) and HATU (19 mg, 0.05 mmol) according to the general procedure, yielding a sticky white solid (2 mg, 9 %). RP-HPLC: 99 %, (t_R = 19.79 min, *k* = 5.82). ^{1}H-NMR (400 MHz, $CDCl_3$): δ 8.09 (s, 1H), 7.97 (s, 1H), 7.89 – 7.84 (m, 4H), 7.57 – 7.51 (m, 2H), 7.45 (dd, J = 8.5, 1.5 Hz, 1H), 4.27 (s, 2H), 3.85 (d, J = 52.2 Hz, 2H), 3.53 (s, 2H), 1.84 (d, J = 12.4 Hz, 4H), 1.71 (d, J = 12.1 Hz, 2H), 1.45 (t, J = 12.0 Hz, 2H), 1.18 (m, J = 24.8, 23.3, 10.9 Hz, 5H), 1.00 (s, 2H). ^{13}C-NMR (101 MHz, $CDCl_3$): δ 170.67, 160.10, 133.32, 133.07, 128.42, 128.35, 127.82, 127.11, 126.75, 124.12, 77.22, 67.57, 52.41, 40.83, 39.39, 38.37, 37.19, 29.67, 27.00, 26.41. HRMS (ESI-MS): m/z [M+H]$^+$ calculated for $C_{25}H_{31}N_4O^+$: 403.2492, found: 403.2502. $C_{25}H_{30}N_4O$ × $C_2HF_3O_2$ (516.56).

(4-((1*H*-1,2,4-Triazol-1-yl)methyl)-4-cyclohexylpiperidin-1-yl)(quinolin-2-yl) methanone dihydrotrifluoroacetate (96)

The title compound was synthesized with **91** (14 mg, 0,03 mmol), quinoline-2-carboxylic acid (11 mg, 0.06 mmol), DIPEA (16 µL, 0,09 mmol) and HATU (11 mg, 0.03 mmol) according to the general procedure, yielding a sticky white solid (4 mg, 23 %). RP-HPLC: 95 %, (t_R = 17.29 min, *k* = 4.96). ^{1}H-NMR (400 MHz, $CDCl_3$): δ 8.33 (d, J = 8.5 Hz, 1H), 8.29 (s, 1H), 8.20 – 8.15 (m, 1H), 8.04 (s, 1H), 7.89 (dd, J = 8.3, 1.7 Hz, 1H), 7.81 (m, 1H), 7.70 – 7.62 (m, 2H), 4.30 (d, J = 2.8 Hz, 2H), 3.95 – 3.85 (m, 2H), 3.71 – 3.58 (m, 2H), 1.96 – 1.68 (m, 6H), 1.59 – 1.46 (m, 2H), 1.45 – 1.37 (m, 1H), 1.30 – 0.90 (m, 6H). ^{13}C-NMR (101 MHz, $CDCl_3$): δ 185.75, 167.19, 149.53, 138.37, 130.86, 128.74, 128.16, 128.07, 127.80, 120.45, 77.22, 53.03, 42.81, 40.61, 38.34, 38.03, 30.39, 29.42, 26.94, 26.42. HRMS (ESI-MS): m/z [M+H]$^+$ calculated for $C_{24}H_{30}N_5O^+$: 404.2445, found: 404.2450. $C_{24}H_{29}N_5O$ × $C_4H_2F_6O_4$ (631.57).

(4-((1*H*-1,2,4-Triazol-1-yl)methyl)-4-cyclohexylpiperidin-1-yl)(isoquinolin-1-yl) methanone dihydrotrifluoroacetate (97)

The title compound was synthesized with **91** (48 mg, 0,10 mmol), isoquinoline-1-carboxylic acid (35 mg, 0.20 mmol), DIPEA (53 µL, 0,30 mmol) and HATU (42 mg, 0.11 mmol) according to the general procedure, yielding an orange solid (7 mg, 10 %). RP-HPLC: 99 %, (t_R = 10.52 min, *k* = 5.97). ^{1}H-NMR (500 MHz, $CDCl_3$): δ 8.56 (d, J = 5.9 Hz, 1H), 8.25 (s, 1H), 8.03 – 8.00 (m, 2H), 7.96 (d, J = 8.3 Hz, 1H), 7.86 (m, 2H), 7.73 (m, 1H), 4.32 – 4.23 (m, 2H), 4.05 – 3.92 (m, 2H), 3.24 (t, J = 5.9 Hz, 2H), 1.95 (m, 1H), 1.88 – 1.75 (m, 3H), 1.73 – 1.64 (m, 2H), 1.59 (m, 1H), 1.47 (m, 1H), 1.35 – 0.86 (m, 7H). ^{13}C-NMR (126 MHz, $CDCl_3$): δ 164.48, 153.87, 138.45, 137.35, 132.77, 129.38, 127.39, 126.37, 125.20, 122.79, 77.22, 52.71, 42.63, 40.56, 38.45, 37.53, 30.29, 29.42, 26.91, 26.33. HRMS (ESI-MS): m/z [M+H]$^+$ calculated for $C_{24}H_{30}N_5O^+$: 404.2445, found: 404.2450. $C_{24}H_{29}N_5O$ × $C_4H_2F_6O_4$ (631.57).

(4-((1*H*-1,2,4-Triazol-1-yl)methyl)-4-cyclohexylpiperidin-1-yl)(quinolin-3-yl)methanone dihydrotrifluoroacetate (98)

The title compound was synthesized with **91** (48 mg, 0,10 mmol), quinoline-3-carboxylic acid (35 mg, 0.20 mmol), DIPEA (53 µL, 0,30 mmol) and HATU (42 mg, 0.11 mmol) according to the general procedure, yielding a white solid (18 mg, 29 %). RP-HPLC: 99 %, (t_R = 10.22 min, *k* = 5.77). ^{1}H-NMR (500 MHz, $CDCl_3$): δ 9.20 (d, J = 2.0 Hz, 1H), 8.68 (d, J = 2.4 Hz, 1H), 8.40 – 8.35

(m, 2H), 8.09 – 8.05 (m, 2H), 8.00 (m, 1H), 7.82 (m, 1H), 4.30 (s, 2H), 3.86 (d, J = 33.9 Hz, 2H), 3.65 – 3.53 (m, 2H), 1.93 – 1.69 (m, 7H), 1.57 – 1.48 (m, 2H), 1.29 – 0.96 (m, 6H). ^{13}C-NMR (126 MHz, $CDCl_3$): δ 165.41, 149.35, 145.02, 143.82, 141.09, 134.04, 129.69, 129.49, 128.92, 127.95, 124.35, 77.23, 53.03, 43.78, 40.42, 38.26, 30.46, 29.19, 26.92, 26.40. HRMS (ESI-MS): m/z $[M+H]^+$ calculated for $C_{24}H_{30}N_5O^+$: 404.2445, found: 404.2451. $C_{24}H_{29}N_5O \times C_4H_2F_6O_4$ (631.57).

(4-((1*H*-1,2,4-Triazol-1-yl)methyl)-4-cyclohexylpiperidin-1-yl)(quinolin-4-yl)methanone dihydrotrifluoroacetate (99)

The title compound was synthesized with **91** (48 mg, 0,10 mmol), quinoline-4-carboxylic acid (35 mg, 0.20 mmol), DIPEA (53 µL, 0,30 mmol) and HATU (42 mg, 0.11 mmol) according to the general procedure, yielding a yellow solid (33 mg, 51 %). RP-HPLC: 99 %, (t_R = 9.47 min, k = 5.27). ^{1}H-NMR (500 MHz, $CDCl_3$): δ 9.21 (d, J = 5.2 Hz, 1H), 8.43 (d, J = 8.6 Hz, 1H), 8.28 (s, 1H), 8.06 – 7.97 (m, 2H), 7.84 (q, J = 10.2, 9.1 Hz, 2H), 7.72 – 7.65 (m, 1H), 4.33 – 4.20 (m, 2H), 4.09 – 4.01 (m, 1H), 3.87 (dd, J = 9.4, 4.2 Hz, 1H), 3.31 – 3.11 (m, 2H), 1.98 – 1.46 (m, 9H), 1.35 – 0.90 (m, 6H). ^{13}C-NMR (126 MHz, $CDCl_3$): δ 164.37, 149.77, 148.91, 145.89, 143.97, 134.21, 130.33, 125.40, 123.92, 118.22, 77.24, 52.72, 42.81, 40.12, 38.44, 37.53, 30.45, 29.53, 26.89, 26.24. HRMS (ESI-MS): m/z $[M+H]^+$ calculated for $C_{24}H_{30}N_5O^+$: 404.2445, found: 404.2450. $C_{24}H_{29}N_5O \times C_4H_2F_6O_4$ (631.57).

(4-((1*H*-1,2,4-Triazol-1-yl)methyl)-4-cyclohexylpiperidin-1-yl)(benzo[d][1,3]dioxol-5-yl)methanone hydrotrifluoroacetate (100)

The title compound was synthesized with **91** (48 mg, 0,10 mmol), benzo[d][1,3]dioxole-5-carboxylic acid (34 mg, 0.20 mmol), DIPEA (53 µL, 0,30 mmol) and HATU (42 mg, 0.11 mmol) according to the general procedure, yielding a white solid (9 mg, 18 %). RP-HPLC: 96 %, (t_R = 10.81 min, k = 6.16). ^{1}H-NMR (500 MHz, $CDCl_3$): δ 8.22 (s, 1H), 8.02 (s, 1H), 6.90 – 6.85 (m, 2H), 6.81 (d, J = 7.9 Hz, 1H), 6.00 (s, 2H), 4.27 (s, 2H), 3.60 (s, 4H), 1.88 – 1.68 (m, 7H), 1.42 (m, 2H), 1.26 – 1.07 (m, 4H), 1.04 – 0.93 (m, 2H). ^{13}C-NMR (126 MHz, $CDCl_3$): δ 170.44, 150.02, 149.01, 147.67, 143.84, 128.85, 121.52, 108.29, 107.88, 101.50, 77.23, 52.66, 40.81, 38.35, 29.85, 26.96, 26.41, 26.35. HRMS (ESI-MS): m/z $[M+H]^+$ calculated for $C_{22}H_{29}N_4O_3^+$: 397.2234, found: 397.2239. $C_{22}H_{28}N_4O_3 \times C_2HF_3O_2$ (510.51).

(4-((1*H*-1,2,4-Triazol-1-yl)methyl)-4-cyclohexylpiperidin-1-yl)(2,3dihydrobenzo[b][1,4] dioxin-6-yl)methanone hydrotrifluoroacetate (101)

The title compound was synthesized with **91** (48 mg, 0,10 mmol), 2,3-dihydrobenzo[b][1,4]dioxine-6-carboxylic acid (36 mg, 0.20 mmol), DIPEA (53 µL, 0,30 mmol) and HATU (42 mg, 0.11 mmol) according to the general procedure, yielding a white solid (7 mg, 13 %). RP-HPLC: 95 %, (t_R = 10.83 min, k = 6.17). ^{1}H-NMR (500 MHz, $CDCl_3$): δ 8.22 (s, 1H), 8.02 (s, 1H), 6.92 – 6.90 (m, 1H), 6.88 – 6.86 (m, 2H), 4.30 – 4.25 (m, 6H), 3.57 (s, 4H), 1.88 – 1.68 (m, 7H), 1.42 (m, 2H), 1.26 – 1.09 (m, 4H), 1.03 – 0.92 (m, 2H). ^{13}C-NMR (126 MHz, $CDCl_3$): δ 170.51, 149.93, 145.14, 143.51, 143.38, 128.19, 120.61, 117.35, 116.59, 77.23, 64.47, 64.30, 52.67, 40.84, 38.35, 29.33, 26.95, 26.41, 26.35. HRMS (ESI-MS): m/z $[M+H]^+$ calculated for $C_{23}H_{31}N_4O_3^+$: 411.2391, found: 411.2397. $C_{23}H_{30}N_4O_3 \times C_2HF_3O_2$ (524.54).

(4-((1*H*-Imidazol-1-yl)methyl)-4-cyclohexylpiperidin-1-yl)(2,3-dihydrobenzo[b][1,4]dioxin-2-yl)methanone hydrotrifluoroacetate (102)

The title compound was synthesized with **92** (31 mg, 0,07 mmol), 2,3-dihydrobenzo[b][1,4]dioxine-2-carboxylic acid (25 mg, 0.14 mmol), DIPEA (37 µL, 0,21 mmol) and HATU (29 mg, 0.08 mmol) according to the general procedure, yielding a white solid (14 mg, 41 %). RP-HPLC: 97 %, (t_R = 7.21 min, k = 3.77). ^{1}H-NMR (500 MHz, $CDCl_3$): δ 8.70 (d, J = 11.3 Hz, 1H), 7.38 (s, 1H), 7.01 (s, 1H), 6.91 – 6.80 (m, 4H), 4.79 (dd, J = 7.7, 2.6 Hz, 1H), 4.42 (m, 1H), 4.32 – 4.26 (m, 1H), 4.23 – 4.08 (m, 2H), 4.01 (d, J = 13.7 Hz, 1H), 3.92 – 3.85 (m, 1H), 3.77 – 3.61 (m, 1H), 3.50 – 3.38 (m, 1H), 1.97 – 1.60 (m, 8H), 1.33 – 0.97 (m, 7H). ^{13}C-NMR (126 MHz, $CDCl_3$): δ 143.31, 142.42, 136.75, 122.32, 121.72, 121.49, 121.02, 117.47, 117.18, 77.23, 70.53, 65.07, 53.21, 38.10, 37.20, 30.01, 29.25, 26.92, 26.33, 26.20. HRMS (ESI-MS): m/z $[M+H]^+$ calculated for $C_{24}H_{32}N_3O_3^+$: 410.2438, found: 410.2439. $C_{24}H_{31}N_3O_3 \times C_2HF_3O_2$ (523.55).

(4-((1*H*-Imidazol-1-yl)methyl)-4-cyclohexylpiperidin-1-yl)(chroman-3-yl)methanone hydrotrifluoroacetate (103)

The title compound was synthesized with **92** (31 mg, 0,07 mmol), chromane-3-carboxylic acid (26 mg, 0.14 mmol), DIPEA (37 µL, 0,21 mmol) and HATU (29 mg, 0.08 mmol) according to the general procedure, yielding a white solid (3 mg, 8 %) RP-HPLC: 97 %, (t_R = 7.34 min, k = 3.86). ^{1}H-NMR (500 MHz, $CDCl_3$), δ 8.68 (s, 1H), 7.40 (s, 1H), 7.08 (td, J = 14.6, 13.3, 7.4 Hz, 2H), 7.01 (s, 1H), 6.90 – 6.79 (m, 2H), 4.33 – 4.28 (m, 1H), 4.20 – 4.10 (m, 2H), 4.05 – 3.94 (m, 2H), 3.69 (d, J = 13.0 Hz, 1H), 3.52 (t, J = 10.5 Hz, 1H), 3.35 (t, 1H), 3.14 – 3.05 (m, 2H), 2.78 (t, J = 12.1 Hz, 1H), 1.94 – 1.72 (m, 6H), 1.66 (t, J = 12.1 Hz, 1H), 1.35 – 0.98 (m, 8H). ^{13}C-NMR (126 MHz, $CDCl_3$) δ 136.81, 129.83, 127.29, 122.24, 121.25, 116.69, 77.23, 70.30, 67.26, 53.12, 40.54, 38.93, 38.06, 36.64, 35.82, 30.00, 29.24, 28.98, 26.90, 26.36, 26.20. HRMS (ESI-MS) m/z $[M+H]^+$ calculated for $C_{25}H_{34}N_3O_2^+$: 408.2646, found: 408.2650. $C_{25}H_{33}N_3O_2 \times C_2HF_3O_2$ (521.58).

(4-((1*H*-Imidazol-1-yl)methyl)-4-cyclohexylpiperidin-1-yl)(naphthalen-2-yl)methanone hydrotrifluoroacetate (104)

The title compound was synthesized with **92** (31 mg, 0,07 mmol), 2-naphthoic acid (26 mg, 0.14 mmol), DIPEA (37 µL, 0,21 mmol) and HATU (29 mg, 0.08 mmol) according to the general procedure, yielding a colorless oil (5 mg, 15 %). RP-HPLC: 99 %, (t_R = 7.58 min, k = 4.02). ^{1}H-NMR (500 MHz, $CDCl_3$): δ 8.62 (s, 1H), 7.87 (s, 1H), 7.86 – 7.84 (m, 3H), 7.53 (m, 2H), 7.43 – 7.39 (m, 2H), 7.01 (s, 1H), 4.14 (s, 2H), 3.54 (s, 4H), 1.95 – 1.83 (m, 3H), 1.80 – 1.63 (m, 5H), 1.24 (dd, J = 14.4, 10.5 Hz, 3H), 1.14 (m, 2H), 1.07 – 0.97 (m, 2H). ^{13}C-NMR (126 MHz, $CDCl_3$): δ 170.80, 136.62, 133.76, 132.68, 132.62, 128.49, 128.46, 127.83, 127.27, 126.89, 126.86, 124.00, 122.37, 121.33, 77.23, 53.37, 38.97, 38.20, 26.91, 26.37, 26.21. HRMS (ESI-MS): m/z [M+H]$^+$ calculated for $C_{26}H_{32}N_3O^+$: 402.2540, found: 402.2544. $C_{26}H_{31}N_3O$ × $C_2HF_3O_2$ (515.57).

(4-((1*H*-Imidazol-1-yl)methyl)-4-cyclohexylpiperidin-1-yl)(quinolin-2-yl)methanone dihydrotrifluoroacetate (105)

The title compound was synthesized with **92** (31 mg, 0,07 mmol), quinoline-2-carboxylic acid (26 mg, 0.14 mmol), DIPEA (37 µL, 0,21 mmol) and HATU (29 mg, 0.08 mmol) according to the general procedure, yielding a orange solid (16.2 mg, 39 %). RP-HPLC: 96 %, (t_R = 6.78 min, k = 3.49). ^{1}H-NMR (500 MHz, $CDCl_3$): δ 8.71 (d, J = 1.4 Hz, 1H), 8.31 (d, J = 8.6 Hz, 1H), 8.11 (d, J = 8.5 Hz, 1H), 7.87 (dd, J = 8.2, 1.5 Hz, 1H), 7.79 (m, 1H), 7.69 – 7.61 (m, 2H), 7.41 (t, J = 1.6 Hz, 1H), 7.06 (d, J = 1.7 Hz, 1H), 4.17 (d, J = 5.5 Hz, 2H), 3.70 (m, 1H), 3.54 (m, 2H), 1.99 – 1.68 (m, 8H), 1.40 – 0.98 (m, 8H). ^{13}C-NMR (126 MHz, $CDCl_3$): δ 167.34, 152.98, 145.83, 138.30, 136.58, 130.79, 128.85, 128.05, 127.82, 122.69, 120.69, 120.52, 77.23, 53.78, 42.30, 38.80, 37.34, 29.86, 29.06, 26.90, 26.31. HRMS (ESI-MS): m/z [M+H]$^+$ calculated for $C_{25}H_{31}N_4O^+$: 403.2492, found: 403.2494. $C_{25}H_{30}N_4O$ × $C_4H_2F_6O_4$ (630.58).

(4-((1*H*-Imidazol-1-yl)methyl)-4-cyclohexylpiperidin-1-yl)(isoquinolin-1-yl)methanone dihydrotrifluoroacetate (106)

The title compound was synthesized with **92** (29 mg, 0,06 mmol), isoquinoline-1-carboxylic acid (22 mg, 0.12 mmol), DIPEA (32 µL, 0,18 mmol) and HATU (27 mg, 0.07 mmol) according to the general procedure, yielding a yellow solid (21 mg, 55 %). RP-HPLC: 96 %, (t_R = 6.52 min, k = 3.32). ^{1}H-NMR (400 MHz, $CDCl_3$): δ 8.69 (s, 1H), 8.51 (d, J = 6.1 Hz, 1H), 8.02 – 7.84 (m, 4H), 7.73 (m, 1H), 7.41 – 7.36 (m, 1H), 7.13 – 7.09 (m, 1H), 4.19 (s, 2H), 3.69 – 3.59 (m, 1H), 3.28 – 3.11 (m, 2H), 2.02 – 1.93 (m, 1H), 1.88 – 1.58 (m, 7H), 1.48 – 1.35 (m, 1H), 1.30 – 0.92 (m, 7H). ^{13}C-NMR (101 MHz, $CDCl_3$): δ 164.04, 153.22, 137.54, 136.41, 133.35, 129.85, 127.48, 126.36, 123.27, 122.87, 120.50, 77.24, 53.35, 42.14, 38.96, 37.10, 29.57, 28.82, 26.76, 26.23, 26.15. HRMS (ESI-MS): m/z [M+H]$^+$ calculated for $C_{25}H_{31}N_4O^+$: 403.2492, found: 403.2498. $C_{25}H_{30}N_4O$ × $C_4H_2F_6O_4$ (630.58).

(4-((1*H*-Imidazol-1-yl)methyl)-4-cyclohexylpiperidin-1-yl)(quinolin-3-yl)methanone dihydrotrifluoroacetat (107)

The title compound was synthesized with **92** (29 mg, 0,06 mmol), quinoline-3-carboxylic acid (21 mg, 0.12 mmol), DIPEA (32 µL, 0,18 mmol) and HATU (27 mg, 0.07 mmol) according to the general procedure, yielding a yellow solid (3 mg, 7 %). RP-HPLC: 99 %, (t_R = 6.33 min, k = 3.19). ^{1}H-NMR (500 MHz, $CDCl_3$): δ 9.12 (d, J = 2.1 Hz, 1H), 8.75 (s, 1H), 8.53 (d, J = 2.1 Hz, 1H), 8.28 (d, J = 8.6 Hz, 1H), 8.03 – 7.99 (m, 1H), 7.93 (m, 1H), 7.75 (m, 1H), 7.42 – 7.40 (m, 1H), 7.11 – 7.08 (m, 1H), 4.16 (s, 2H), 3.50 (t, J = 10.9 Hz, 2H), 1.97 – 1.71 (m, 8H), 1.35 – 1.01 (m, 9H). ^{13}C-NMR (126 MHz, $CDCl_3$): δ 166.44, 146.02, 144.39, 139.28, 136.84, 132.95, 128.96, 128.78, 127.69, 126.15, 122.60, 121.17, 77.22, 53.68, 43.23, 38.70, 38.17, 30.27, 26.90, 26.39, 26.18 . HRMS (ESI-MS): m/z [M+H]$^+$ calculated for $C_{25}H_{31}N_4O^+$: 403.2492, found: 403.2498. $C_{25}H_{30}N_4O$ × $C_4H_2F_6O_4$ (630.58).

(4-((1*H*-Imidazol-1-yl)methyl)-4-cyclohexylpiperidin-1-yl)(quinolin-4-yl)methanone dihydrotrifluoroacetate (108)

The title compound was synthesized with **92** (29 mg, 0,06 mmol), quinoline-4-carboxylic acid (21 mg, 0.12 mmol), DIPEA (32 µL, 0,18 mmol) and HATU (27 mg, 0.07 mmol) according to the general procedure, yielding a yellow solid (14 mg, 35 %). RP-HPLC: 95 %, (t_R = 5.95 min, k = 2.94). ^{1}H-NMR (400 MHz, $CDCl_3$): δ 9.17 (s, 1H), 8.89 (d, J = 20.1 Hz, 1H), 8.39 (t, J = 11.0 Hz, 1H), 8.03 – 7.56 (m, 4H), 7.43 (s, 1H), 7.08 (d, J = 14.1 Hz, 1H), 4.22 (s, 2H), 3.70 – 3.52 (m, 1H), 3.20 (d, J = 15.7 Hz, 2H), 2.03 – 1.60 (m, 8H), 1.42 – 0.91 (m, 8H). ^{13}C-NMR (126 MHz, $CDCl_3$): δ 164.82, 147.92, 146.38, 136.90, 133.61, 130.02, 125.37, 124.96, 118.64, 77.23, 53.24, 42.27, 38.81, 38.25, 36.97, 30.14, 29.78, 29.10, 26.82, 26.31, 2 6.12. HRMS (ESI-MS): m/z [M+H]$^+$ calculated for $C_{25}H_{31}N_4O^+$: 403.2492, found: 403.2499. $C_{25}H_{30}N_4O$ × $C_4H_2F_6O_4$ (630.58).

(4-((1*H*-Imidazol-1-yl)methyl)-4-cyclohexylpiperidin-1-yl)(benzo[d][1,3]dioxol-5-yl)methanon hydrotrifluoroacetate (109)

The title compound was synthesized with **92** (35 mg, 0,07 mmol), benzo[d][1,3]dioxole-5-carboxylic acid (24 mg, 0.15 mmol), DIPEA (39 µL, 0,22 mmol) and HATU (30 mg, 0.08 mmol) according to the general procedure, yielding a white solid (18 mg, 48 %). RP-HPLC: 99 %, (t_R = 6.49 min, k = 3.30). ^{1}H-NMR (500 MHz, $CDCl_3$): δ 8.73 (s, 1H), 7.40 (s, 1H), 7.04 (s, 1H), 6.87 – 6.78 (m, 3H), 5.99 (s, 2H), 4.17 (s, 2H), 3.49 – 3.41 (m, 2H), 1.92 – 1.60 (m, 9H), 1.30 – 0.96 (m, 8H). ^{13}C-NMR (126 MHz, $CDCl_3$): δ 170.36, 149.08, 147.71, 136.73, 128.74, 122.52, 121.53, 120.86, 108.28, 107.91, 101.51, 77.23, 53.47, 39.02, 38.14, 29.68,

26.91, 26.33, 26.20. HRMS (ESI-MS): m/z [M+H]$^+$ calculated for $C_{23}H_{30}N_3O_3^+$: 396.2282, found: 396.2287. $C_{23}H_{29}N_3O_3 \times C_2HF_3O_2$ (509.52).

(4-((1*H*-Imidazol-1-yl)methyl)-4-cyclohexylpiperidin-1-yl)(2,3-dihydrobenzo[b] [1,4] dioxin-6-yl)methanone hydrotrifluoroacetate (110)

The title compound was synthesized with **92** (35 mg, 0,07 mmol), 2,3-dihydrobenzo[b][1,4]dioxine-6-carboxylic acid (27 mg, 0.15 mmol), DIPEA (39 µL, 0,22 mmol) and HATU (30 mg, 0.08 mmol) according to the general procedure**,** yielding a white solid (22.0 mg, 57 %). RP-HPLC: 99 %, (t_R = 6.55 min, *k* = 3.34). ^{1}H-NMR (500 MHz, $CDCl_3$): δ 8.74 (s, 1H), 7.40 (s, 1H), 7.04 (s, 1H), 6.89 – 6.81 (m, 3H), 4.29 – 4.24 (m, 4H), 4.17 (s, 2H), 3.50 – 3.41 (m, 2H), 1.91 – 1.60 (m, 9H), 1.30 – 0.96 (m, 8H). ^{13}C-NMR (126 MHz, $CDCl_3$): δ 170.40, 145.21, 143.42, 136.70, 128.11, 122.56, 120.78, 120.56, 117.36, 116.60, 77.23, 64.46, 64.28, 53.46, 39.03, 38.13, 29.64, 26.91, 26.31, 26.20. HRMS (ESI-MS): m/z [M+H]$^+$ calculated for $C_{24}H_{32}N_3O_3^+$: 410.2438, found: 410.2443. $C_{24}H_{31}N_3O_3 \times C_2HF_3O_2$ (523.55).

2-Chloro-*N*-(*p*-tolyl)acetamide (112)

4-Methylaniline (428 mg, 4 mmol, 1 eq) was dissolved in DCM and cooled down in a 0 °C ice bath. Afterwards, TEA (1.1 ml, 8 mmol, 2 eq) was added first, followed by chloracetyl chloride (**111**, 478 µl, 6 mmol, 1.5 eq). The solution was allowed to warm to rt and stirred for 4-6 h. To quench the reaction, 1 ml of H_2O was added. After further purification, the product was obtained as a white solid (617 mg, 84 %).^{1}H NMR (400 MHz, $CDCl_3$) δ 8.12 (s, 1H), 7.38 – 7.31 (m, 2H), 7.12 – 7.04 (m, 2H), 4.10 (s, 2H), 2.26 (s, 3H).^{13}C NMR (101 MHz, $CDCl_3$): δ 163.77, 135.01, 134.14, 129.64, 120.26, 77.27, 42.91, 20.93. HRMS (ESI-MS): m/z [M+H$^+$] calculated for $C_9H_{11}ClNO$: 184.0529, found: 184.0525. $C_9H_{10}ClNO$ (183.64).

***tert*-Butyl 4-(2-oxo-2-(*p*-tolylamino)ethyl)piperazine-1-carboxylate (113)**

tert-Butyl-piperazine-1-carboxylate (761 mg, 4.1 mmol, 3 eq) in 3 mL acetonitrile was added dropwise to a solution of **112** (250 mg, 1.3 mmol, 1 eq) in 2 mL acetonitrile under stirring. Potassium carbonate (900 mg, 16.4 mmol, 5 eq) was then added to the mixture. The solution was refluxed for 5 hours at 80 °C. After completion, the reaction mixture was cooled to room temperature. After filtration, the product was purified using flash column chromatography and was obtained as a white solid (390 mg, 90 %). ^{1}H NMR (400 MHz, $CDCl_3$): δ 8.94 (s, 1H), 7.47 – 7.40 (m, 2H), 7.18 – 7.10 (m, 2H), 3.50 (t, *J* = 5.1 Hz, 4H), 3.14 (s, 2H), 2.57 (t, *J* = 5.1 Hz, 4H), 2.32 (s, 3H), 1.47 (s, 9H).^{13}C NMR (101 MHz, $CDCl_3$): δ 167.72, 154.64, 134.91, 134.00, 129.57, 119.51, 80.07, 77.23, 62.11, 53.29, 28.41, 20.89. HRMS (ESI-MS): m/z [M+H$^+$] calculated for $C_{18}H_{28}N_3O_3$: 334.2125, found: 334.2131. $C_{18}H_{27}N_3O_3$ (333.43).

2-(Piperazin-1-yl)-*N*-(*p*-tolyl)acetamide (114)

The vessel was purged with argon and **113** (300 mg, 0.9 mmol, 1 eq) was dissolved in 4 mL TFA in DCM (50 %). The solution was stirred at room temperature for 3 hours. The product was then purified using flash column chromatography. The product was obtained as a beige solid (199 mg, 95 %).^{1}H NMR (400 MHz, DMSO-*d$_6$*): δ 11.01 (s, 1H), 10.13 (s, 2H), 7.79 – 7.71 (m, 2H), 7.41 – 7.35 (m, 2H), 4.40 (s, 2H), 3.78 (m, 4H), 3.65 (m, 4H), 2.49 (s, 3H).^{13}C NMR (101 MHz, $CDCl_3$): δ 168.17, 135.01, 133.86, 129.52, 119.51, 62.60, 54.52, 46.13, 29.70, 20.88. HRMS (ESI-MS): m/z [M+H$^+$] calculated for $C_{13}H_{20}N_3O^+$: 234.1601, found: 234.1606. $C_{13}H_{19}N_3O$ x $C_2HF_3O_2$ (347.34).

General procedure for the synthesis of ***115-124***

HATU (3.1 - 4.3 mmol, 1.2 - 1.5 eq) and DIPEA (5.7 – 16.5 mmol, 2-3 eq) were added to an ice-cold (-15°C) stirring mixture of the respective 3-phenylpropanoic acid derivative (2.5 – 5.5 mmol, 1 eq) in DCM (5 mL). **114** (2.8 – 6.0 mmol, 1.1-1.6 eq) was dissolved in a minimum amount of DCM with TEA (2.6 - 16.5 mmol, 2 eq) and was added to the reaction mixture. The reaction was stirred for about 12 hours. To quench the reaction, a water-HCl mixture (0.05 N) was added. Afterward, the organic layer was washed with Na_2CO_3 and separated from the aqueous layer. The organic layer was then dried over Na_2SO_4 and purified using preparative HPLC. The desired products **115-124** were obtained as white solids.

2-(4-(3-(4-Chlorophenyl)propanoyl)piperazin-1-yl)-*N*-(*p*-tolyl)acetamide hydrotrifluoroacetate (115)

The title compound was synthesized according to the general procedure with HATU (251 mg, 6.6 mmol, 1.2 eq), DIPEA (213 mg, 16.5 mmol, 3 eq), 3-(4-chlorophenyl)propanoic acid (305 mg, 5.5 mmol, 1 eq)**, 114** (210 mg, 6.1 mmol, 1.1 eq), TEA (99 mg, 9.7 mmol, 2eq) yielding a fluffy white solid (164 mg, 58 %). RP-HPLC: 99 %, (t_R = 8.62, *k* = 1.97) ^{1}H NMR (500 MHz, $CDCl_3$): δ 9.79 (s, 2H), 7.42 (s, 2H), 7.29 (s, 1H), 7.28 (s, 1H), 7.17 – 7.11 (m, 4H), 3.82 (m, 4H), 3.63 (t, *J* = 5.2 Hz, 2H), 3.10 (m, 4H), 2.95 (t, *J* = 7.6 Hz, 2H), 2.59 (t, *J* = 7.6 Hz, 2H), 2.30 (s, 3H).^{13}C NMR (126 MHz, $CDCl_3$): δ 170.45, 162.08, 139.06, 135.02, 134.43, 132.27, 129.88, 129.63, 128.77, 120.04, 58.92, 52.39, 52.20, 42.76, 38.99, 34.31, 30.44, 20.88. HRMS (ESI-MS): m/z [M+H$^+$] calculated for $C_{22}H_{27}ClN_3O_2^+$: 400.1787 Da, found: 400.1790. $C_{22}H_{26}ClN_3O_2$ x $C_2HF_3O_2$ (513.94).

2-(4-(3-(3-Chlorophenyl)propanoyl)piperazin-1-yl)-*N*-(*p*-tolyl)acetamide hydrotrifluoroacetate (116)

The title compound was synthesized according to the general procedure with HATU (175 mg, 4.6 mmol, 1.2 eq), DIPEA (189 mg, 14.7 mmol, 3 eq), 3-(4-chlorophenyl)propanoic acid (90 mg, 4.9 mmol, 1 eq), **114** (187 mg, 5.4 mmol, 1.1 eq), TEA (99 mg, 9.7 mmol, 2 eq) yielding a fluffy white solid (170 mg, 68 %). RP-HPLC: 96 %, (t_R = 8.55, *k* = 1.95). ^{1}H NMR (500 MHz, $CDCl_3$): δ 9.77 (s, 1H), 7.43 (d, *J* = 8.2 Hz, 2H), 7.27 – 7.18 (m, 3H), 7.12 (dd, *J* = 10.4, 7.7 Hz, 3H), 3.88 (m, 2H), 3.73 (s, 2H), 3.61 (m, 2H), 3.14 – 3.08 (m, 2H), 2.97 (m, 4H), 2.61 (t, *J* = 7.5 Hz, 2H), 2.31 (s, 3H). ^{13}C NMR (126 MHz, $CDCl_3$): δ 170.36, 162.21, 142.70, 134.93, 134.50, 134.34, 130.04, 129.62, 128.63, 126.77, 126.73, 119.99, 59.10, 52.51, 52.21, 42.90, 39.08, 34.12, 30.89, 20.88. HRMS (ESI-MS): m/z [M+H$^+$] calculated for $C_{22}H_{27}ClN_3O_2^+$: 400.1787, found: 400.1786. $C_{22}H_{26}ClN_3O_2$ x $C_2HF_3O_2$ (513.94).

2-(4-(3-(2-Chlorophenyl)propanoyl)piperazin-1-yl)-*N*-(*p*-tolyl)acetamide hydrotrifluoroacetate (117)

The title compound was synthesized according to the general procedure with HATU (175 mg, 4.6 mmol, 1.2 eq), DIPEA (189 mg, 14.7 mmol, 3 eq), 3-(4-chlorophenyl)propanoic acid (90 mg, 4.9 mmol, 1 eq), **114** (145 mg, 5.3 mmol, 1.1 eq), TEA (99 mg, 9.7 mmol, 2 eq) yielding a fluffy white solid (181 mg, 73 %). RP-HPLC: 96 %, (t_R = 8.64, *k* = 1.98). ^{1}H NMR (500 MHz, $CDCl_3$): δ 9.74 (s, 1H), 7.43 (d, *J* = 8.1 Hz, 2H), 7.37 (dd, *J* = 7.3, 1.9 Hz, 1H), 7.27 (d, *J* = 2.2 Hz, 1H), 7.24 – 7.17 (m, 2H), 7.13 (d, *J* = 8.1 Hz, 2H), 3.86 – 3.82 (m, 2H), 3.70 – 3.54 (m, 4H), 3.10 (t, *J* = 7.6 Hz, 2H), 3.01 (d, *J* = 5.2 Hz, 2H), 2.89 – 2.85 (m, 2H), 2.65 (t, *J* = 7.7 Hz, 2H), 2.31 (s, 3H). ^{13}C NMR (126 MHz, $CDCl_3$): δ 170.62, 162.98, 138.09, 134.76, 134.60, 133.85, 131.11, 129.70, 129.61, 128.23, 127.17, 119.88, 59.65, 52.59, 52.43, 43.40, 39.47, 32.41, 29.71, 20.88. HRMS (ESI-MS): m/z [M+H$^+$] calculated for $C_{22}H_{27}ClN_3O_2^+$: 400.1787 Da, found: 400.1792. $C_{22}H_{26}ClN_3O_2$ x $C_2HF_3O_2$ (513.94).

2-(4-(3-(4-Bromophenyl)propanoyl)piperazin-1-yl)-*N*-(*p*-tolyl)acetamide hydrotrifluoroacetate (118)

The title compound was synthesized according to the general procedure with HATU (119 mg, 3.1 mmol, 1.2 eq), DIPEA (102 mg, 7.9 mmol, 3 eq), 3-(4-chlorophenyl)propanoic acid (60 mg, 2.6 mmol, 1 eq), **114** (100 mg, 2.9 mmol, 1.61 eq), TEA (53 mg, 5.2 mmol, 2 eq) yielding a fluffy white solid (108 mg, 76 %). RP-HPLC: 98 %, (t_R = 8.92, *k* = 2.07). ^{1}H NMR (500 MHz, $CDCl_3$): δ 7.46 – 7.38 (m, 4H), 7.10 (t, *J* = 8.9 Hz, 4H), 3.84 (m, 4H), 3.64 (m, 2H), 3.13 (m, 4H), 2.93 (t, *J* = 7.5 Hz, 2H), 2.58 (t, *J* = 7.6 Hz, 2H), 2.30 (s, 3H). ^{13}C NMR (126 MHz, $CDCl_3$): δ 170.42, 161.85, 139.57, 135.06, 134.40, 131.73, 130.29, 129.63, 120.27, 120.05, 58.80, 52.37, 52.16, 42.67, 38.90, 34.21, 30.50, 20.89. HRMS (ESI-MS): m/z [M+H$^+$] calculated for $C_{22}H_{27}BrN_3O_2^+$: 444.1282, found: 444.1289. $C_{22}H_{26}BrN_3O_2$ x $C_2HF_3O_2$ (558.40).

2-(4-(3-(3-Bromophenyl)propanoyl)piperazin-1-yl)-*N*-(*p*-tolyl)acetamide hydrotrifluoroacetate (119)

The title compound was synthesized according to the general procedure with HATU (175 mg, 4.6 mmol, 1.2 eq), DIPEA (189 mg, 14.7 mmol, 3 eq), 3-(4-chlorophenyl)propanoic acid (90 mg, 4.9 mmol, 1 eq), **114** (145 mg, 5.3 mmol, 1.1 eq), TEA (99 mg, 9.7 mmol, 2 eq) yielding a fluffy white solid (123 mg, 86 %). RP-HPLC: 99 %, (t_R = 8.96, *k* = 2.09). ^{1}H NMR (500 MHz, $CDCl_3$): δ 9.83 (s, 1H), 7.45 – 7.33 (m, 4H), 7.20 (t, *J* = 7.7 Hz, 1H), 7.17 – 7.08 (m, 3H), 3.82 (s, 4H), 3.62 (t, *J* = 5.3 Hz, 2H), 3.19 (s, 2H), 3.07 – 2.90 (m, 4H), 2.59 (t, *J* = 7.5 Hz, 2H), 2.30 (s, 3H). ^{13}C NMR (126 MHz, $CDCl_3$): δ 170.41, 161.70, 142.96, 135.06, 134.40, 131.55, 130.39, 129.70, 129.64, 127.25, 122.60, 120.06, 58.86, 52.49, 52.13, 42.67, 38.84, 34.08, 30.87, 20.88. HRMS (ESI-MS): m/z [M+H$^+$] calculated for $C_{22}H_{27}BrN_3O_2^+$: 444.1282, found: 444.1289. $C_{22}H_{26}BrN_3O_2$ x $C_2HF_3O_2$ (558.40).

2-(4-(3-(2-Bromophenyl)propanoyl)piperazin-1-yl)-*N*-(*p*-tolyl)acetamide hydrotrifluoroacetate (120)

The title compound was synthesized according to the general procedure with HATU (175 mg, 4.6 mmol, 1.2 eq), DIPEA (189 mg, 14.7 mmol, 3 eq), 3-(4-chlorophenyl)propanoic acid (90 mg, 4.9 mmol, 1 eq), **114** (145 mg, 5.3 mmol, 1.1 eq), TEA (99 mg, 9.7 mmol, 2 eq) yielding a fluffy white solid (123 mg, yielding 86 %). RP-HPLC: 98 %, (t_R = 8.71, *k* = 2.00). ^{1}H NMR (500 MHz, $CDCl_3$): δ 9.88 (s, 1H), 7.56 (d, *J* = 8.0 Hz, 1H), 7.41 (d, *J* = 8.2 Hz, 2H), 7.12 (dd, *J* = 8.1, 5.6 Hz, 3H), 3.79 (s, 4H), 3.64 (t, *J* = 5.2 Hz, 2H), 3.20 – 3.06 (m, 4H), 2.98 (m, 2H), 2.65 (t, *J* = 7.6 Hz, 2H), 2.30 (s, 3H). ^{13}C NMR (126 MHz, $CDCl_3$): δ 170.67, 161.60, 139.62, 135.04, 134.43, 133.06, 131.14, 129.64, 128.56, 127.86, 124.24, 120.01, 58.86, 52.45, 52.17, 42.77, 38.79, 32.37, 32.13, 20.88. HRMS (ESI-MS): m/z [M+H$^+$] calculated for $C_{22}H_{27}BrN_3O_2^+$: 444.1282, found: 444.1289. $C_{22}H_{26}BrN_3O_2$ x $C_2HF_3O_2$ (558.40).

2-(4-(3-(4-Fluorophenyl)propanoyl)piperazin-1-yl)-*N*-(*p*-tolyl)acetamide hydrotrifluoroacetate (121)

The title compound was synthesized according to the general procedure with HATU (163 mg, 4.3 mmol, 1.5 eq), DIPEA (74 mg, 5.7 mmol, 2 eq), 3-(4-chlorophenyl)propanoic acid (48 mg, 2.9 mmol, 1 eq), **114** (158 mg, 4.6 mmol, 1.6 eq), TEA (58 mg, 5.7 mmol, 2 eq) yielding a fluffy white solid (89 mg, 63 %). RP-HPLC: 97 %, (t_R = 7.99, *k* = 1.76). ^{1}H NMR (500 MHz, $CDCl_3$): δ 9.80 (s, 1H), 7.44 – 7.38 (m, 2H), 7.20 – 7.14 (m, 2H), 7.12 (d, *J* = 8.1 Hz, 2H), 7.04 – 6.97 (m, 2H), 3.82 (s, 4H), 3.64 (m, 2H), 3.25 – 3.04 (m, 4H), 2.95 (t, *J* = 7.5 Hz, 2H), 2.59 (t, *J* = 7.6 Hz, 2H), 2.30 (s, 3H). ^{13}C NMR (126 MHz, $CDCl_3$): δ 170.61, 162.56, 161.82, 160.61, 136.19 (d, *J* = 3.1 Hz) , 135.05, 134.40, 129.90 (d, J = 7.6 Hz), 129.63, 120.04, 115.55, 115.38, 77.29, 77.03,

76.78, 58.81, 52.28 (d, *J* = 28.9 Hz), 42.69, 38.90, 34.56, 30.35, 20.87. HRMS (ESI-MS): m/z [M+H+] calculated for $C_{22}H_{27}FN_3O_2^+$: 384.2082, found: 384.2088. $C_{22}H_{26}FN_3O_2$ x $C_2HF_3O_2$ (497.49).

2-(4-(3-(3-Fluorophenyl)propanoyl)piperazin-1-yl)-*N*-(*p*-tolyl)acetamide hydrotrifluoroacetate (122)

The title compound was synthesized according to the general procedure with HATU (163 mg, 4.3 mmol, 1.5 eq), DIPEA (74 mg, 5.7 mmol, 2 eq), 3-(4-chlorophenyl)propanoic acid (48 mg, 2.9 mmol, 1 eq), **114** (158 mg, 4.6 mmol, 1.6 eq), TEA (58 mg, 5.7 mmol, 2 eq) yielding a fluffy white solid (107 mg, 75 %). RP-HPLC: 99 %, (t_R = 8.23, *k* = 1.83). ^{1}H NMR (500 MHz, $CDCl_3$): δ 7.43 – 7.37 (m, 2H), 7.28 – 7.25 (m, 1H), 7.12 (d, *J* = 8.2 Hz, 2H), 6.99 (d, *J* = 7.6 Hz, 1H), 6.97 – 6.90 (m, 2H), 3.83 (m, 4H), 3.63 (t, *J* = 5.2 Hz, 2H), 3.14 (d, *J* = 56.8 Hz, 4H), 2.98 (t, *J* = 7.5 Hz, 2H), 2.60 (t, *J* = 7.6 Hz, 2H), 2.30 (s, 3H). ^{13}C NMR (126 MHz, $CDCl_3$) δ 170.46, 163.93, 162.51, 161.85 (d, *J* = 31.8 Hz), 143.10 (d, *J* = 7.2 Hz), 135.07, 134.40, 130.24, 130.20 (d, *J* = 8.50 Hz), 129.64, 124.14 (d, *J* = 2.8 Hz), 120.05, 115.37 (d, *J* = 21.4 Hz), 113.45 (d, *J* = 21.4 Hz), 58.79, 52.29 (d, *J* = 37.6 Hz), 42.65, 38.86, 34.11, 30.90, 20.86. HRMS (ESI-MS): m/z [M+H+] calculated for $C_{22}H_{27}FN_3O_2^+$: 384.2082, found: 384.2088. $C_{22}H_{26}FN_3O_2$ x $C_2HF_3O_2$ (497.49).

2-(4-(3-(2-Fluorophenyl)propanoyl)piperazin-1-yl)-*N*-(*p*-tolyl)acetamide hydrotrifluoroacetate (123)

The title compound was synthesized according to the general procedure with HATU (163 mg, 4.3 mmol, 1.5 eq), DIPEA (74 mg, 5.7 mmol, 2 eq), 3-(4-chlorophenyl)propanoic acid (48 mg, 2.9 mmol, 1 eq), **114** (158 mg, 4.6 mmol, 1.6 eq), TEA (58 mg, 5.7 mmol, 2 eq) yielding a fluffy white solid (98 mg, 69 %). RP-HPLC: 95 %, (t_R = 7.90, *k* = 1.72). ^{1}H NMR (500 MHz, $CDCl_3$): δ 9.86 (s, 1H), 8.06 (s, 2H), 7.45 – 7.38 (m, 2H), 7.15 – 7.02 (m, 4H), 3.82 (s, 4H), 3.64 (t, *J* = 5.2 Hz, 2H), 3.18 (s, 2H), 3.01 (t, *J* = 7.6 Hz, 2H), 2.63 (t, *J* = 7.6 Hz, 2H), 2.30 (s, 3H). ^{13}C NMR (126 MHz, $CDCl_3$) δ 170.68, 162.18 (d, *J* = 4.7 Hz), 161.6, 160.23, 135.04, 134.45, 131.11 (d, *J* = 5.0 Hz), 129.63, 128.52 (d, *J* = 8.5 Hz) , 127.19 (d, *J* = 15.5 Hz), 124.35 (d, *J* = 3.5 Hz), 120.01, 115.44 (d, *J* = 21.4 Hz), 58.84, 52.47, 52.20, 42.67, 38.80, 32.87, 25.30, 20.85. HRMS (ESI-MS): m/z [M+H+] calculated for $C_{22}H_{27}FN_3O_2^+$: 384.2082, found: 384.2088. $C_{22}H_{26}FN_3O_2$ x $C_2HF_3O_2$ (497.49).

2-(4-(3-(4-(Dimethylamino)phenyl)propanoyl)piperazin-1-yl)-*N*-(*p*-tolyl)acetamide hydrotrifluoroacetate (124)

The title compound was synthesized according to the general procedure with HATU (153 mg, 4.0 mmol, 1.3 eq), DIPEA (80 mg, 6.2 mmol, 2 eq), 3-(4-chlorophenyl)propanoic acid (60 mg, 3.1 mmol, 1 eq), **114** (90 mg, 5.0 mmol, 1.6 eq), TEA (62 mg, 6.2 mmol, 2 eq) yielding a fluffy white solid (94 mg, 58 %). RP-HPLC: 99 %, (t_R = 4.66, *k* = 0.61). ^{1}H NMR (500 MHz, DMSO-*d6*): δ 10.59 (s, 1H), 7.55 – 7.43 (m, 2H), 7.18 (dd, *J* = 8.2, 5.7 Hz, 4H), 6.95 (d, *J* = 8.0 Hz, 2H), 4.17 (s, 2H), 3.15 (d, *J* = 3.1 Hz, 2H), 2.95 (s, 6H), 2.76 (t, *J* = 7.7 Hz, 2H), 2.64 (dd, *J* = 8.8, 6.6 Hz, 2H), 2.27 (s, 3H). ^{13}C NMR (126 MHz, DMSO-*d6*): δ 170.76, 163.00, 135.79, 133.80, 129.85, 129.69, 119.96, 117.88, 57.29, 52.28, 52.15, 42.52, 42.07, 38.32, 34.43, 30.13, 20.93. HRMS (ESI-MS): m/z [M+H+] calculated for $C_{24}H_{33}N_4O_2^+$: 409.2599, found: 409.2603. $C_{24}H_{32}N_4O_2$ x $C_2HF_3O_2$ (522.57).

References

(1) Priyam, A.; Woodcroft, B. J.; Rai, V.; Moghul, I.; Munagala, A.; Ter, F.; Chowdhary, H.; Pieniak, I.; Maynard, L. J.; Gibbins, M. A.; Moon, H.; Davis-Richardson, A.; Uludag, M.; Watson-Haigh, N. S.; Challis, R.; Nakamura, H.; Favreau, E.; Gómez, E. A.; Pluskal, T.; Leonard, G.; Rumpf, W.; Wurm, Y. Sequenceserver: A Modern Graphical User Interface for Custom BLAST Databases. *Mol. Biol. Evol.* **2019**, *36* (12), 2922–2924. https://doi.org/10.1093/molbev/msz185.

(2) Webb, B.; Sali, A. Comparative Protein Structure Modeling Using MODELLER. *Curr. Protoc. Bioinformatics* **2016**, *54*, 5.6.1-5.6.37. https://doi.org/10.1002/CPBI.3.

(3) Hawkins, P.C.D.; Skillman, A.G.; Nicholls, A. Comparison of Shape-Matching and Docking as Virtual Screening Tools. *J. Med. Chem.* **2007**, *50* (1), 74–82. https://doi.org/10.1021/jm0603365

(4) Coleman, R. G.; Carchia, M.; Sterling, T.; Irwin, J. J.; Shoichet, B. K. Ligand Pose and Orientational Sampling in Molecular Docking. *PLoS One* **2013**, *8* (10), e75992. https://doi.org/10.1371/JOURNAL.PONE.0075992.

(5) Halgren, T. A. Merck Molecular Force Field. I. Basis, Form, Scope, Parameterization, and Performance of MMFF94. *J. Comput. Chem.* **1996**, *17* (5–6), 490–519. https://doi.org/https://doi.org/10.1002/(SICI)1096-987X(199604)17:5/6<490::AID-JCC1>3.0.CO;2-P.

(6) Passaro, S.; Corso, G.; Wohlwend, J.; Reveiz, M.; Thaler, S.; Somnath, V. A.; Getz, N.; Portnoi, T.; Roy, J.; Stark, H.; Kwabi-Addo, D.; Beaini, D.; Jaakkola, T.; Barzilay R. Boltz-2: Towards Accurate and Efficient Binding Affinity Prediction. *bioRxiv* **2025**. https://doi.org/10.1101/2025.06.14.659707.

(7) Klarenbeek, J.; Goedhart, J.; Van Batenburg, A.; Groenewald, D.; Jalink, K. Fourth-Generation Epac-Based FRET Sensors for CAMP Feature Exceptional Brightness, Photostability and Dynamic Range: Characterization of Dedicated Sensors for FLIM, for Ratiometry and with High Affinity. *PLoS One* **2015**. https://doi.org/10.1371/journal.pone.0122513.

(8) Jiang, L. I.; Collins, J.; Davis, R.; Lin, K. M.; DeCamp, D.; Roach, T.; Hsueh, R.; Rebres, R. A.; Ross, E. M.; Taussig, R.; Fraser, I.; Sternweis, P. C. Use of a CAMP BRET Sensor to Characterize a Novel Regulation of CAMP by the Sphingosine 1-Phosphate/G13 Pathway. *Journal of Biological Chemistry* **2007**. https://doi.org/10.1074/jbc.M609695200.

(9) Schihada, H.; Vandenabeele, S.; Zabel, U.; Frank, M.; Lohse, M. J.; Maiellaro, I. A Universal Bioluminescence Resonance Energy Transfer Sensor Design Enables High-Sensitivity Screening of GPCR Activation Dynamics. *Commun. Biol.* **2018**, *1* (1). https://doi.org/10.1038/s42003-018-0072-0.

(10) Schihada, H.; Shekhani, R.; Schulte, G. Quantitative Assessment of Constitutive G Protein–Coupled Receptor Activity with BRET-Based G Protein Biosensors. *Sci. Signal.* **2021**, *14* (699), 1653. https://doi.org/10.1126/scisignal.abf1653.

Figure S1: Amino acid sequence of GPR3-HaloTag/Nluc biosensor.

MYPYDVPDYAYPYDVPDYAYPYDVPDYADMWGAGSPLAWLSAGSGNVNVSSVGPAEGPTGPAAPLPSPKAWDVVLCISGTLVSCENA
LVVAIIVGTPAFRAPMFLLVGSLAVADLLAGLGLVLHFAAVFCIGSAEMSLVLVGVLAMAFTASIGSLLAITVDRYLSLYNALTYYSETTVT
RTYVMLALVWGGALGLGLLPVLAWNCLDGLTTCGVVYPLSKNHLVVLAIAFFMVFGIMLQLYAQICRIVCRHAQQIALQRASEIGTGFPF
DPHYVEVLGERMHYVDVGPRDGTPVLFLHGNPTSSYVWRNIIPHVAPTHRCIAPDLIGMGKSDKPDLGYFFDDHVRFMDAFIEALGLE
EVVLVIHDWGSALGFHWAKRNPERVKGIAFMEFIRPIPTWDEWPEFARETFQAFRTTDVGRKLIIDQNVFIEGTLPMGVVRPLTEVEM
DHYREPFLNPVDREPLWRFPNELPIAGEPANIVALVEEYMDWLHQSPVPKLLFWGTPGVLIPPAEAARLAKSLPNCKAVDIGPGLNLL
QEDNPDLIGSEIARWLSTLEISGGSHLLPASHYVATRKGIATLAVVLGAFAACWLPFTVYCLLGDAHSPPLYTYLTLLPATYNSMINPIIY
AFRNQDVQKVLWAVCCCCSSSKIPFRSRSPSDVSRVFTLEDFVGDWRQTAGYNLDQVLEQGGVSSLFQNLGVSVTPIQRIVLSGENGLKI
DIHVIIPYEGLSGDQMGQIEKIFKVVYPVDDHHFKVILHYGTLVIDGVTPNMIDYFGRPYEGIAVFDGKKITVTGTLWNGNKIIDERLINP
DGSLLFRVTINGVTGWRLCERILA*

GPR3, 3x HA-tag, HaloTag, Nluc

Figure S2: AF64394-induced BRET responses at GPR3-, α_{2A}AR- and β_2AR-HaloTag/Nluc sensors.

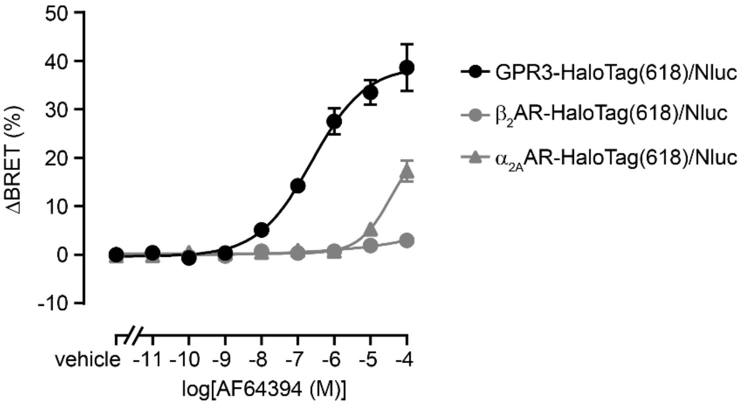

Concentration response curves of AF64394 obtained with the GPR3, β_2AR and α_{2A}AR conformational sensors. Data show mean ± SEM of three independent experiments conducted in HEK293 cells stably expressing the indicated sensors.

Figure S3: Raw data for the determination of the average Z' factor of the GPR3 biosensors.

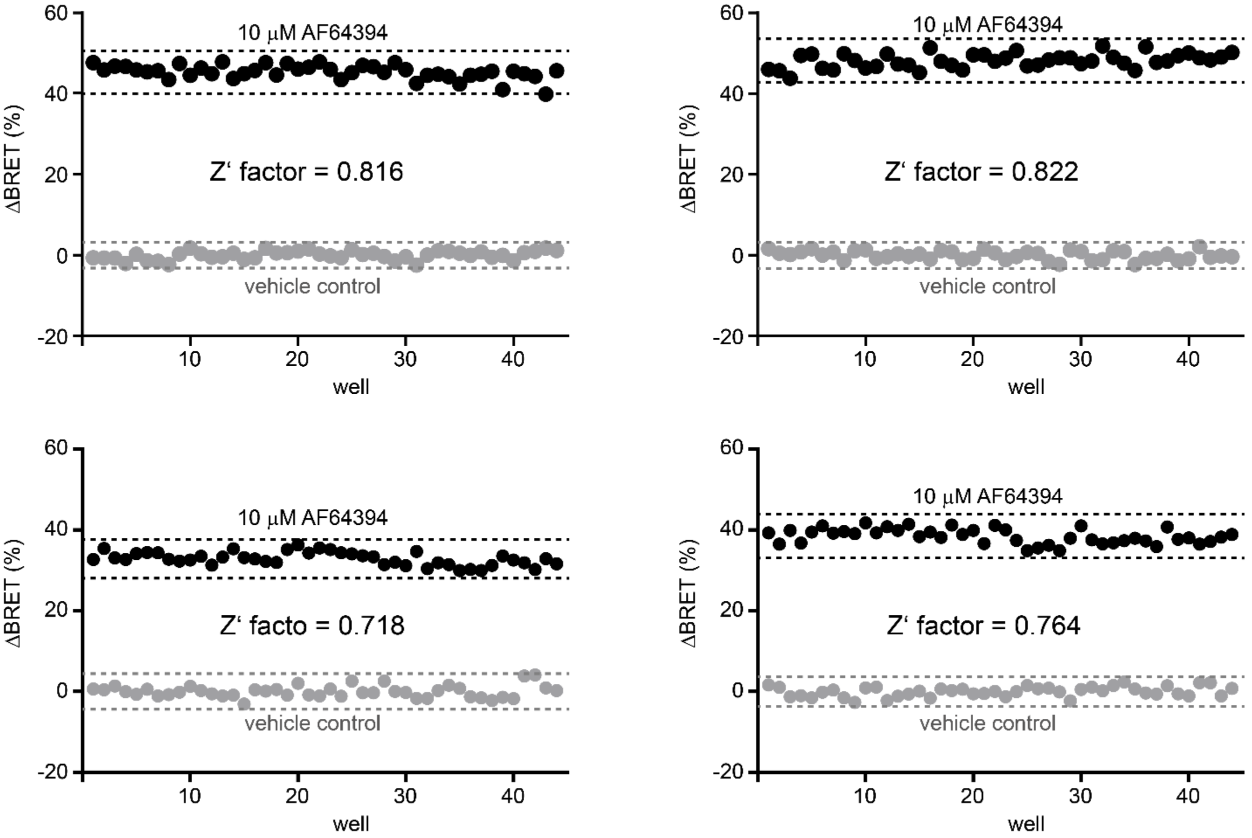

AF64394- and vehicle-induced BRET changes in four independent biological replicates used for the determination of the average Z' factor of the GPR3 conformational biosensor. Each datapoints represents the AF64394 - or vehicle induced BRET response in one technical replicate (well). Experiments were conducted in HEK293 cells stably expressing the GPR3 biosensor.

Figure S4: Homology models of GPR3 used for virtual compound docking.

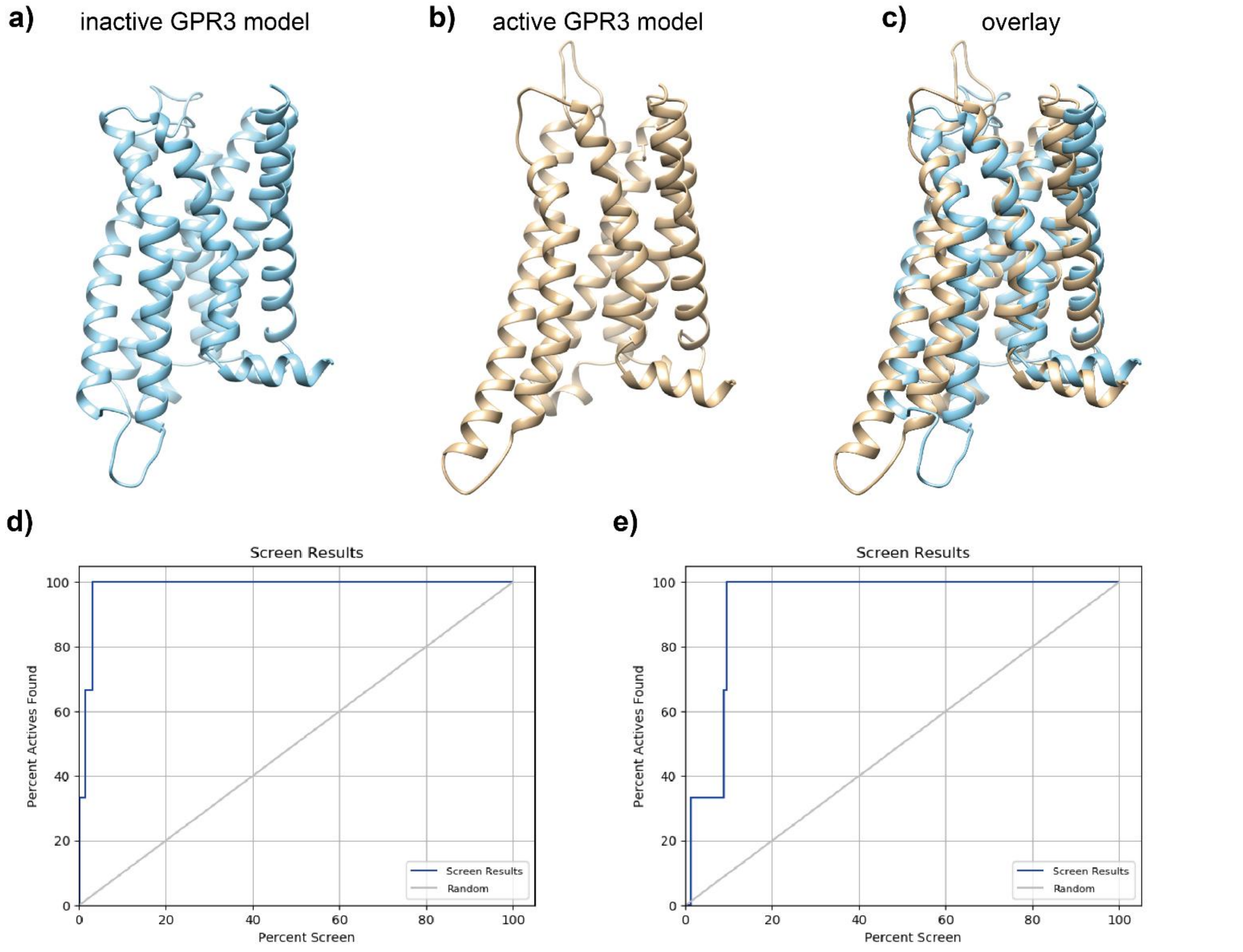

(**a-c**) Structural homology models of GPR3 in the inactive (**a**) and active (**b**) state, and their superimposition (**c**) based on experimental structures of CB_1 (PDB IDs 5TGZ and 6N4B, respectively). (**d-e**) Receiver operating characteristic (ROC) curves of the inactive- (**d**) and active-state (**e**) GPR3 model detecting AF64394 and two structural analogs as active GPR3 ligands in a subset of about 700 randomly selected virtual molecules.

Figure S5: Responses induced at GPR3-HaloTag/Nluc by initial screening hits.

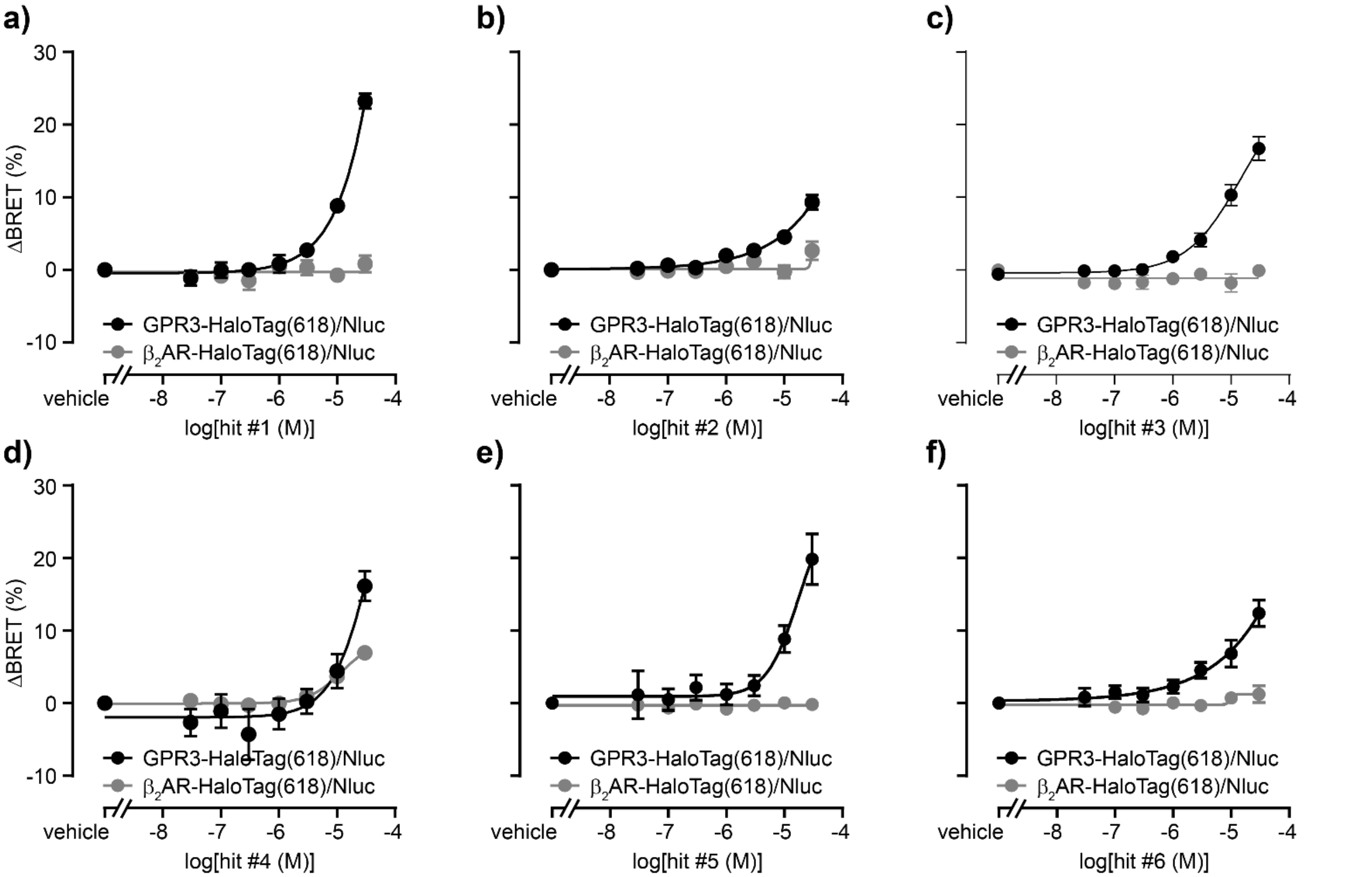

Concentration response curves of three initial hits from the virtual screen (**a-c**) and three chemical analogs (**d-f**) obtained with the GPR3 and β_2AR conformational sensors. Data show mean ± SEM of two (a and b) or three (c-f) independent experiments conducted in HEK293 cells stably expressing the indicated sensors.

Figure S6: Chemical structures of virtual hits VH1, VH2, VH3 and the corresponding in-house synthesized compounds **52**, **93**, and **115**.

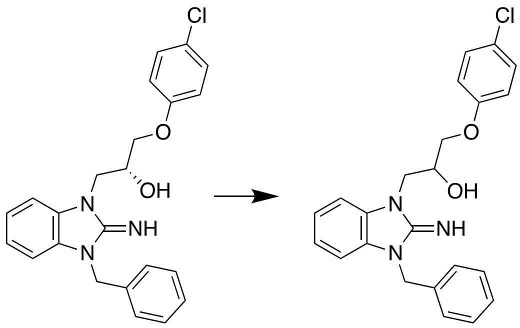

VH1 → **rac-VH1 / 52**

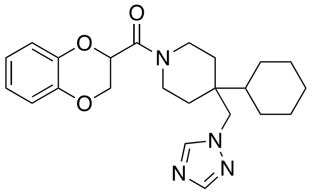

VH2 / 93

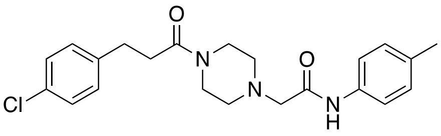

VH3 / 115

NMR spectra of 21-37, 52-77, 80-84, 93-110, and 115-124

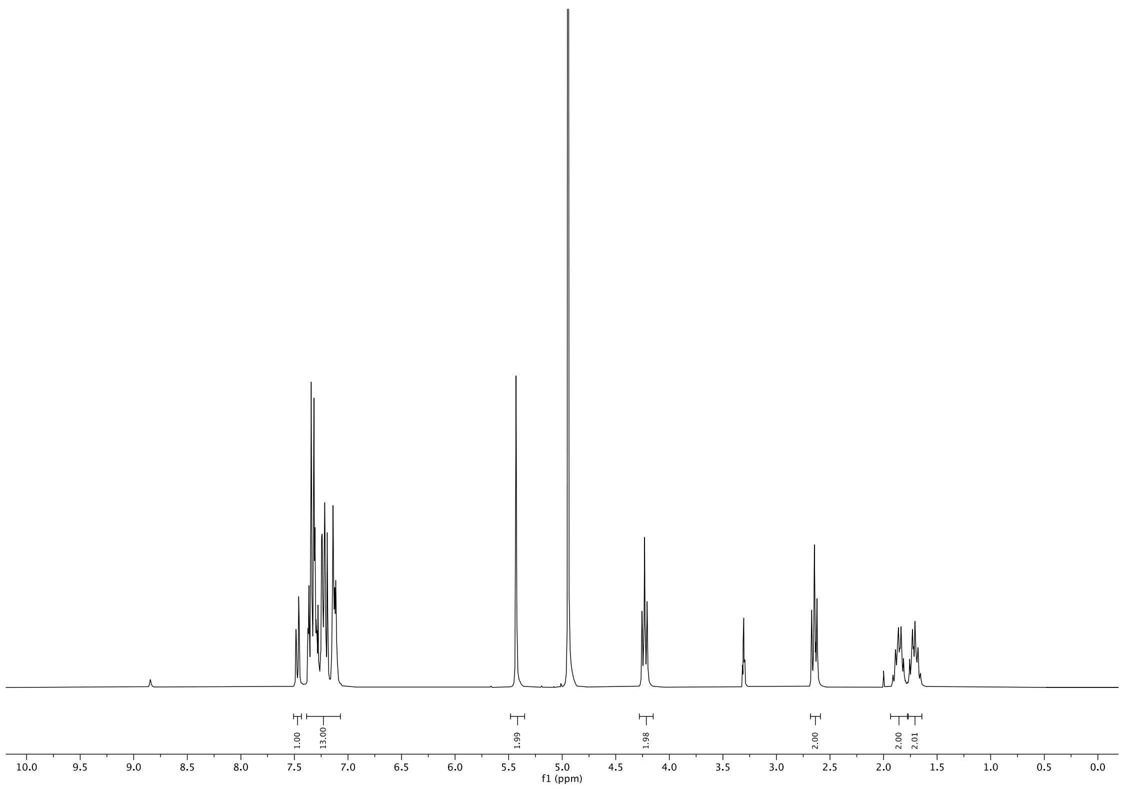

Figure S7. ^{1}H NMR spectrum (300 MHz, CD_3OD) of compound **21**.

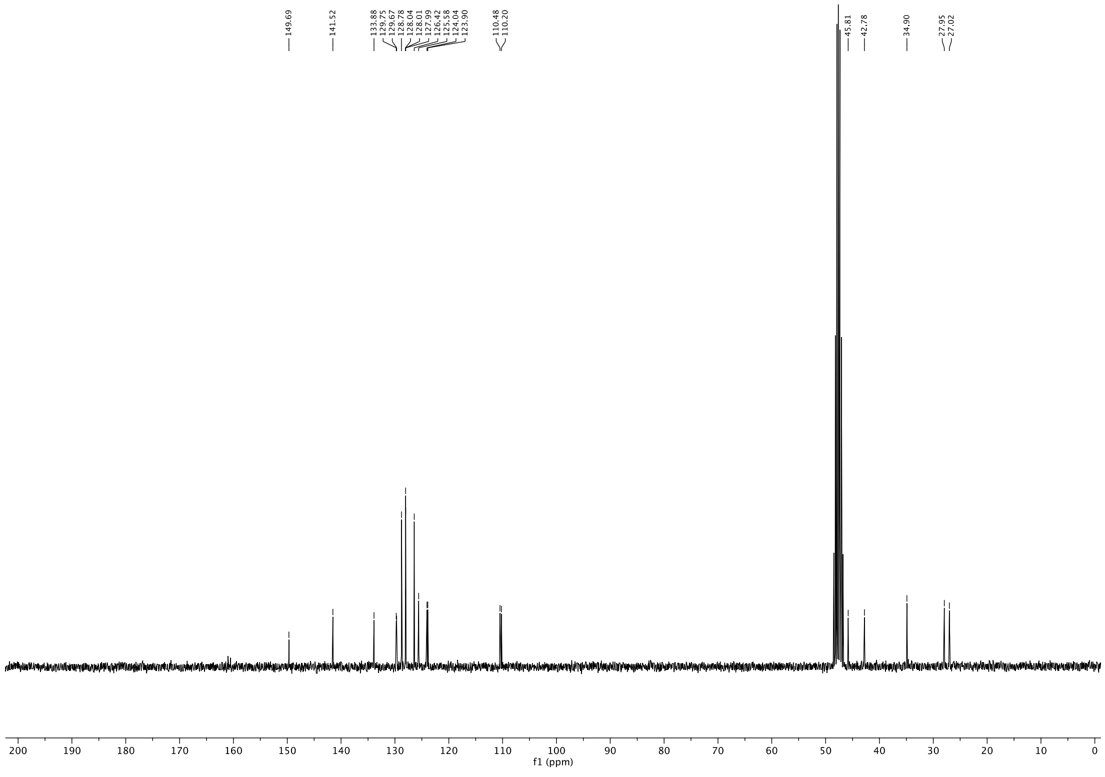

Figure S8. ^{13}C NMR spectrum (75 MHz, CD_3OD) of compound **21**.

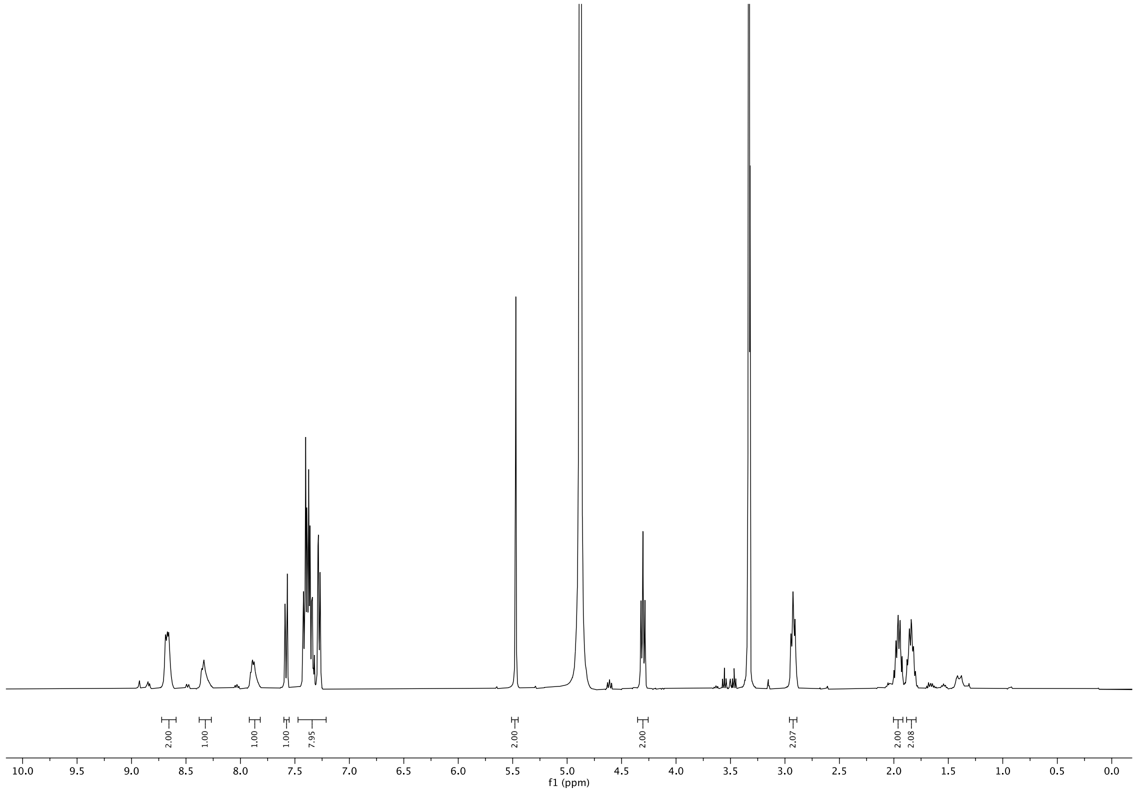

Figure S9. ^{1}H NMR spectrum (400 MHz, CD_3OD) of compound **22**.

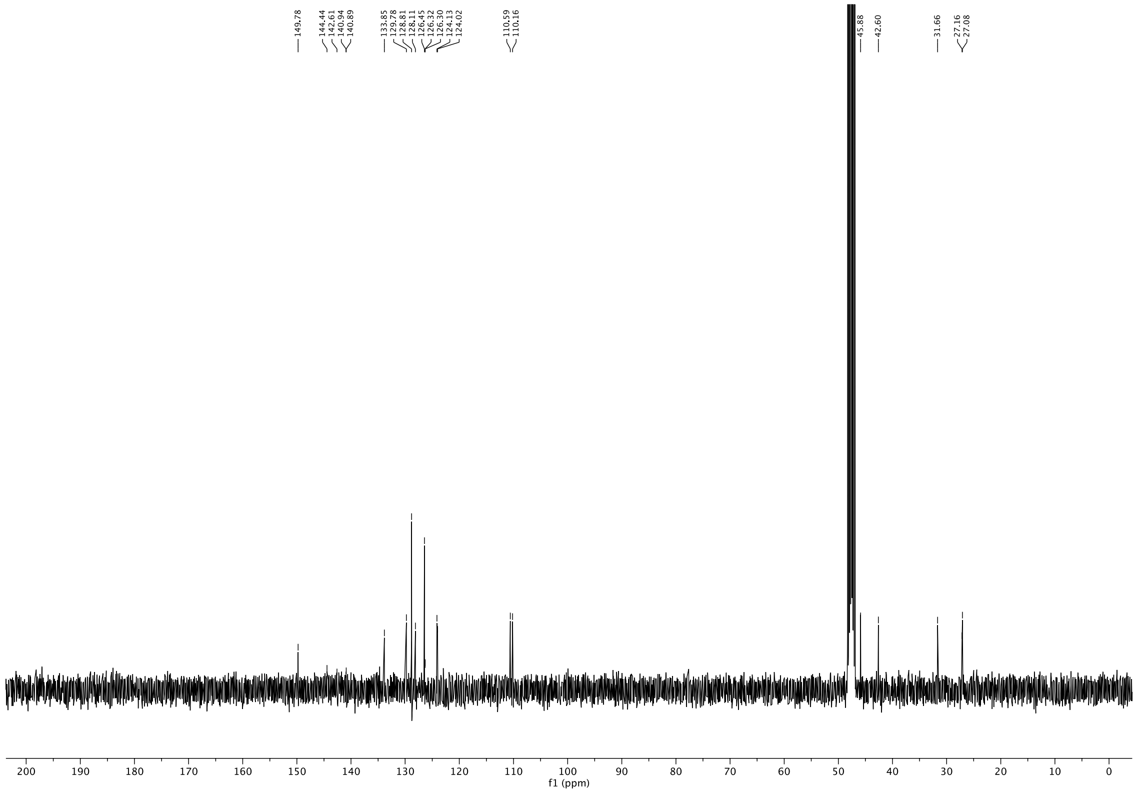

Figure S10. ^{13}C NMR spectrum (101 MHz, CD_3OD) of compound **22**.

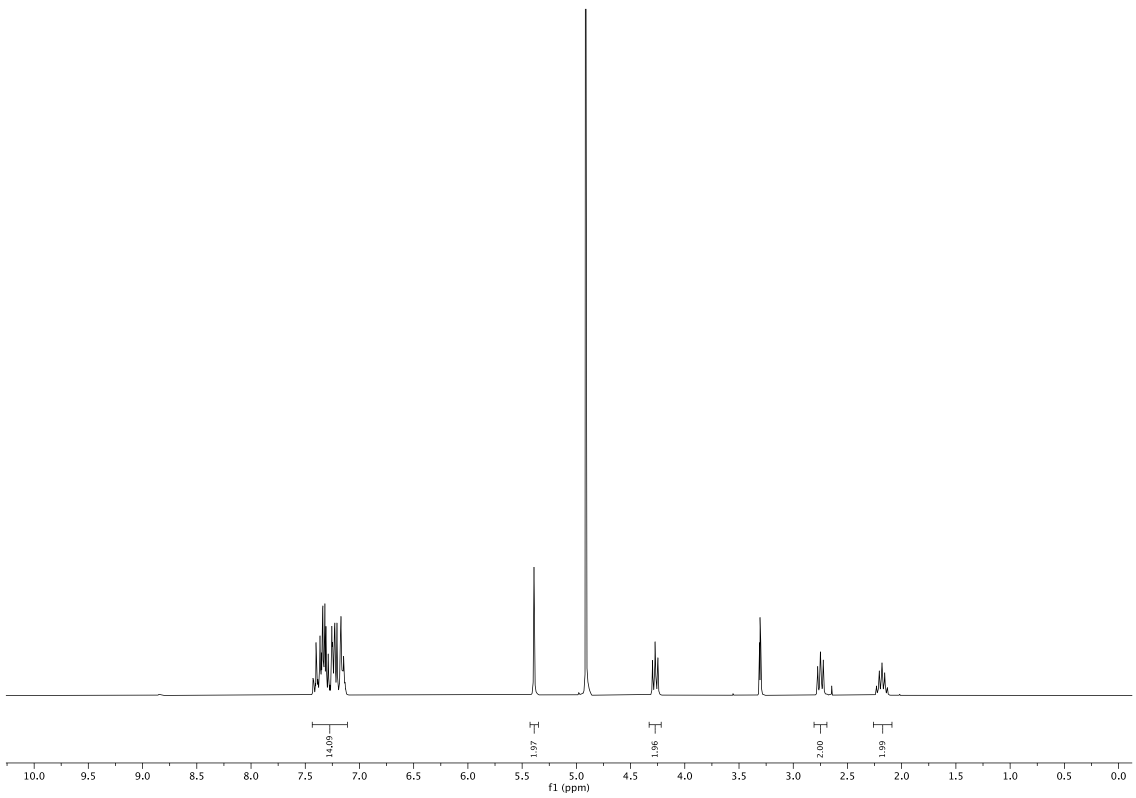

Figure S11. ^{1}H NMR spectrum (300 MHz, CD_3OD) of compound **23**.

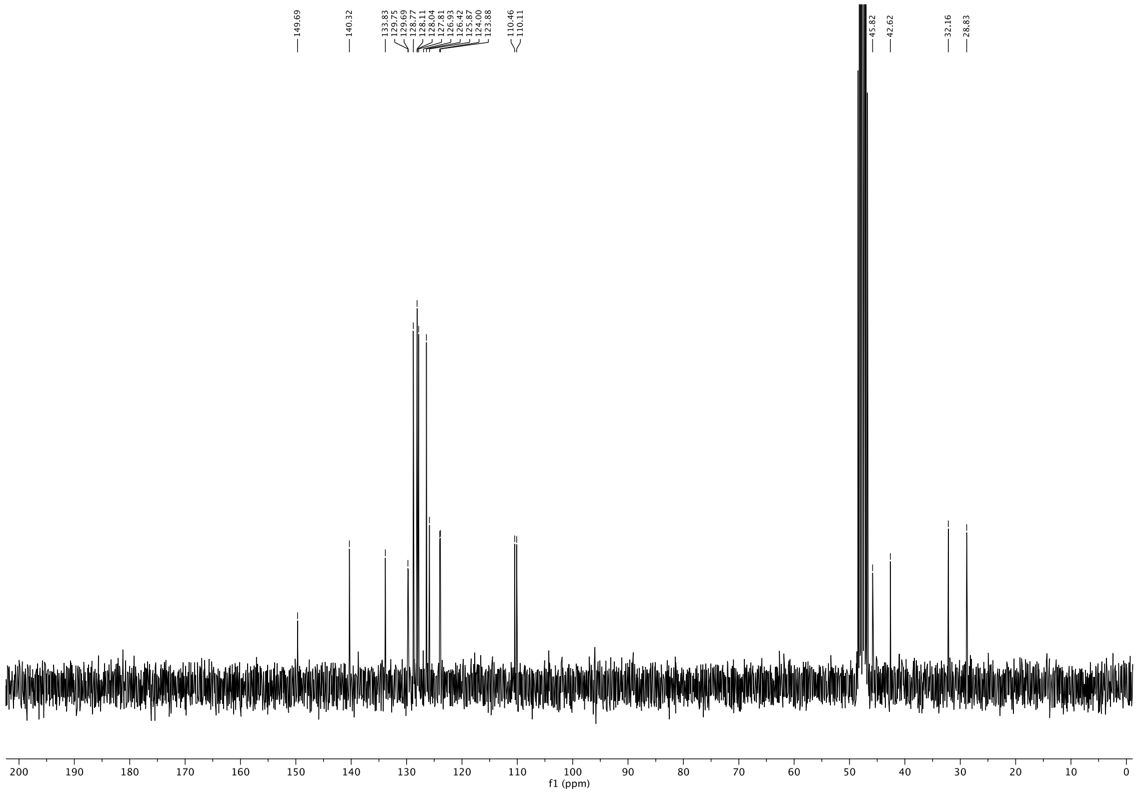

Figure S12. ^{13}C NMR spectrum (75 MHz, CD_3OD) of compound **23**.

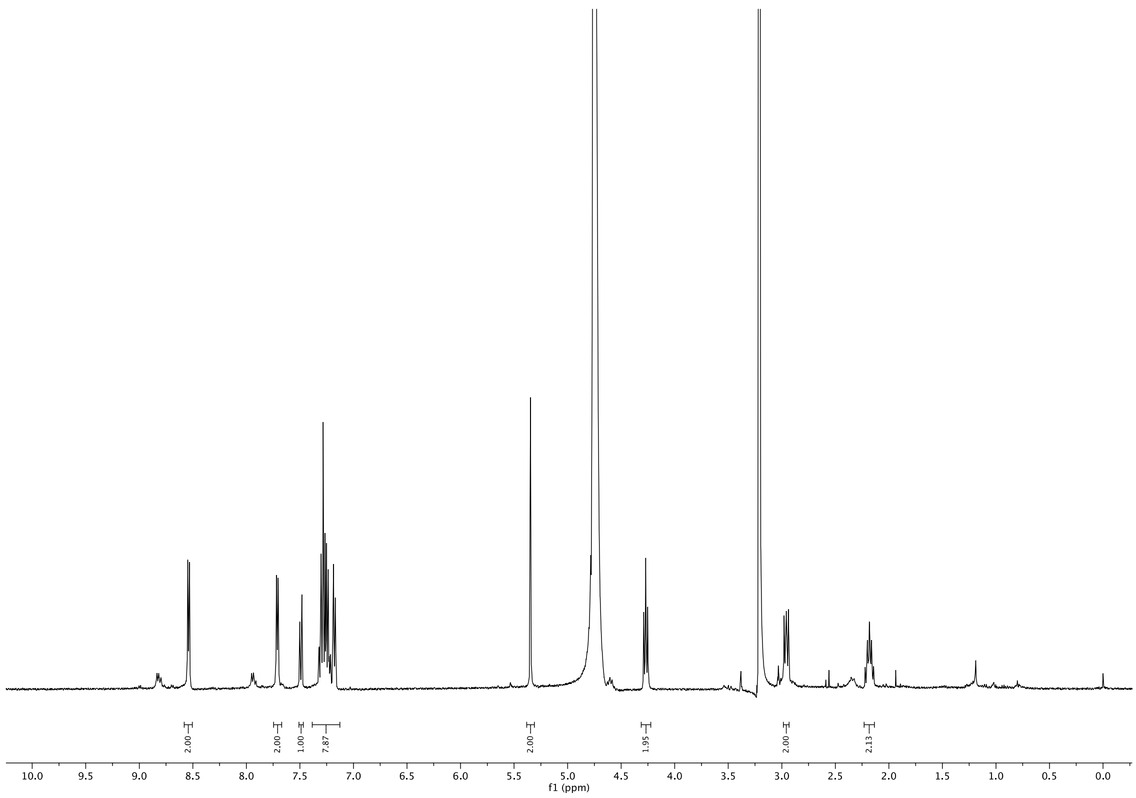

Figure S13. ^{1}H NMR spectrum (400 MHz, CD_3OD) of compound **24**.

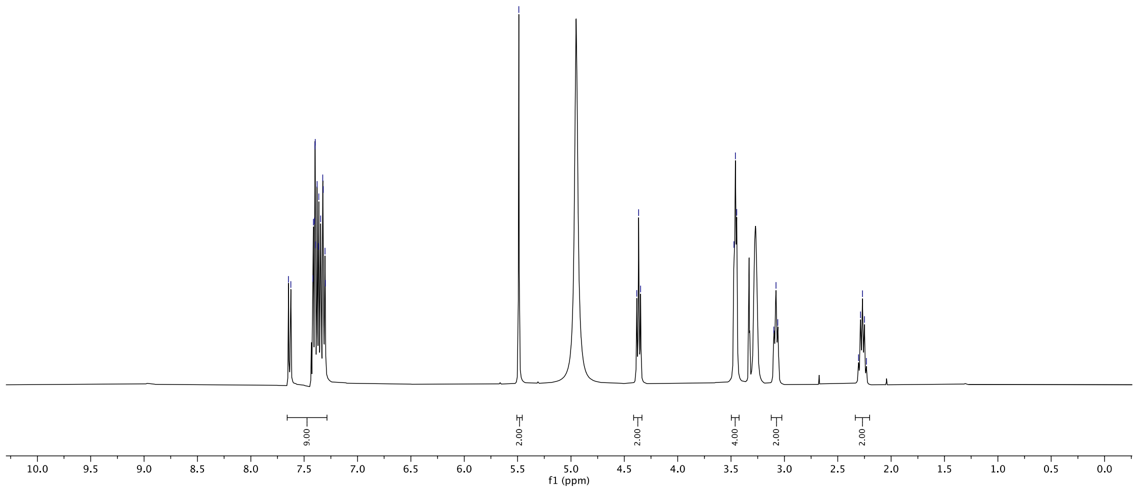

Figure S14. ^{1}H NMR spectrum (400 MHz, CD_3OD) of compound **25**.

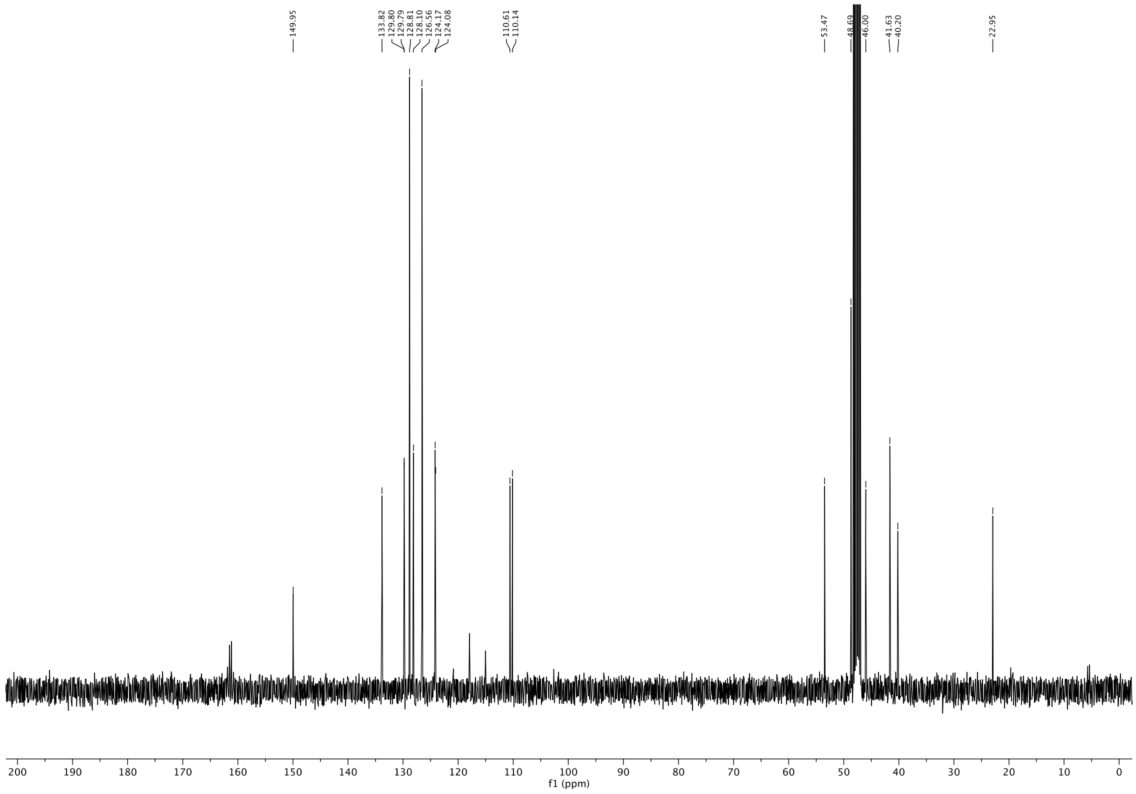

Figure S15. ^{13}C NMR spectrum (101 MHz, CD_3OD) of compound **25**.

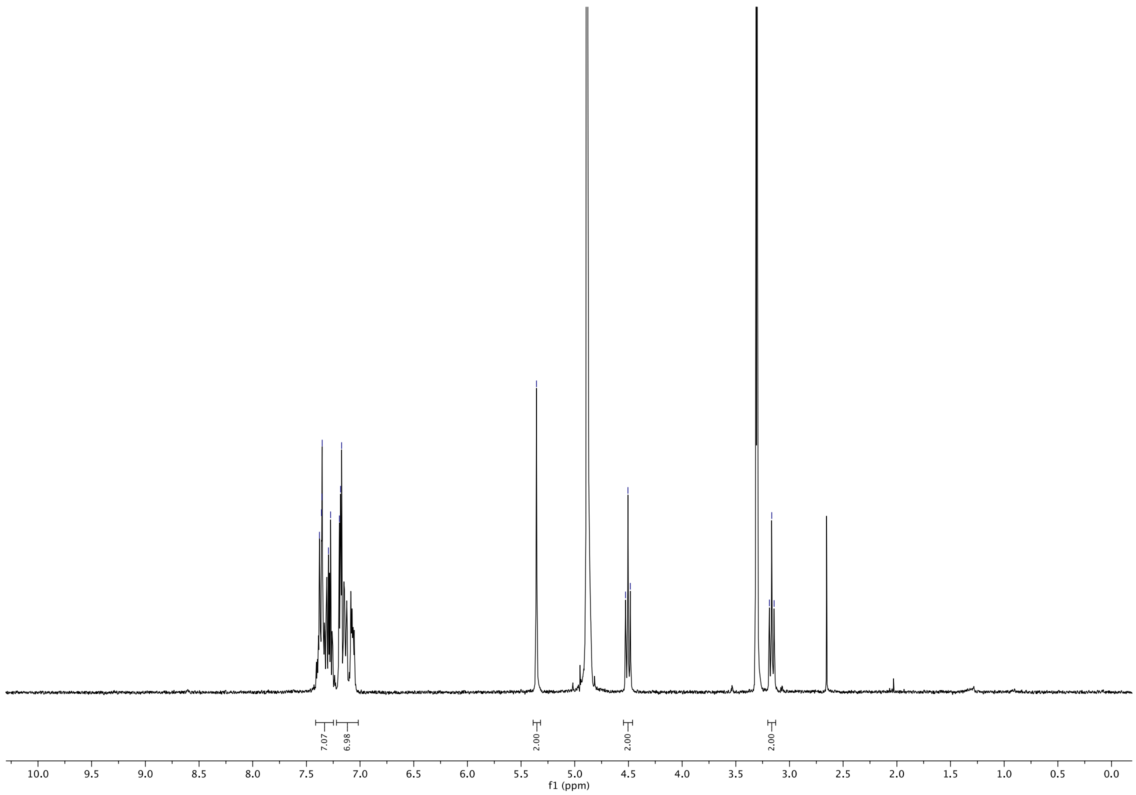

Figure S16. ^{1}H NMR spectrum (300 MHz, CD_3OD) of compound **26**.

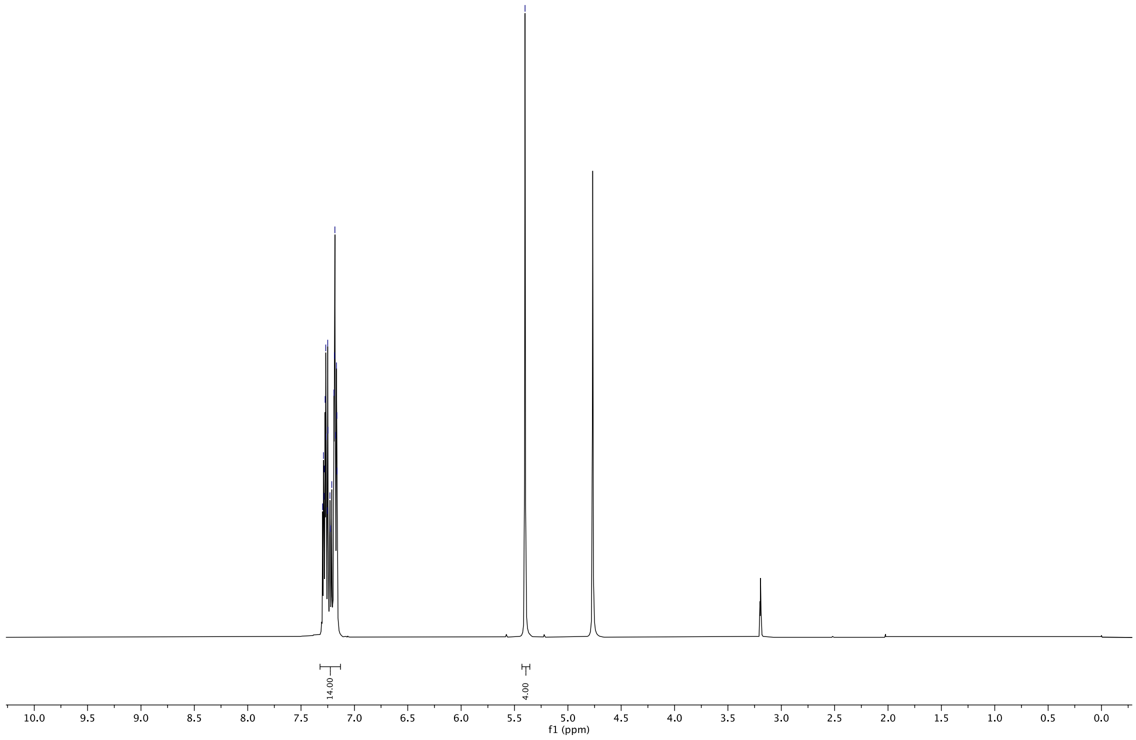

Figure S17. ^{1}H NMR spectrum (400 MHz, CD_3OD) of compound **27**.

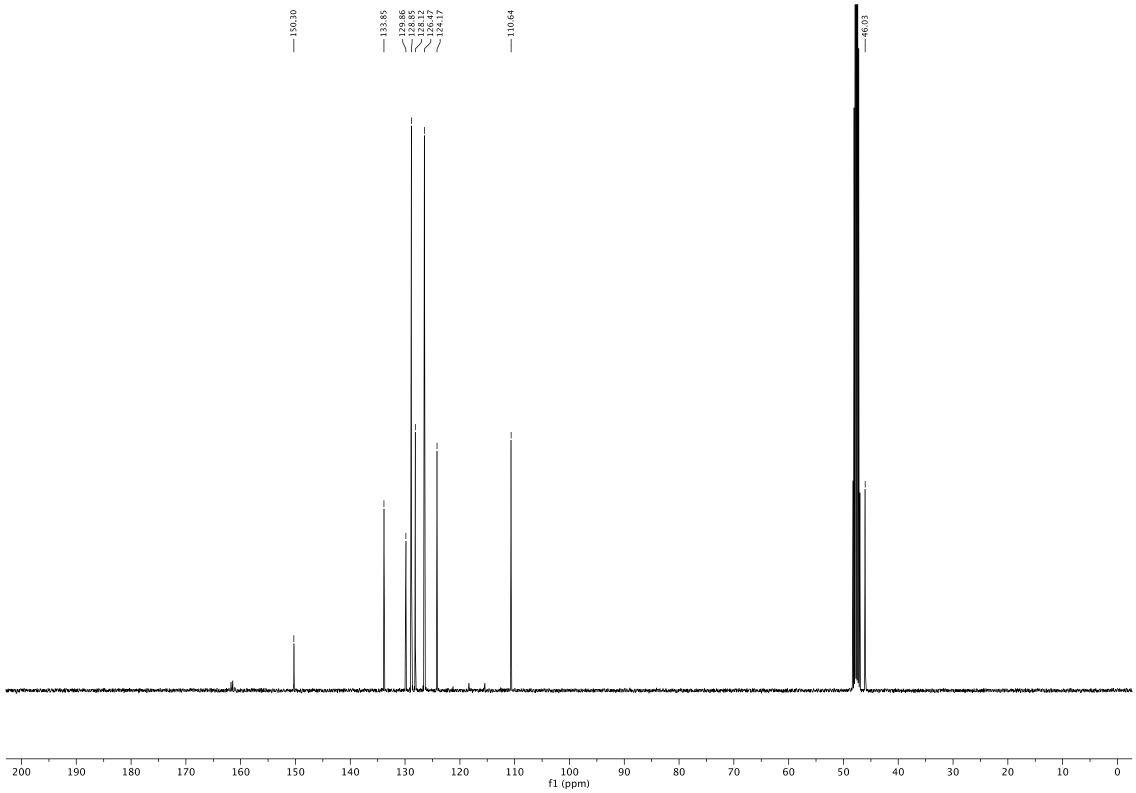

Figure S18. ^{13}C NMR spectrum (101 MHz, CD_3OD) of compound **27**.

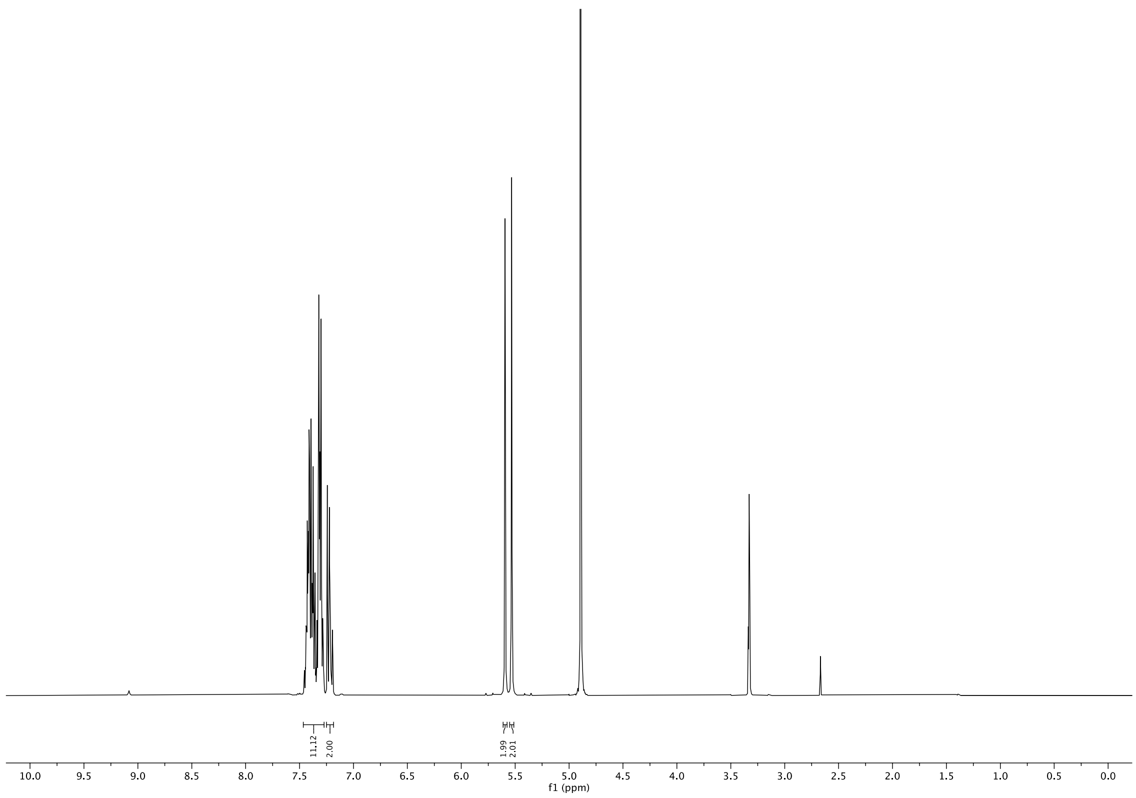

Figure S19. ^{1}H NMR spectrum (400 MHz, CD_3OD) of compound **28**.

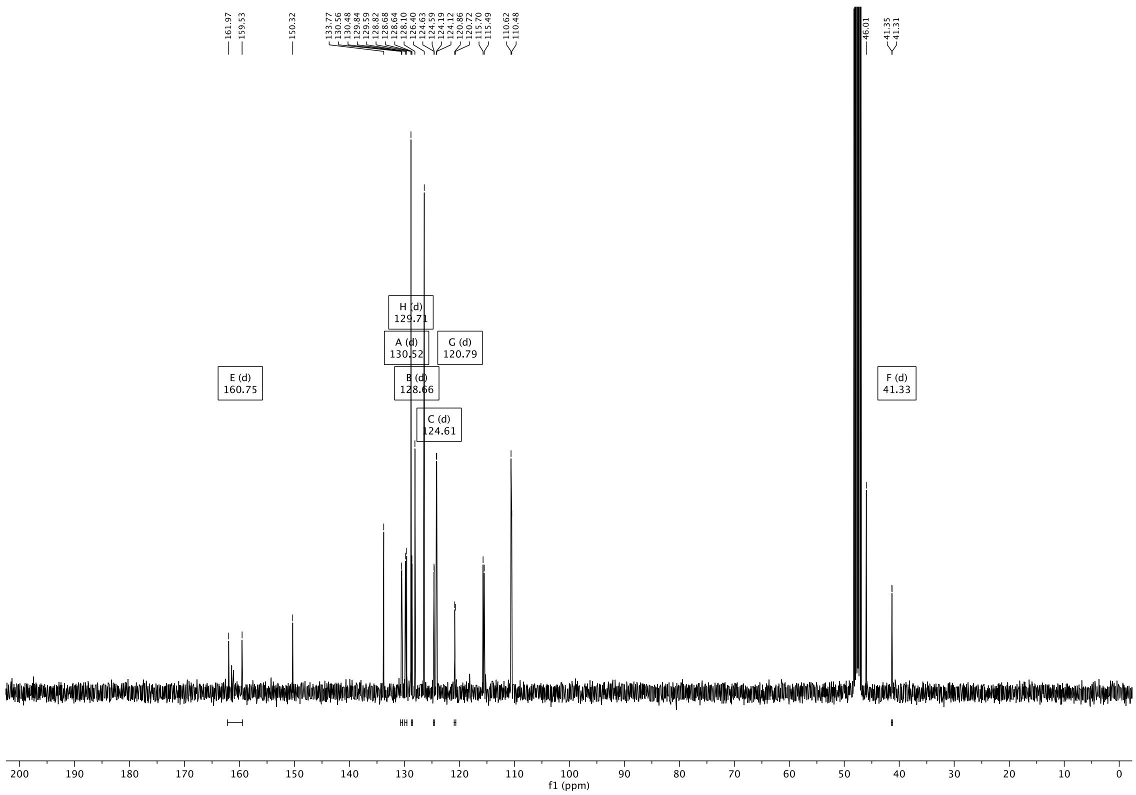

Figure S20. ^{13}C NMR spectrum (101 MHz, CD_3OD) of compound **28**.

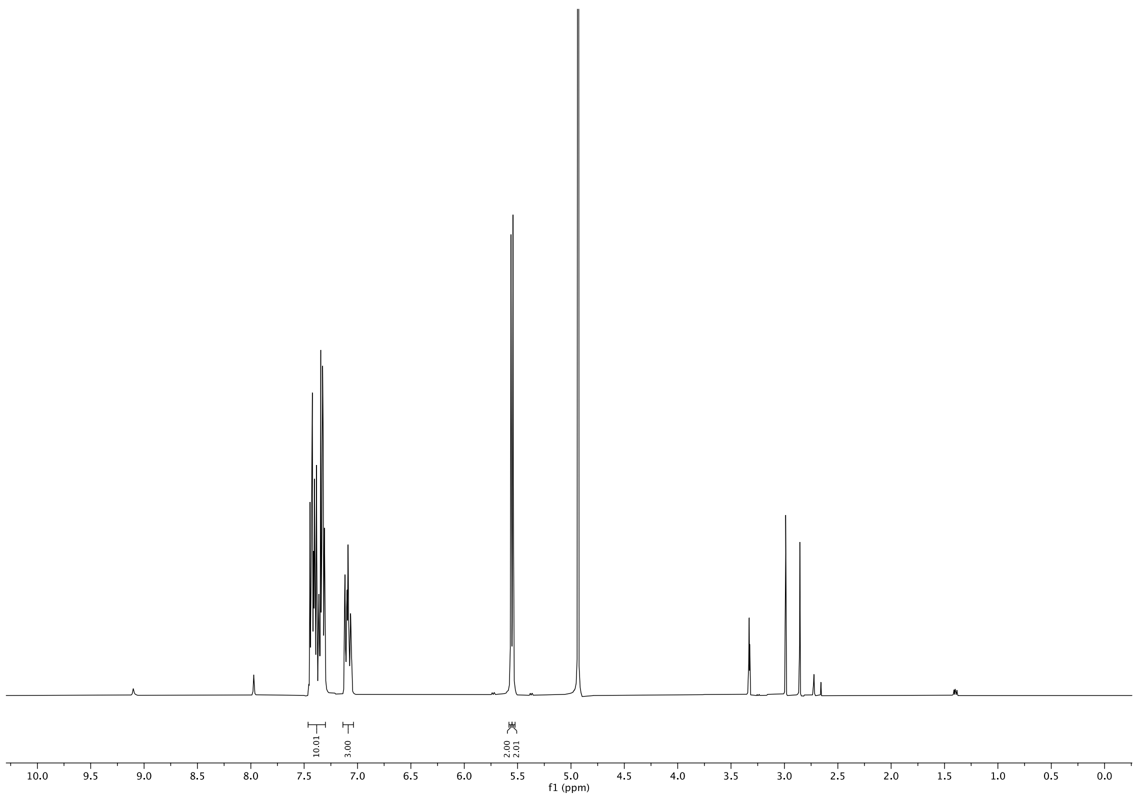

Figure S21. ^{1}H NMR spectrum (400 MHz, CD_3OD) of compound **29**.

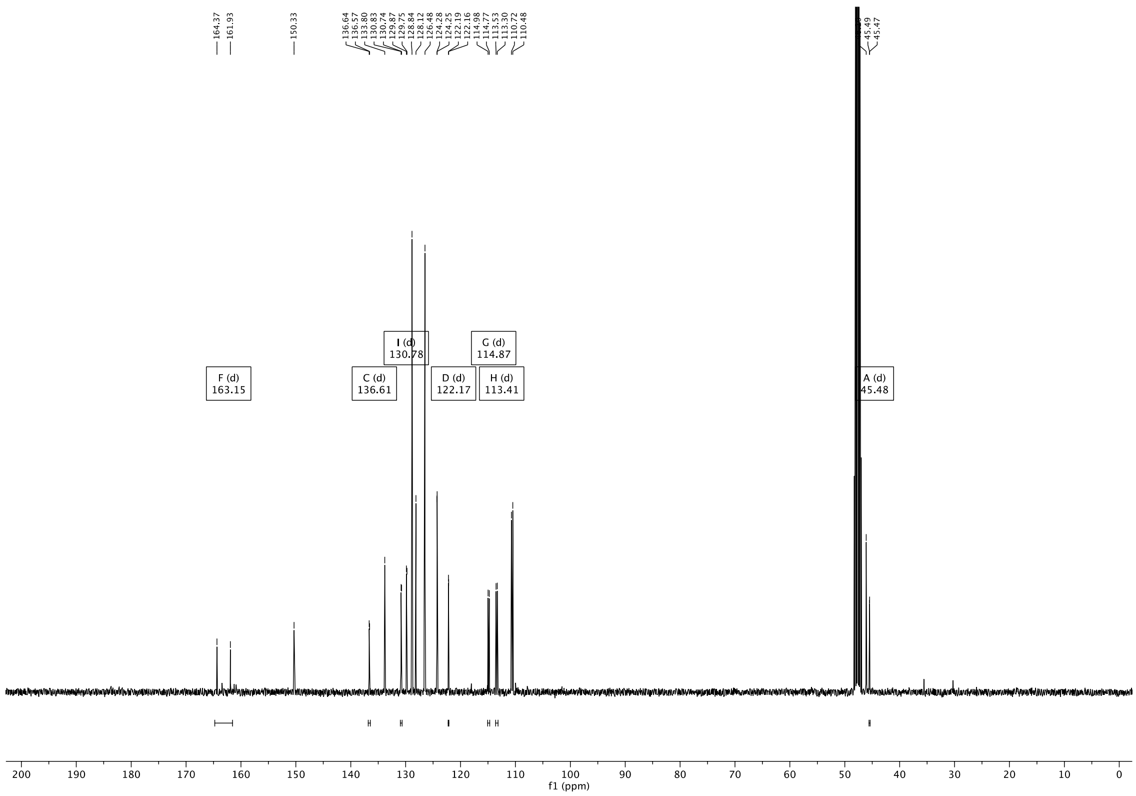

Figure S22. ^{13}C NMR spectrum (101 MHz, CD_3OD) of compound **29**.

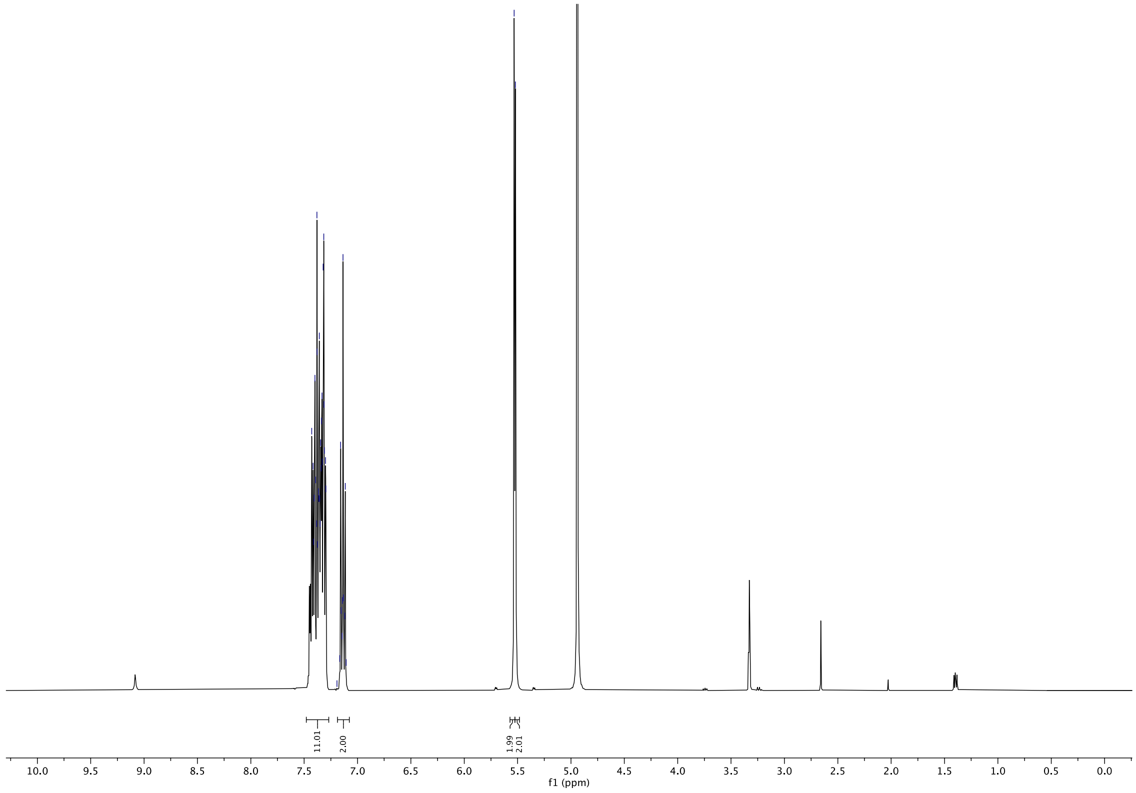

Figure S23. ^{1}H NMR spectrum (400 MHz, CD_3OD) of compound **30**.

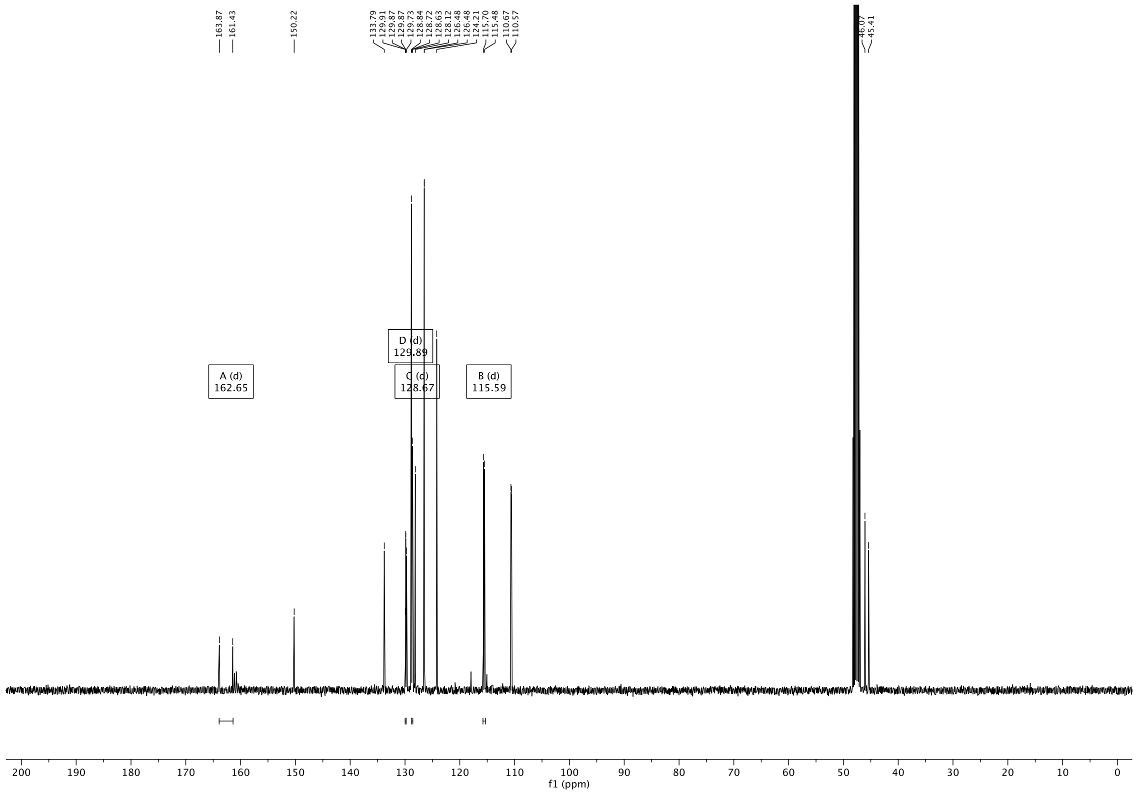

Figure S24. ^{13}C NMR spectrum (101 MHz, CD_3OD) of compound **30**.

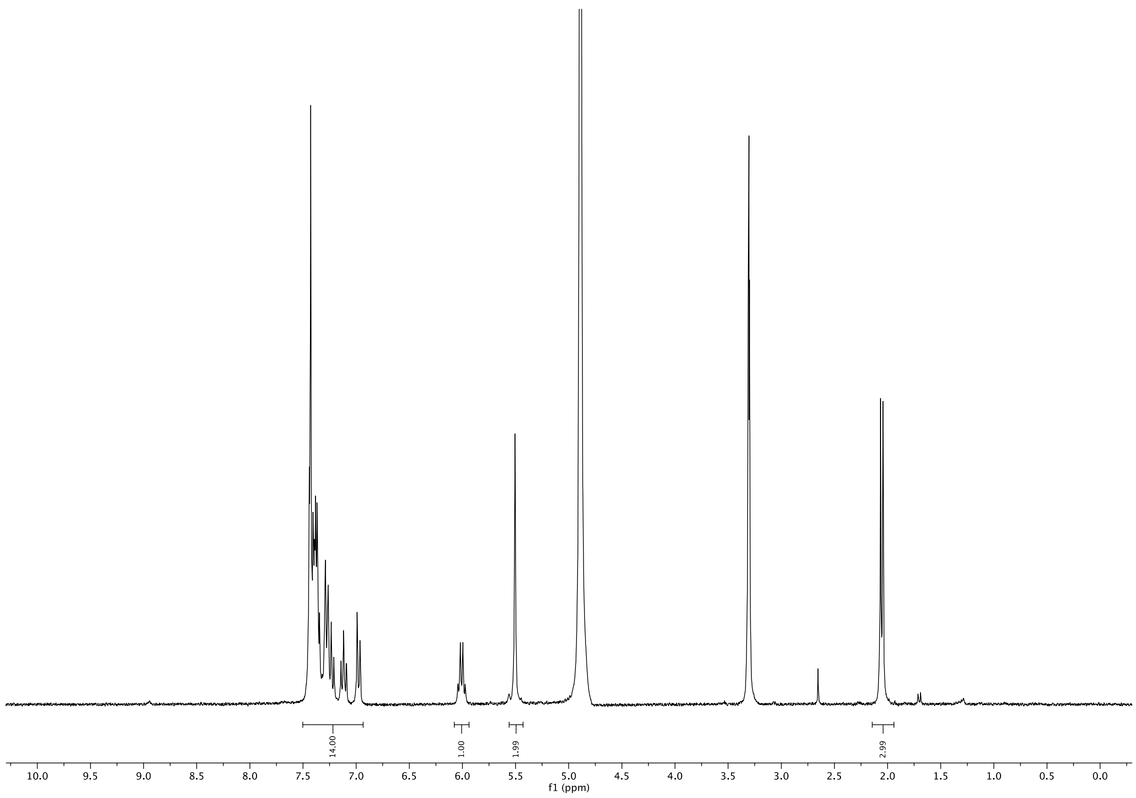

Figure S25. ^{1}H NMR spectrum (300 MHz, CD_3OD) of compound **31**.

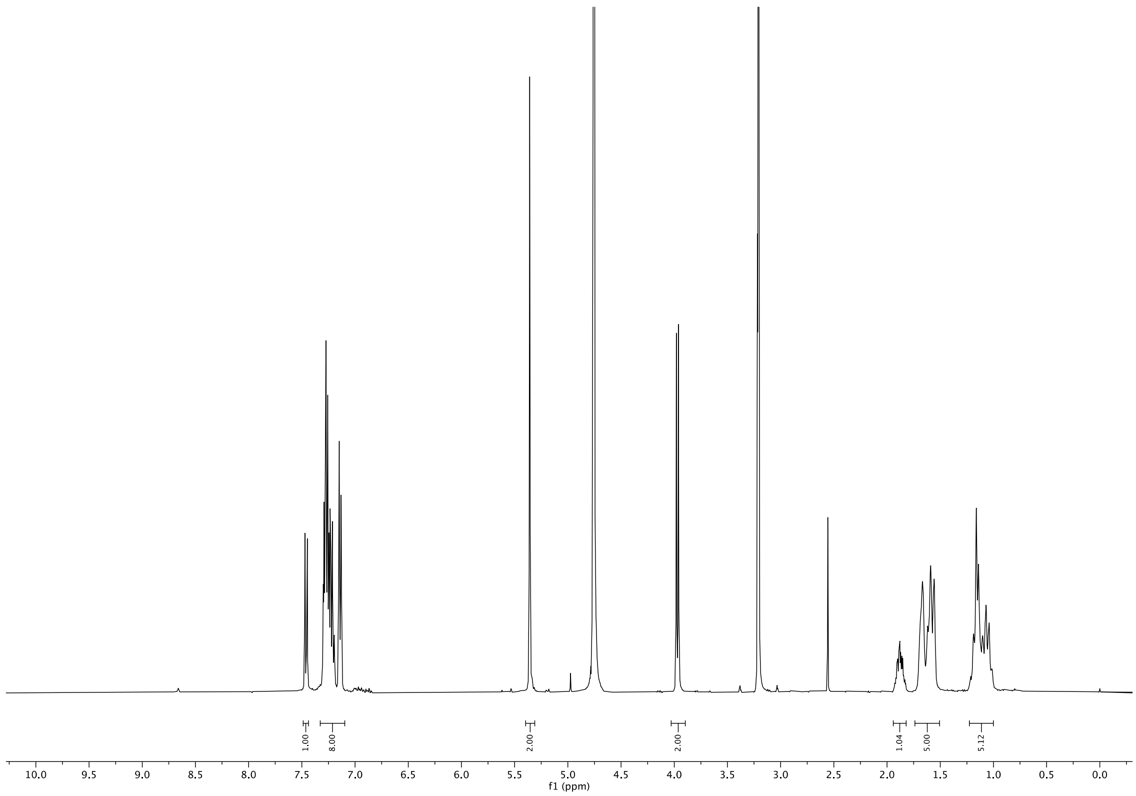

Figure S26. ^{1}H NMR spectrum (400 MHz, CD_3OD) of compound **32**.

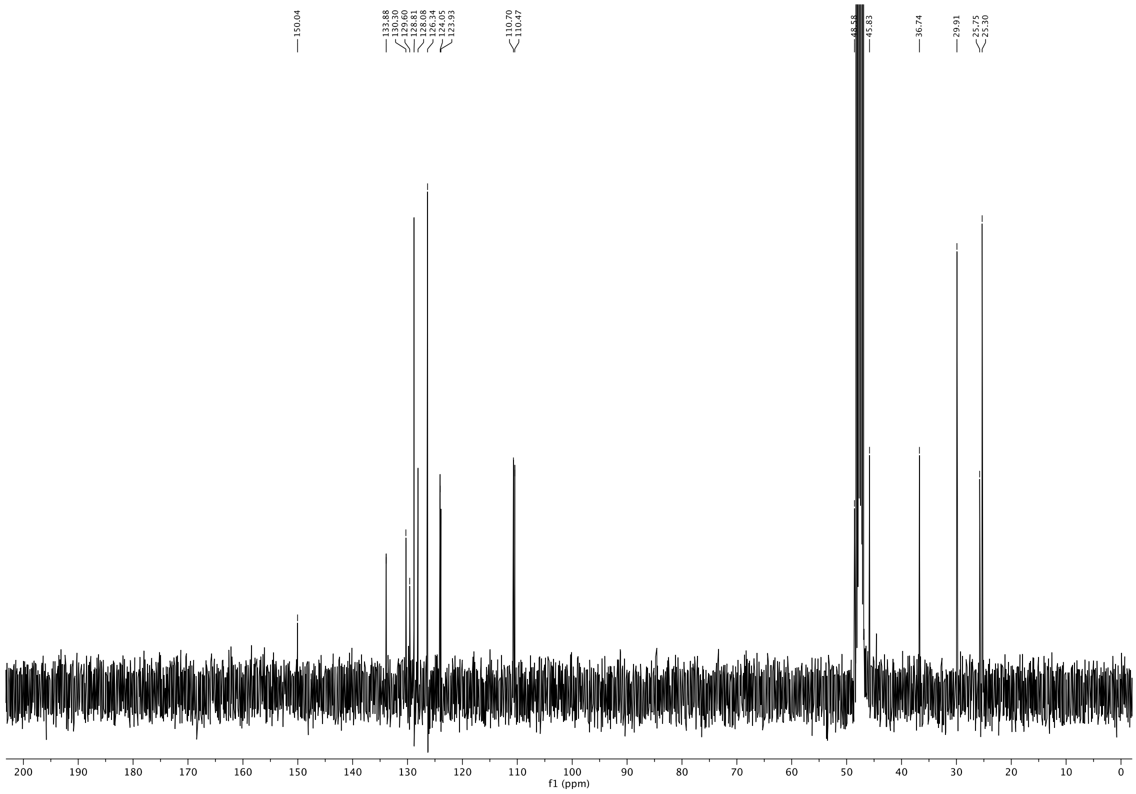

Figure S27. ^{13}C NMR spectrum (101 MHz, CD_3OD) of compound **32**.

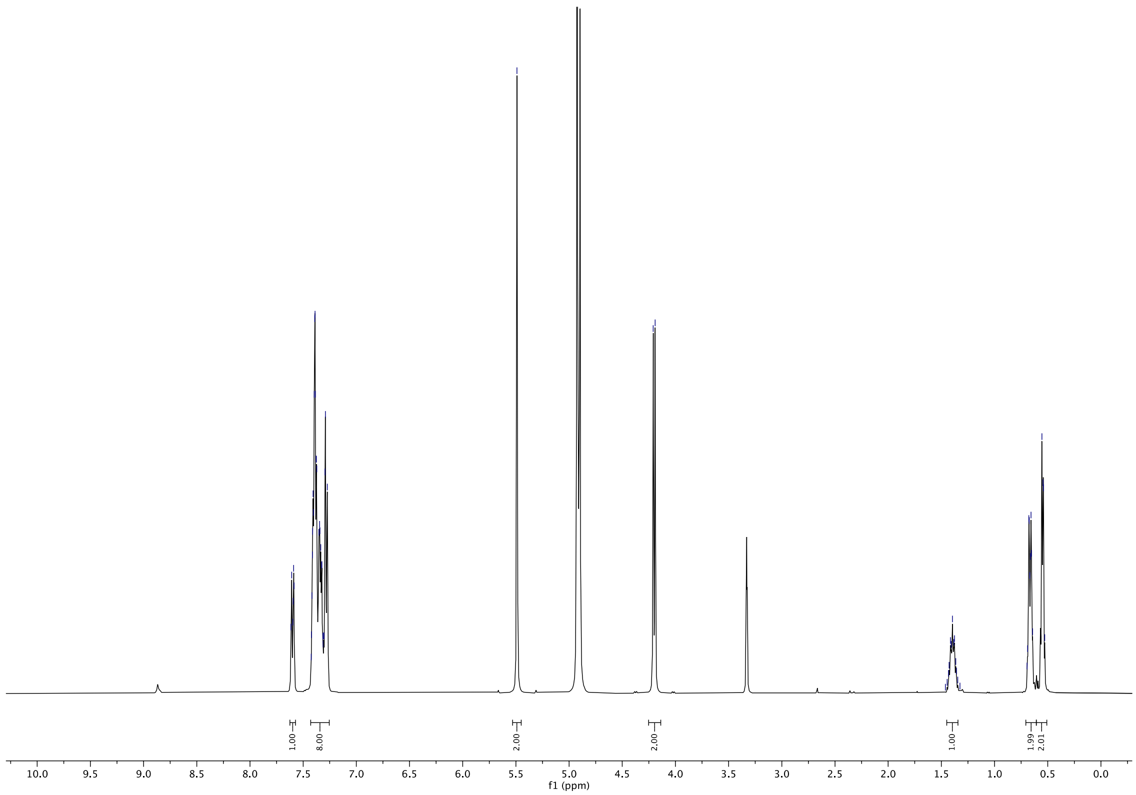

Figure S28. ^{1}H NMR spectrum (400 MHz, CD_3OD) of compound **33**.

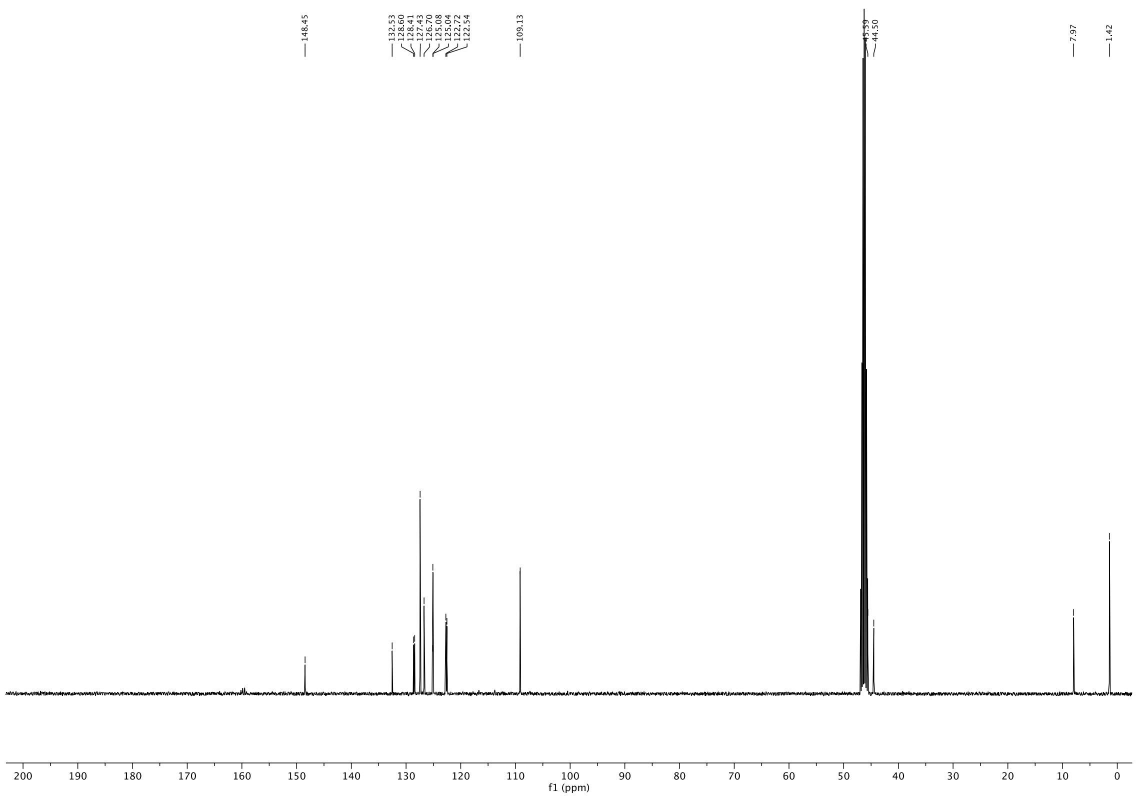

Figure S29. ^{13}C NMR spectrum (101 MHz, CD_3OD) of compound **33**.

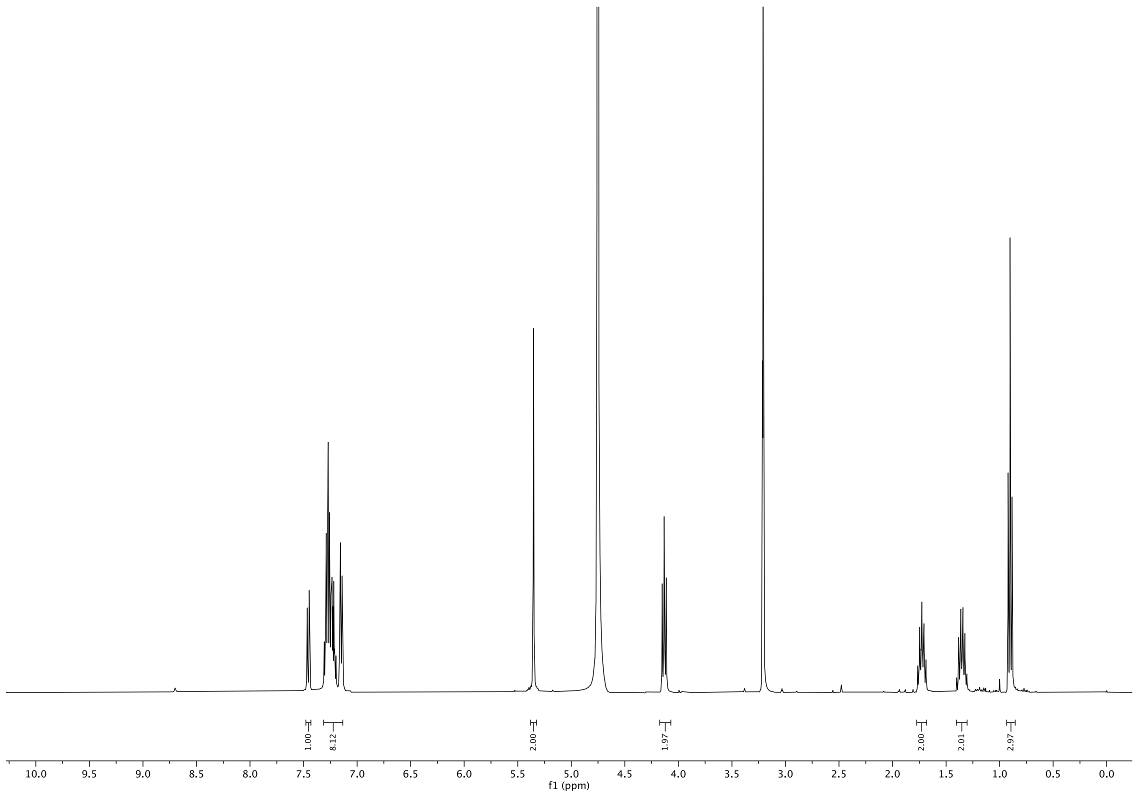

Figure S30. ^{1}H NMR spectrum (400 MHz, CD_3OD) of compound **34**.

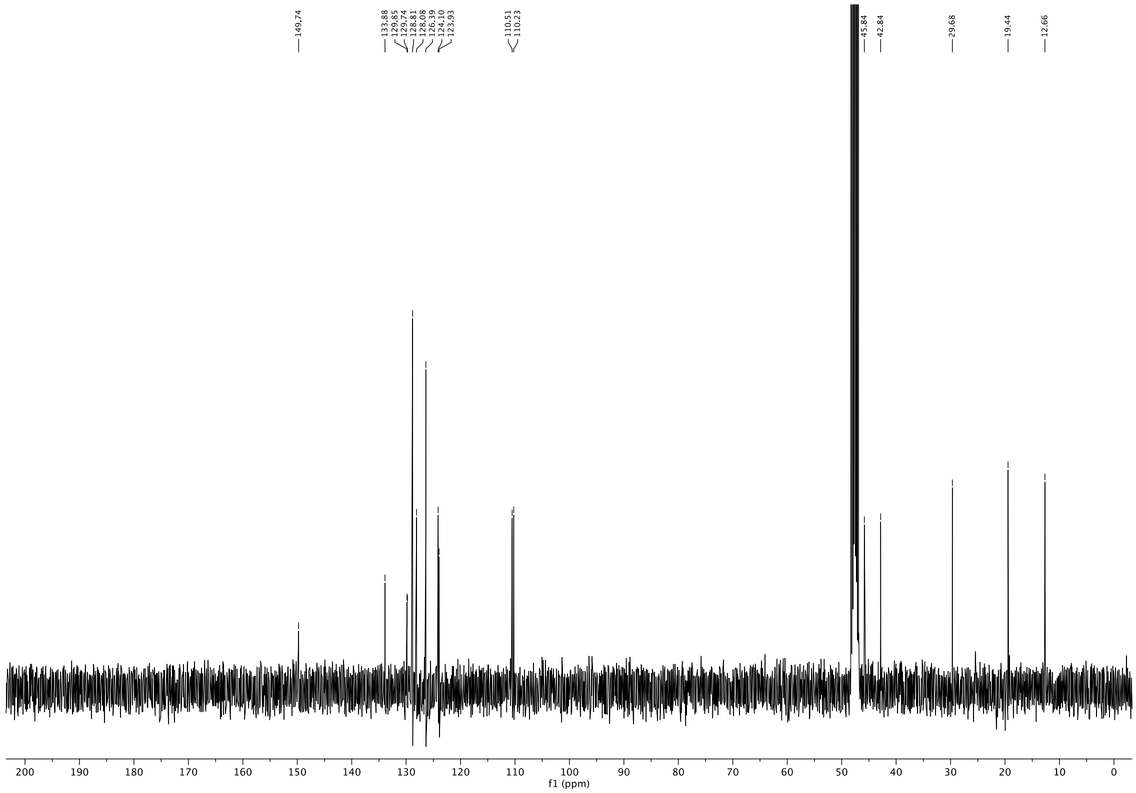

Figure S31. ^{13}C NMR spectrum (101 MHz, CD_3OD) of compound **34**.

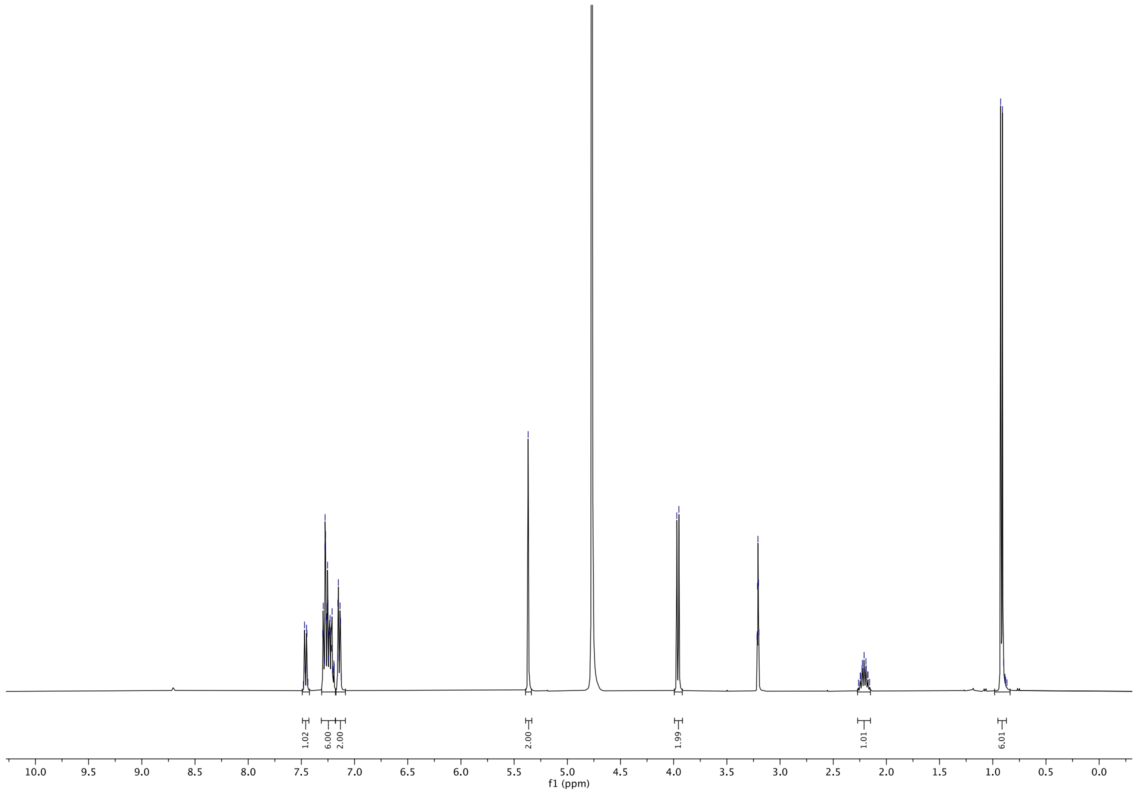

Figure S32. ^{1}H NMR spectrum (400 MHz, CD_3OD) of compound **35**.

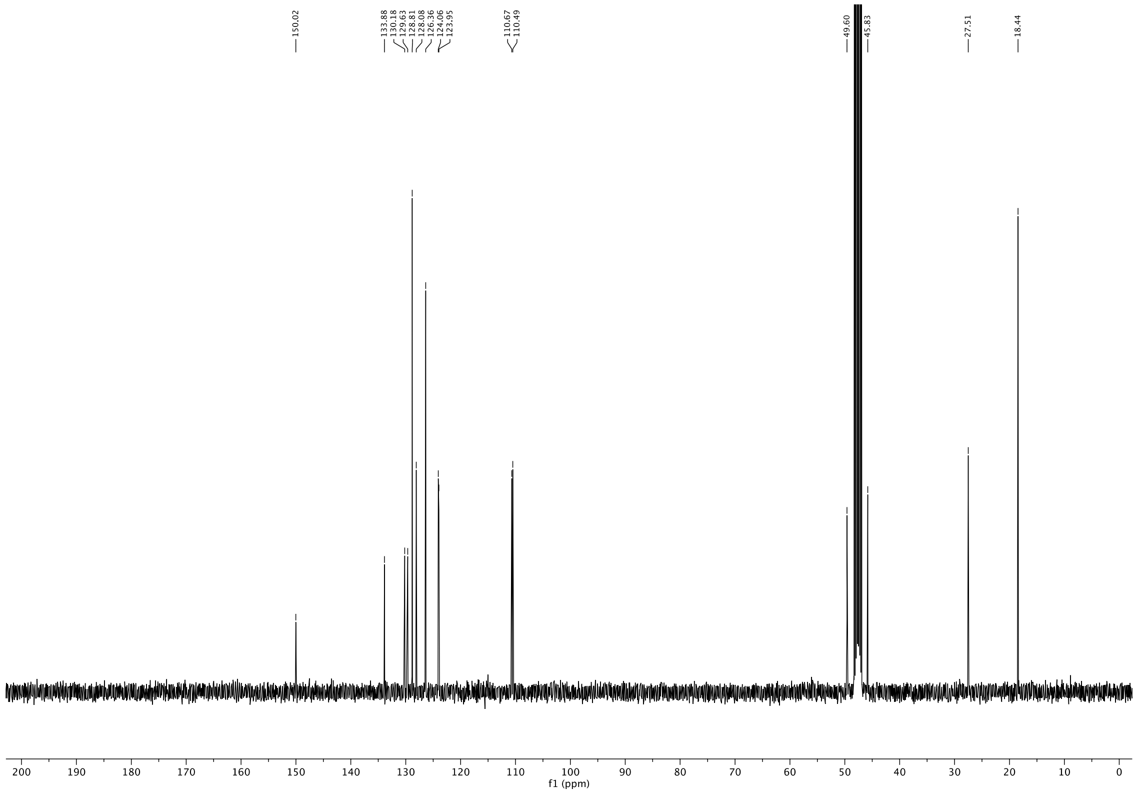

Figure S33. ^{13}C NMR spectrum (101 MHz, CD_3OD) of compound **35**.

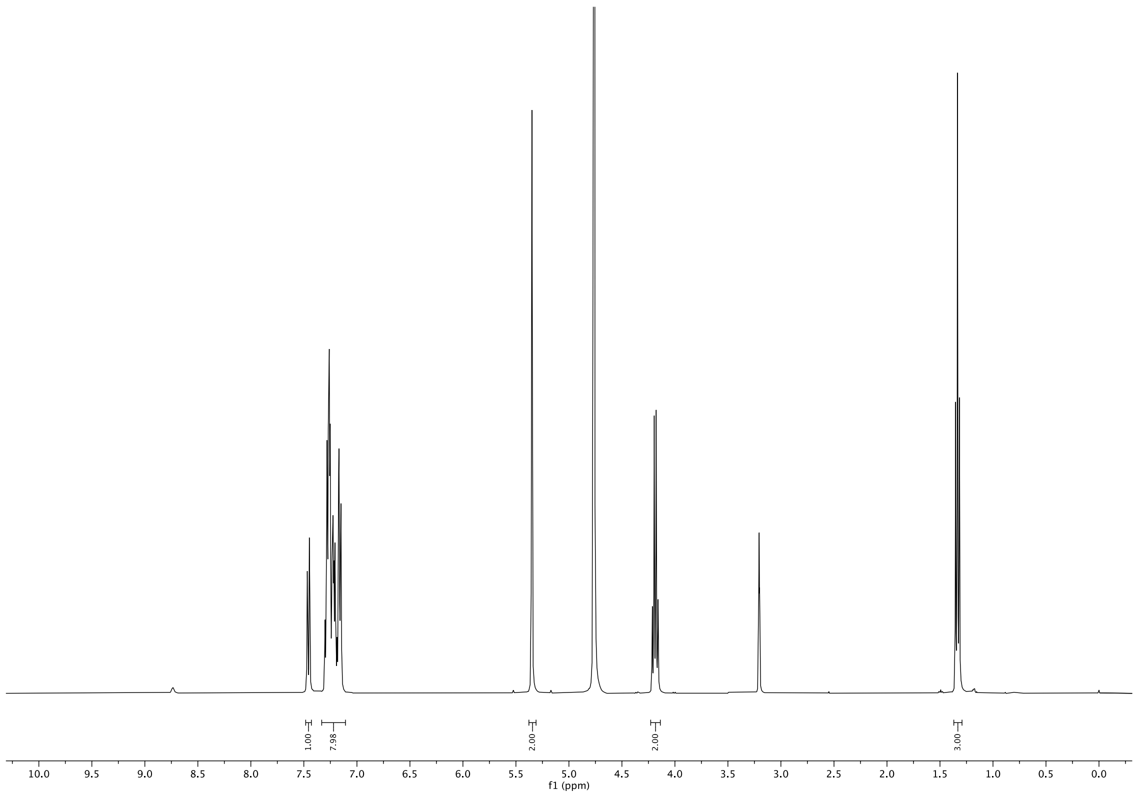

Figure S34. ^{1}H NMR spectrum (400 MHz, CD_3OD) of compound **36**.

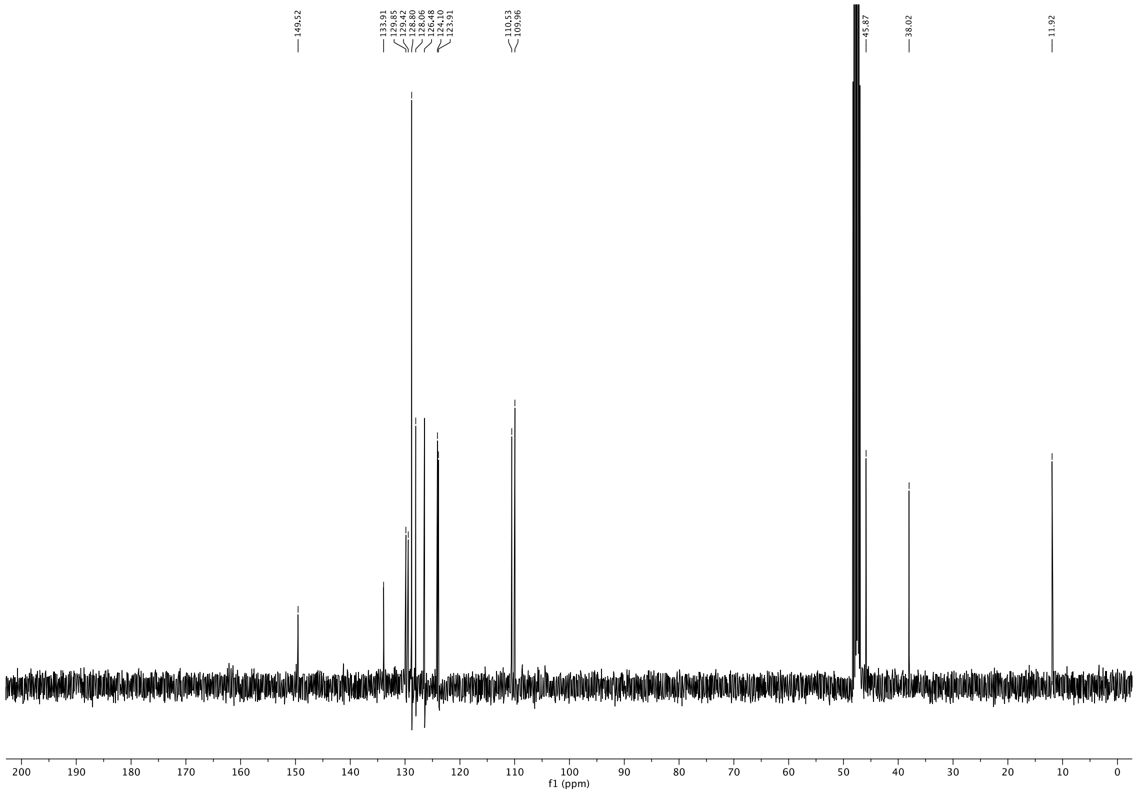

Figure S35. ^{13}C NMR spectrum (101 MHz, CD_3OD) of compound **36**.

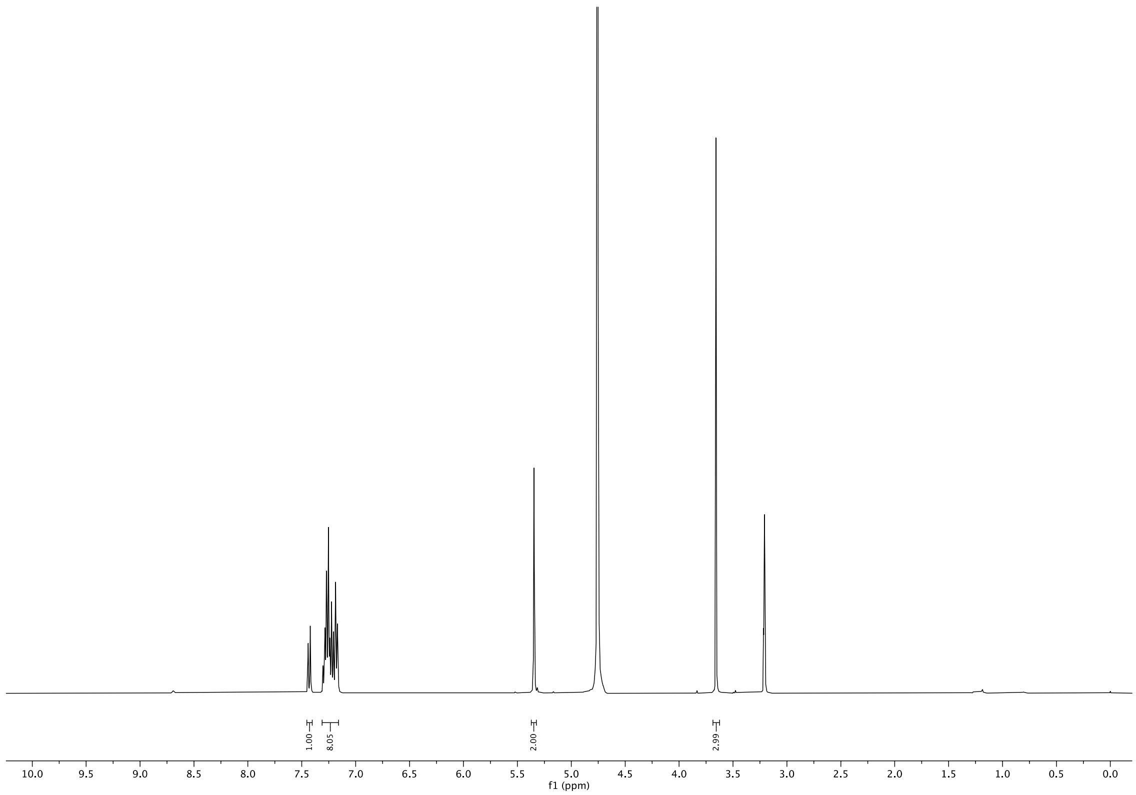

Figure S36. ^{1}H NMR spectrum (400 MHz, CD_3OD) of compound **37**.

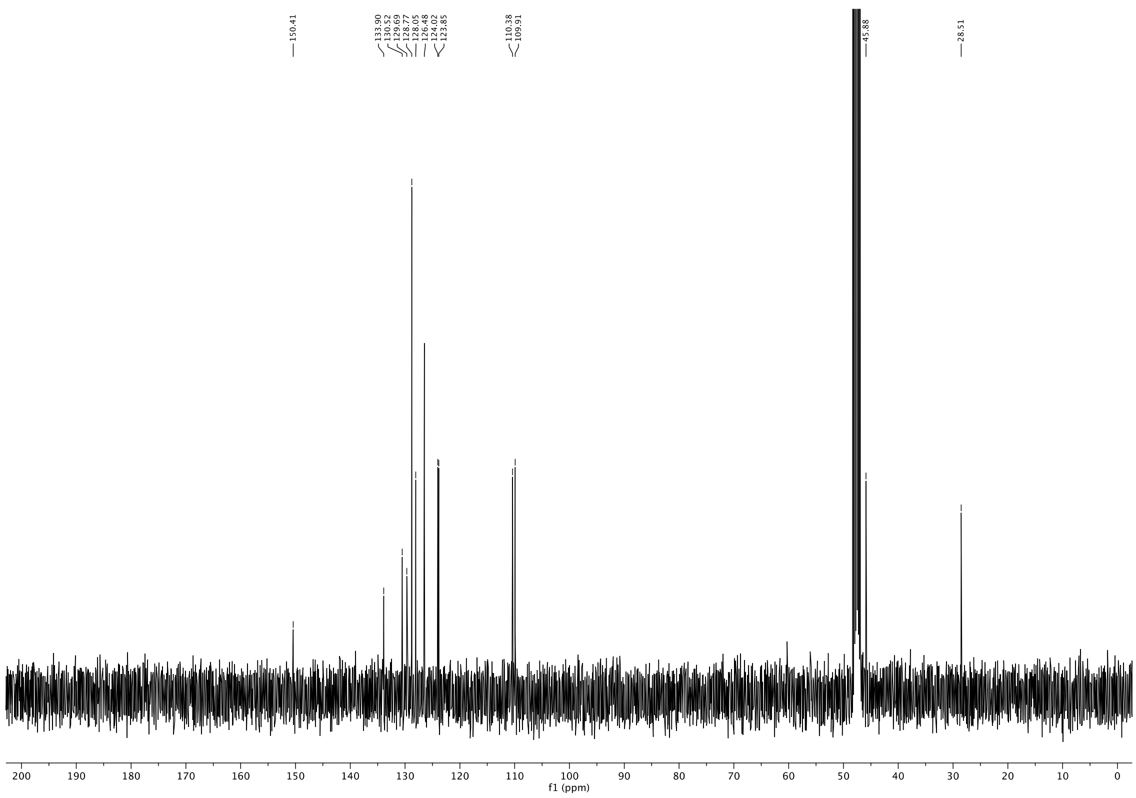

Figure S37. ^{13}C NMR spectrum (101 MHz, CD_3OD) of compound **37**.

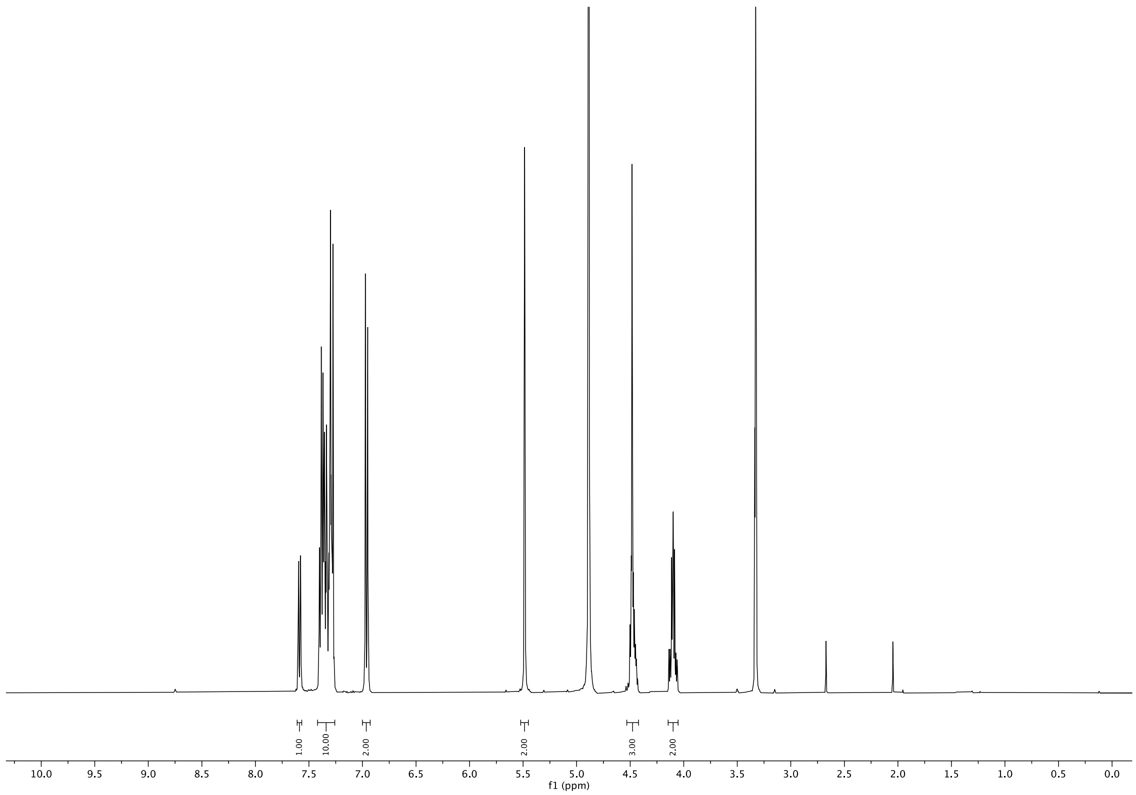

Figure S38. ^{1}H NMR spectrum (400 MHz, CD_3OD) of compound **52**.

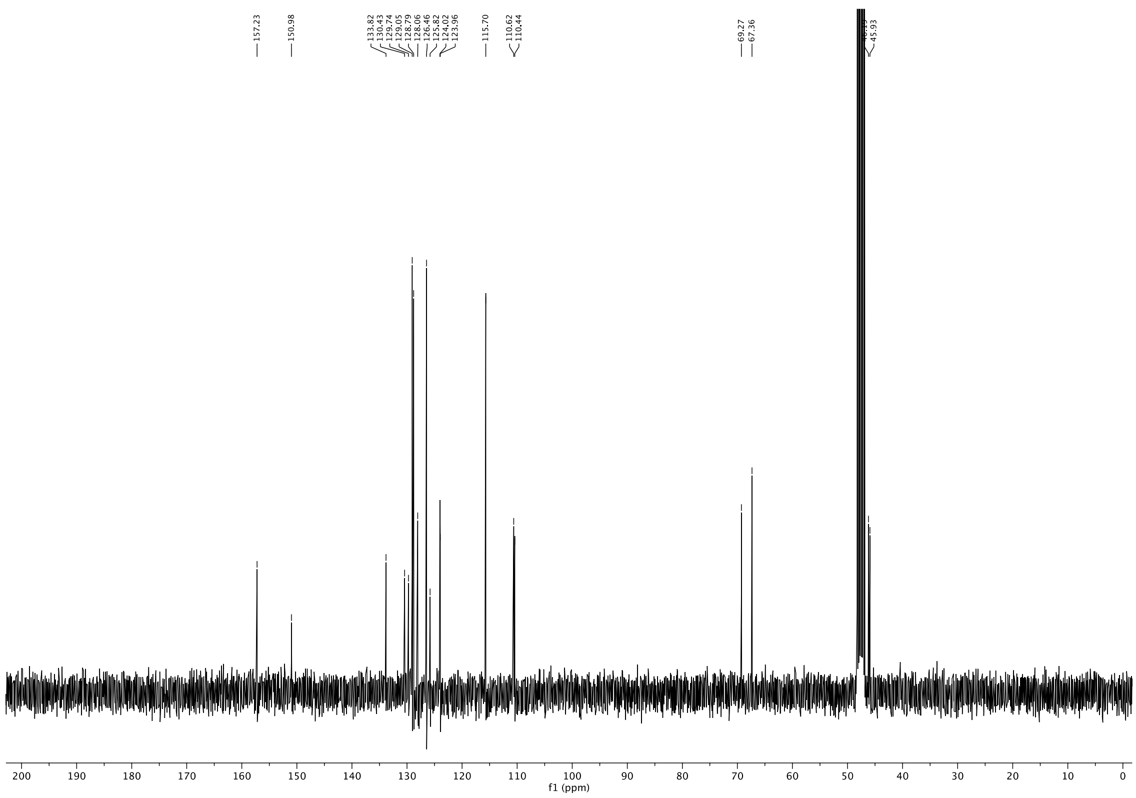

Figure S39. ^{13}C NMR spectrum (101 MHz, CD_3OD) of compound **52**.

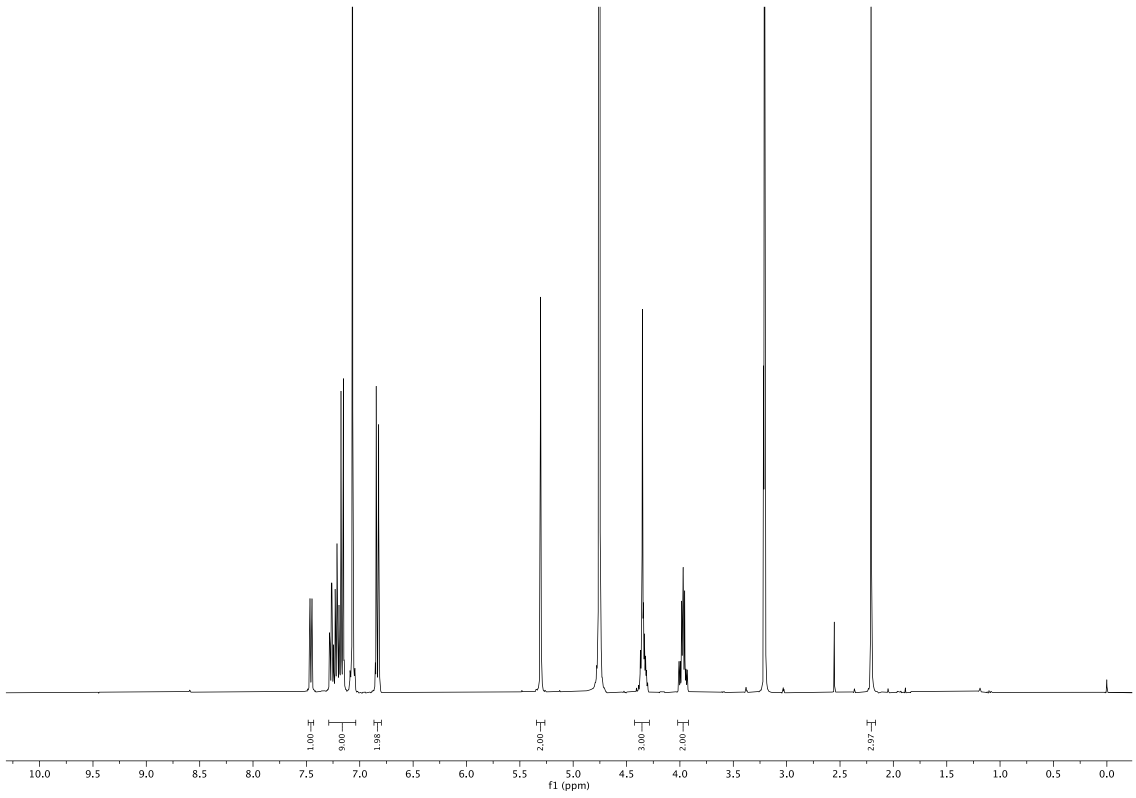

Figure S40. ^{1}H NMR spectrum (400 MHz, CD_3OD) of compound **53**.

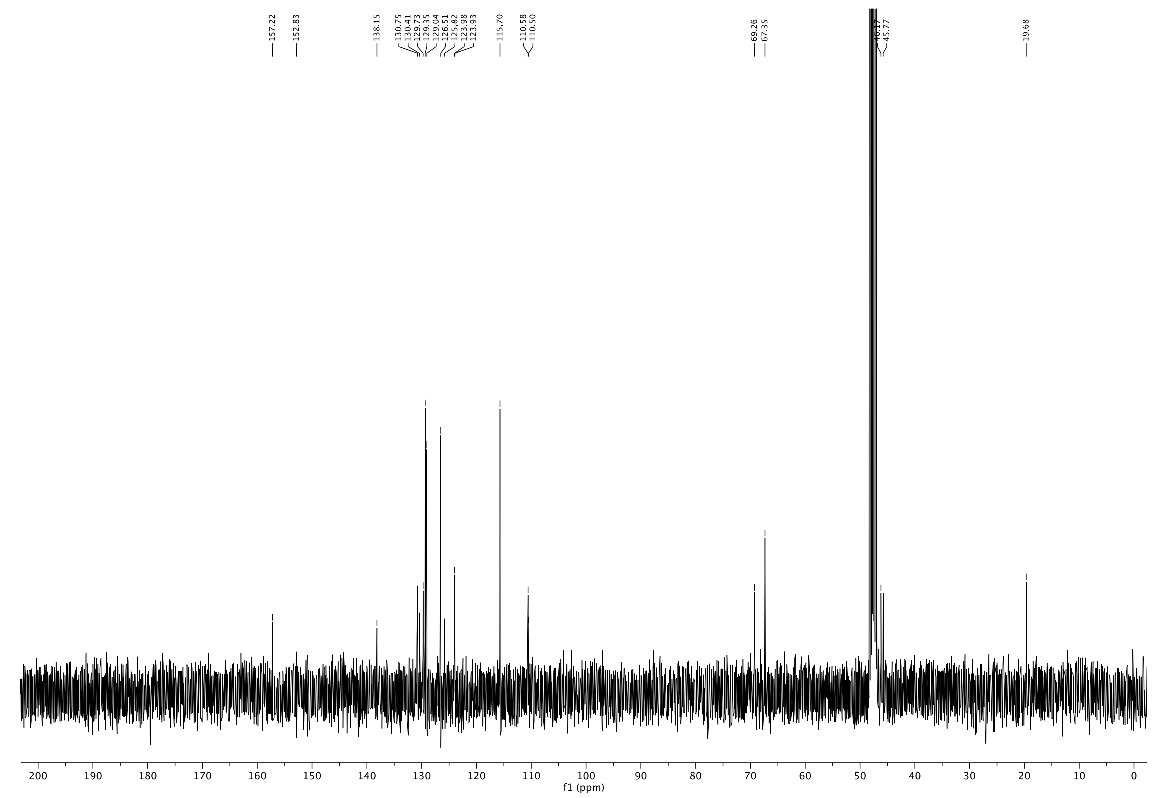

Figure S41. ^{13}C NMR spectrum (101 MHz, CD_3OD) of compound **53**.

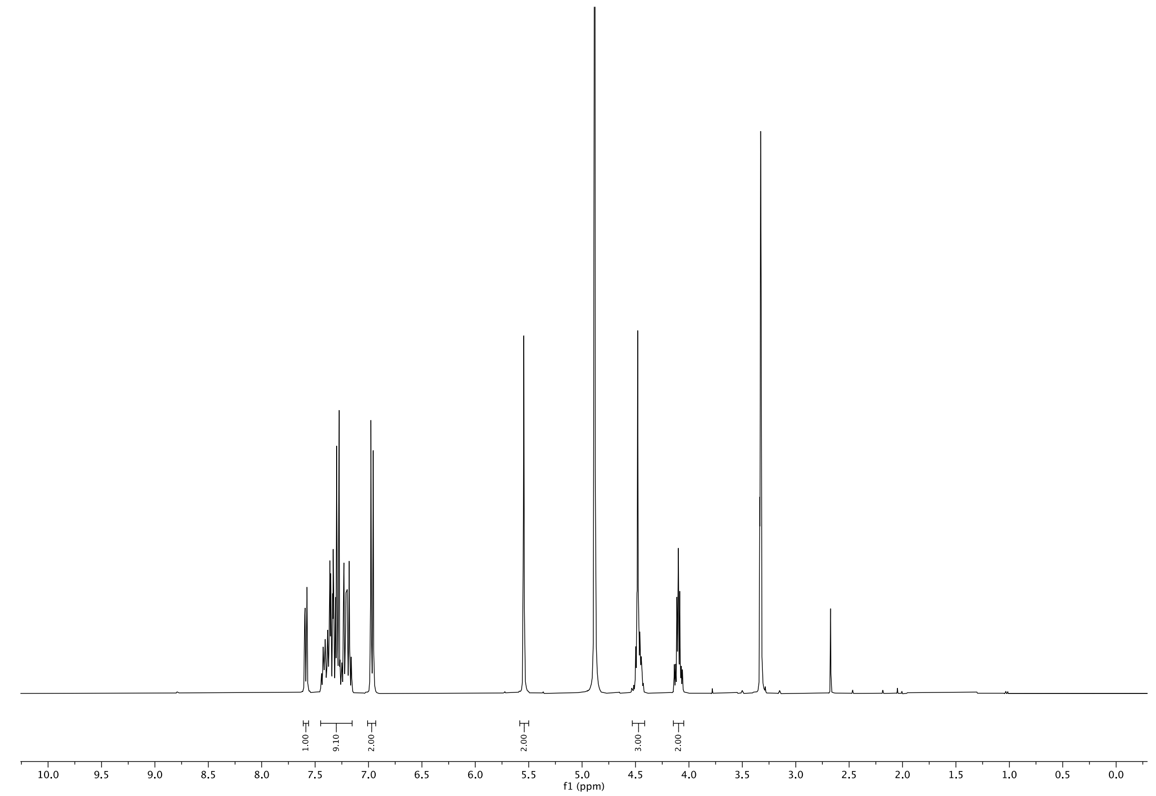

Figure S42. ^{1}H NMR spectrum (400 MHz, CD_3OD) of compound **54**.

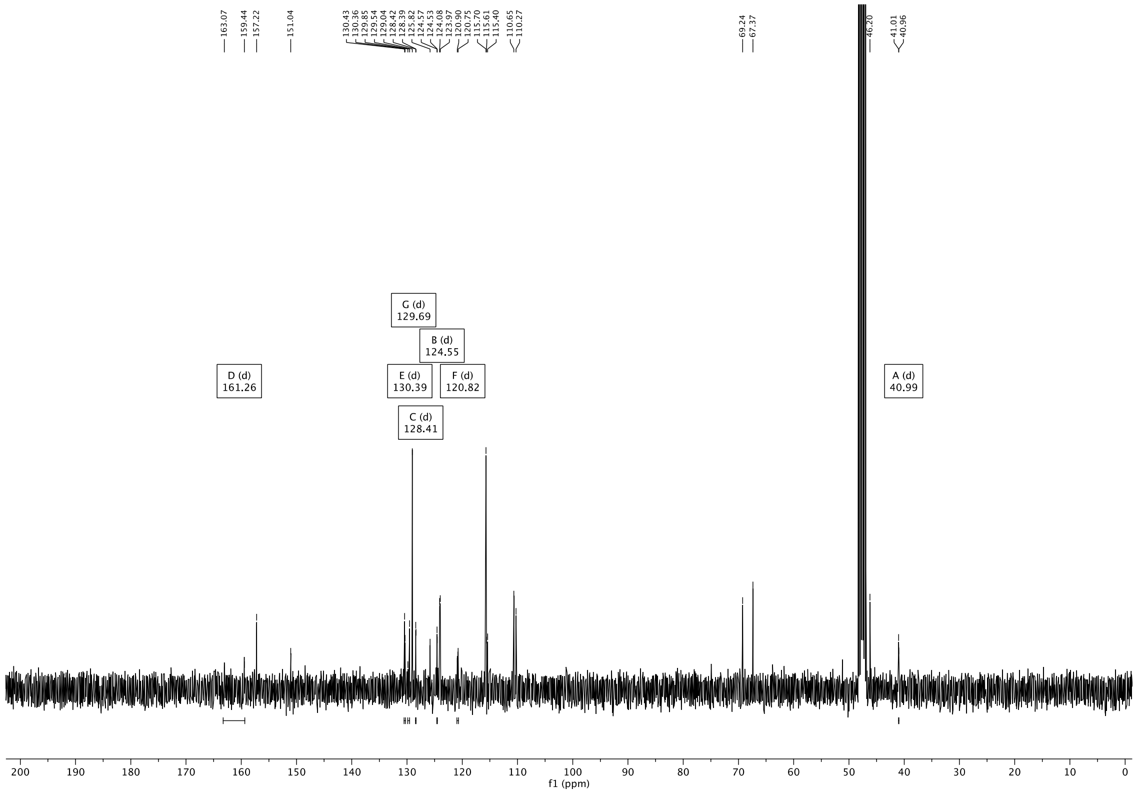

Figure S43. ^{13}C NMR spectrum (101 MHz, CD_3OD) of compound **54**.

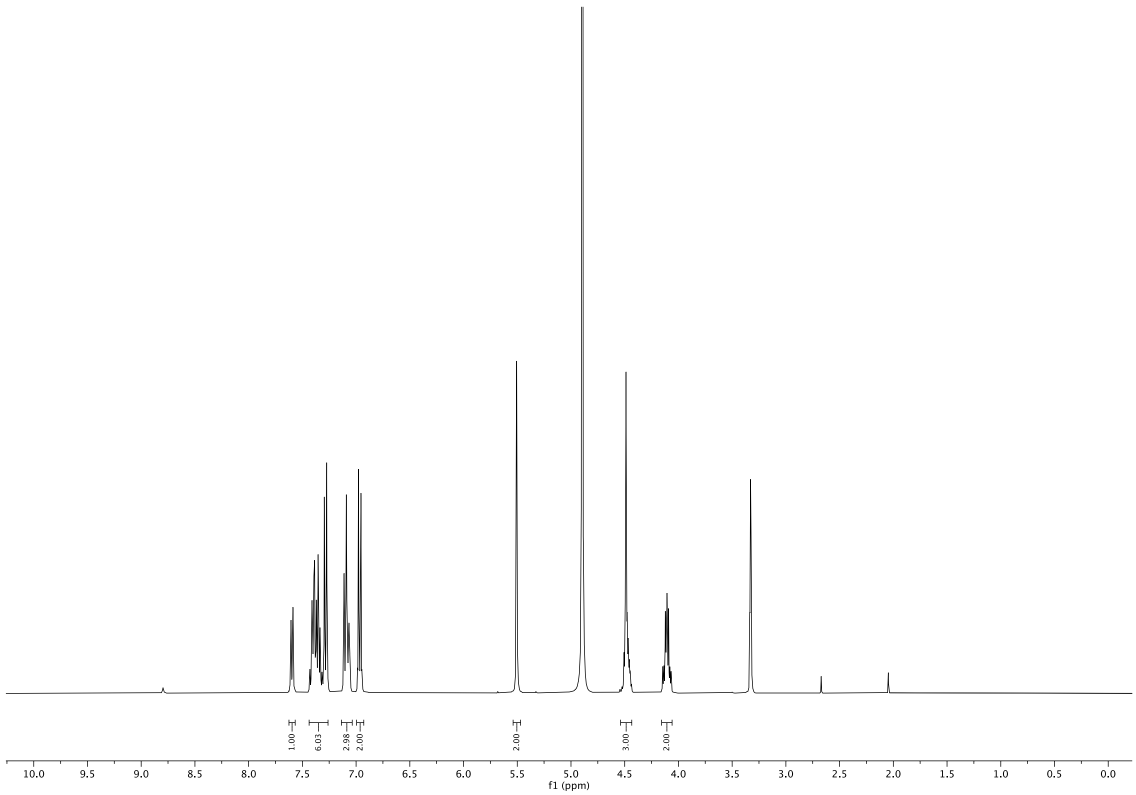

Figure S44. ^{1}H NMR spectrum (400 MHz, CD_3OD) of compound **55**.

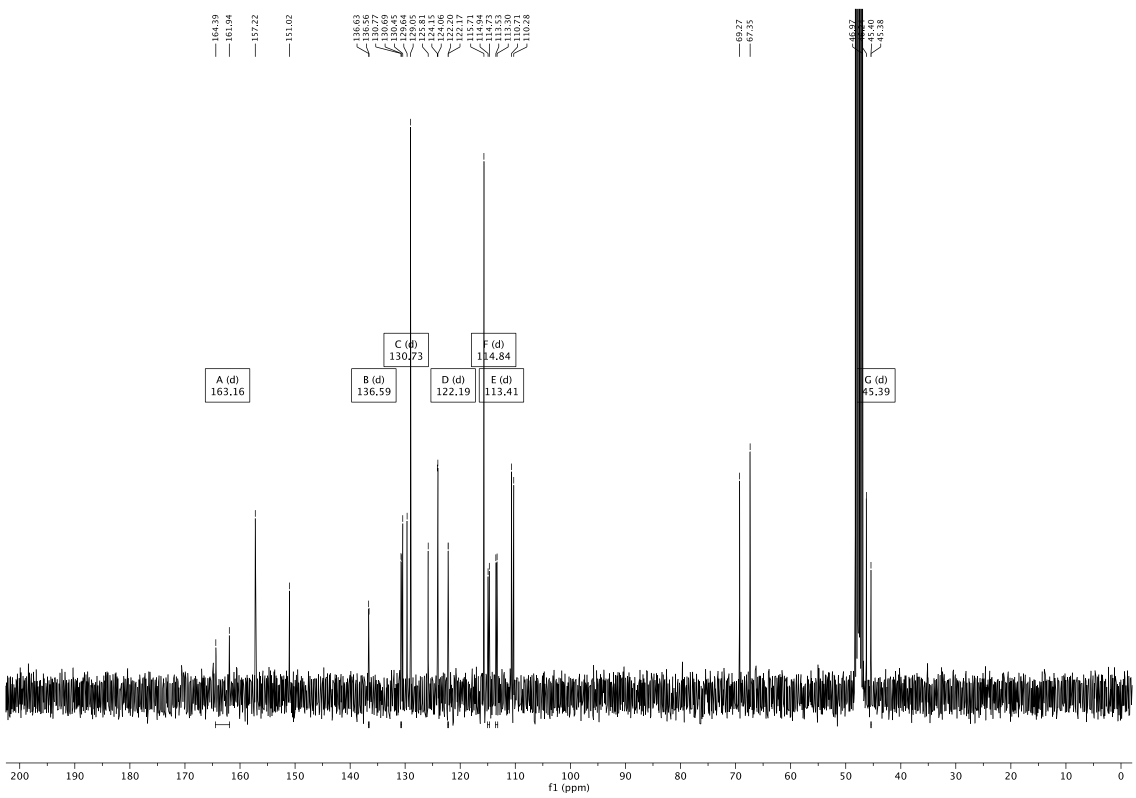

Figure S45. ^{13}C NMR spectrum (101 MHz, CD_3OD) of compound **55**.

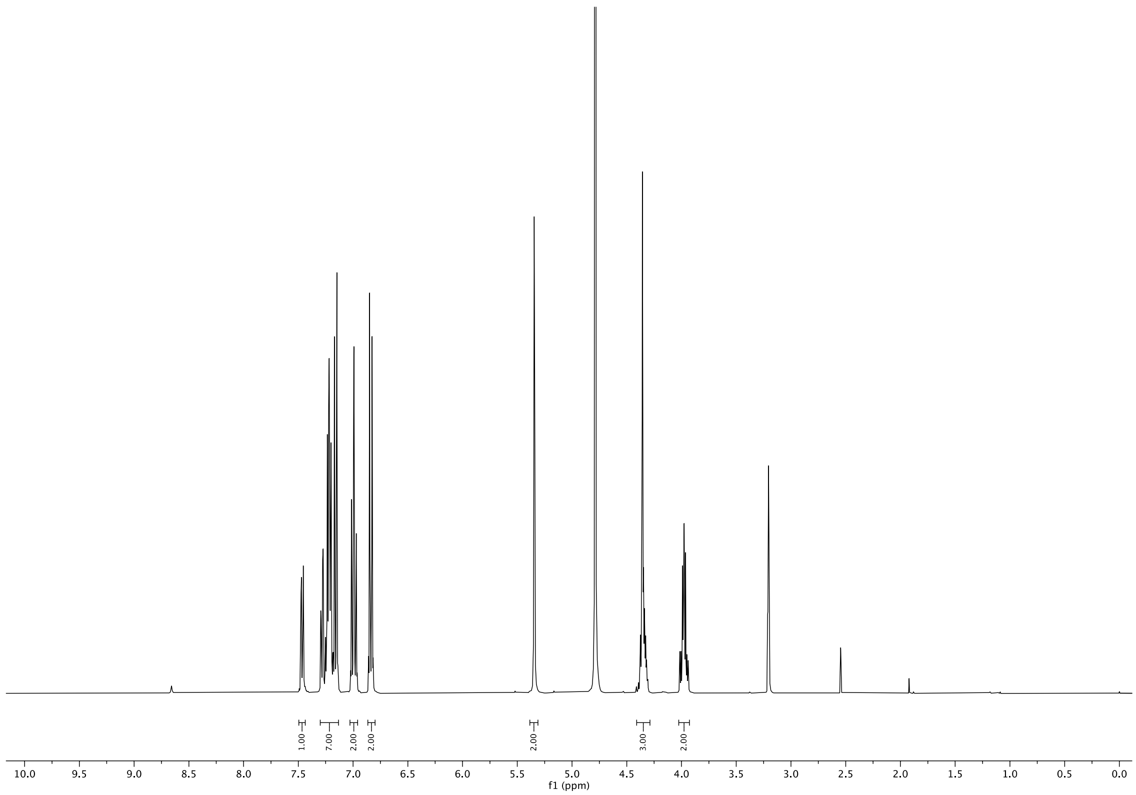

Figure S46. ^{1}H NMR spectrum (400 MHz, CD_3OD) of compound **56**.

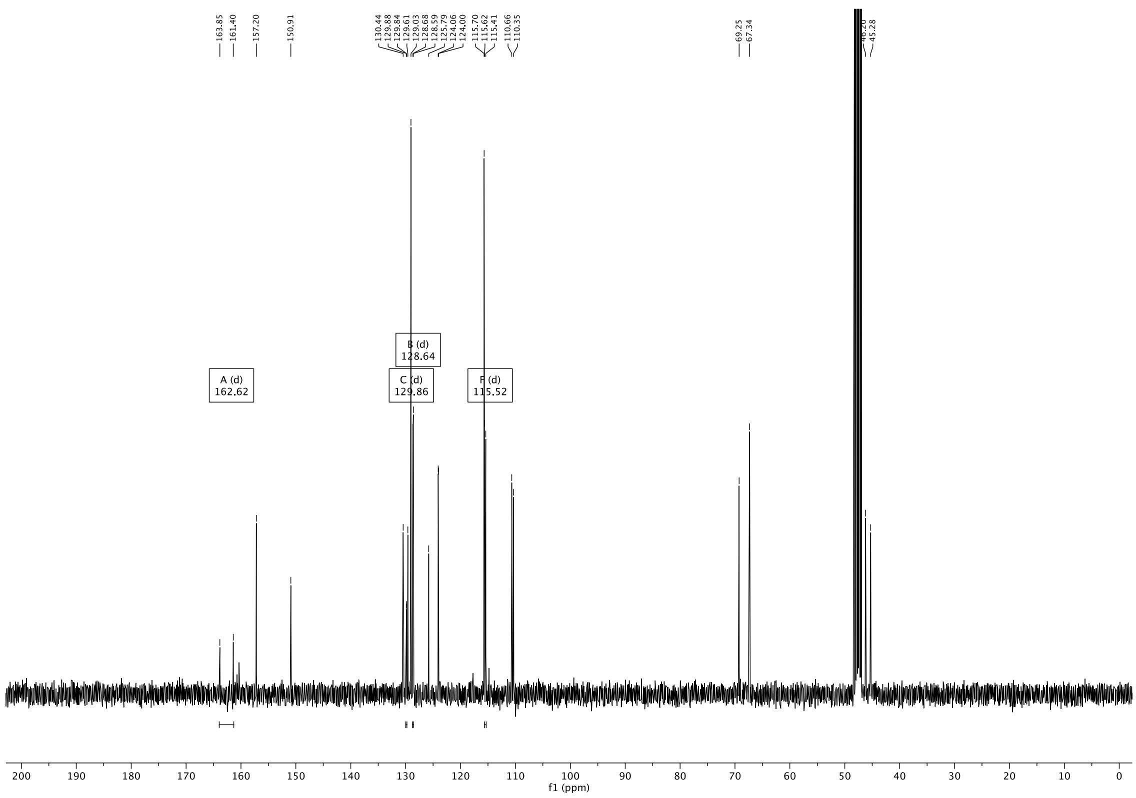

Figure S47. ^{13}C NMR spectrum (101 MHz, CD_3OD) of compound **56**.

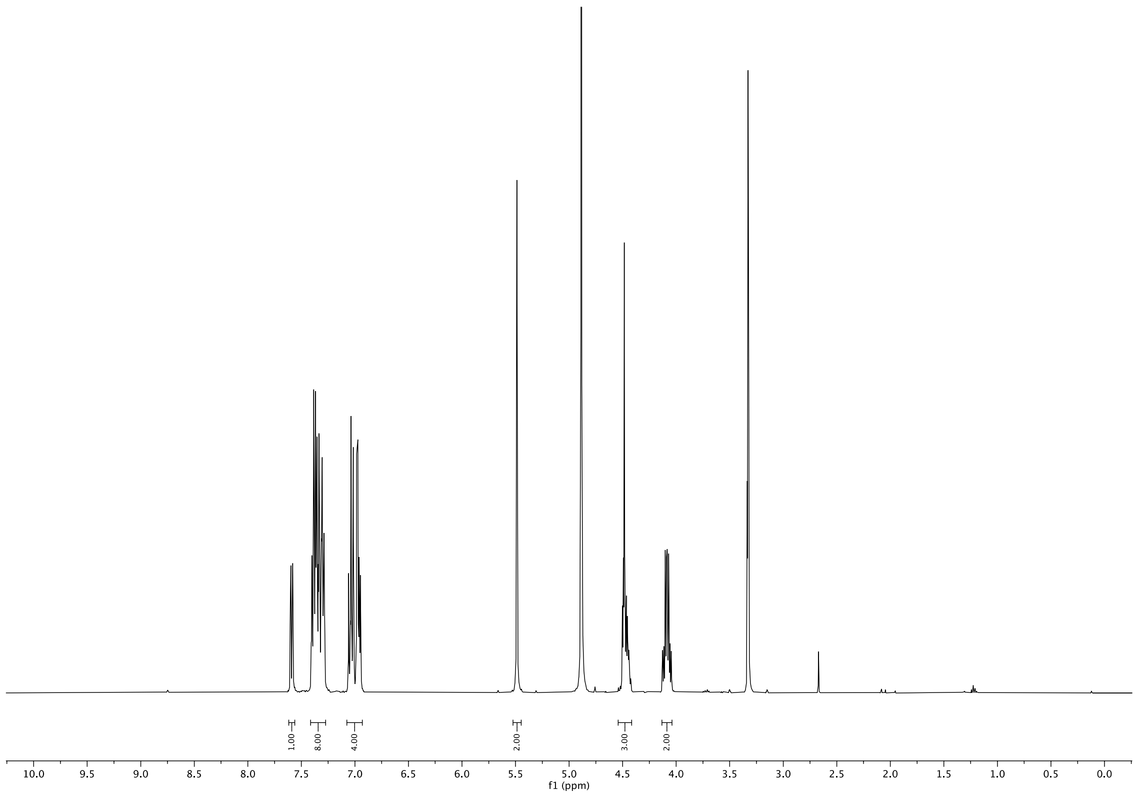

Figure S48. ^{1}H NMR spectrum (400 MHz, CD_3OD) of compound **57**.

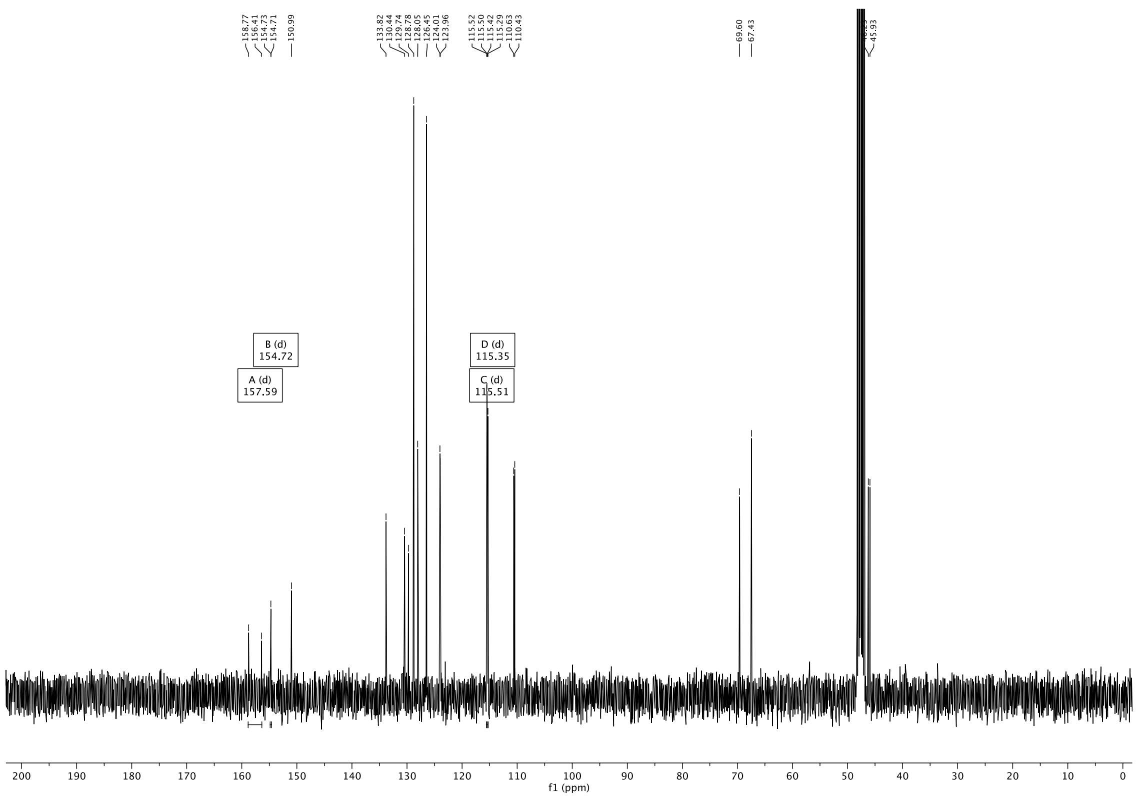

Figure S49. ^{13}C NMR spectrum (101 MHz, CD_3OD) of compound **57**.

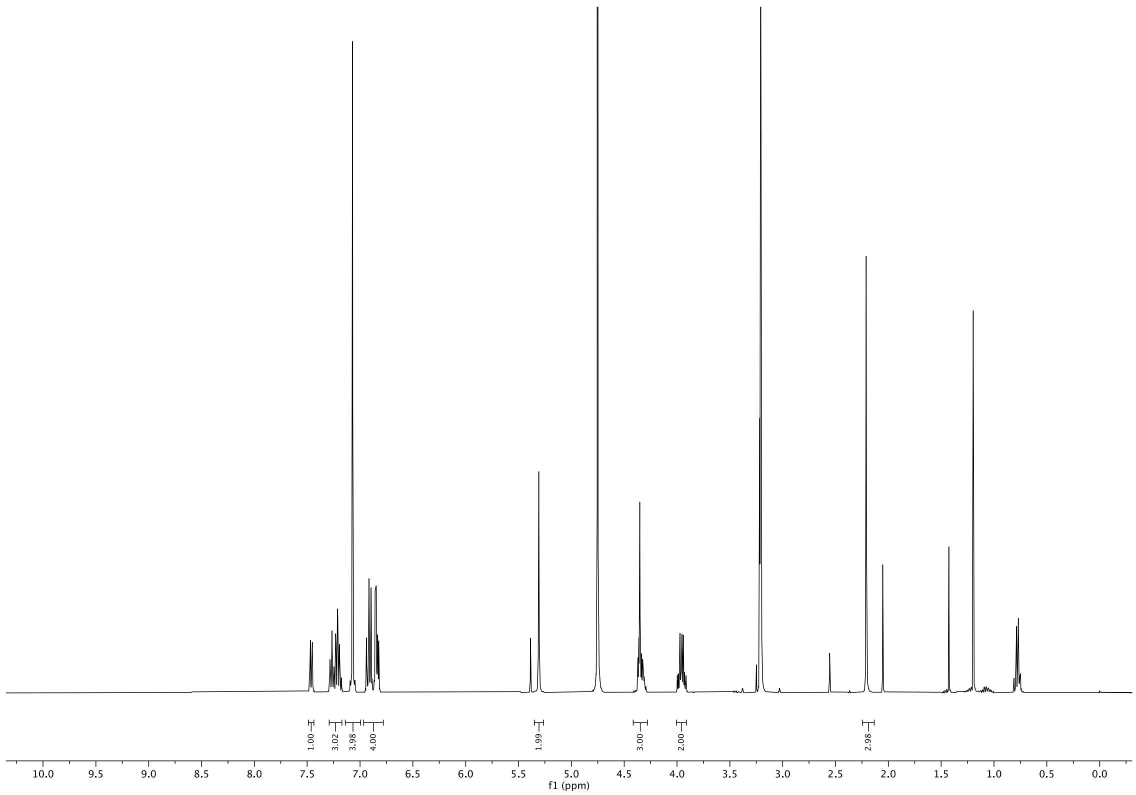

Figure S50. ^{1}H NMR spectrum (400 MHz, CD_3OD) of compound **58**.

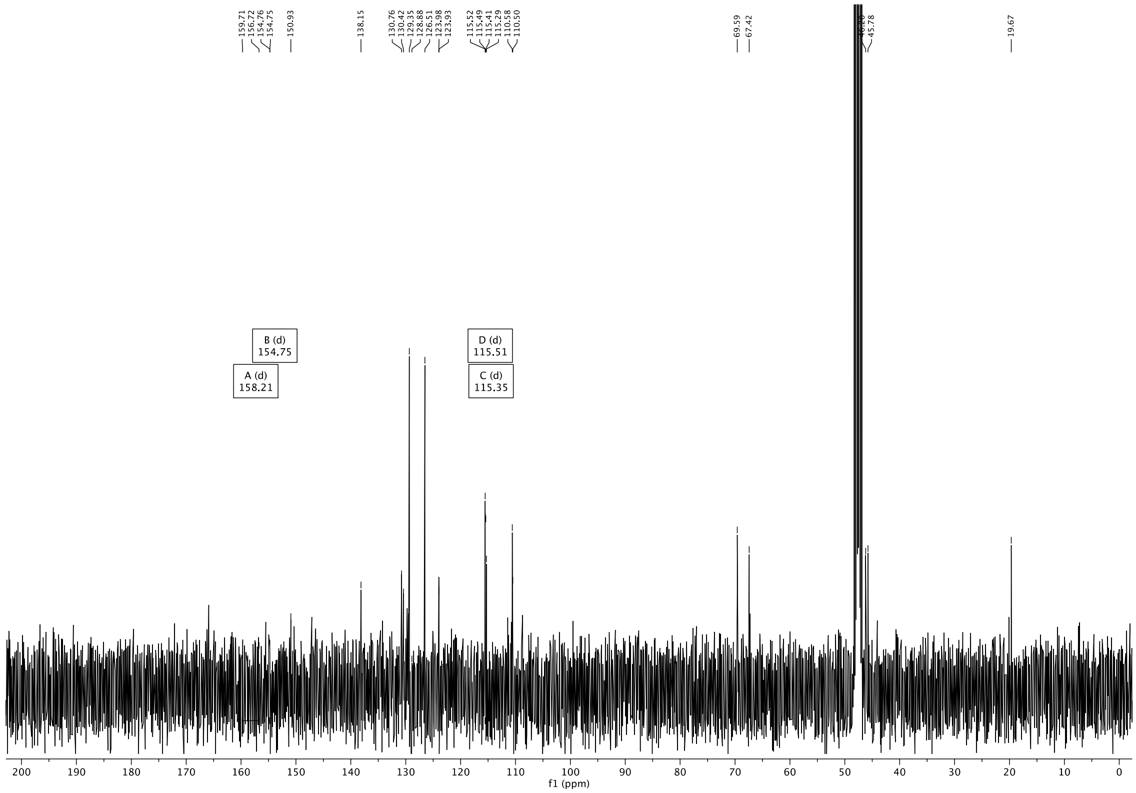

Figure S51. ^{13}C NMR spectrum (101 MHz, CD_3OD) of compound **58**.

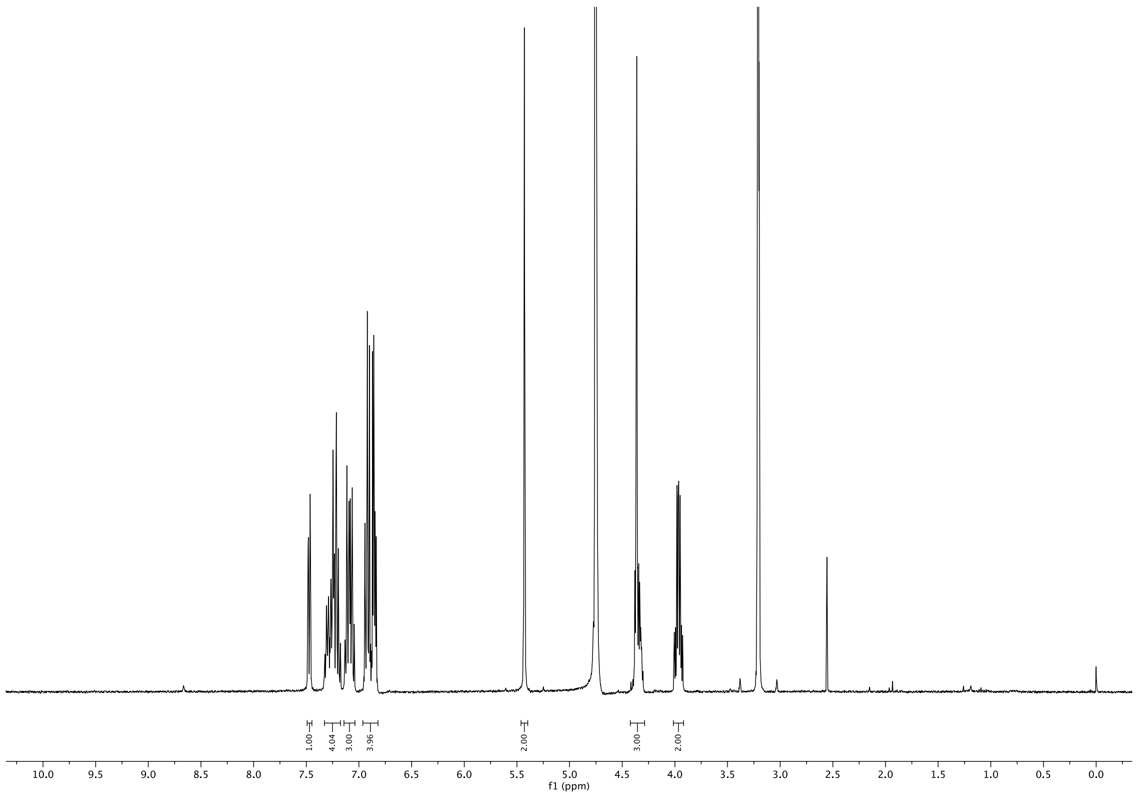

Figure S52. ^{1}H NMR spectrum (400 MHz, CD_3OD) of compound **59**.

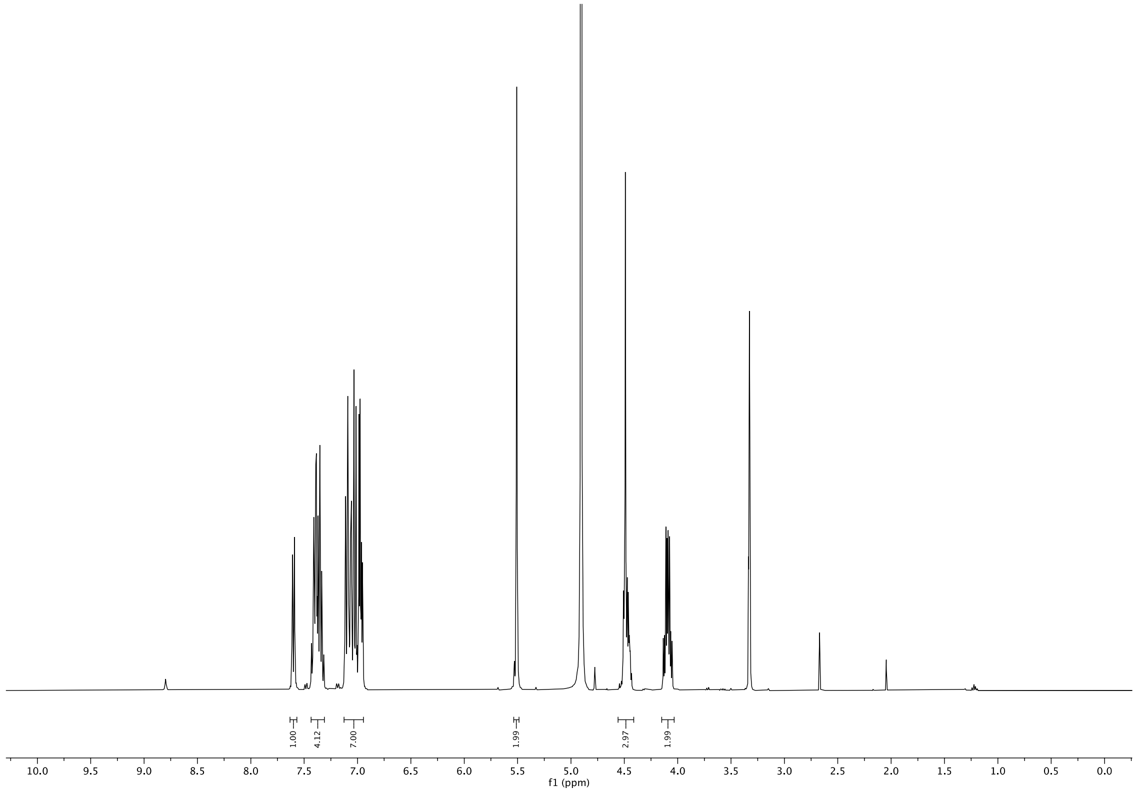

Figure S53. ^{1}H NMR spectrum (400 MHz, CD_3OD) of compound **60**.

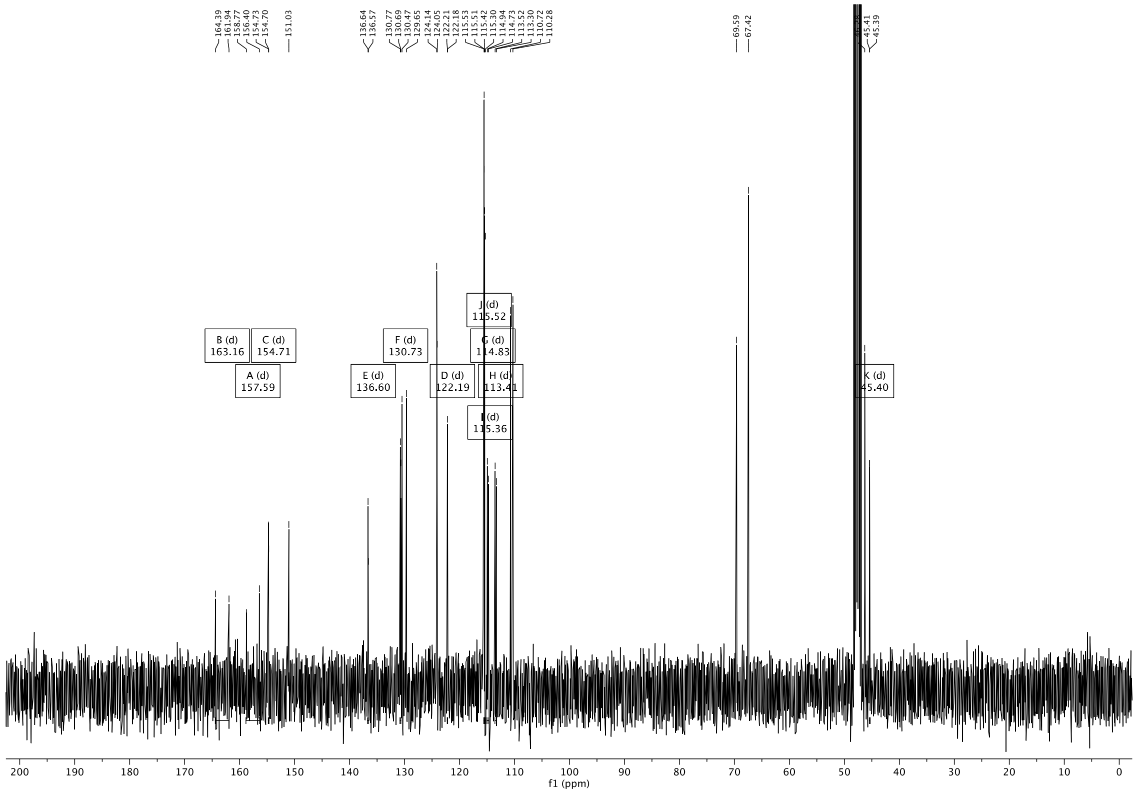

Figure S54. ^{13}C NMR spectrum (101 MHz, CD_3OD) of compound **60**.

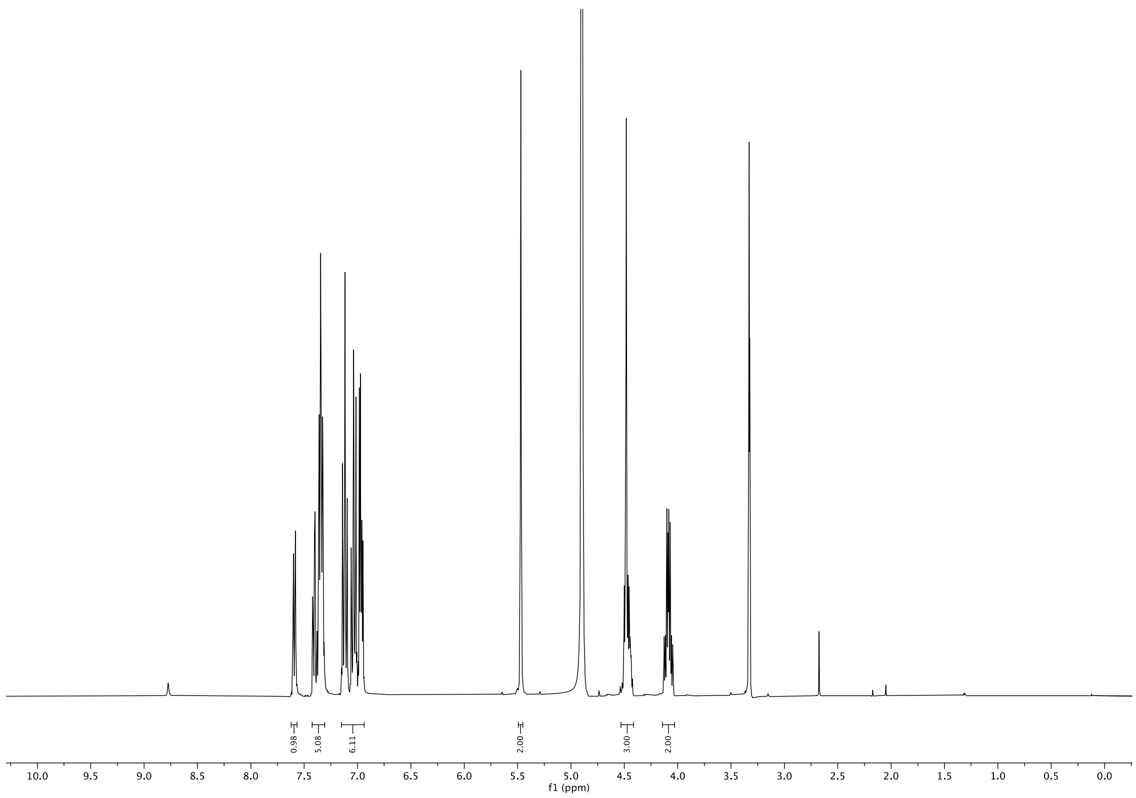

Figure S55. ^{1}H NMR spectrum (400 MHz, CD_3OD) of compound **61**.

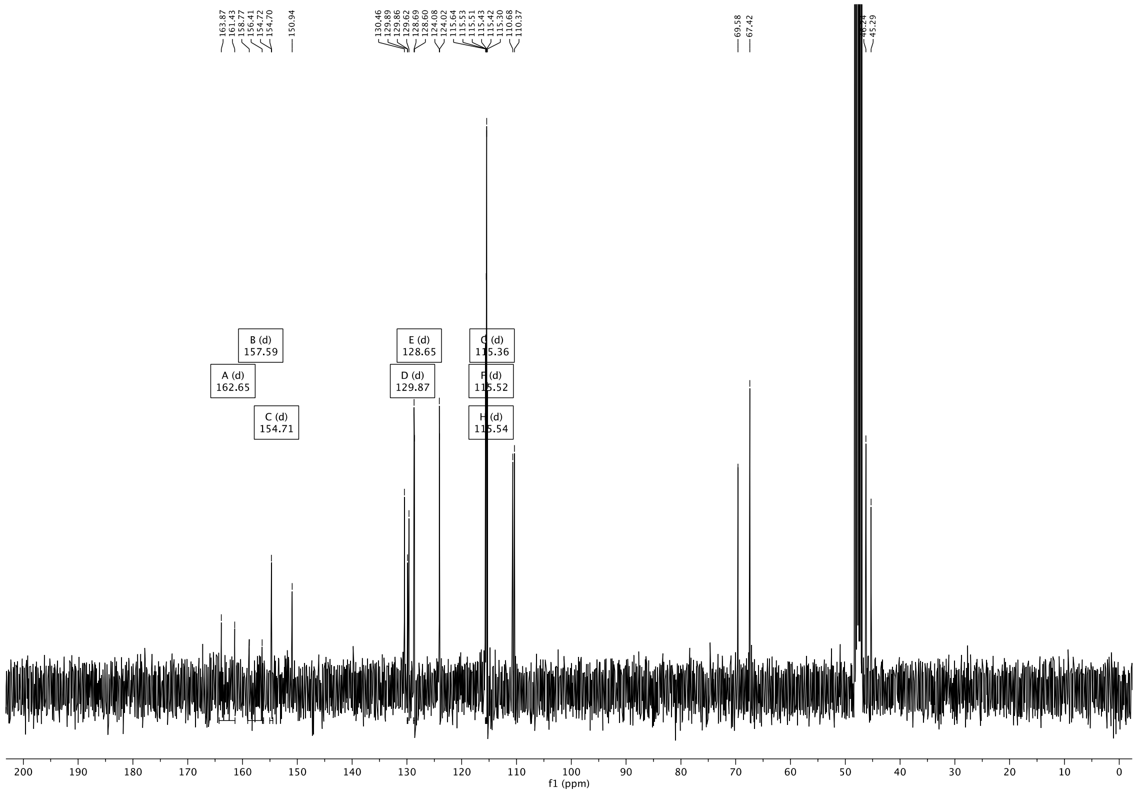

Figure S56. ^{13}C NMR spectrum (101 MHz, CD_3OD) of compound **61**.

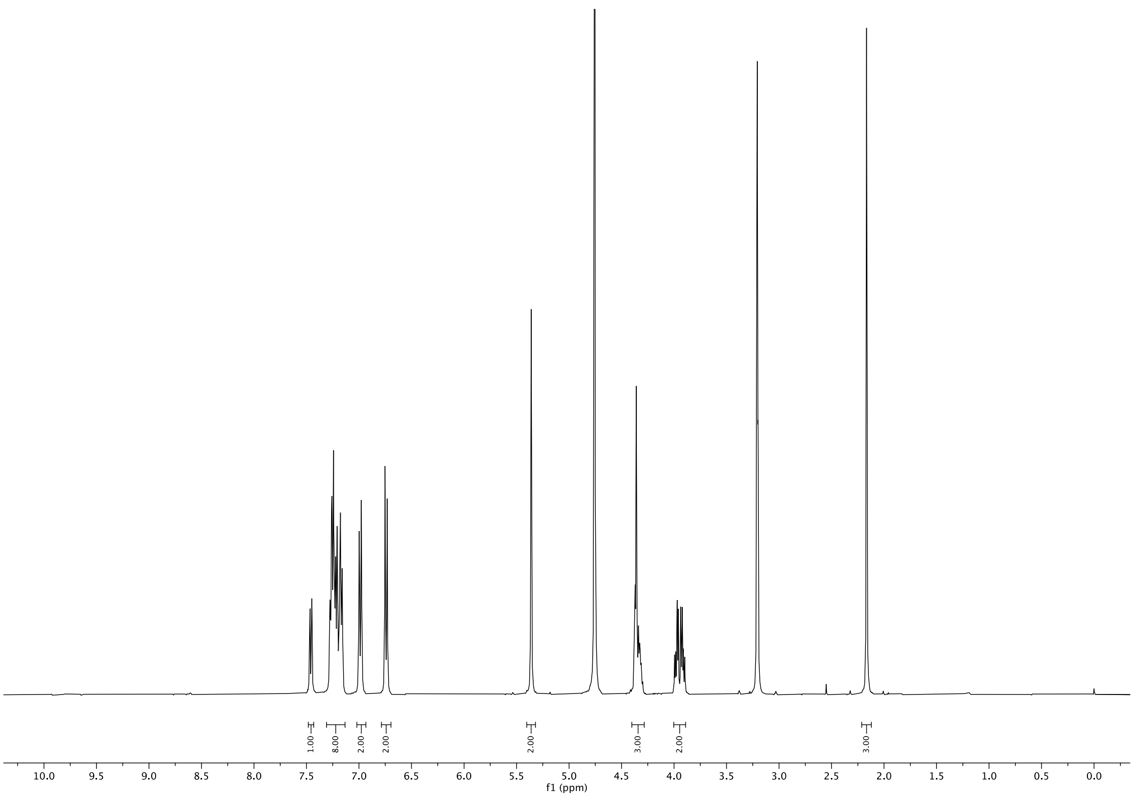

Figure S57. ^{1}H NMR spectrum (400 MHz, CD_3OD) of compound **62**.

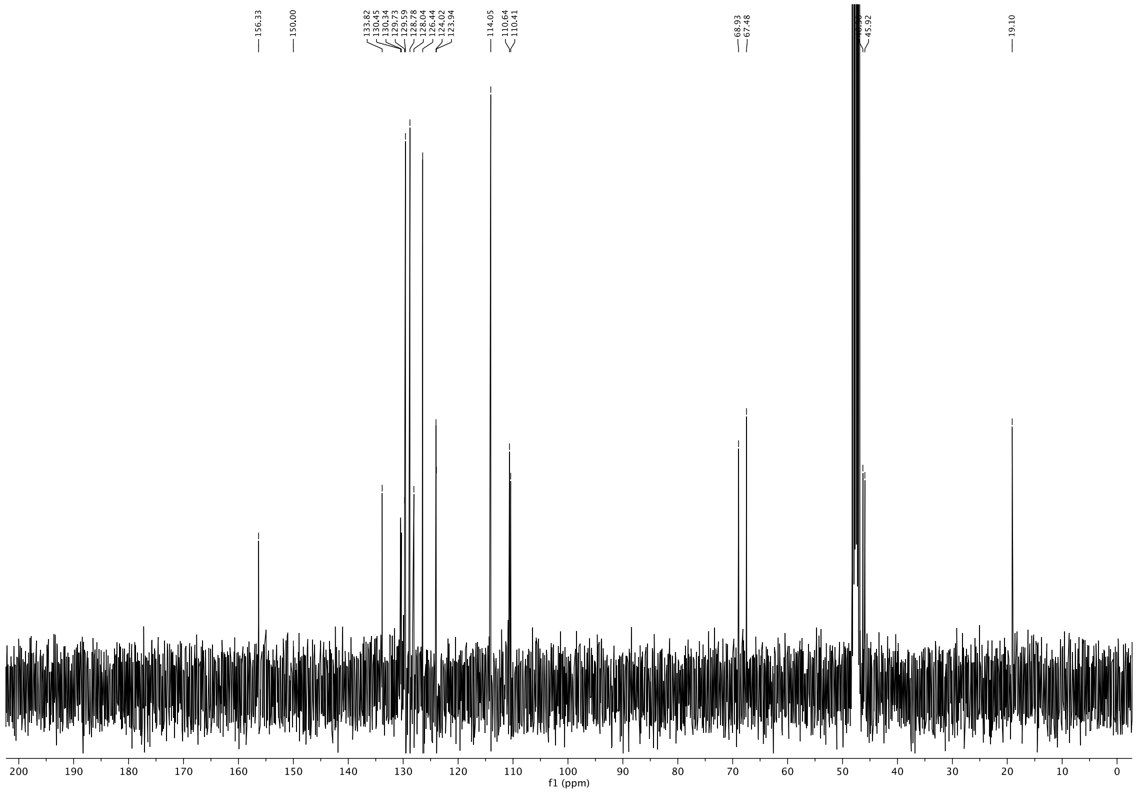

Figure S58. ^{13}C NMR spectrum (101 MHz, CD_3OD) of compound **62**.

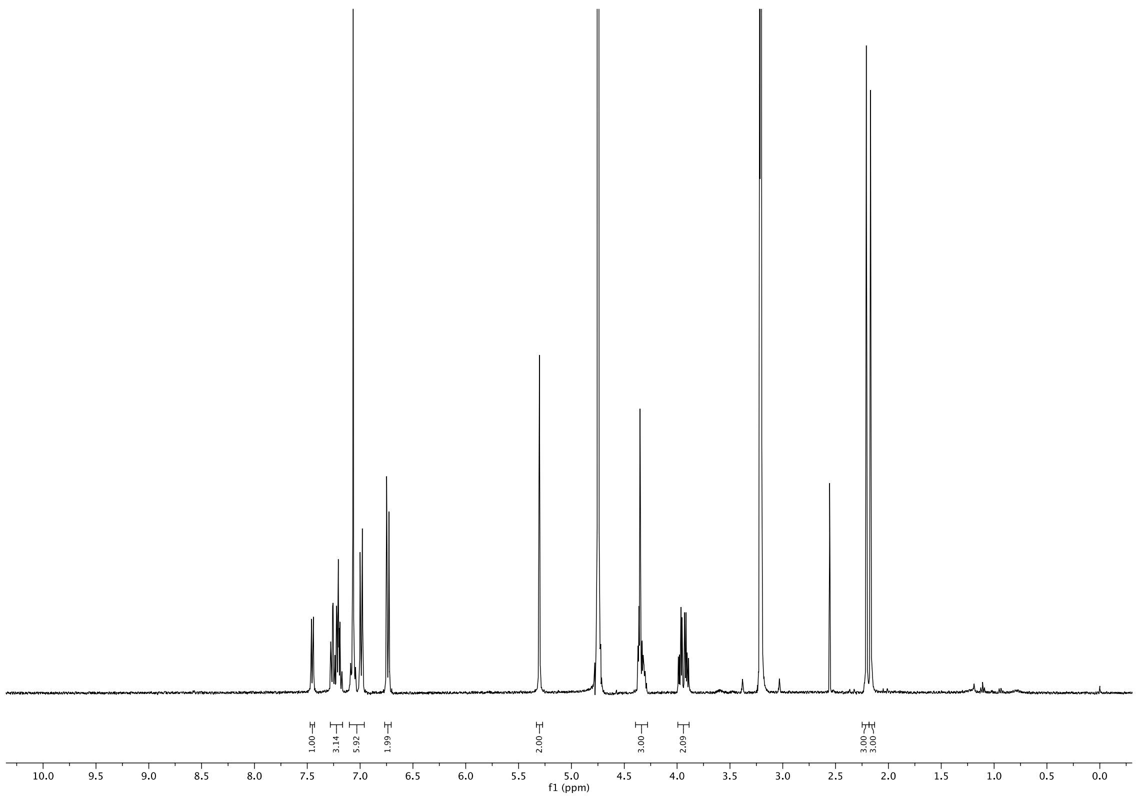

Figure S59. -2¹H NMR spectrum (400 MHz, CD_3OD) of compound **63**.

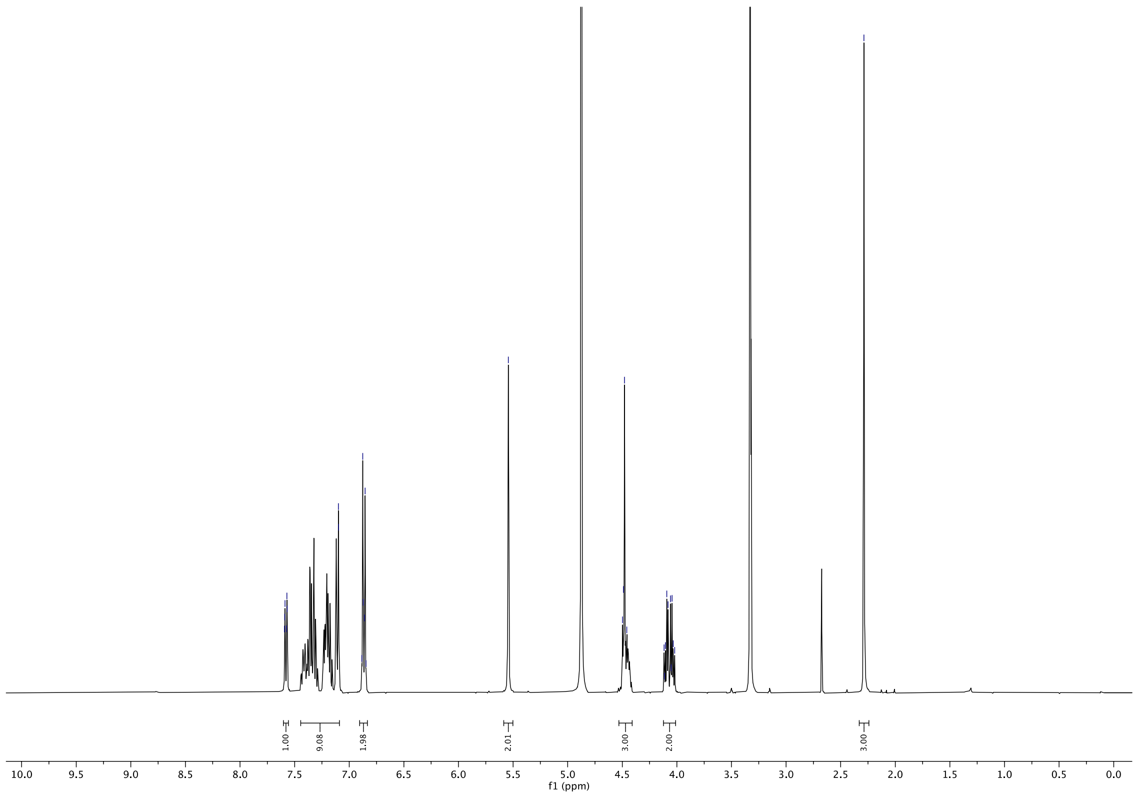

Figure S60. ¹H NMR spectrum (400 MHz, CD_3OD) of compound **64**.

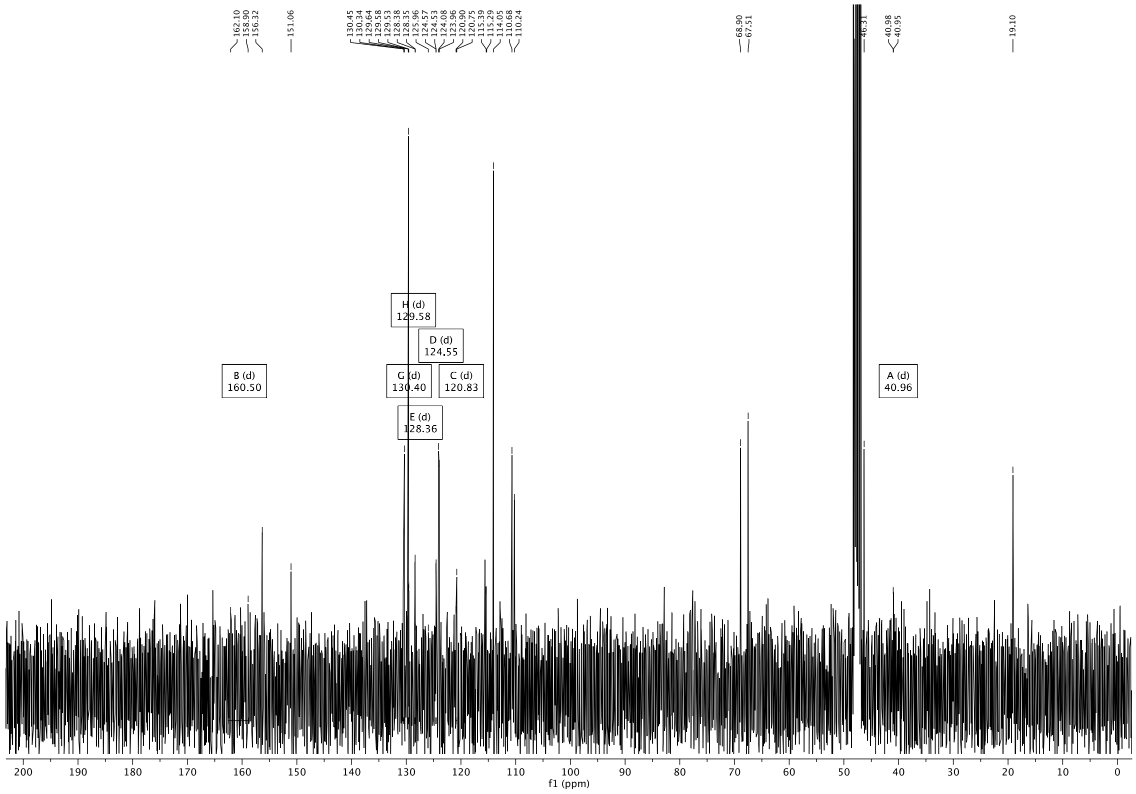

Figure S61. ^{13}C NMR spectrum (101 MHz, CD_3OD) of compound **64**.

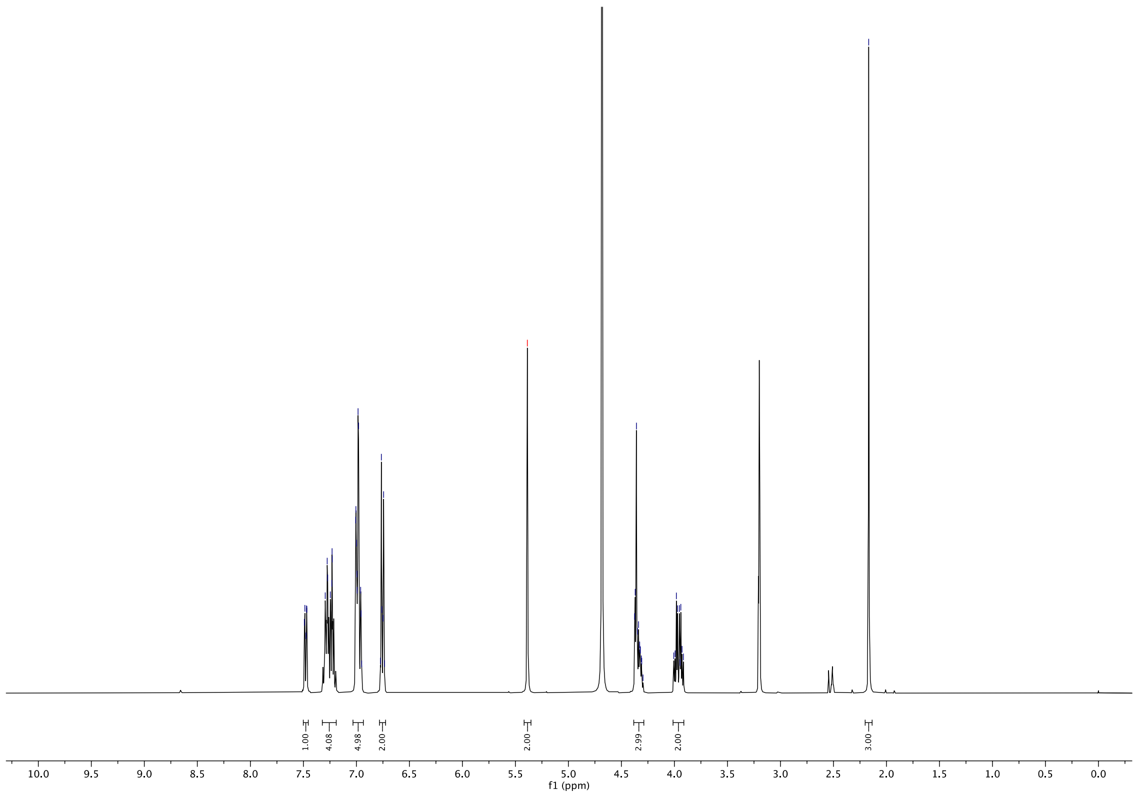

Figure S62. ^{1}H NMR spectrum (400 MHz, CD_3OD) of compound **65**.

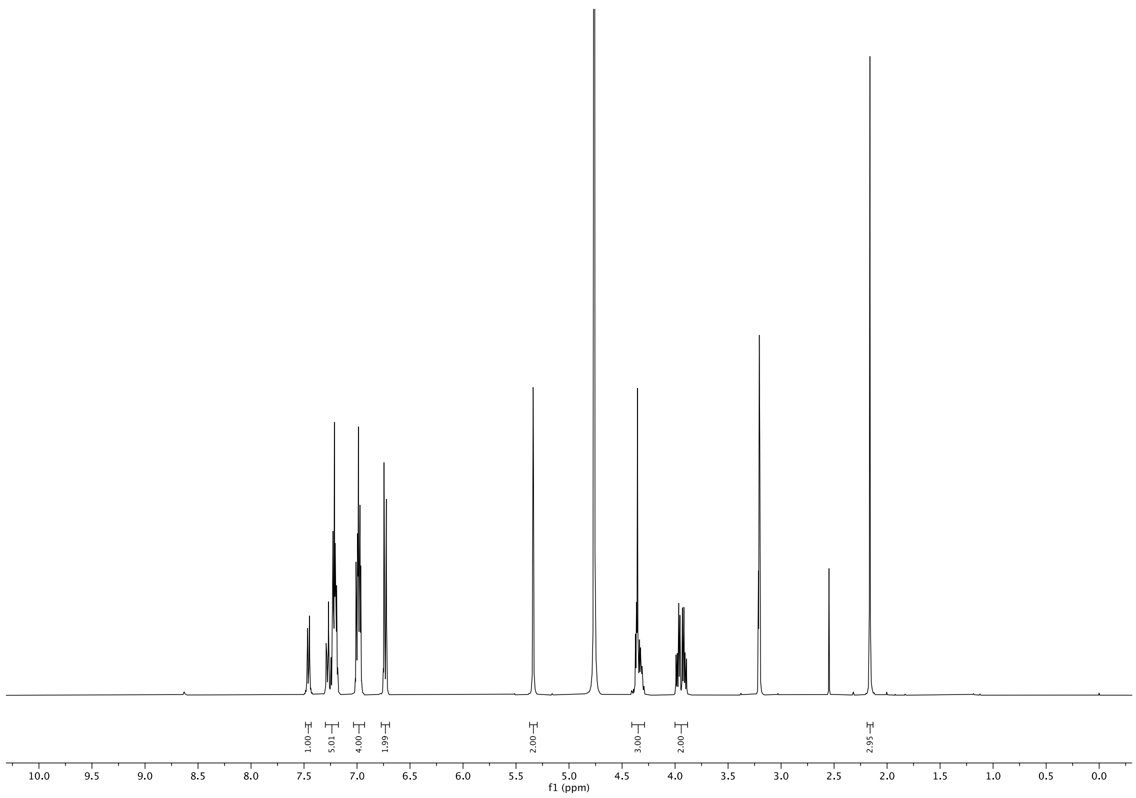

Figure S63. ^{1}H NMR spectrum (400 MHz, CD_3OD) of compound **66**.

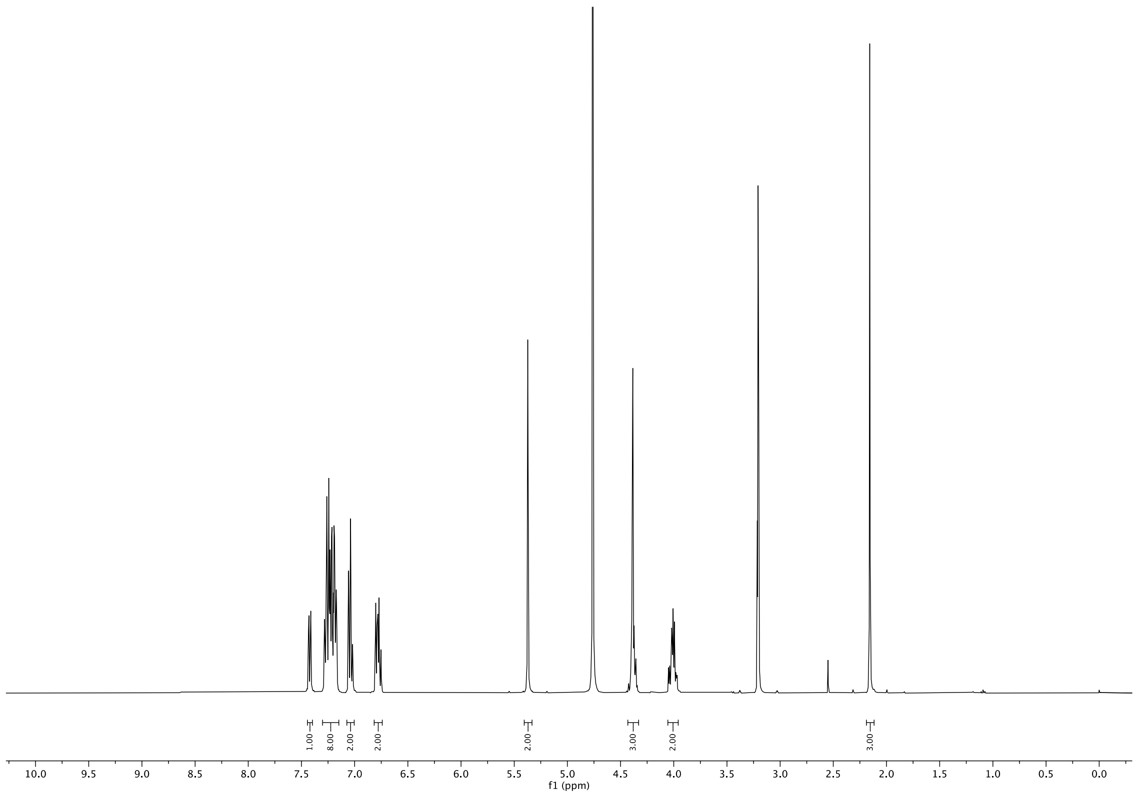

Figure S64. ^{1}H NMR spectrum (400 MHz, CD_3OD) of compound **67**.

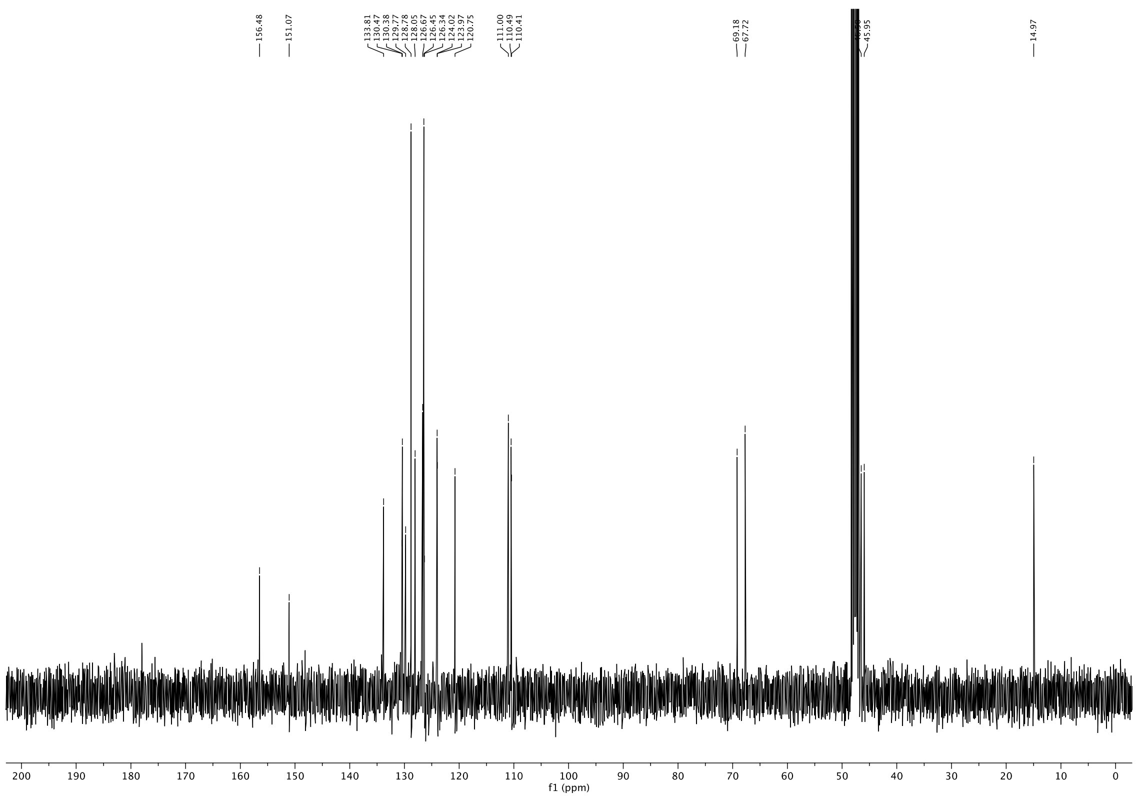

Figure S65. ^{13}C NMR spectrum (101 MHz, CD_3OD) of compound **67**.

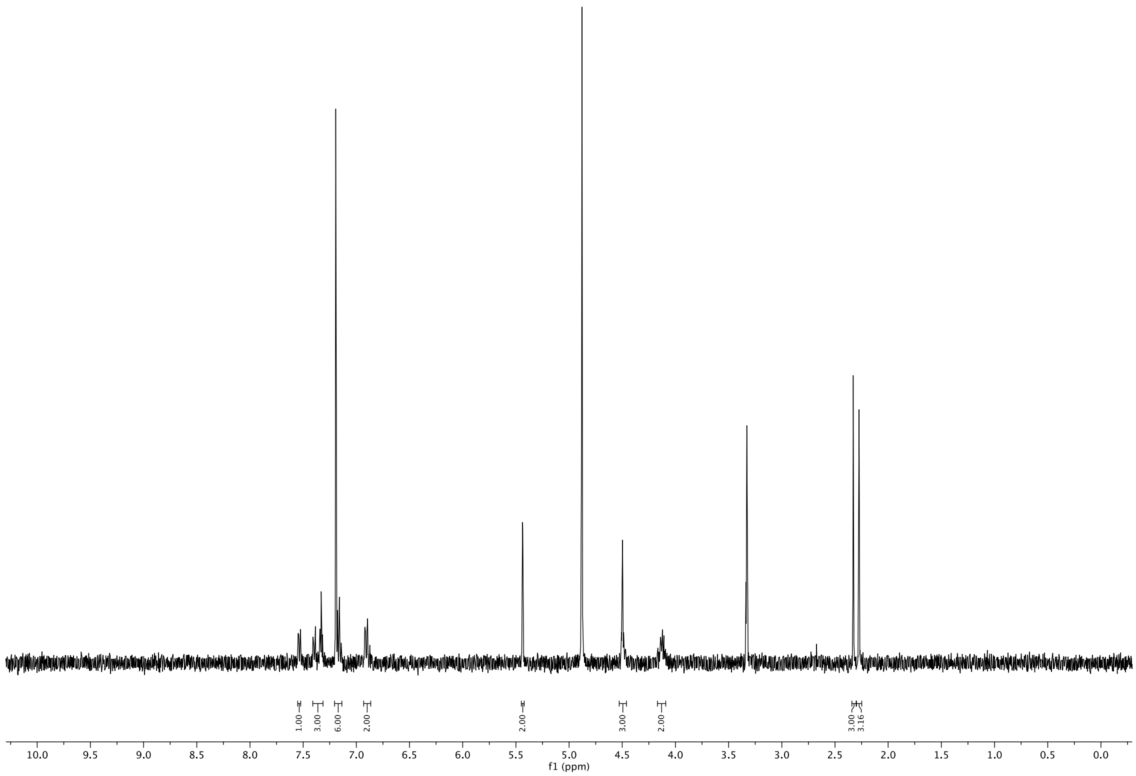

Figure S66. ^{1}H NMR spectrum (400 MHz, CD_3OD) of compound **68**.

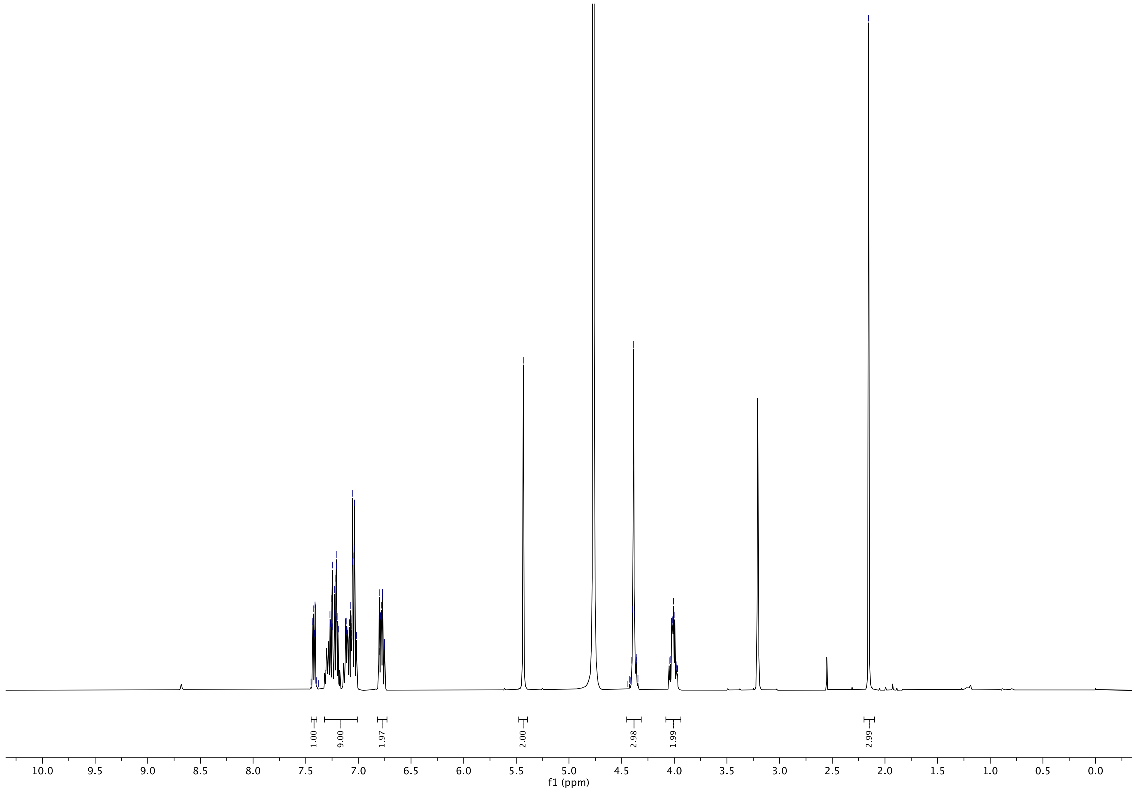

Figure S67. ¹H NMR spectrum (400 MHz, CD_3OD) of compound **69**.

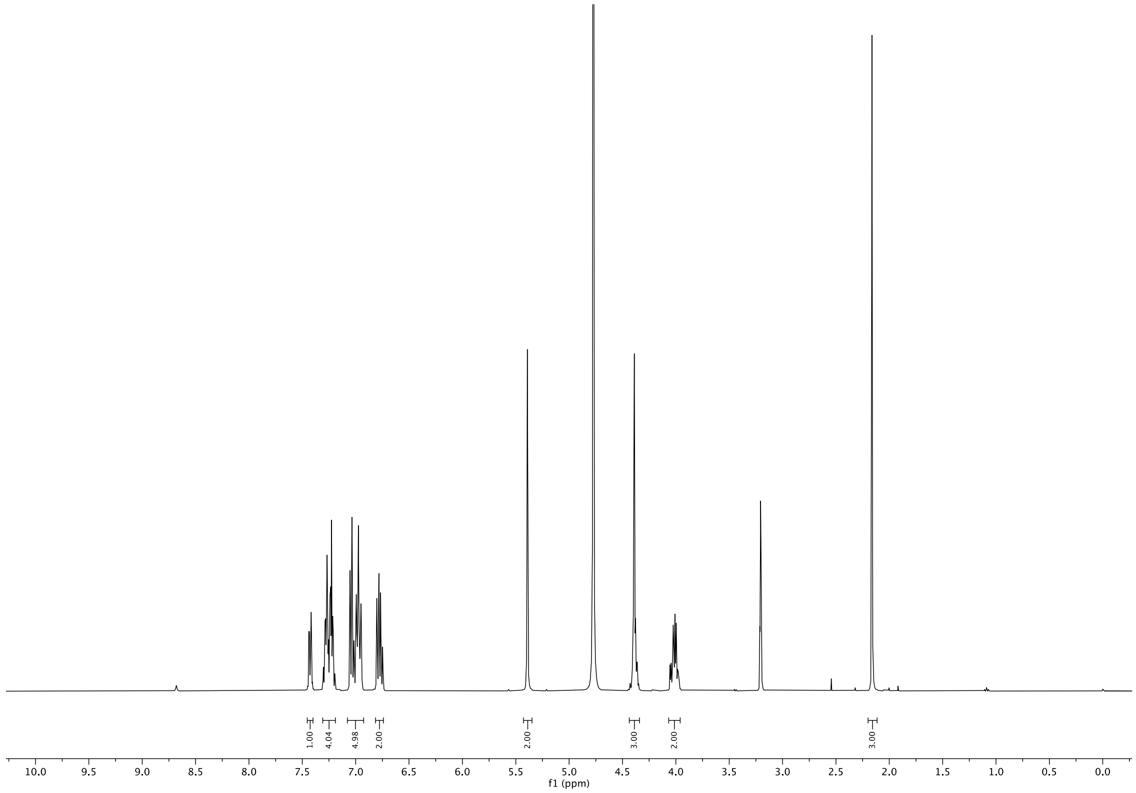

Figure S68. ¹H NMR spectrum (400 MHz, CD_3OD) of compound **70**.

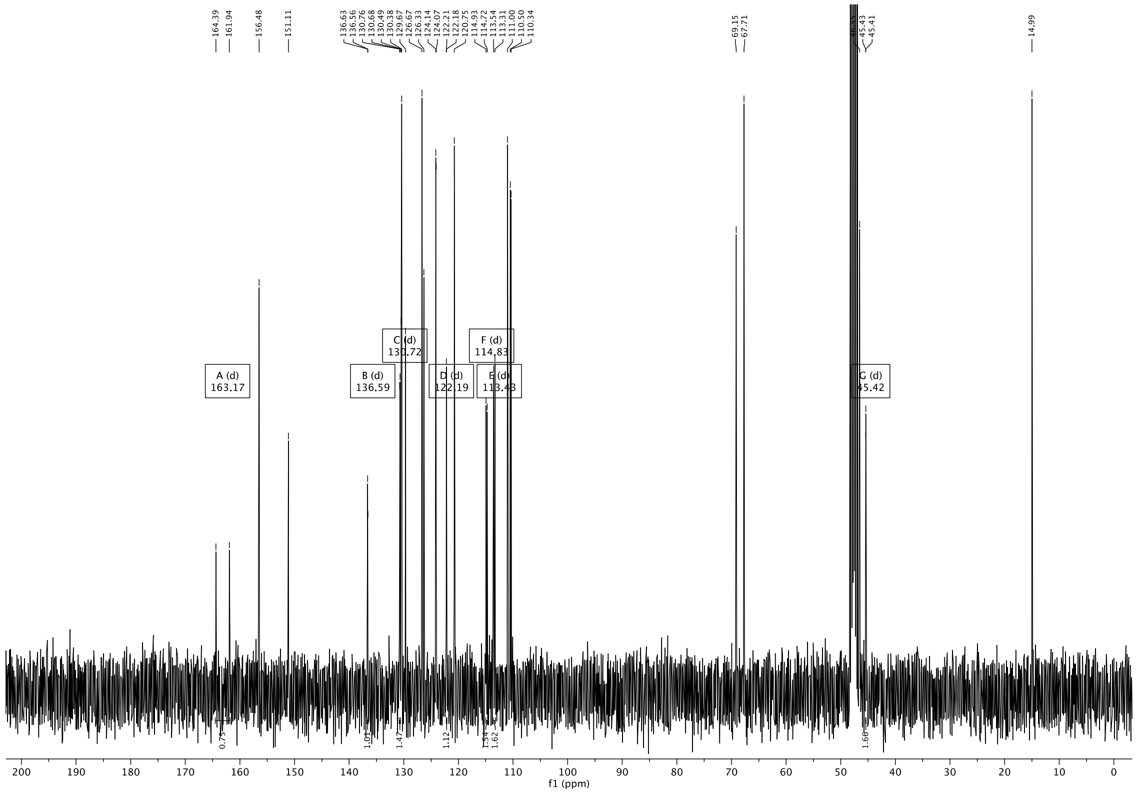

Figure S69. ^{13}C NMR spectrum (101 MHz, CD_3OD) of compound **70**.

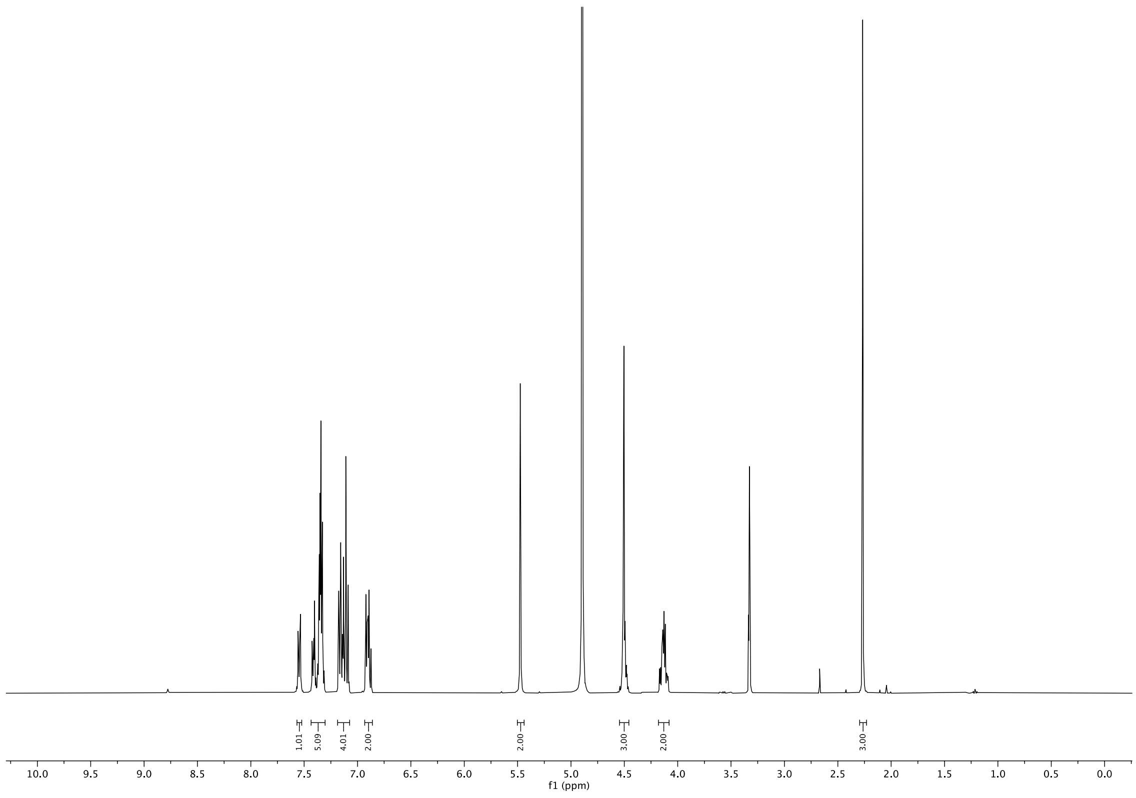

Figure S70. ^{1}H NMR spectrum (400 MHz, CD_3OD) of compound **71**.

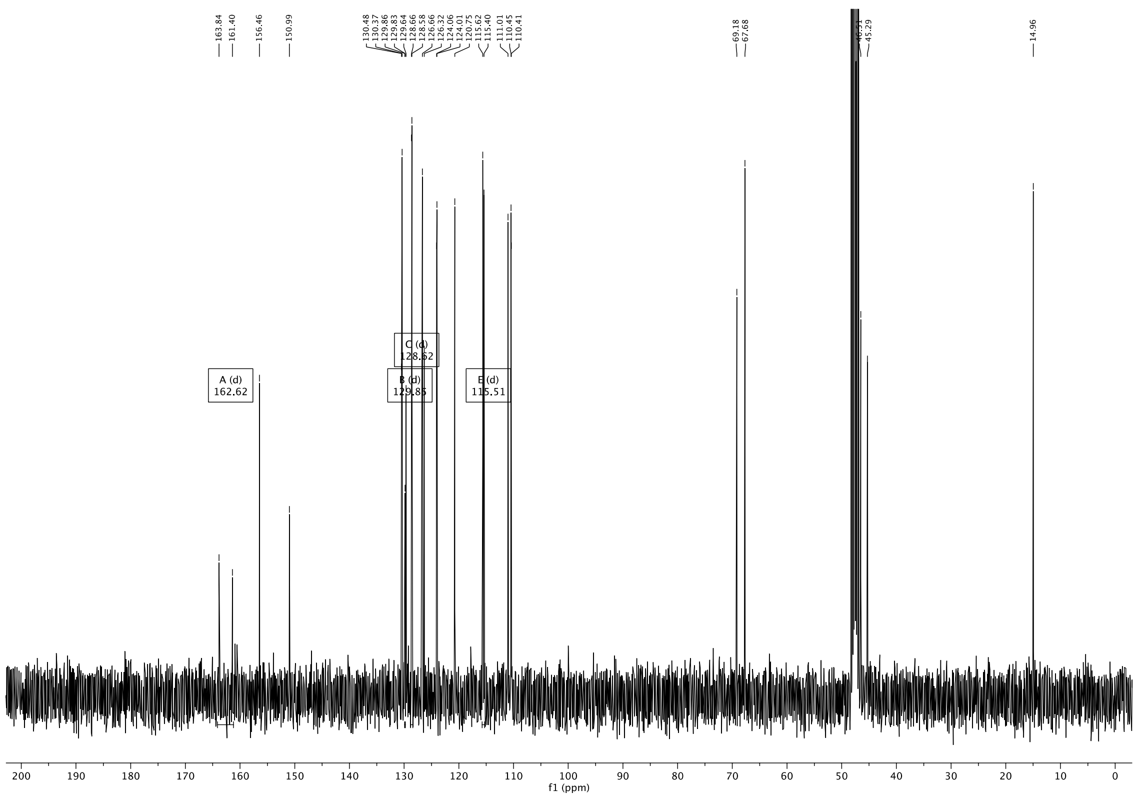

Figure S71. ^{13}C NMR spectrum (101 MHz, CD_3OD) of compound **71**.

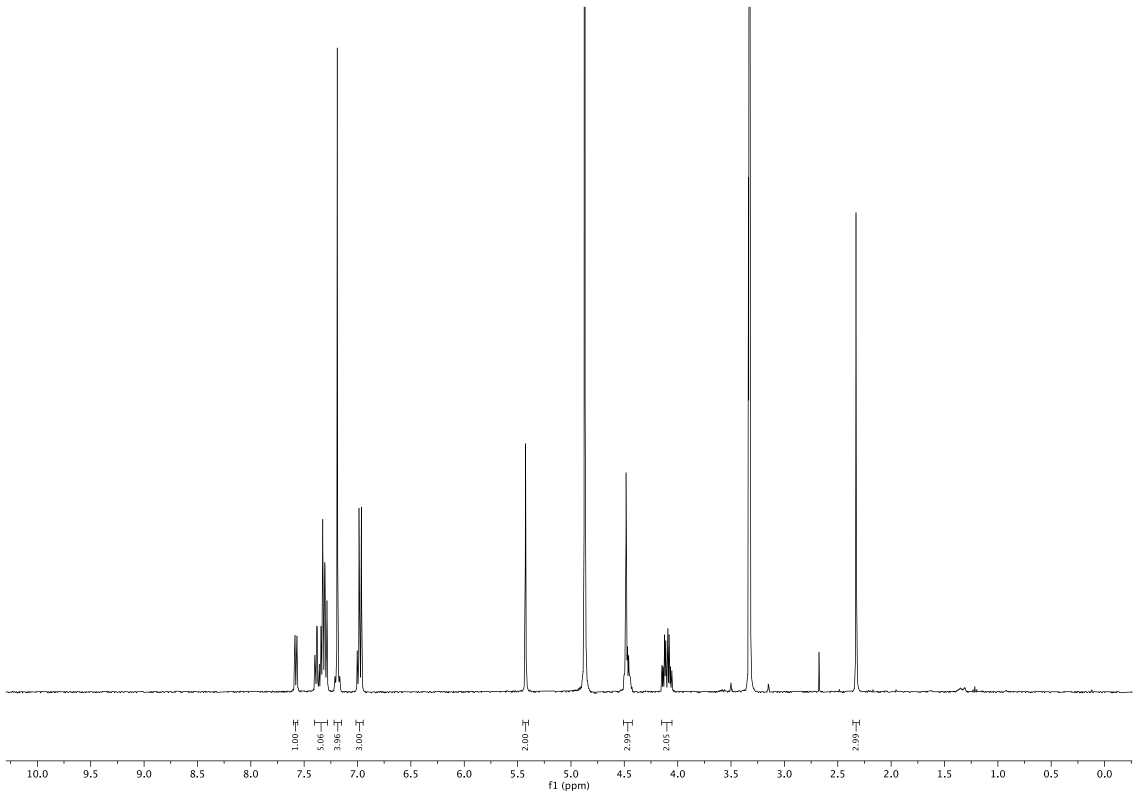

Figure S72. ^{1}H NMR spectrum (400 MHz, CD_3OD) of compound **72**.

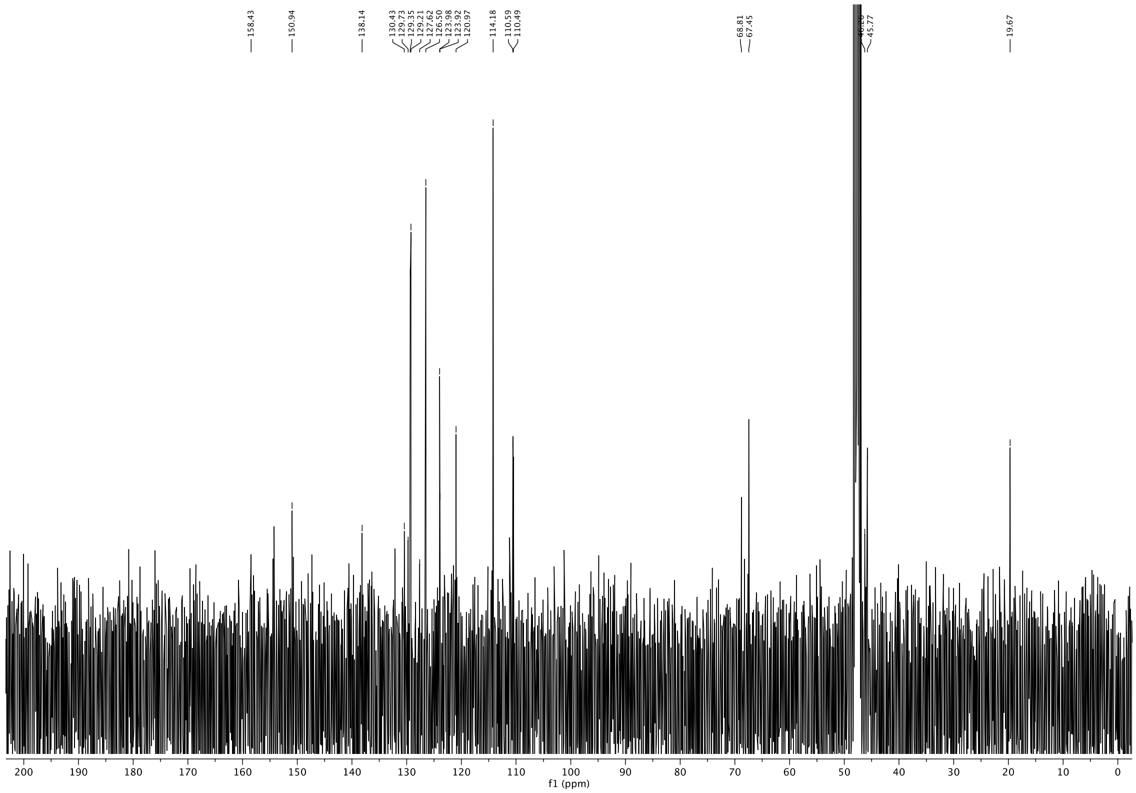

Figure S73. ^{13}C NMR spectrum (101 MHz, CD_3OD) of compound **72**.

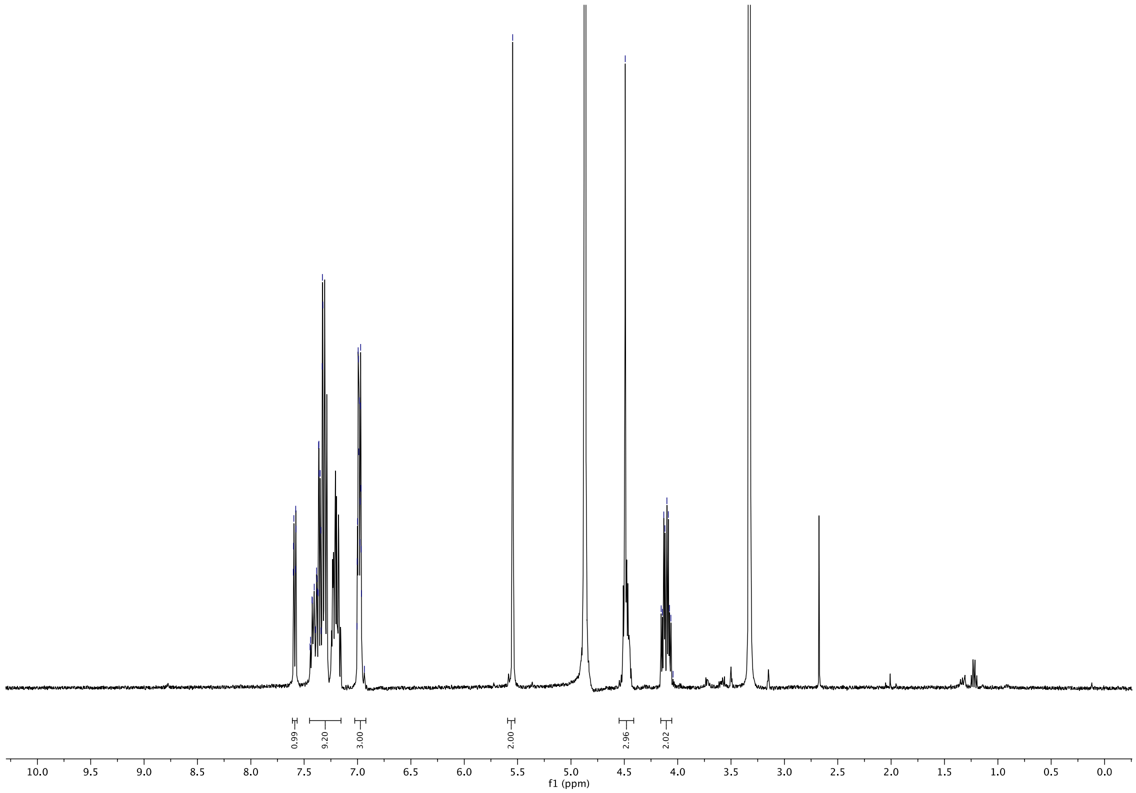

Figure S74. ^{1}H NMR spectrum (400 MHz, CD_3OD) of compound **73**.

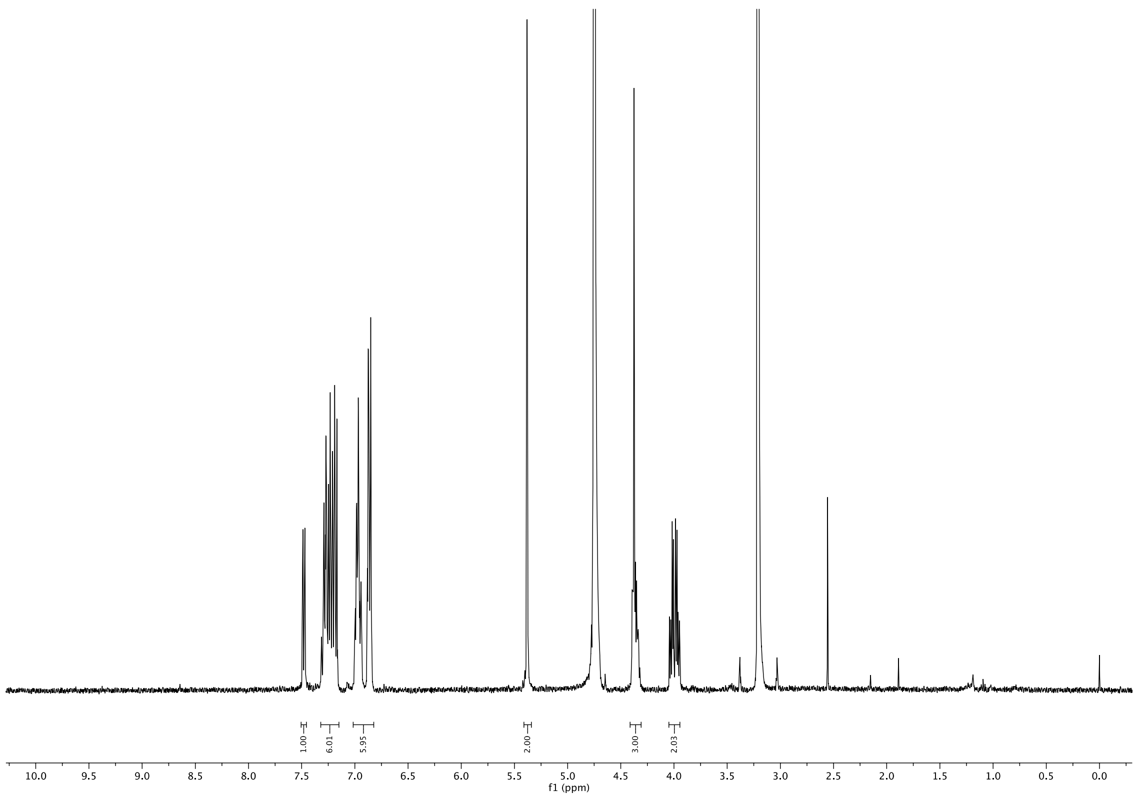

Figure S75. ^{1}H NMR spectrum (400 MHz, CD_3OD) of compound **74**.

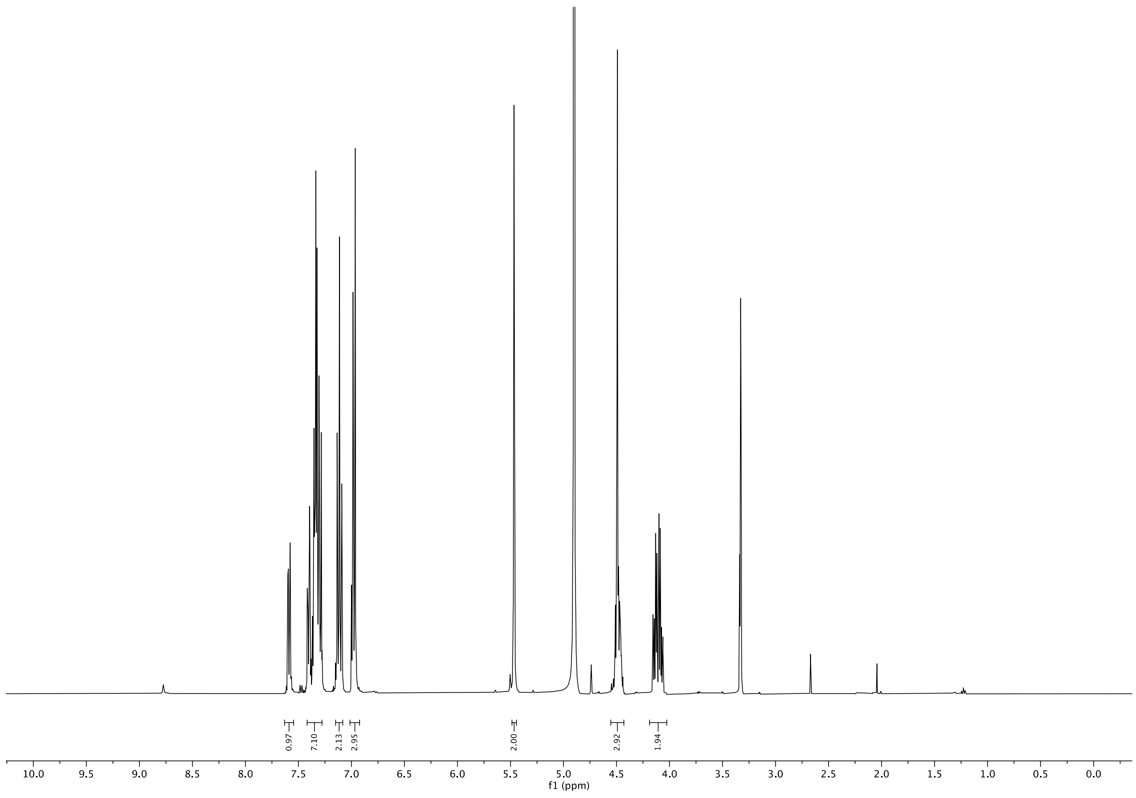

Figure S76. ^{1}H NMR spectrum (400 MHz, CD_3OD) of compound **75**.

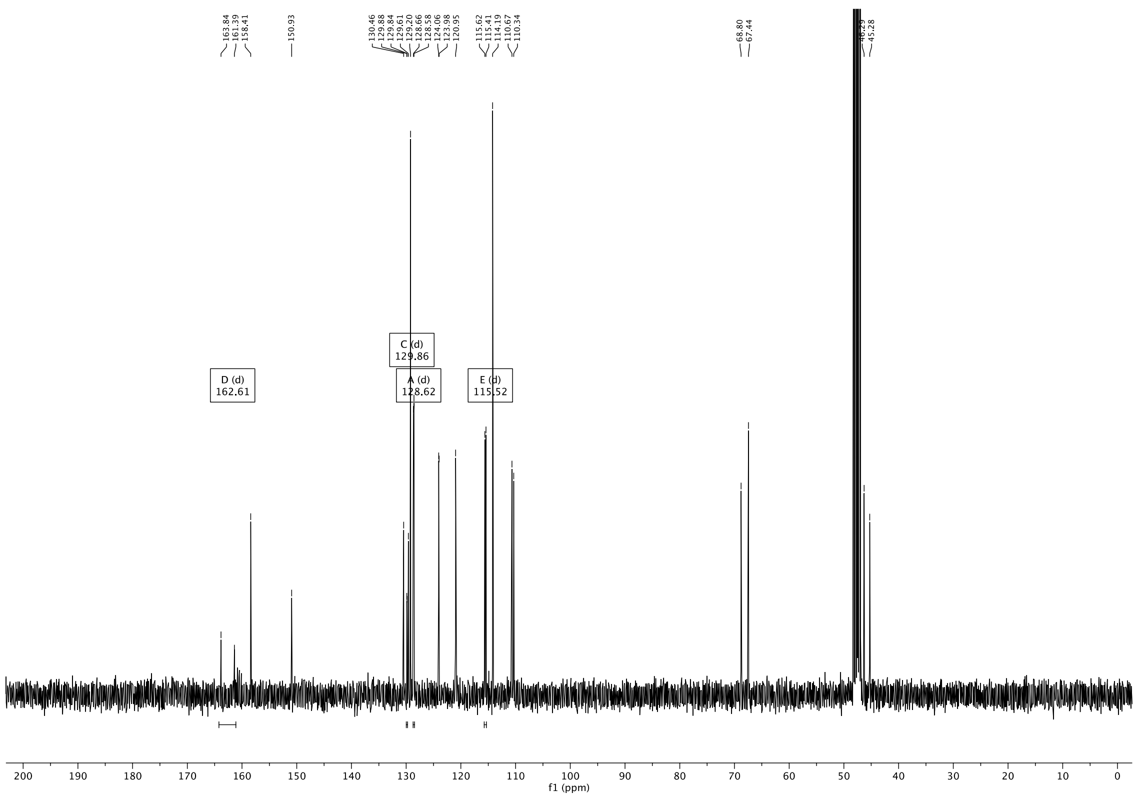

Figure S77. ^{13}C NMR spectrum (101 MHz, CD_3OD) of compound **75**.

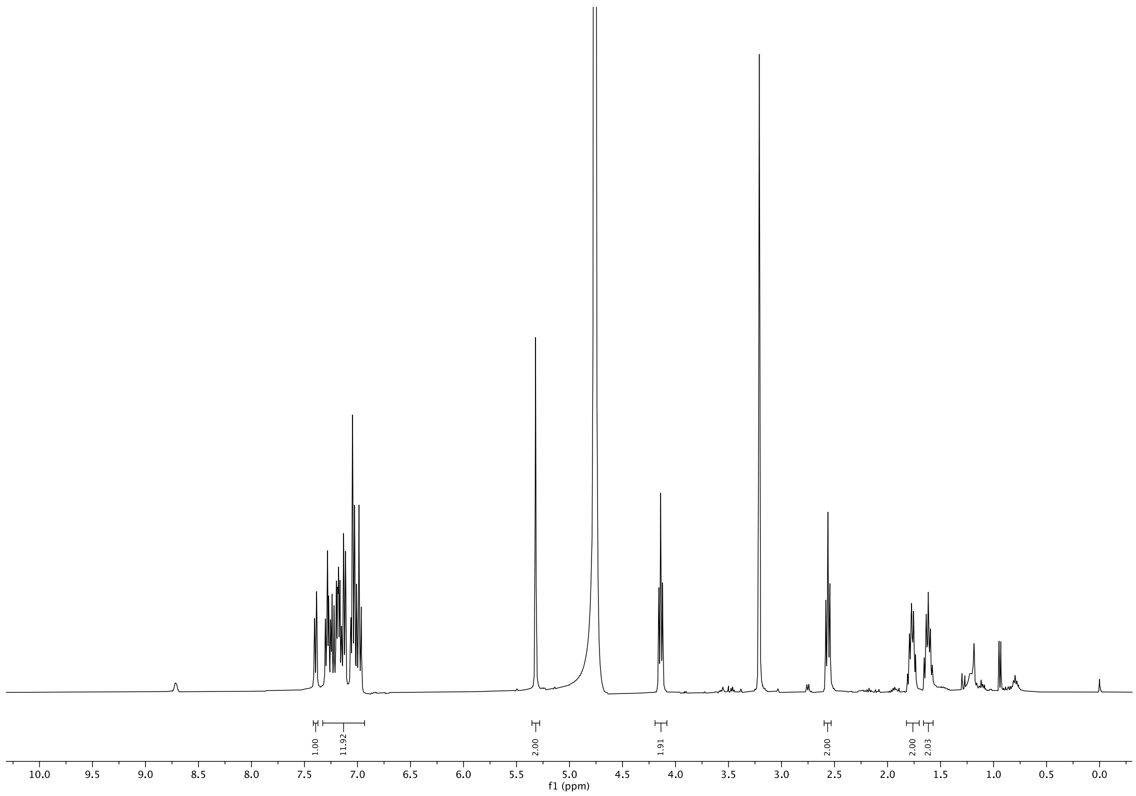

Figure S78. ^{1}H NMR spectrum (400 MHz, CD_3OD) of compound **76**.

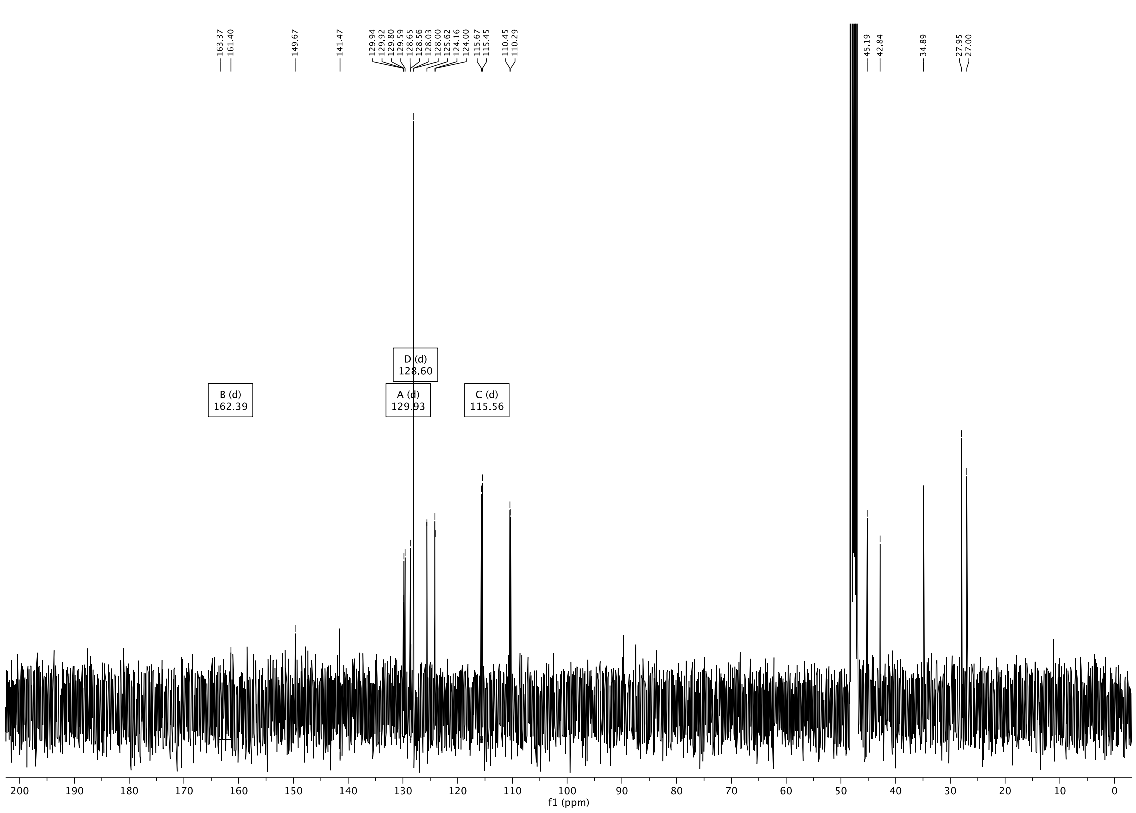

Figure S79. ^{13}C NMR spectrum (101 MHz, CD_3OD) of compound **76**.

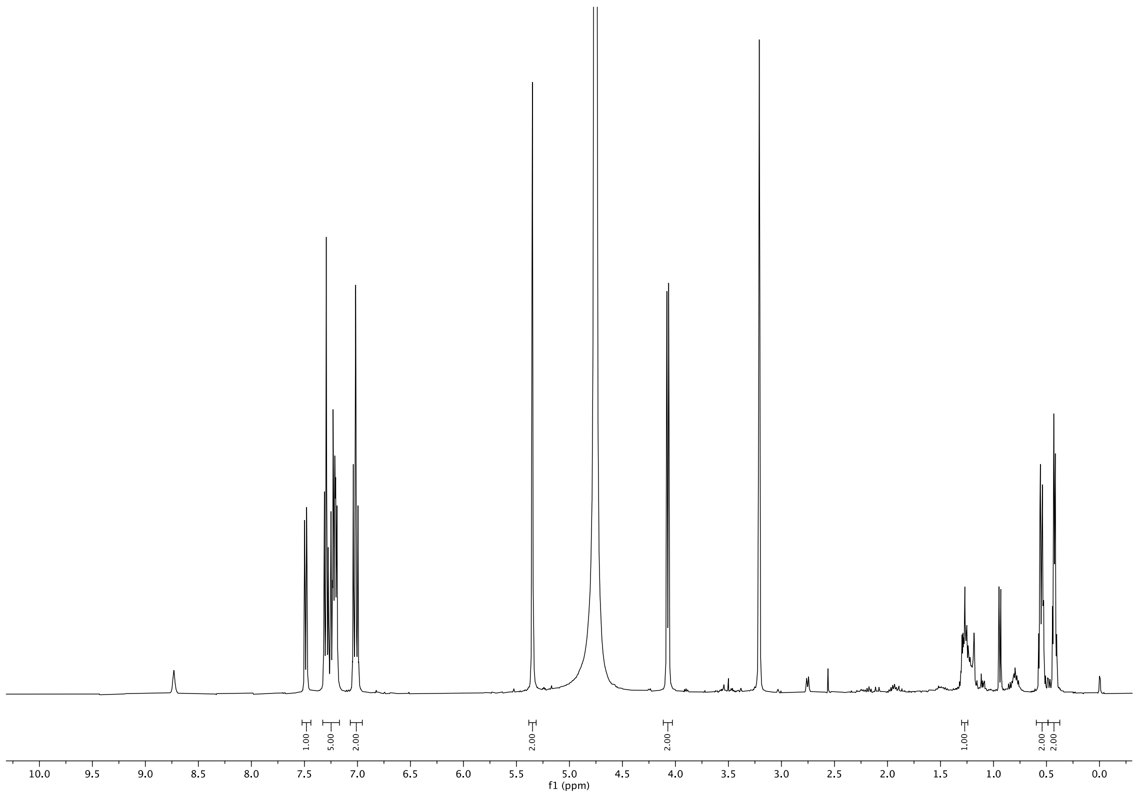

Figure S80. ^{1}H NMR spectrum (400 MHz, CD_3OD) of compound **77**.

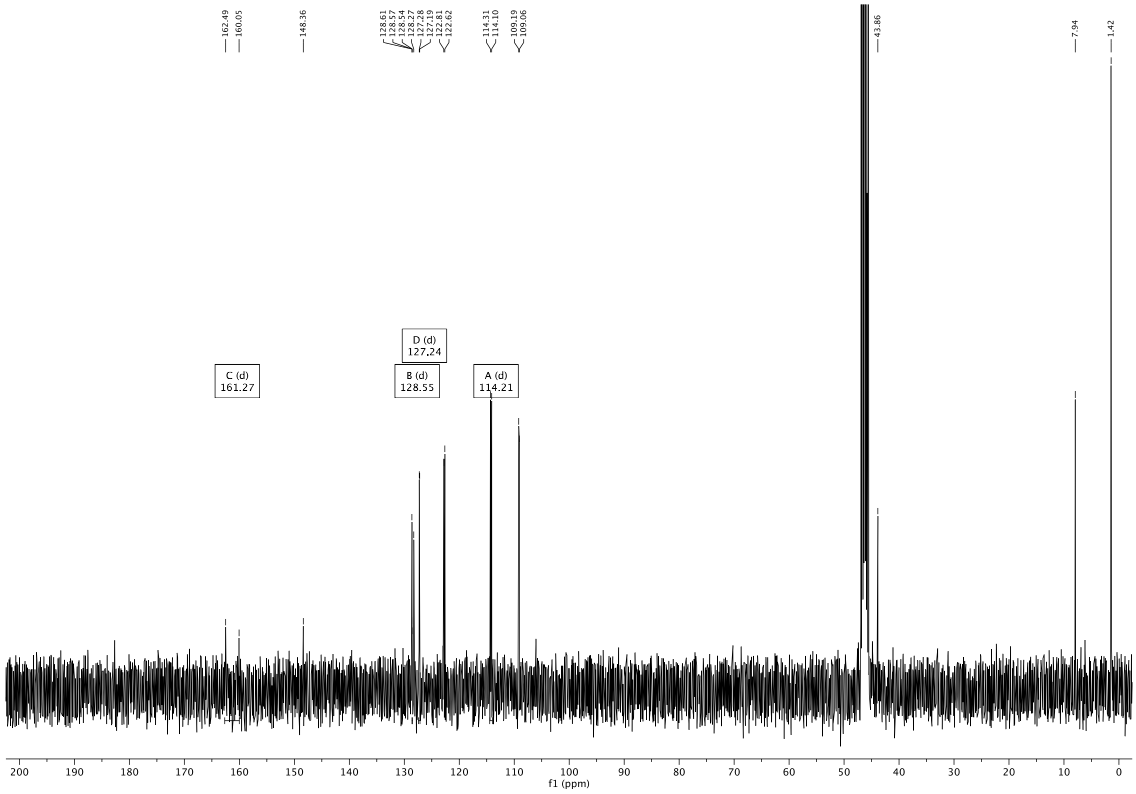

Figure S81. ^{13}C NMR spectrum (101 MHz, CD_3OD) of compound **77**.

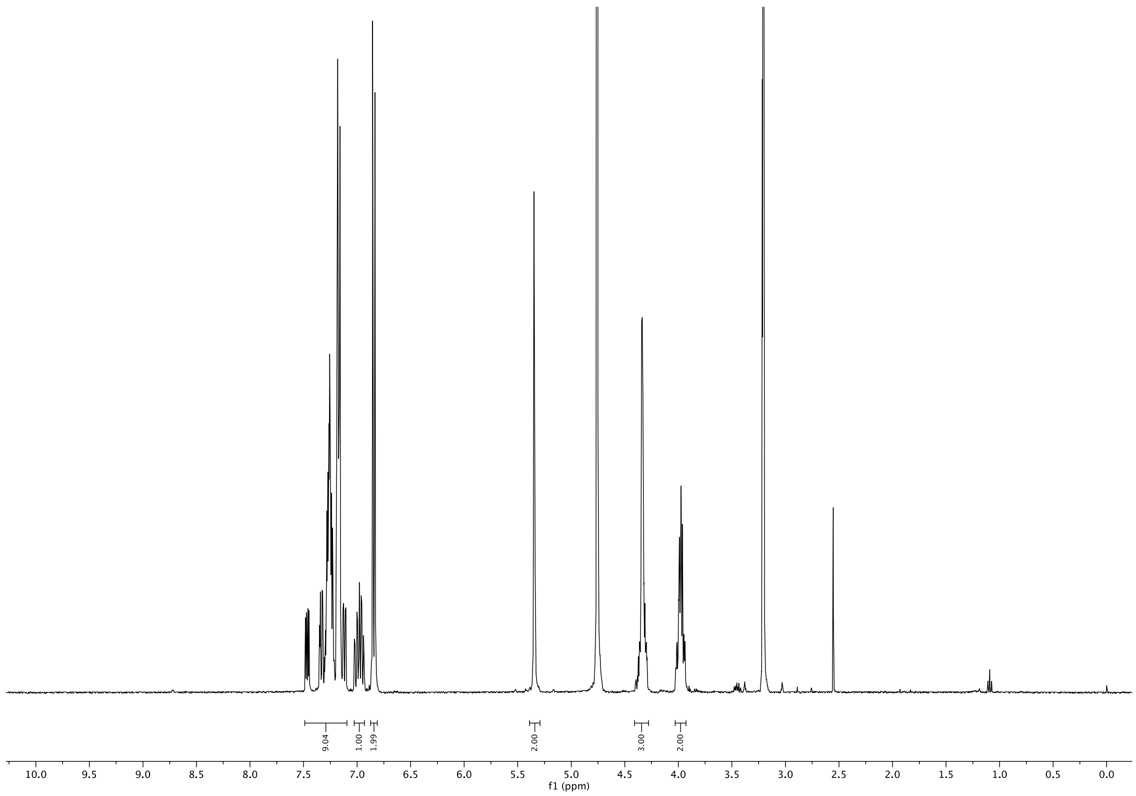

Figure S82. ^{1}H NMR spectrum (400 MHz, CD_3OD) of compound **80** (mixures of isomers).

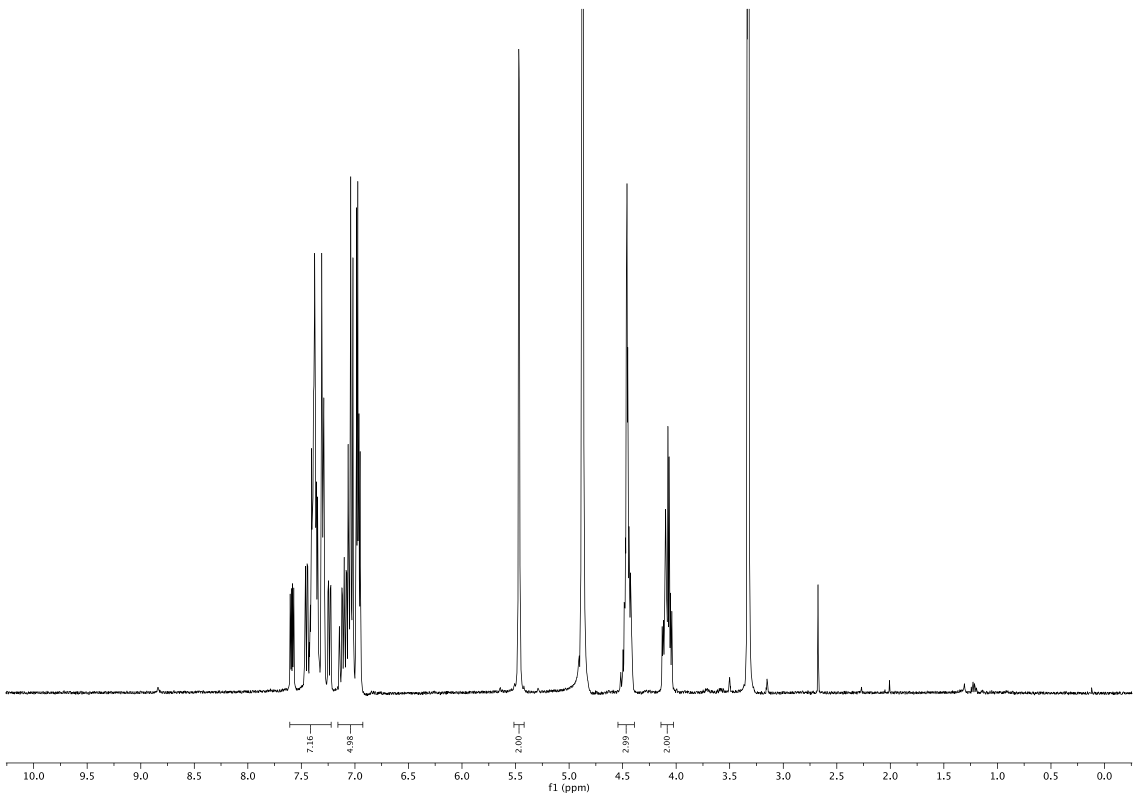

Figure S83. ^{1}H NMR spectrum (400 MHz, CD_3OD) of compound **81** (mixures of isomers).

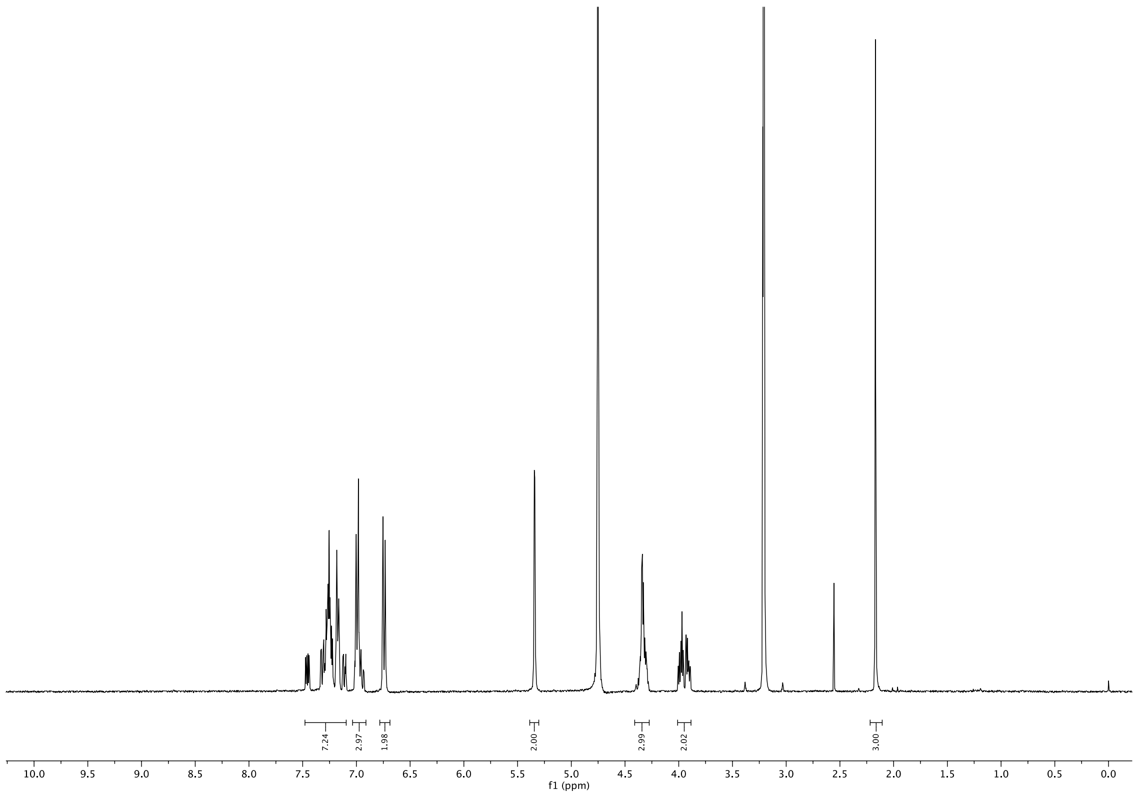

Figure S84. ^{1}H NMR spectrum (400 MHz, CD_3OD) of compound **82** (mixures of isomers).

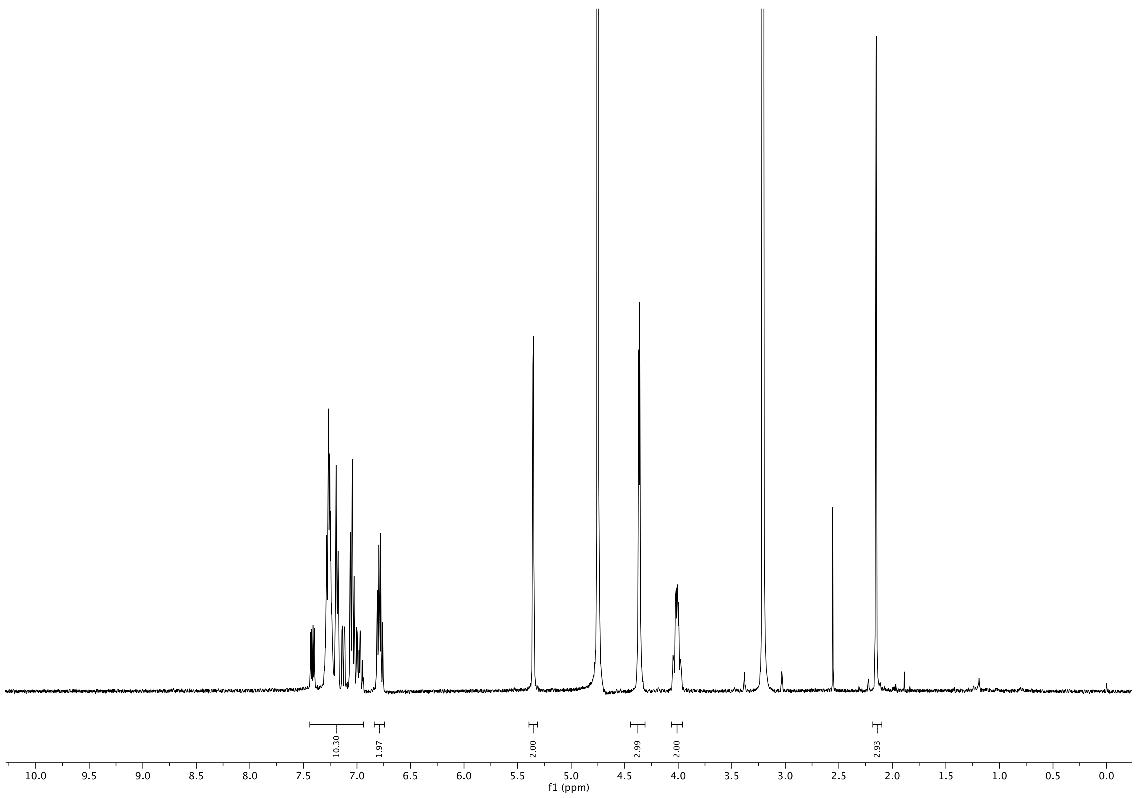

Figure S85. ^{1}H NMR spectrum (400 MHz, CD_3OD) of compound **83** (mixures of isomers).

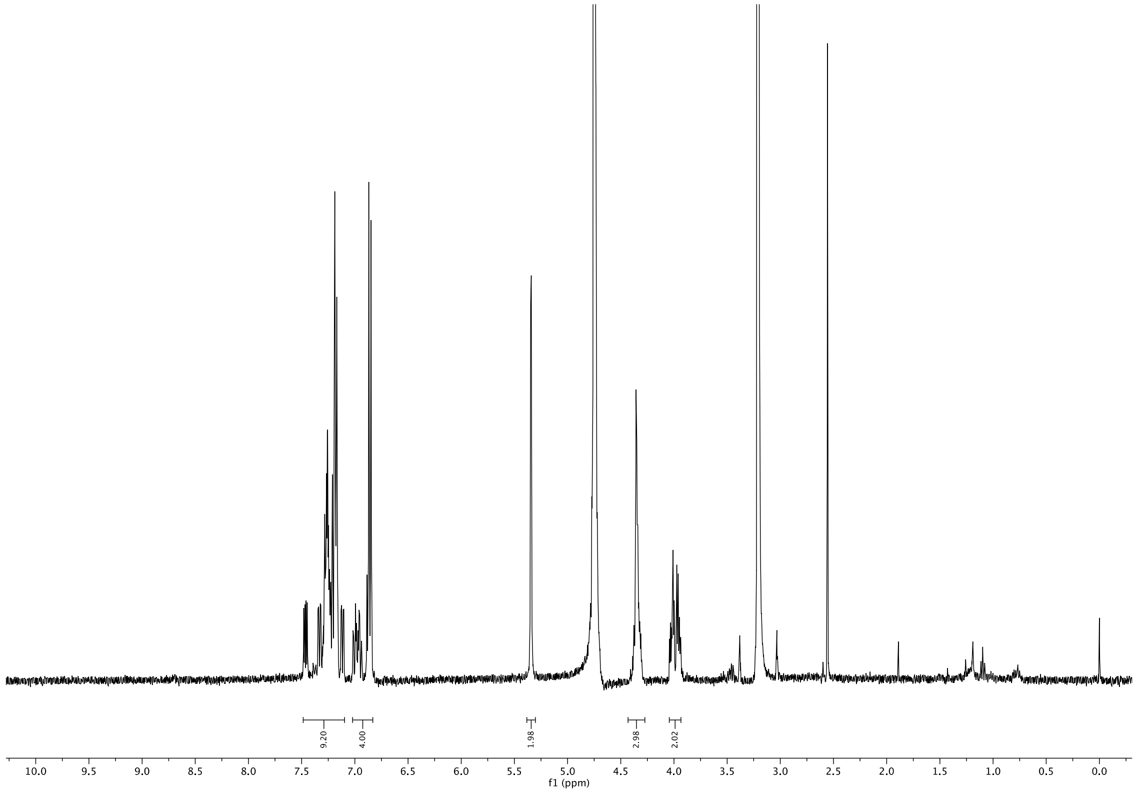

Figure S86. ^{1}H NMR spectrum (400 MHz, CD_3OD) of compound **84** (mixures of isomers).

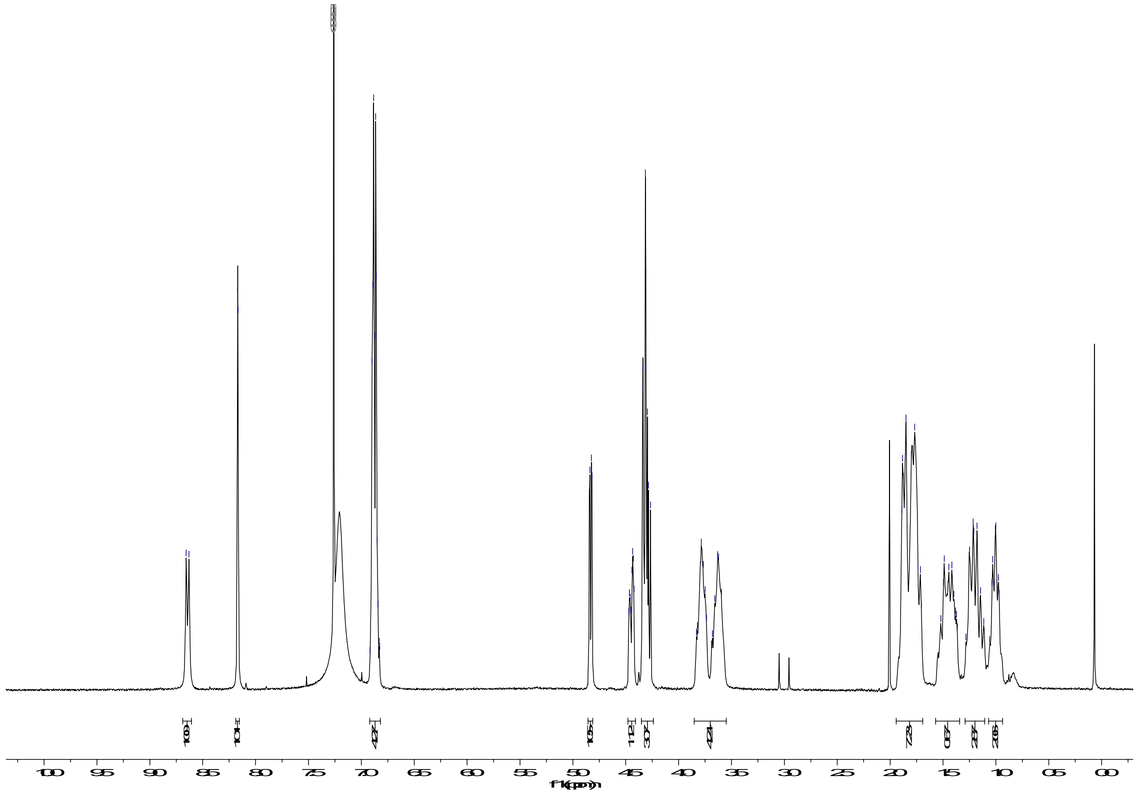

Figure S87. ^{1}H NMR spectrum (400 MHz, $CDCl_3$) of compound **93**.

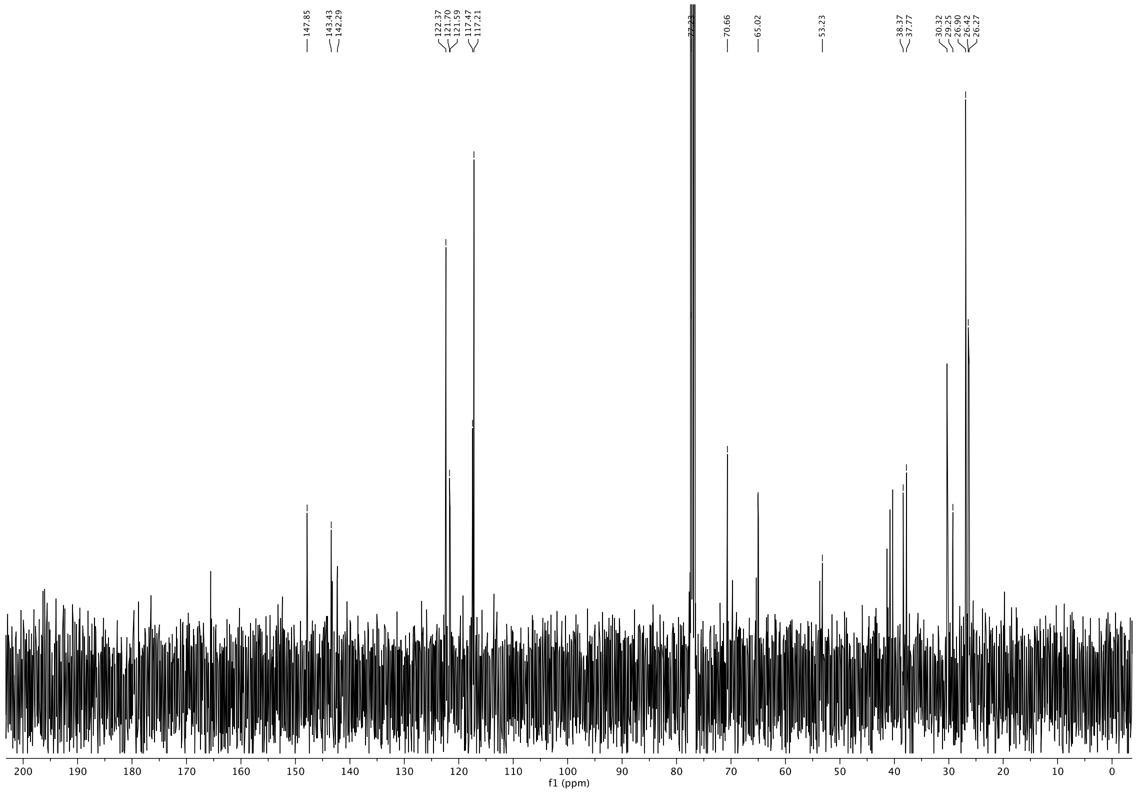

Figure S88. ^{13}C NMR spectrum (101 MHz, $CDCl_3$) of compound **93**.

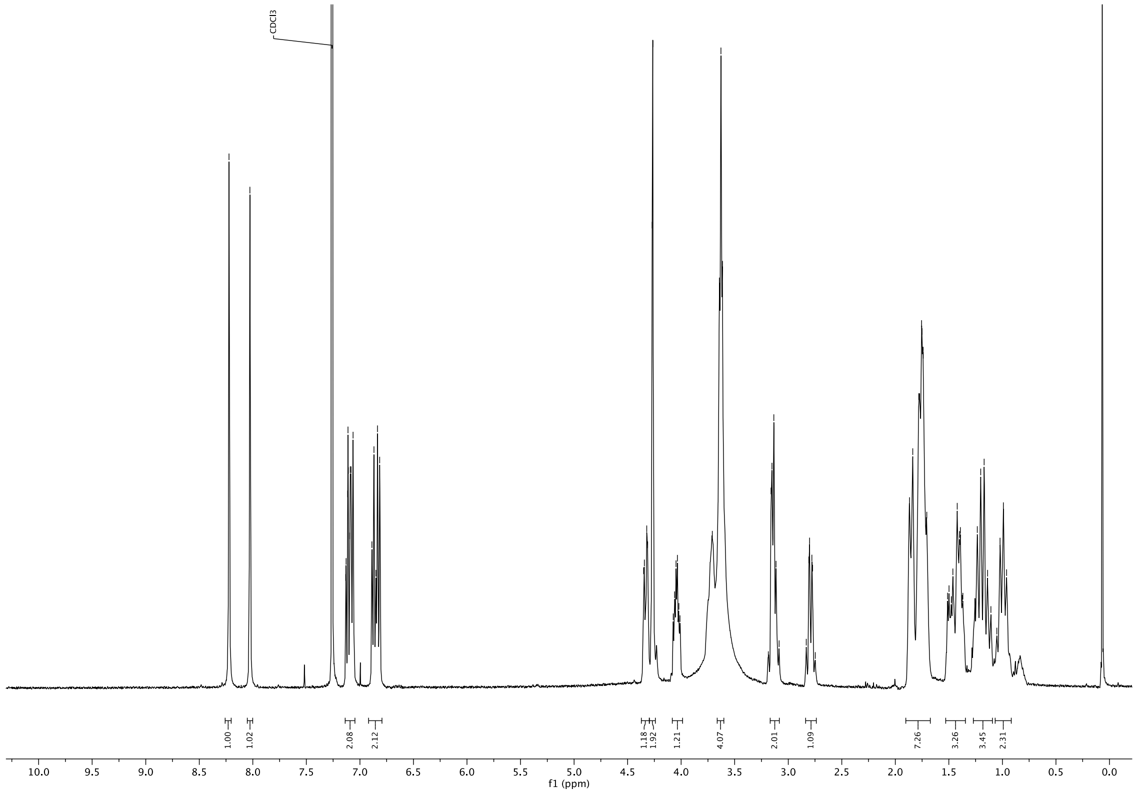

Figure S89. ^{1}H NMR spectrum (400 MHz, $CDCl_3$) of compound **94**.

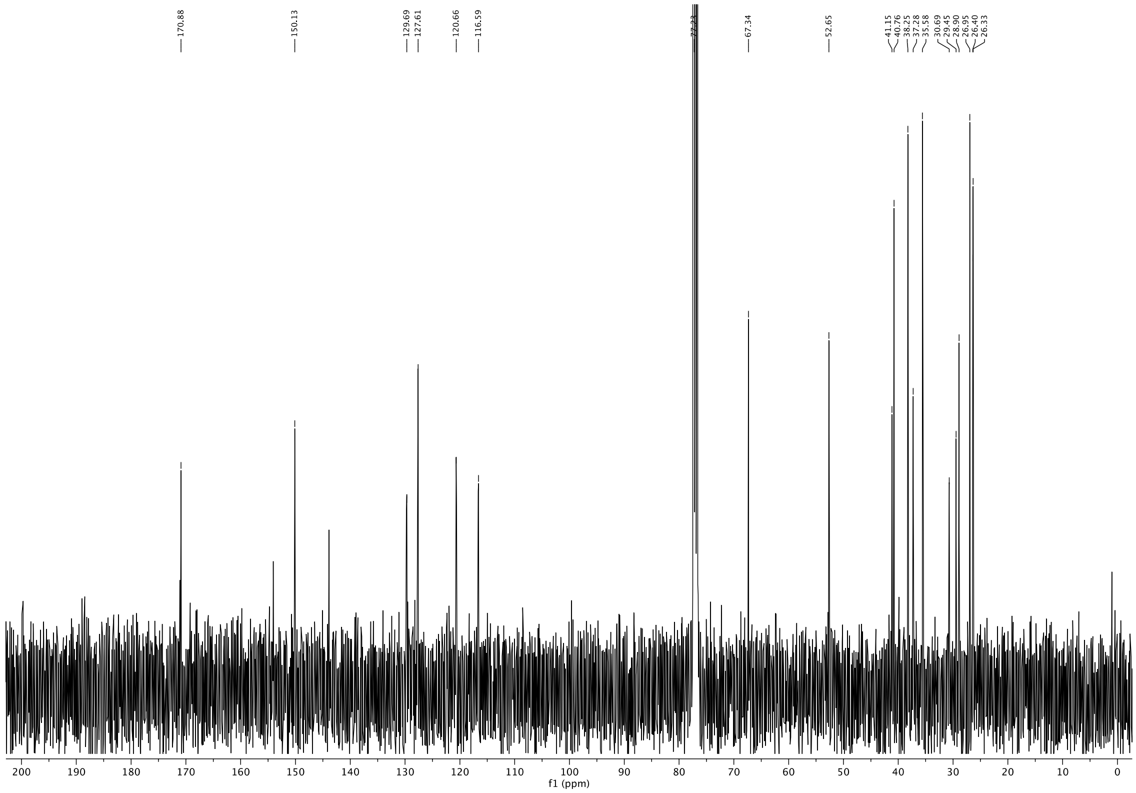

Figure S90. ^{13}C NMR spectrum (101 MHz, $CDCl_3$) of compound **94**.

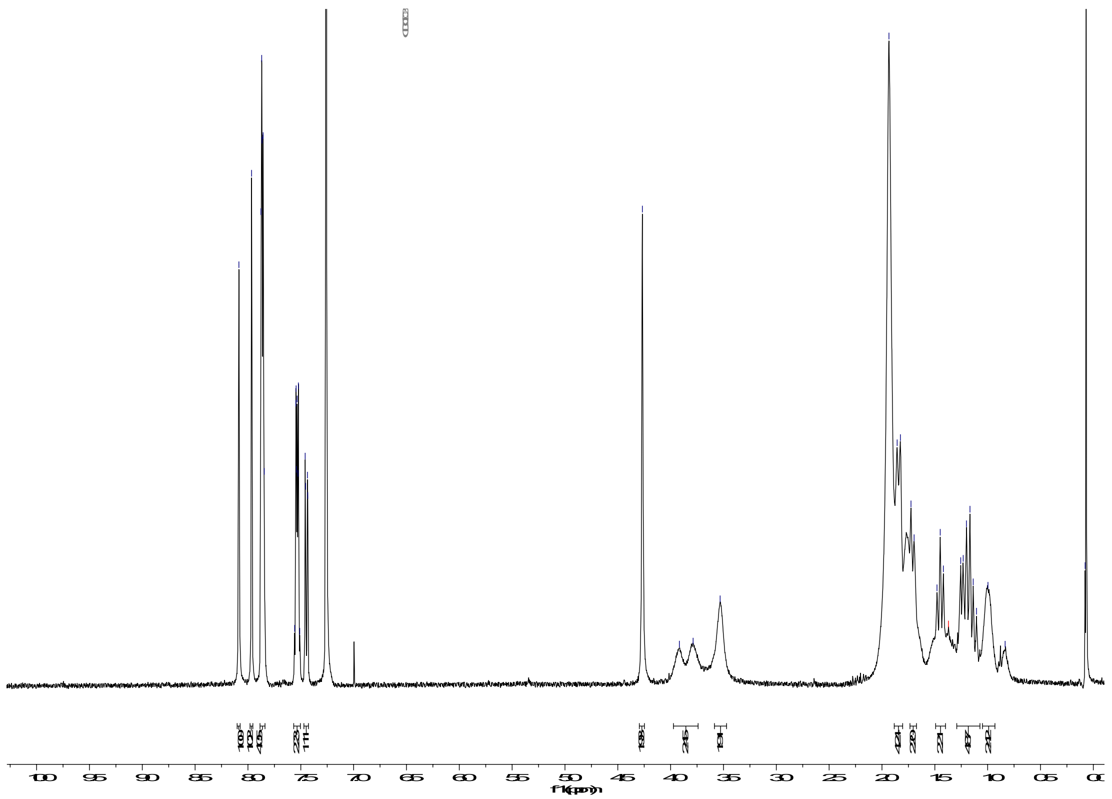

Figure S91. ^{1}H NMR spectrum (400 MHz, $CDCl_3$) of compound **95**.

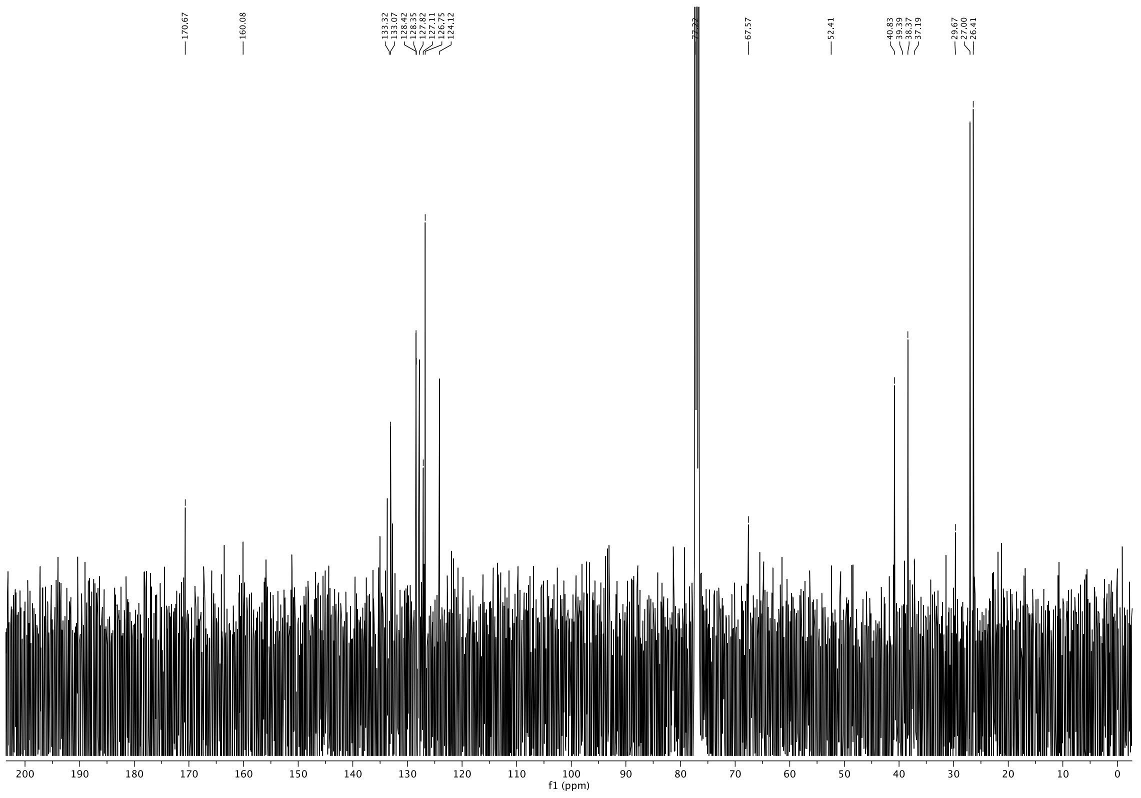

Figure S92. ^{13}C NMR spectrum (101 MHz, $CDCl_3$) of compound **95**.

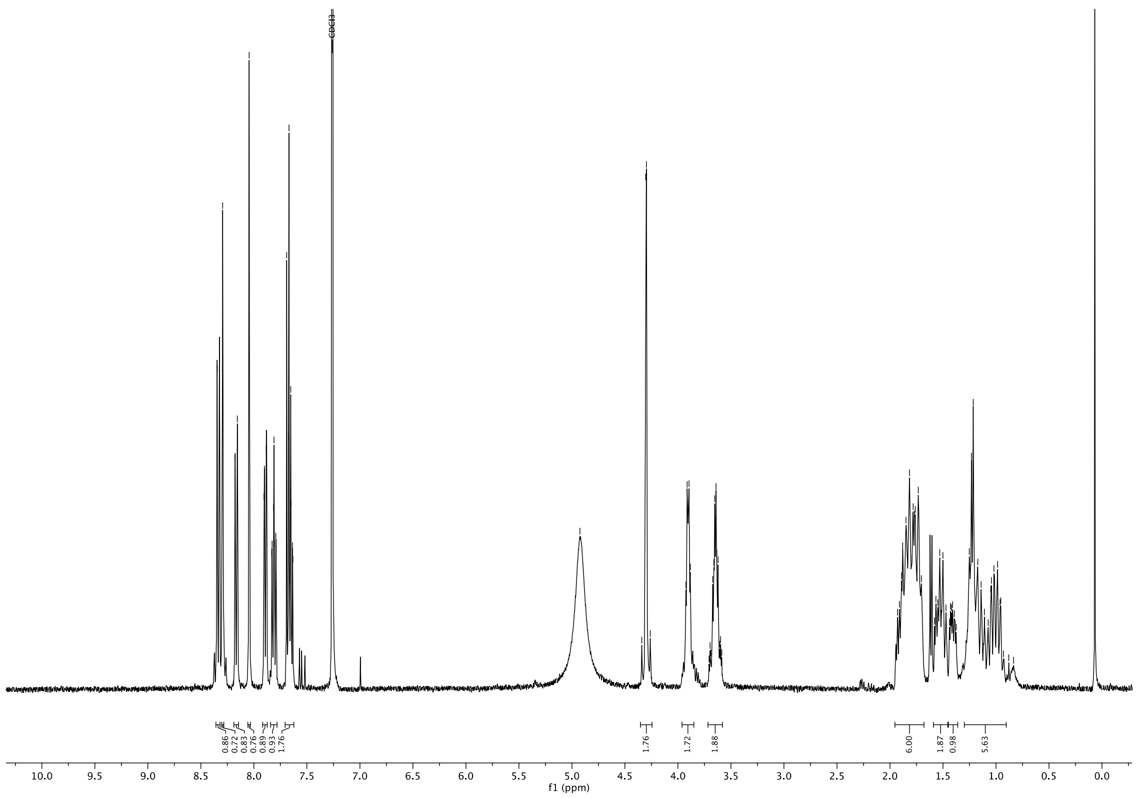

Figure S93. ^{1}H NMR spectrum (400 MHz, $CDCl_3$) of compound **96**.

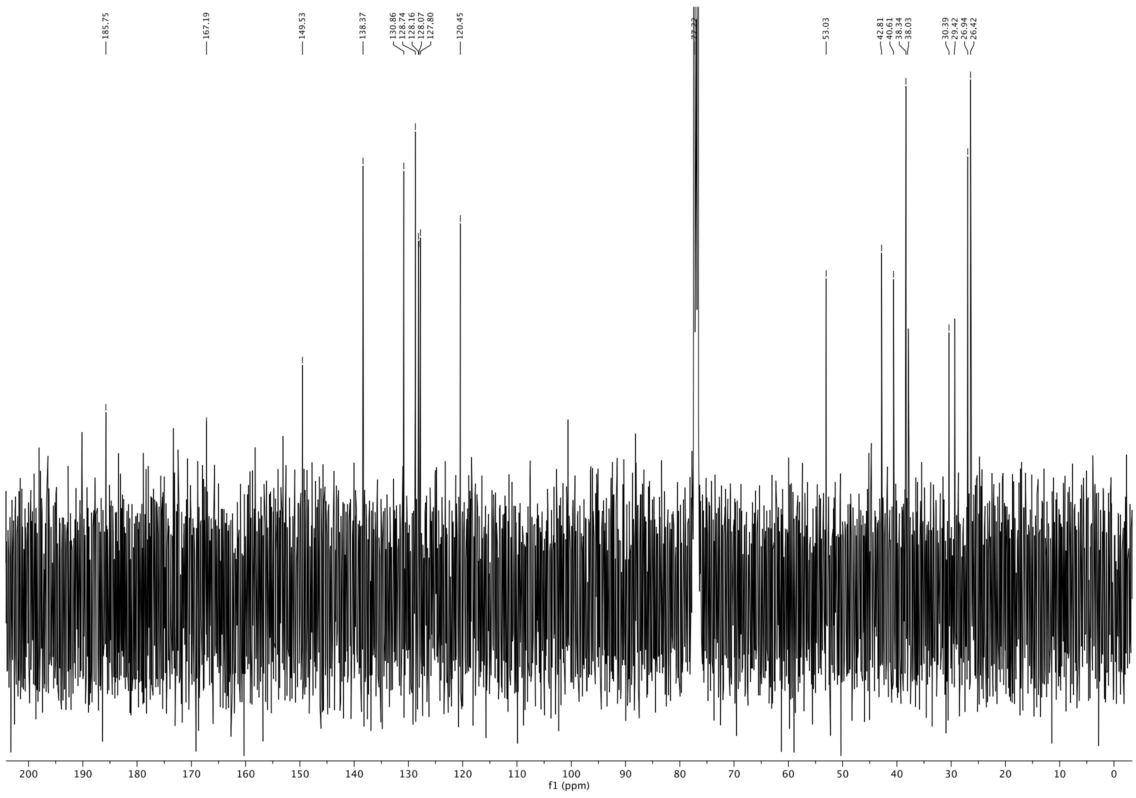

Figure S94. ^{13}C NMR spectrum (101 MHz, $CDCl_3$) of compound **96**.

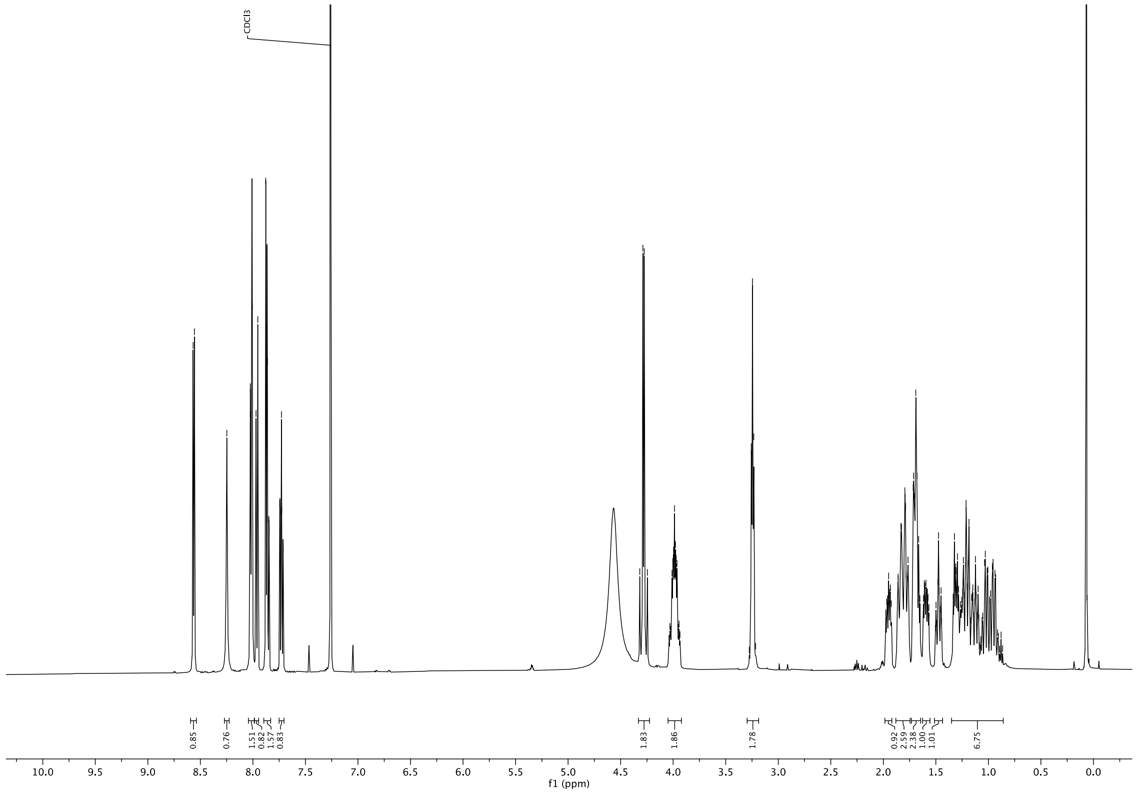

Figure S95. ^{1}H NMR spectrum (400 MHz, $CDCl_3$) of compound **97**.

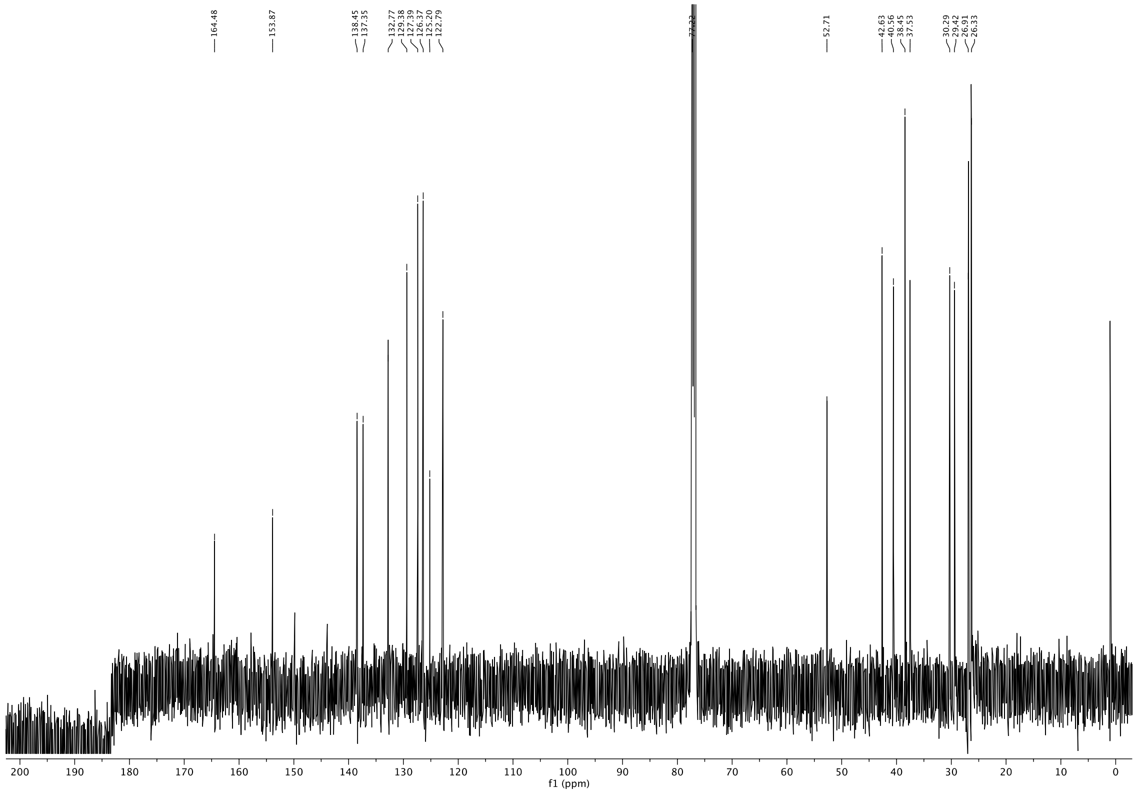

Figure S96. ^{13}C NMR spectrum (101 MHz, $CDCl_3$) of compound **97**.

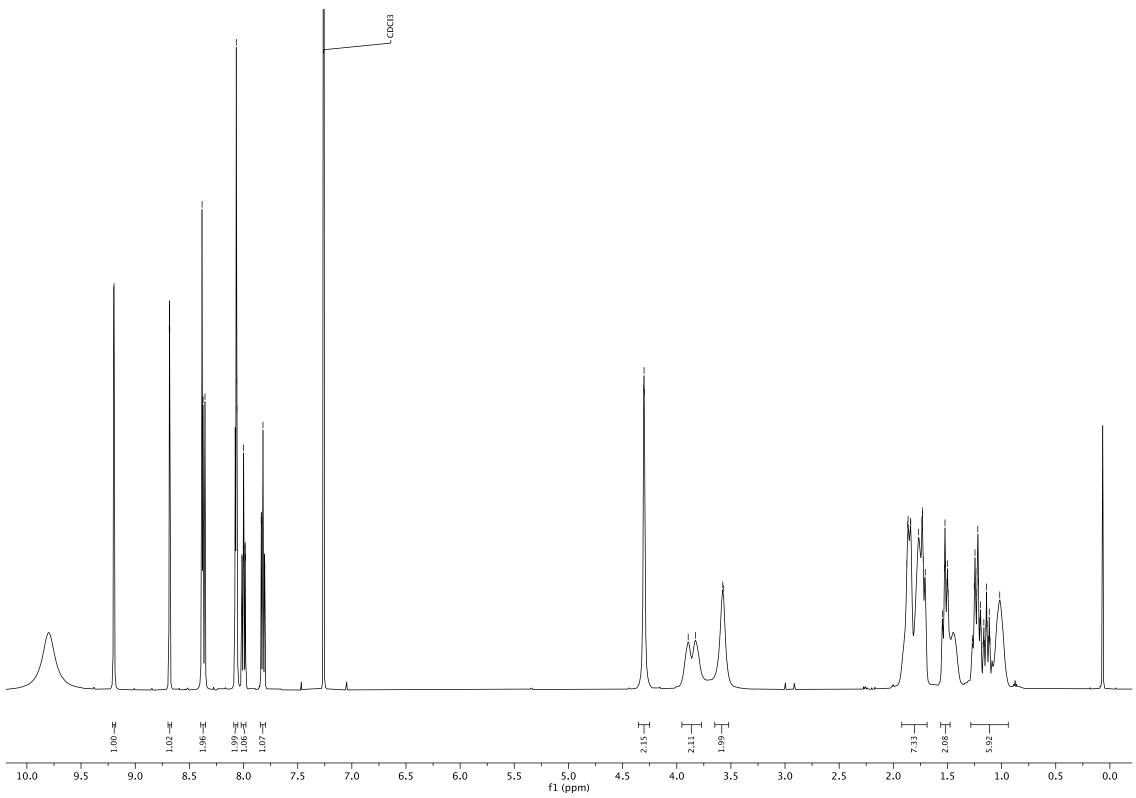

Figure S97. ^{1}H NMR spectrum (500 MHz, $CDCl_3$) of compound **98**.

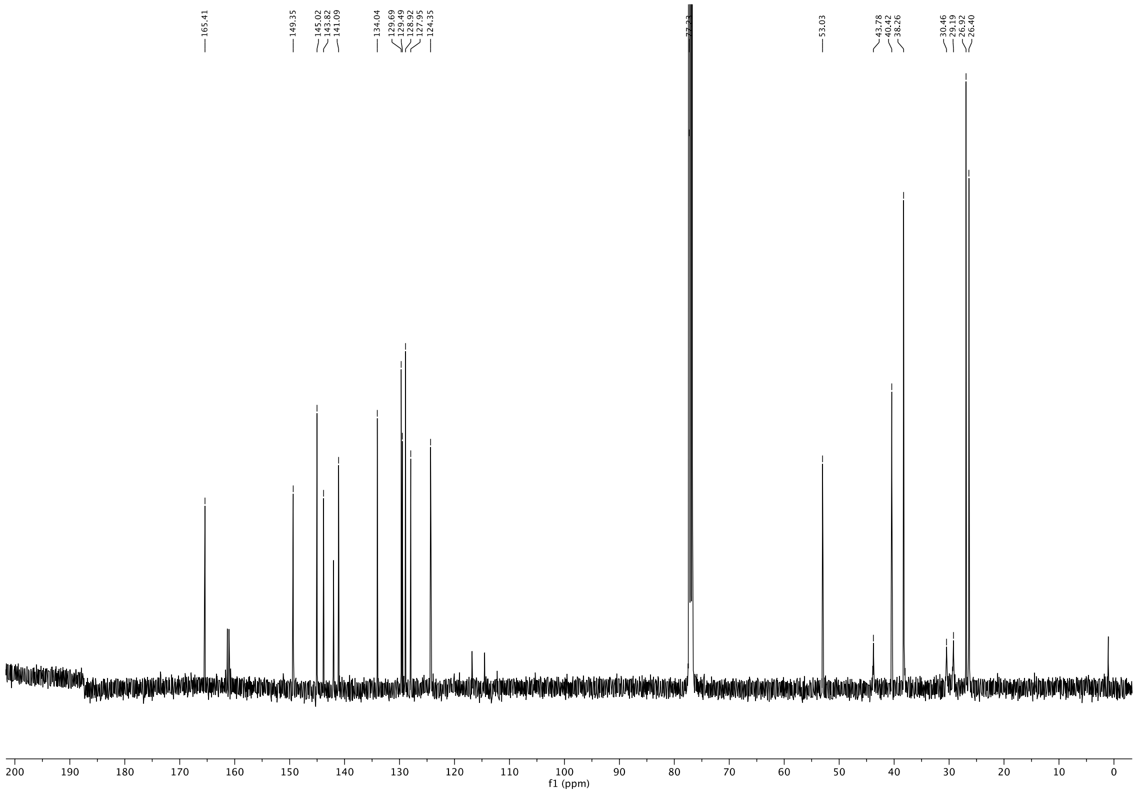

Figure S98. ^{13}C NMR spectrum (126 MHz, $CDCl_3$) of compound **98**.

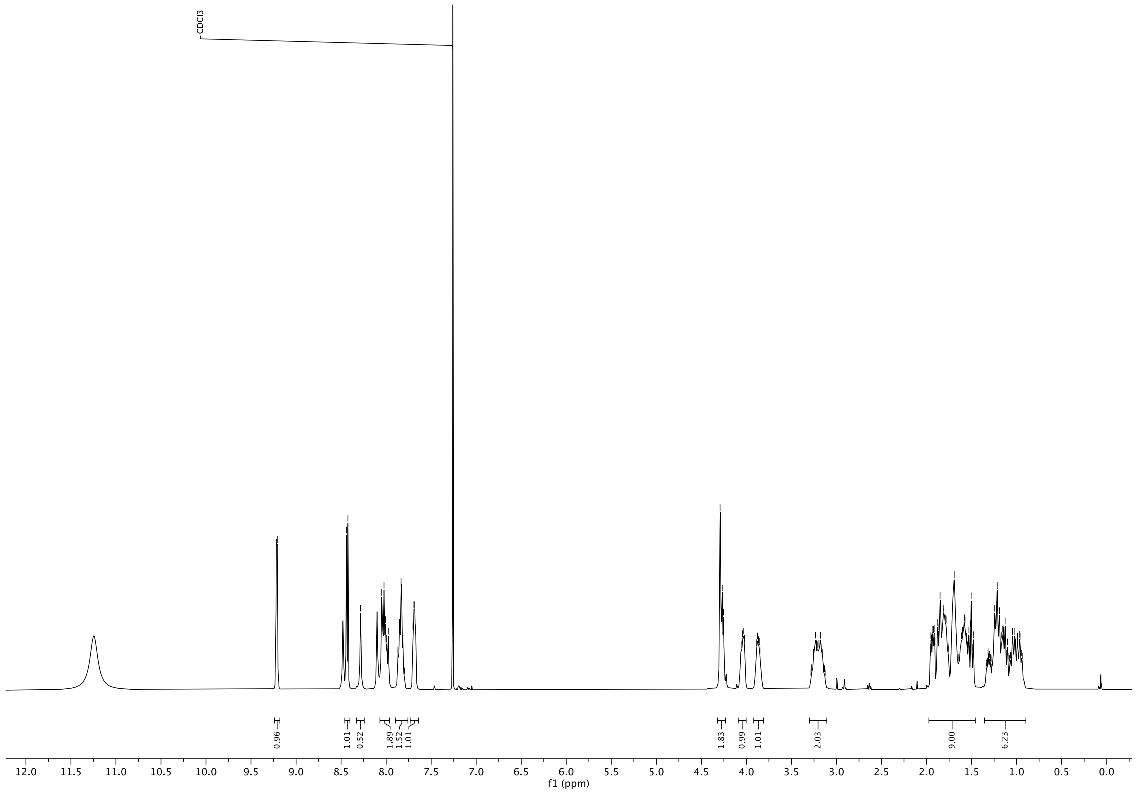

Figure S99. ^{1}H NMR spectrum (500 MHz, $CDCl_3$) of compound **99**.

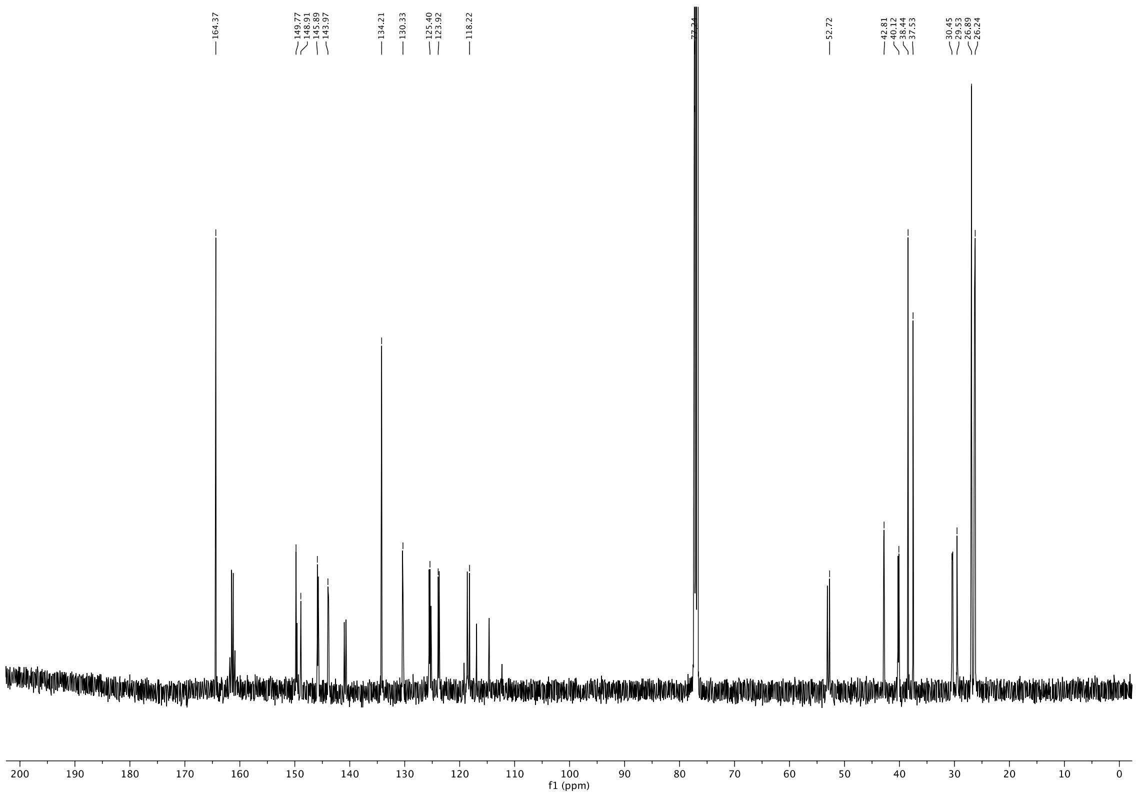

Figure S100. ^{13}C NMR spectrum (126 MHz, $CDCl_3$) of compound **99**.

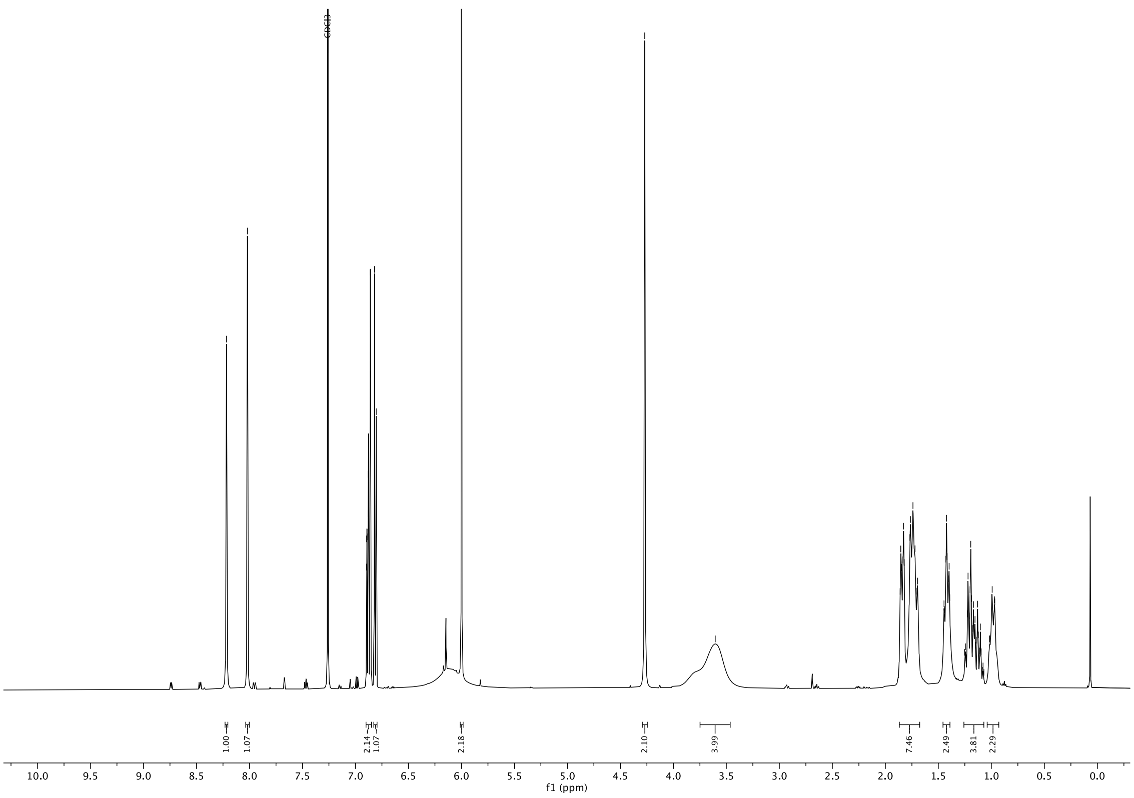

Figure S101. ^{1}H NMR spectrum (500 MHz, $CDCl_3$) of compound **100**.

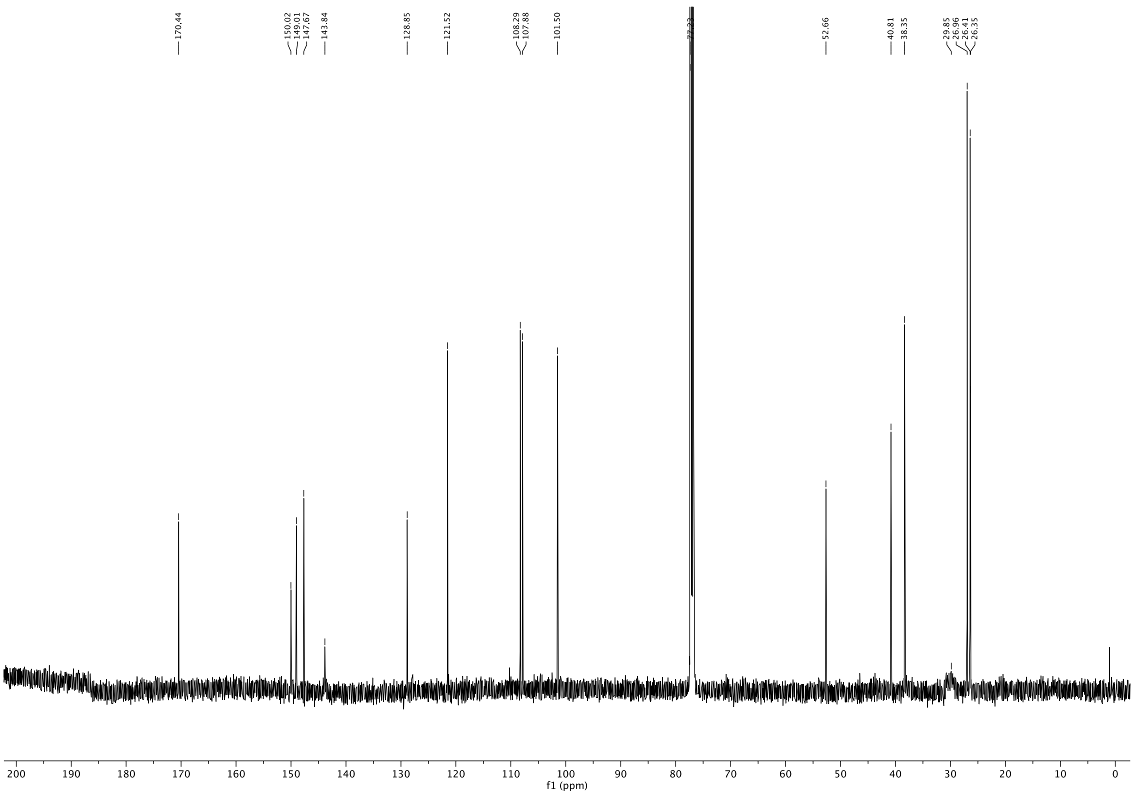

Figure S102. ^{13}C NMR spectrum (126 MHz, $CDCl_3$) of compound **100**.

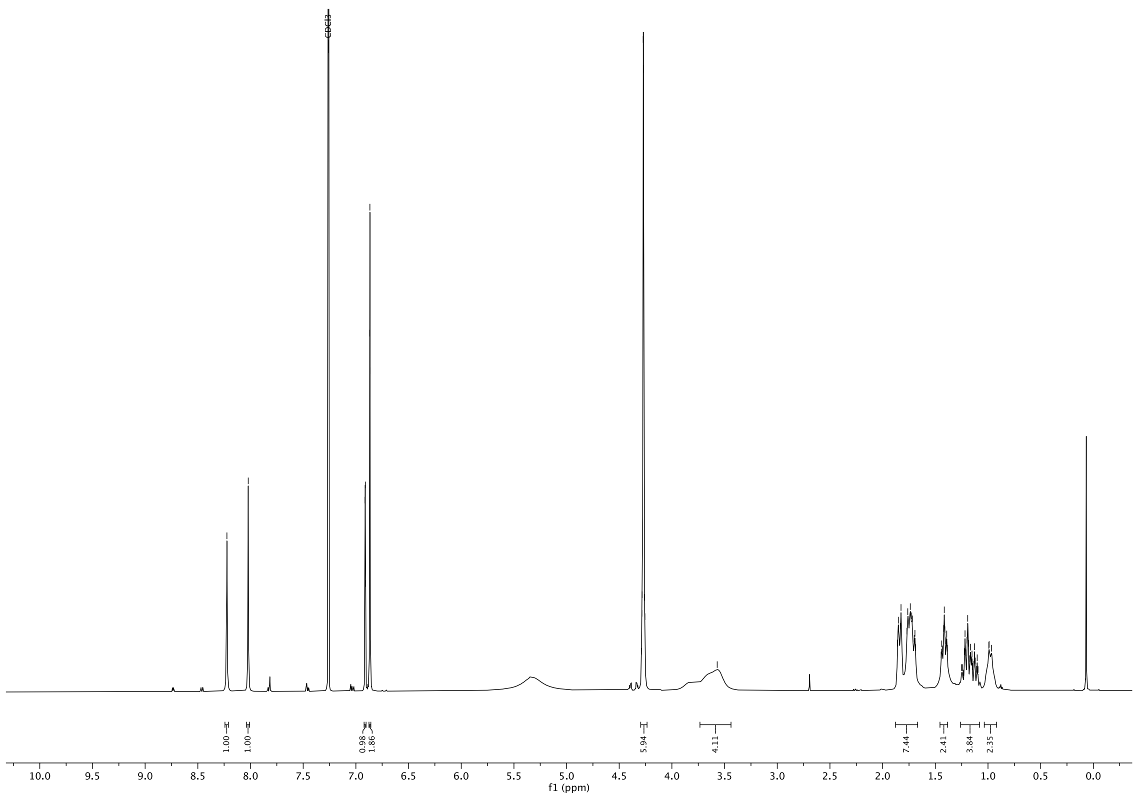

Figure S103. ^{1}H NMR spectrum (500 MHz, $CDCl_3$) of compound **101**.

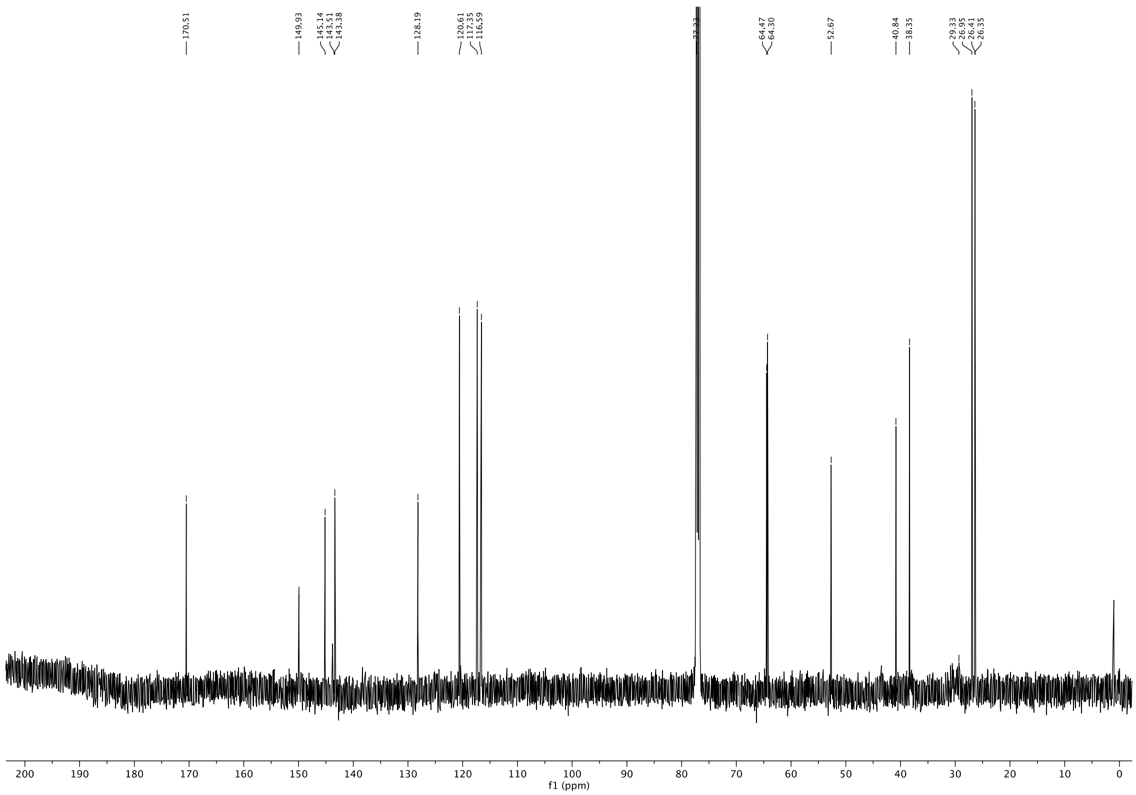

Figure S104. ^{13}C NMR spectrum (126 MHz, $CDCl_3$) of compound **101**.

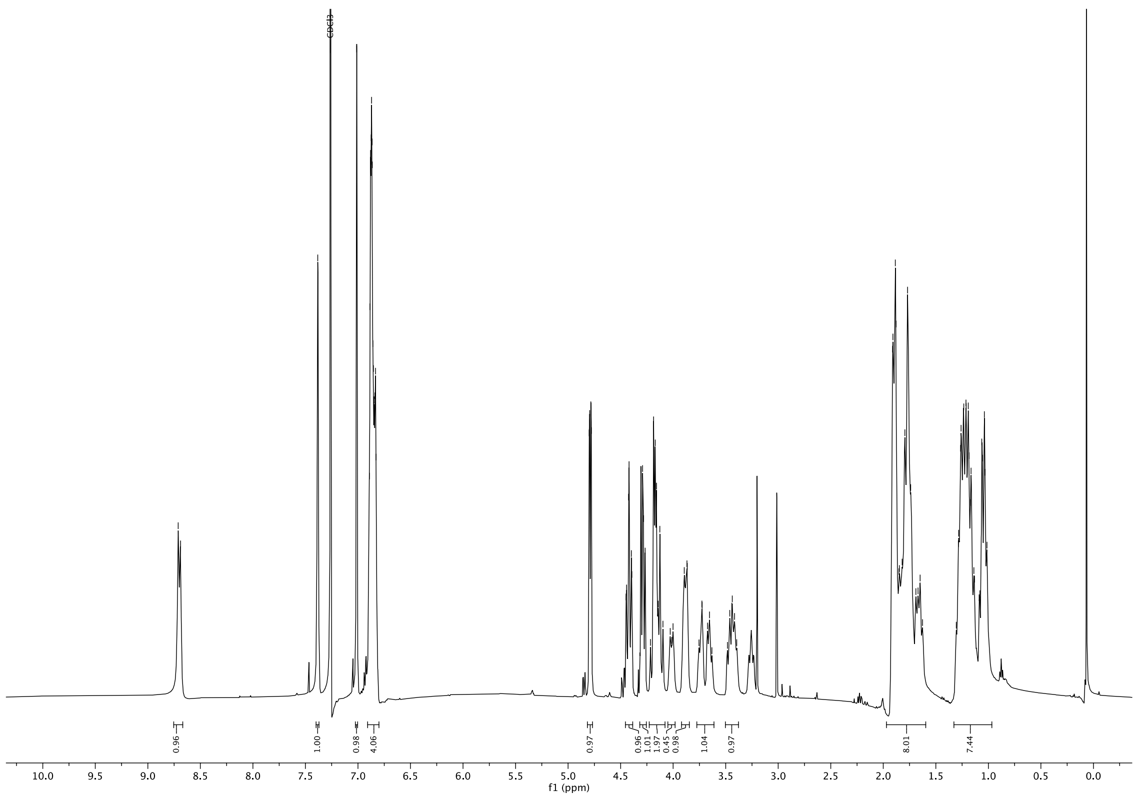

Figure S105. ^{1}H NMR spectrum (500 MHz, $CDCl_3$) of compound **102**.

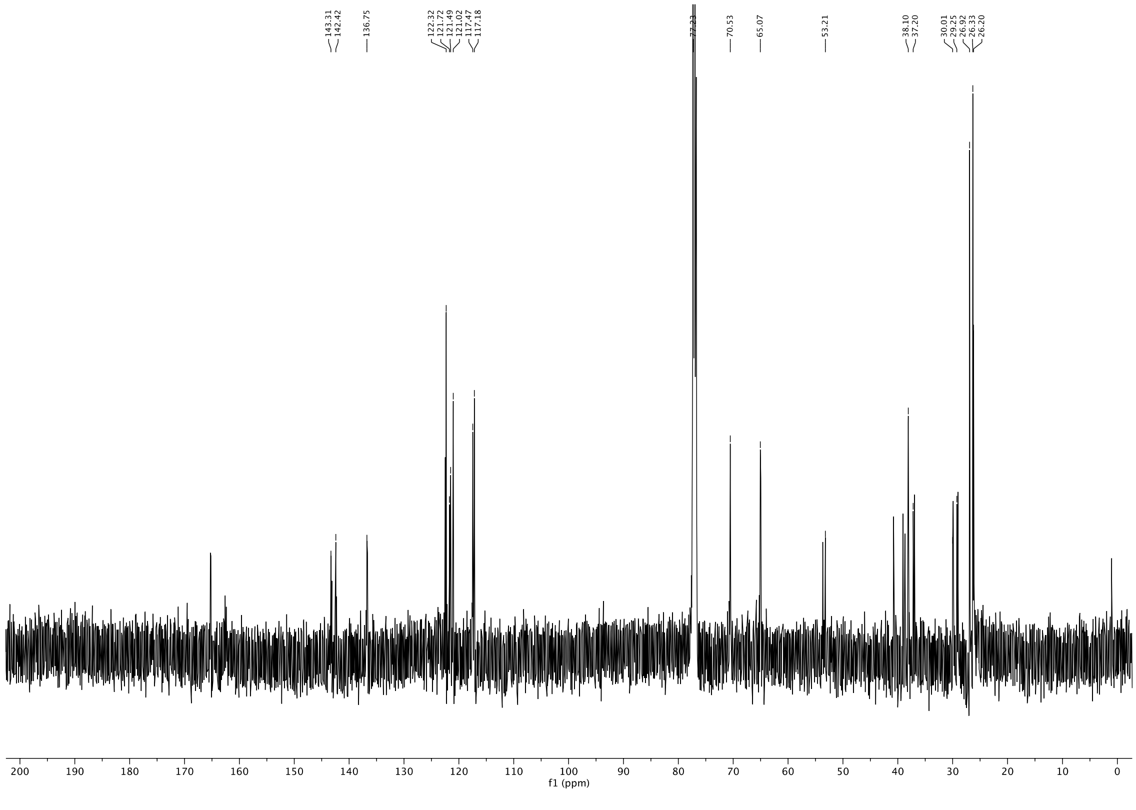

Figure S106. ^{13}C NMR spectrum (126 MHz, $CDCl_3$) of compound **102**.

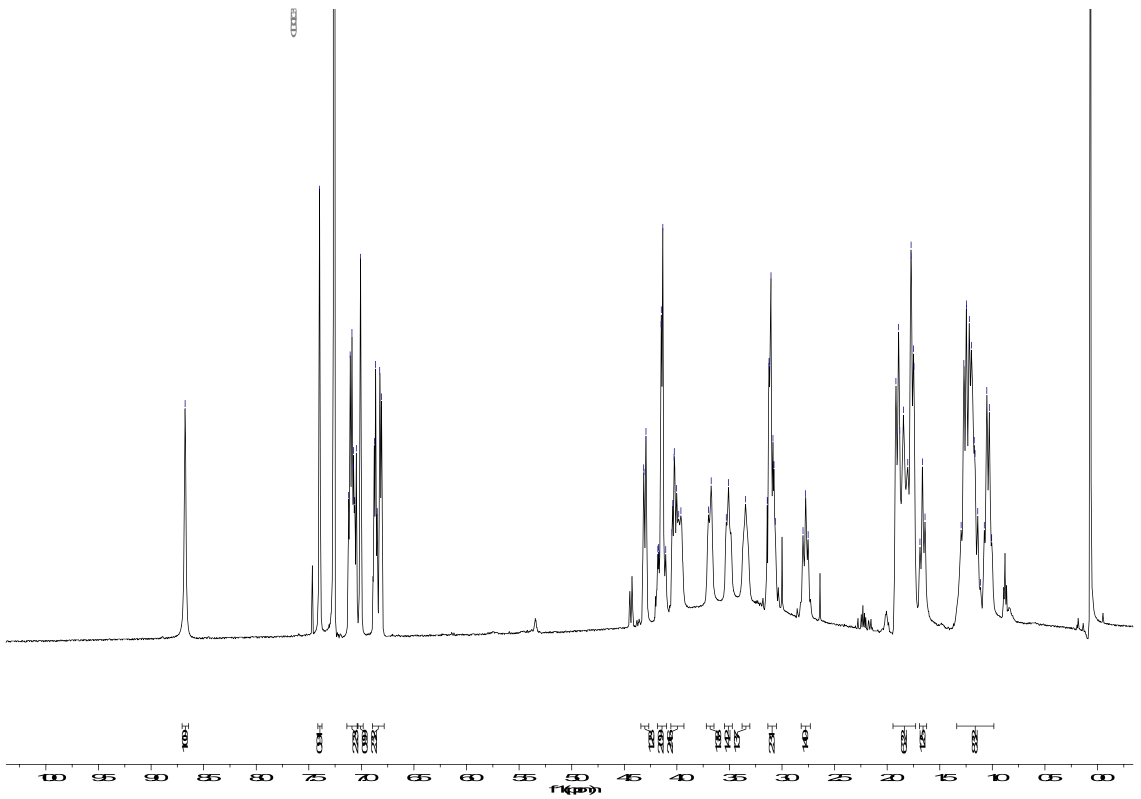

Figure S107. ^{1}H NMR spectrum (500 MHz, $CDCl_3$) of compound **103**.

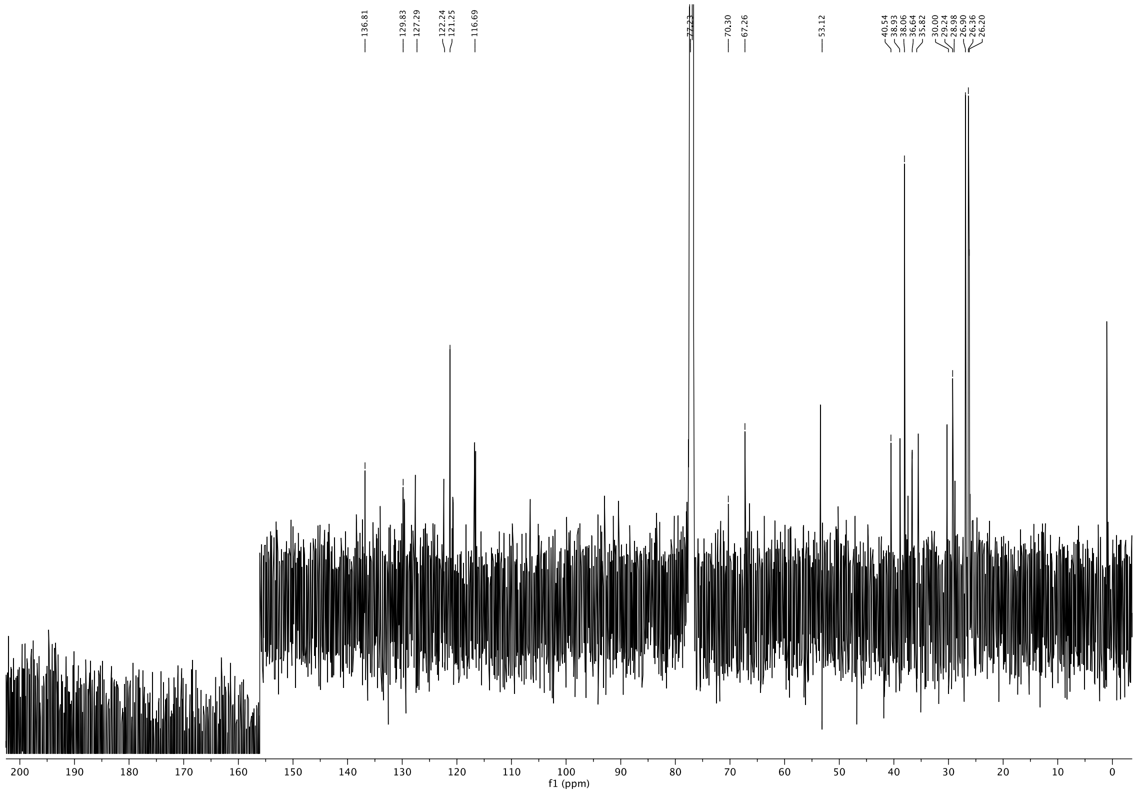

Figure S108. ^{13}C NMR spectrum (126 MHz, $CDCl_3$) of compound **103**.

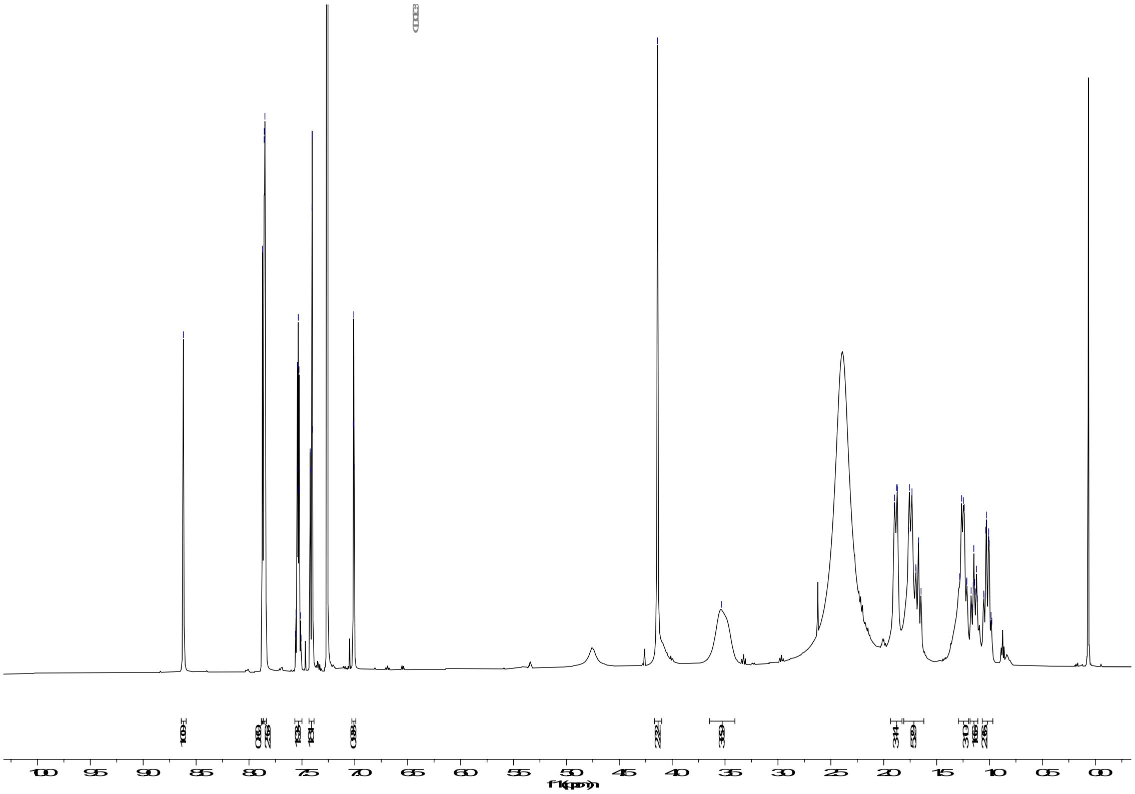

Figure S109. ^{1}H NMR spectrum (500 MHz, $CDCl_3$) of compound **104**.

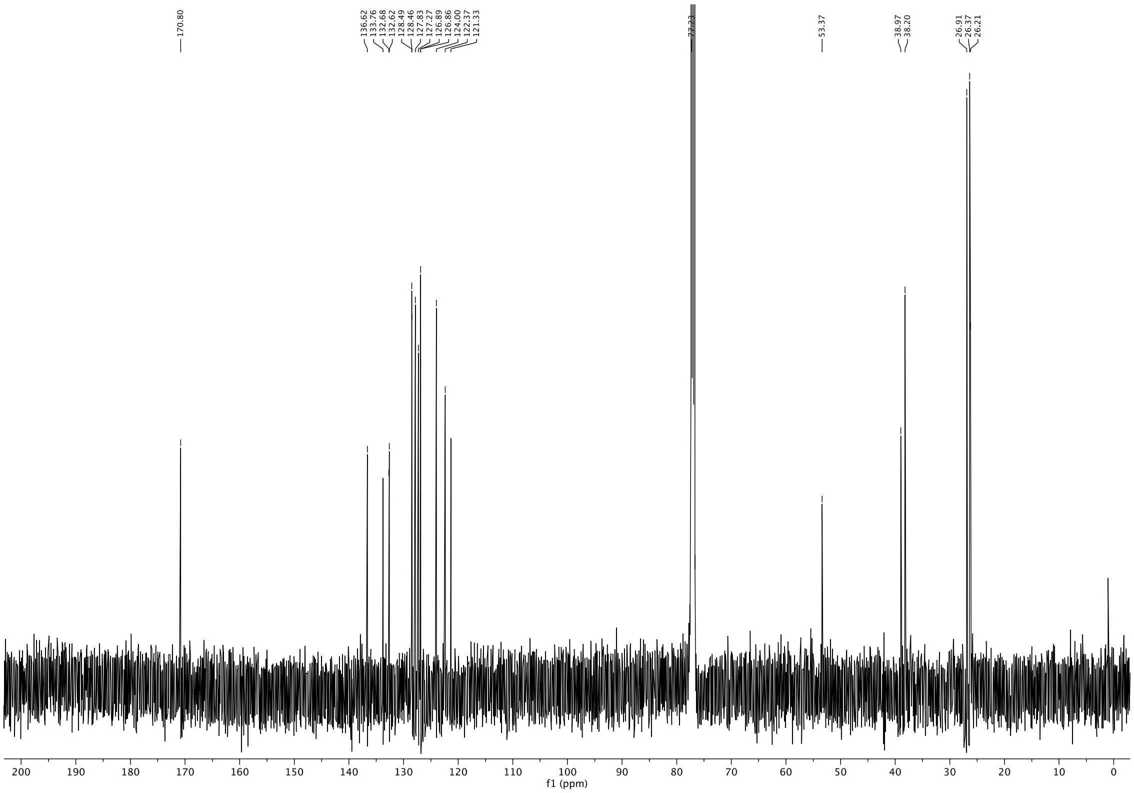

Figure S110. ^{13}C NMR spectrum (126 MHz, $CDCl_3$) of compound **104**.

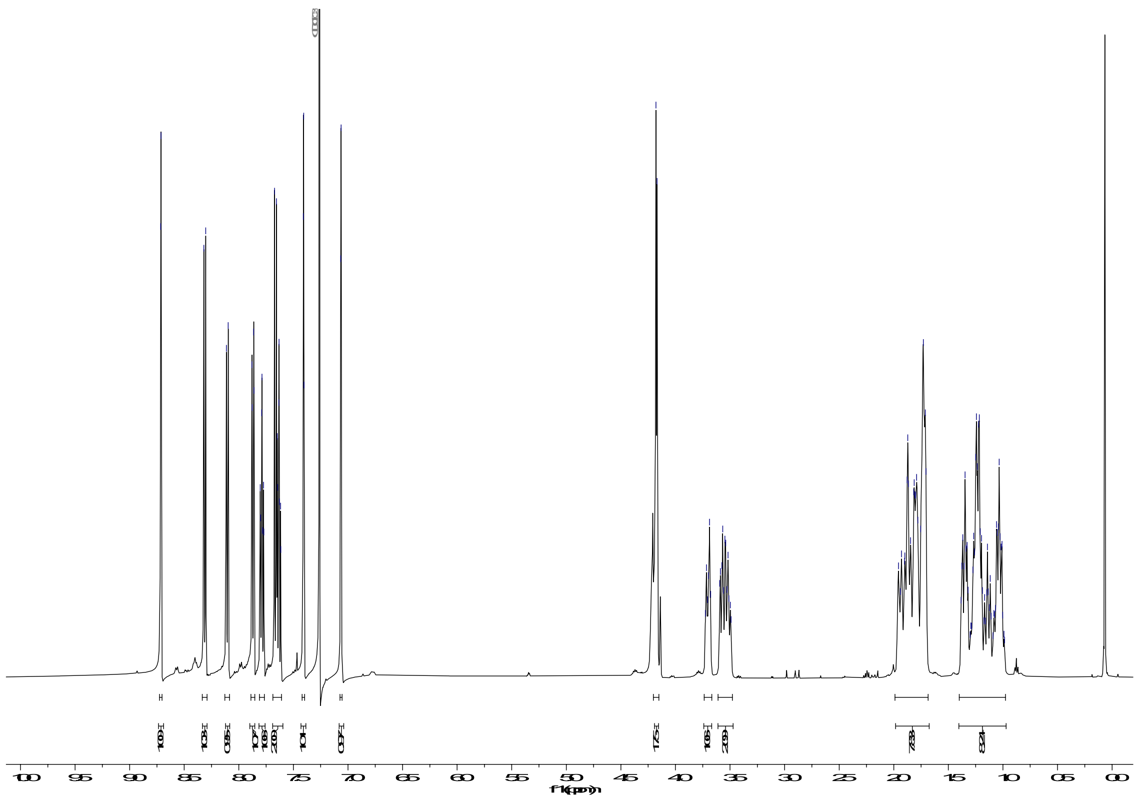

Figure S111. ^{1}H NMR spectrum (500 MHz, $CDCl_3$) of compound **105**.

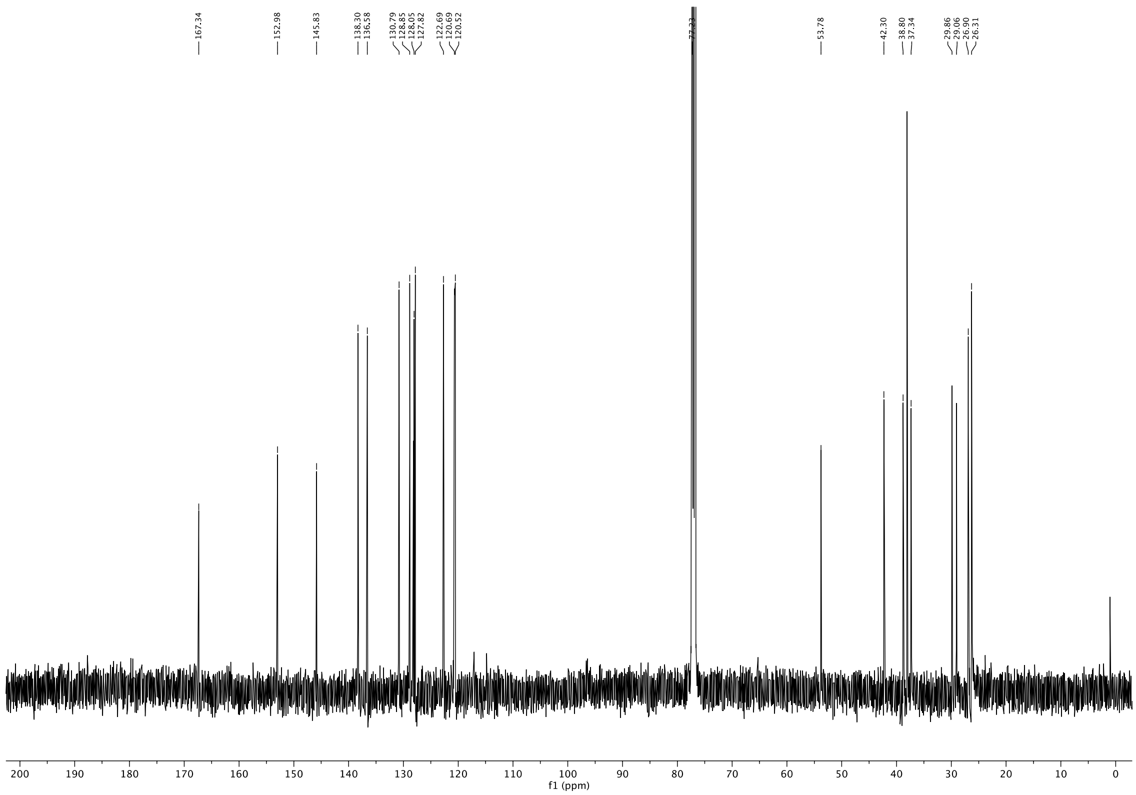

Figure S112. ^{13}C NMR spectrum (126 MHz, $CDCl_3$) of compound **105**.

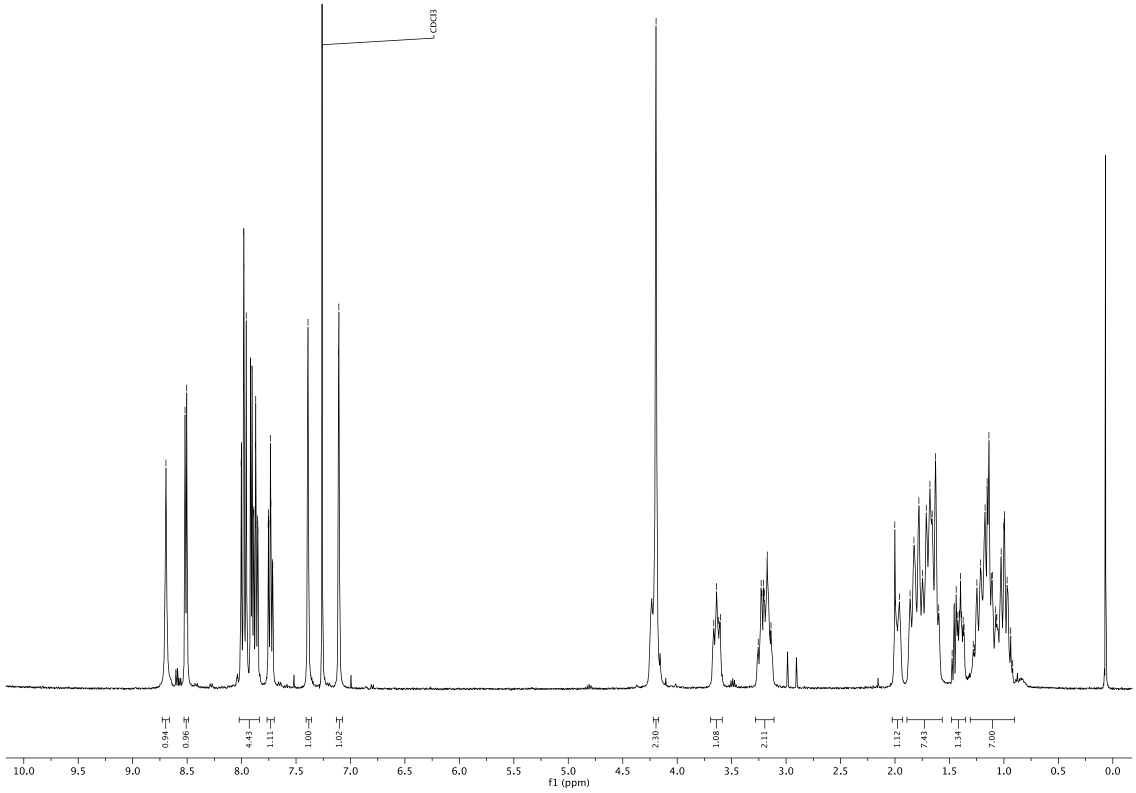

Figure S113. ^{1}H NMR spectrum (400 MHz, $CDCl_3$) of compound **106**.

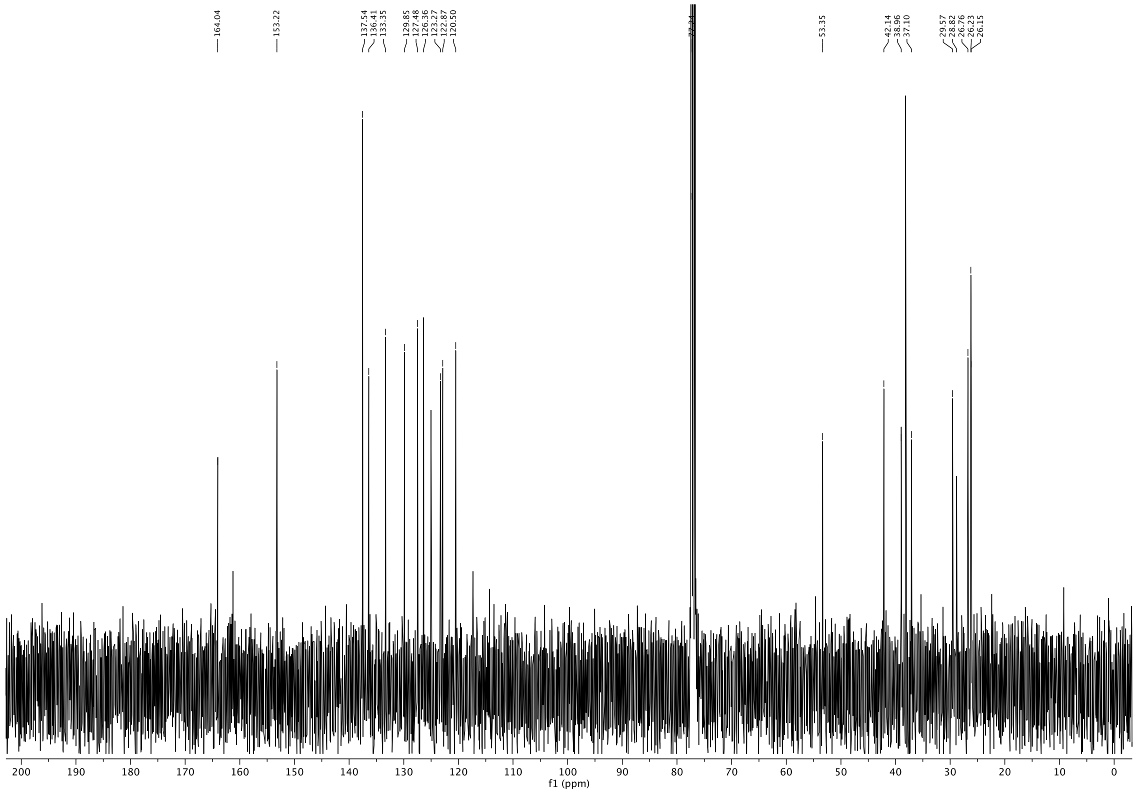

Figure S114. ^{13}C NMR spectrum (101 MHz, $CDCl_3$) of compound **106**.

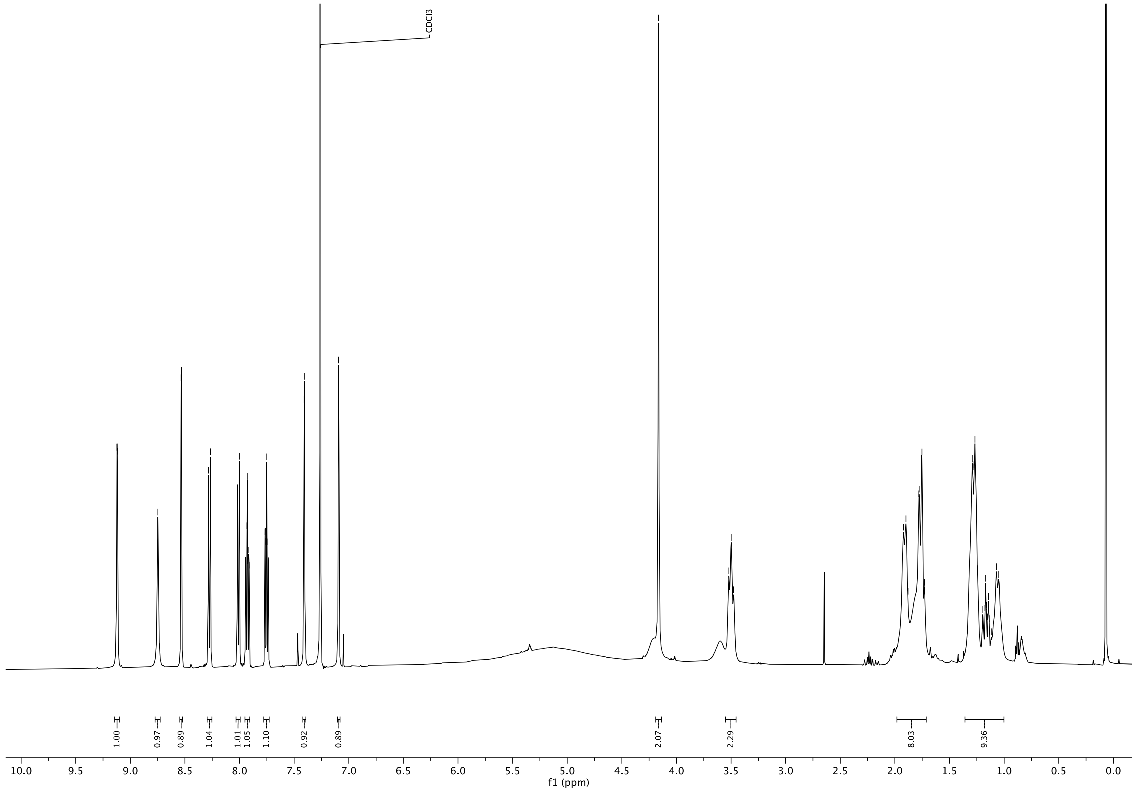

Figure S115. ^{1}H NMR spectrum (500 MHz, $CDCl_3$) of compound **107**.

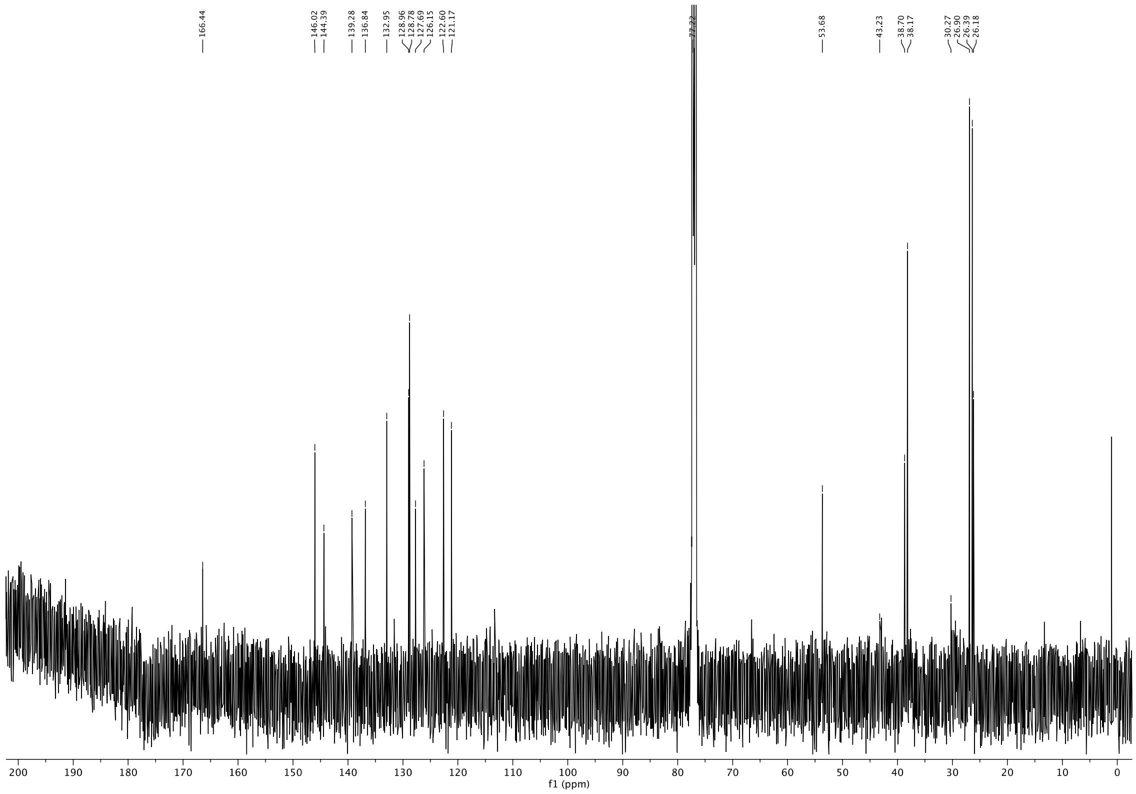

Figure S116. ^{13}C NMR spectrum (126 MHz, $CDCl_3$) of compound **107**.

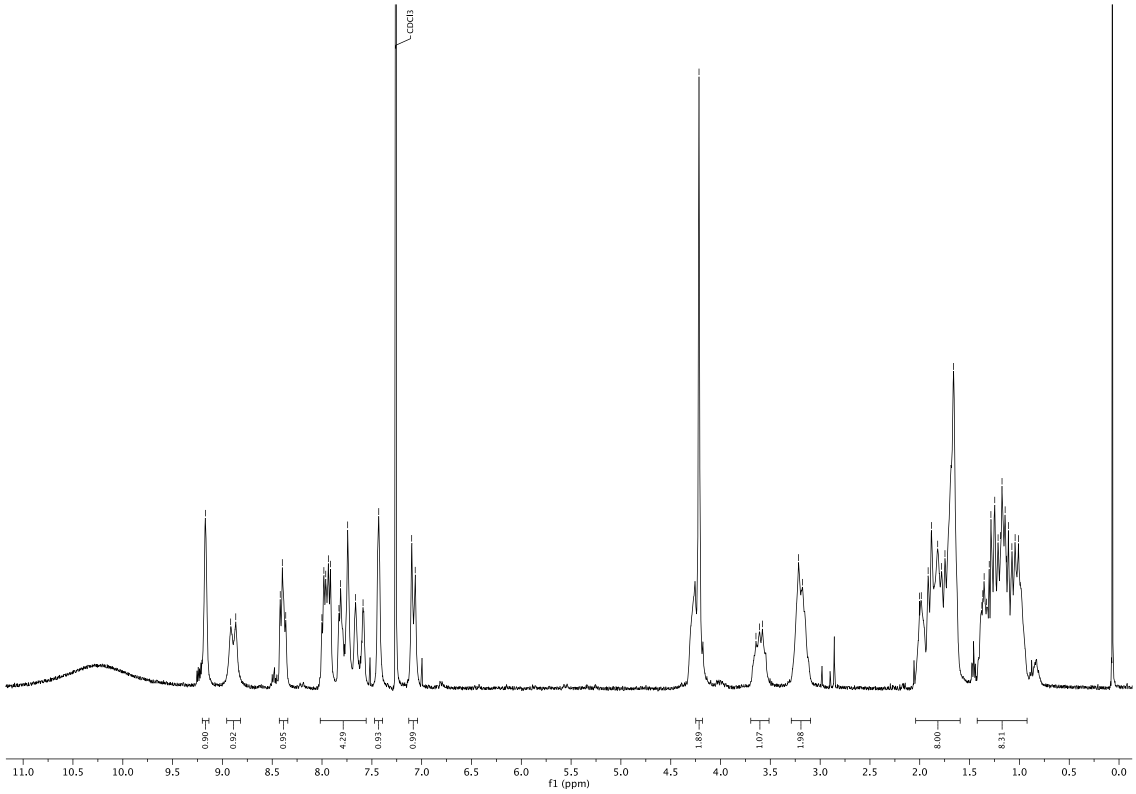

Figure S117. ^{1}H NMR spectrum (400 MHz, $CDCl_3$) of compound **108**.

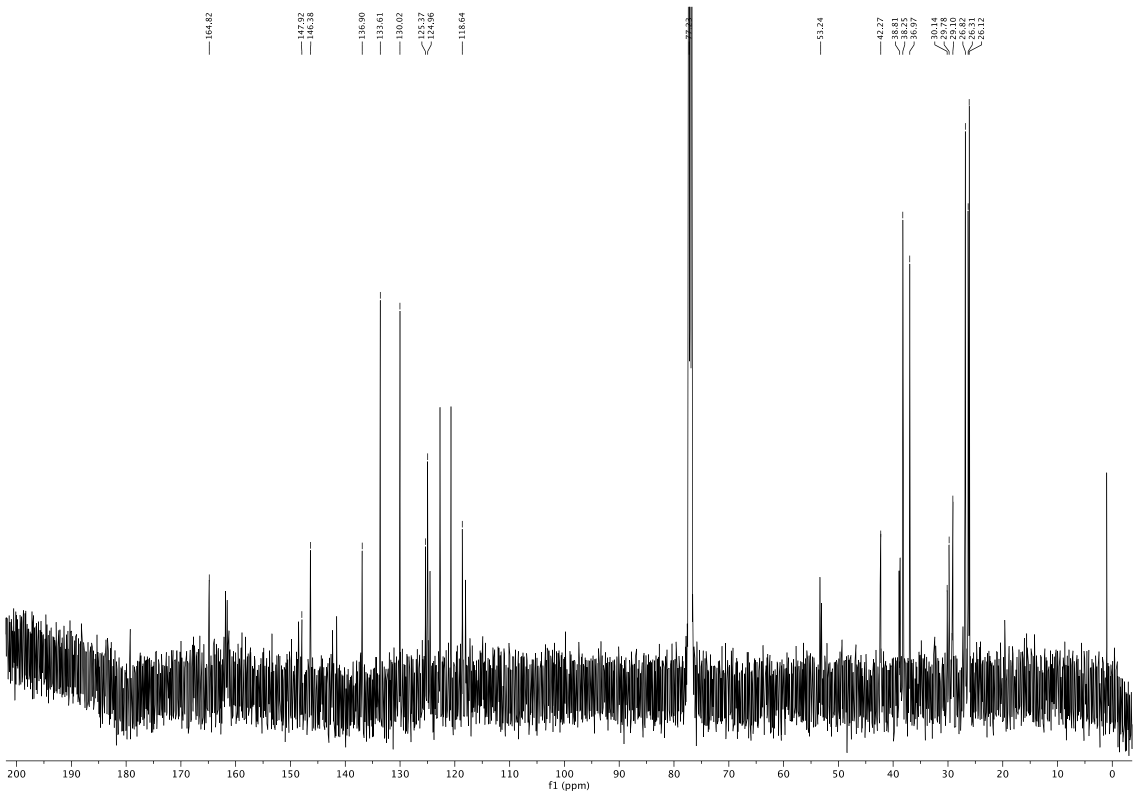

Figure S118. ^{13}C NMR spectrum (126 MHz, $CDCl_3$) of compound **108**.

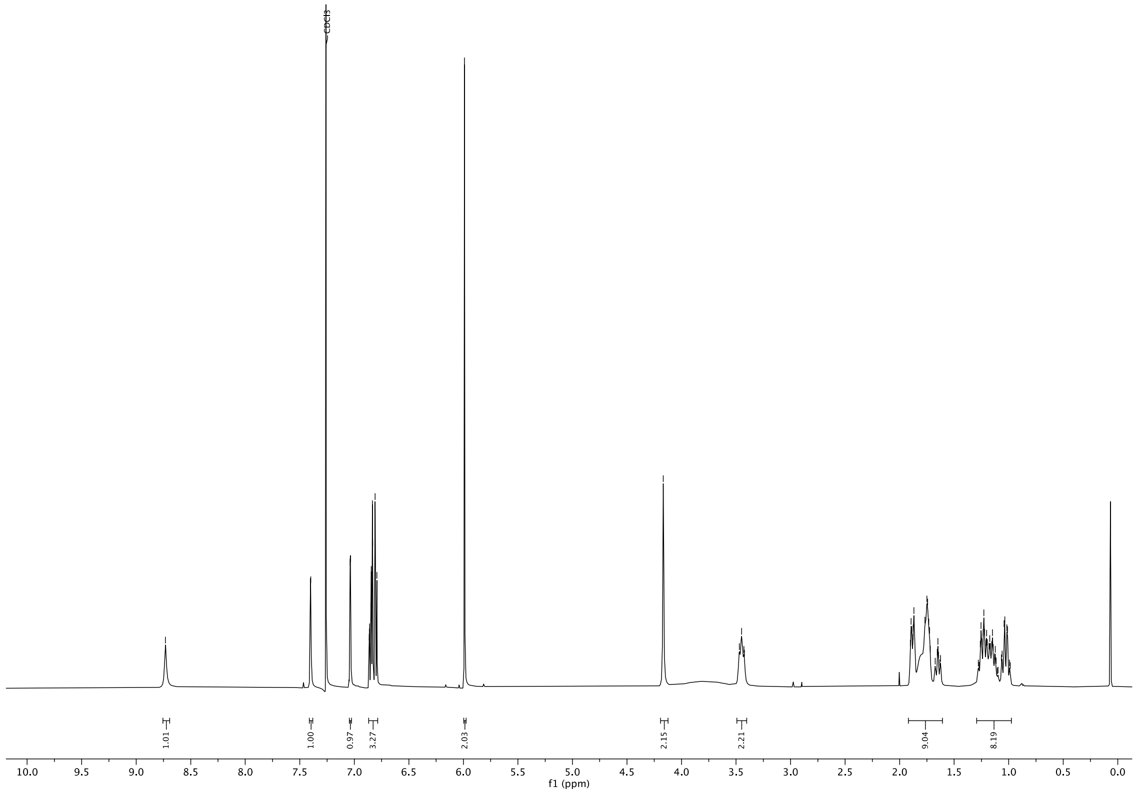

Figure S119. ^{1}H NMR spectrum (500 MHz, $CDCl_3$) of compound **109**.

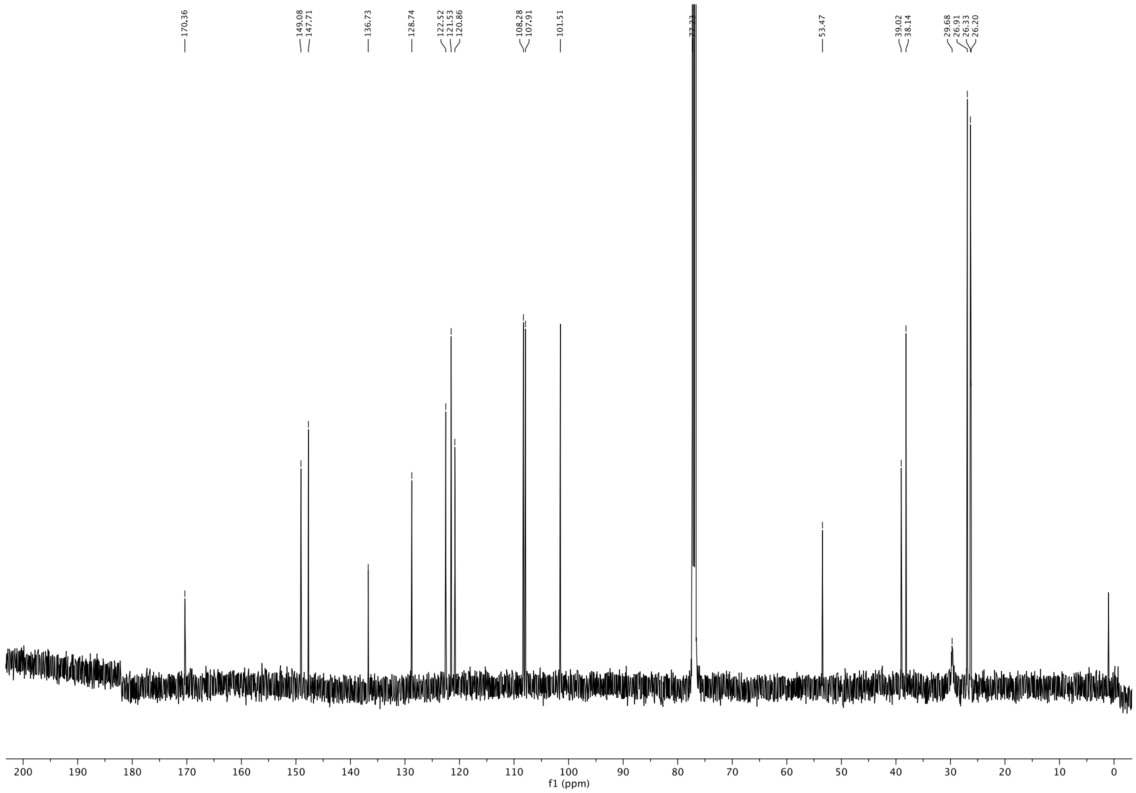

Figure S120. ^{13}C NMR spectrum (126 MHz, $CDCl_3$) of compound **109**.

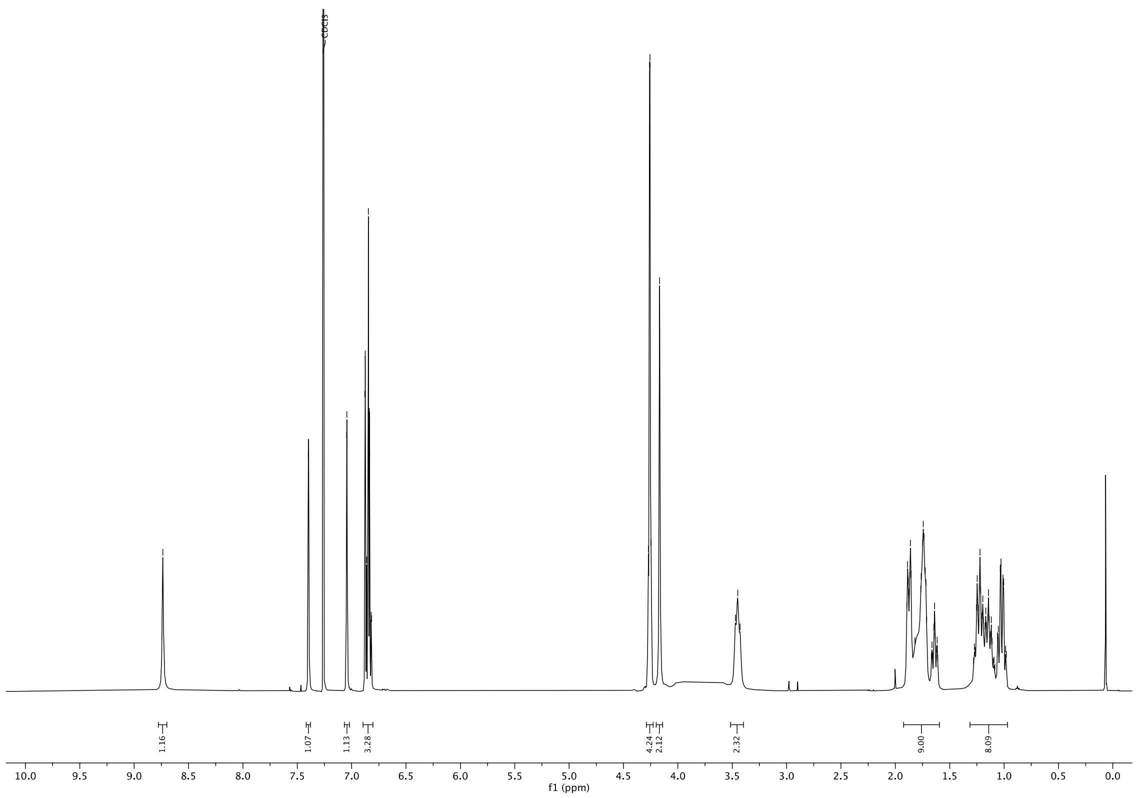

Figure S121. ^{1}H NMR spectrum (500 MHz, $CDCl_3$) of compound **110**.

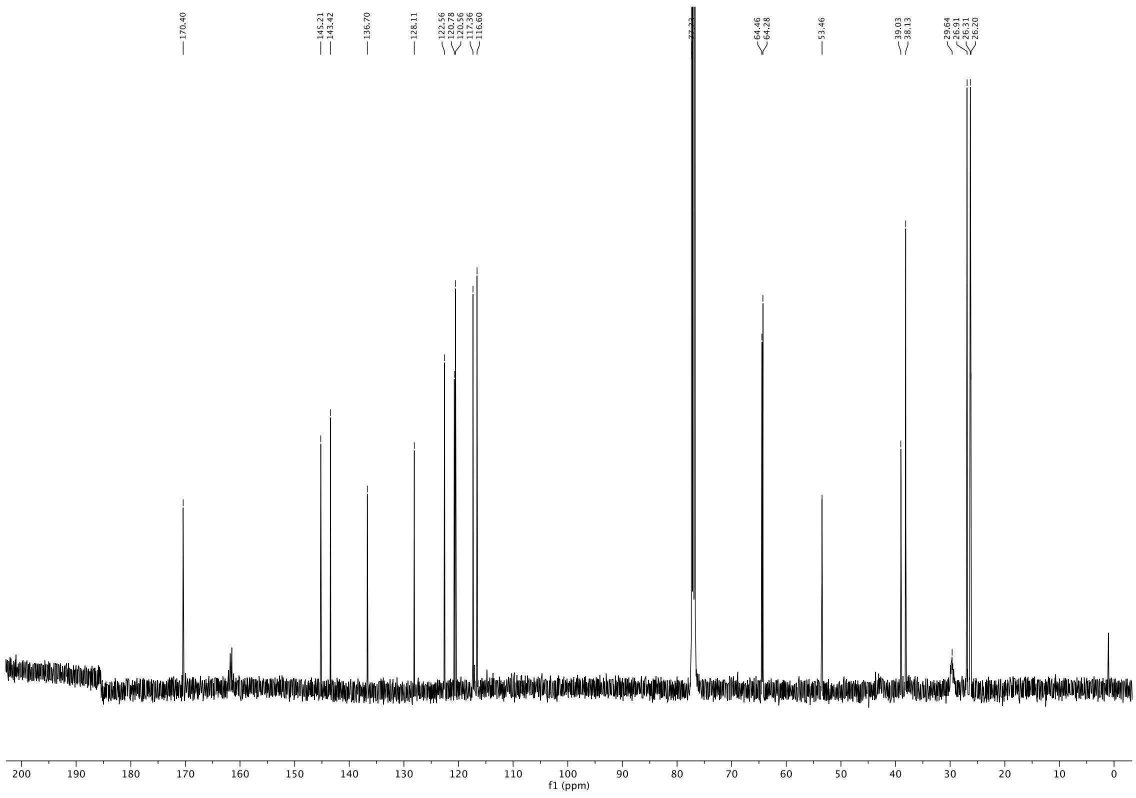

Figure S122. ^{13}C NMR spectrum (126 MHz, $CDCl_3$) of compound **110**.

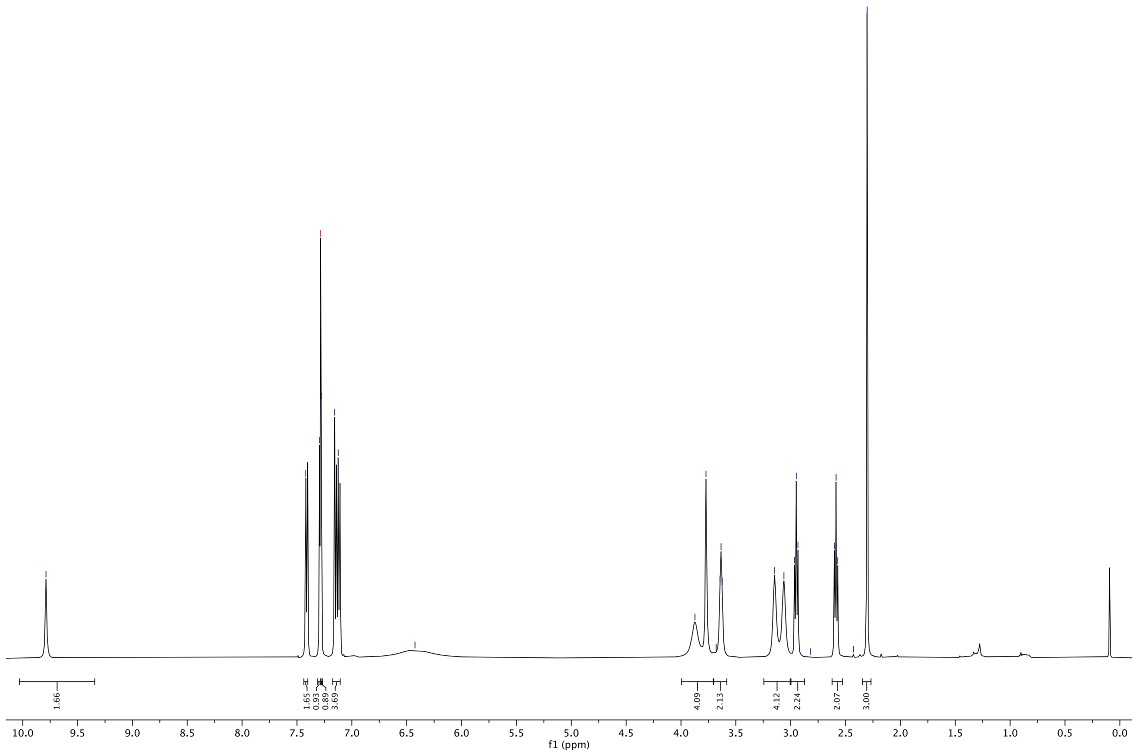

Figure S123. ^{1}H NMR spectrum (500 MHz, $CDCl_3$) of compound **115**.

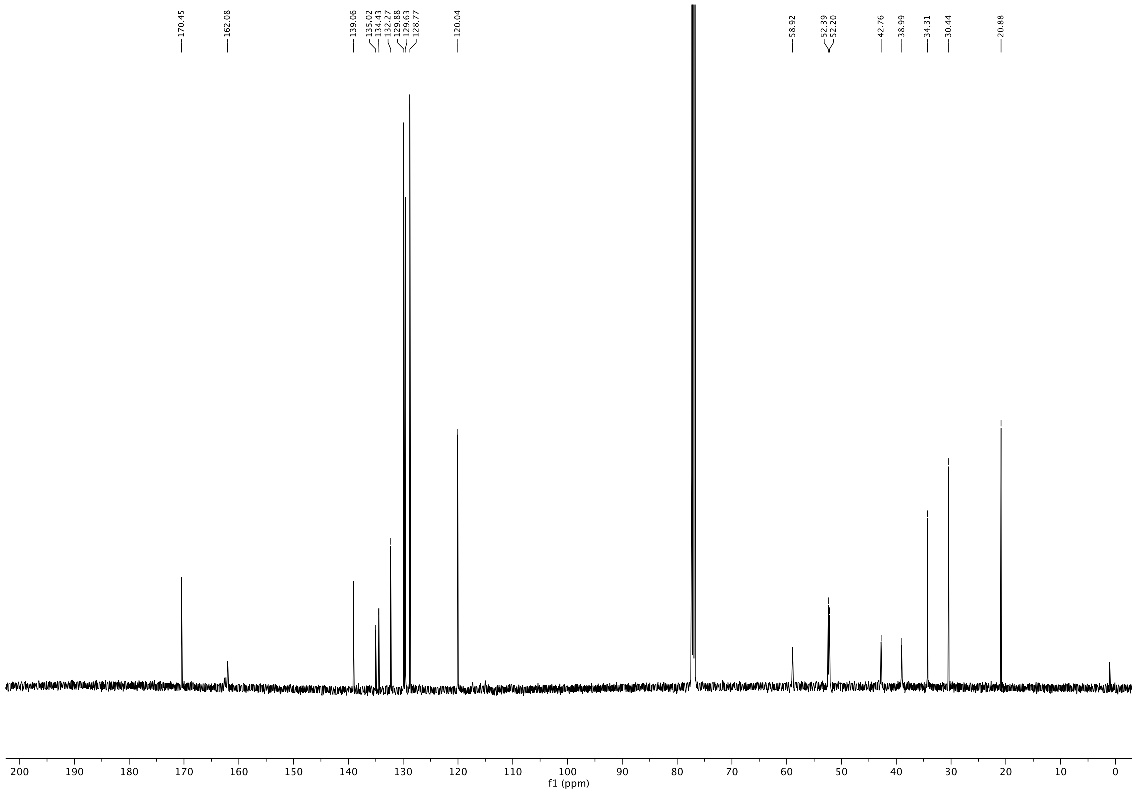

Figure S124. ^{13}C NMR spectrum (126 MHz, $CDCl_3$) of compound **115**.

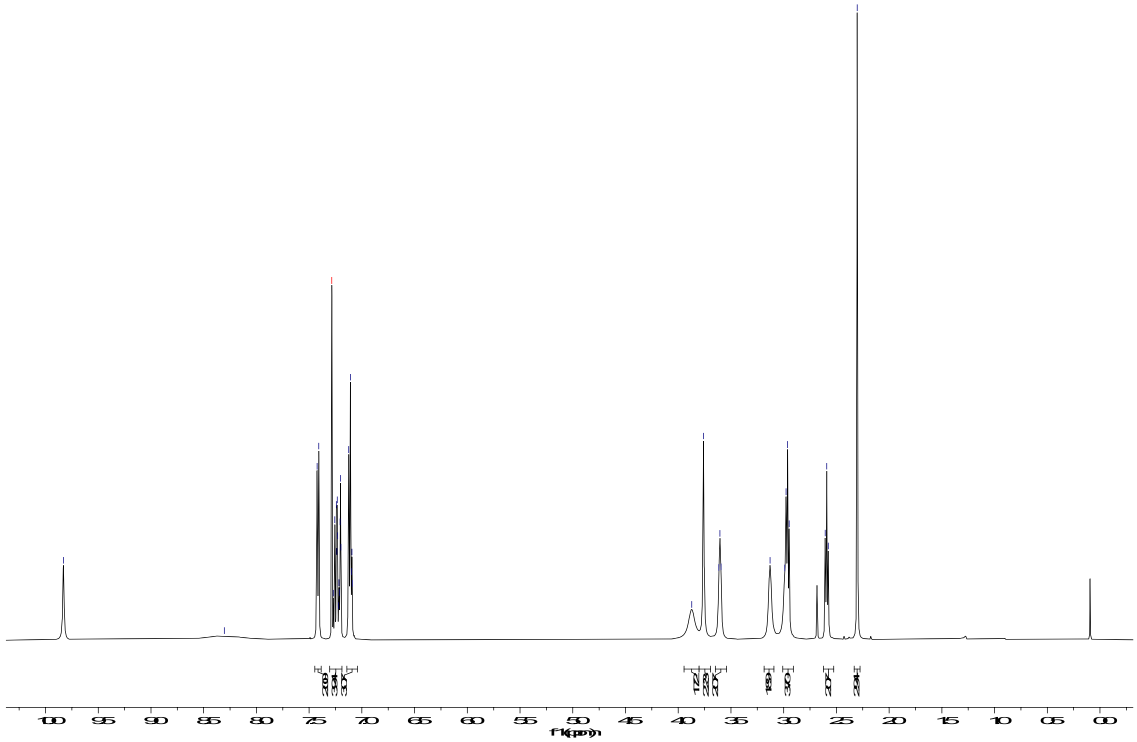

Figure S125. ^{1}H NMR spectrum (500 MHz, $CDCl_3$) of compound **116**.

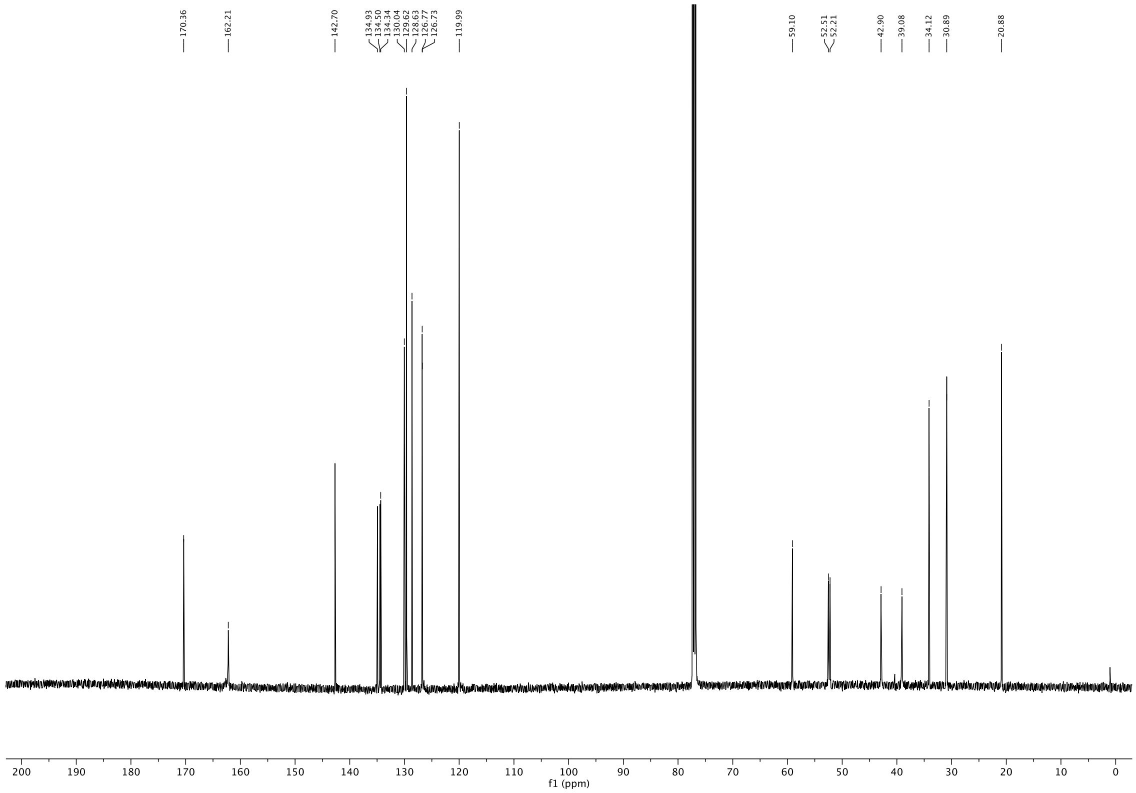

Figure S126. ^{13}C NMR spectrum (126 MHz, $CDCl_3$) of compound **116**.

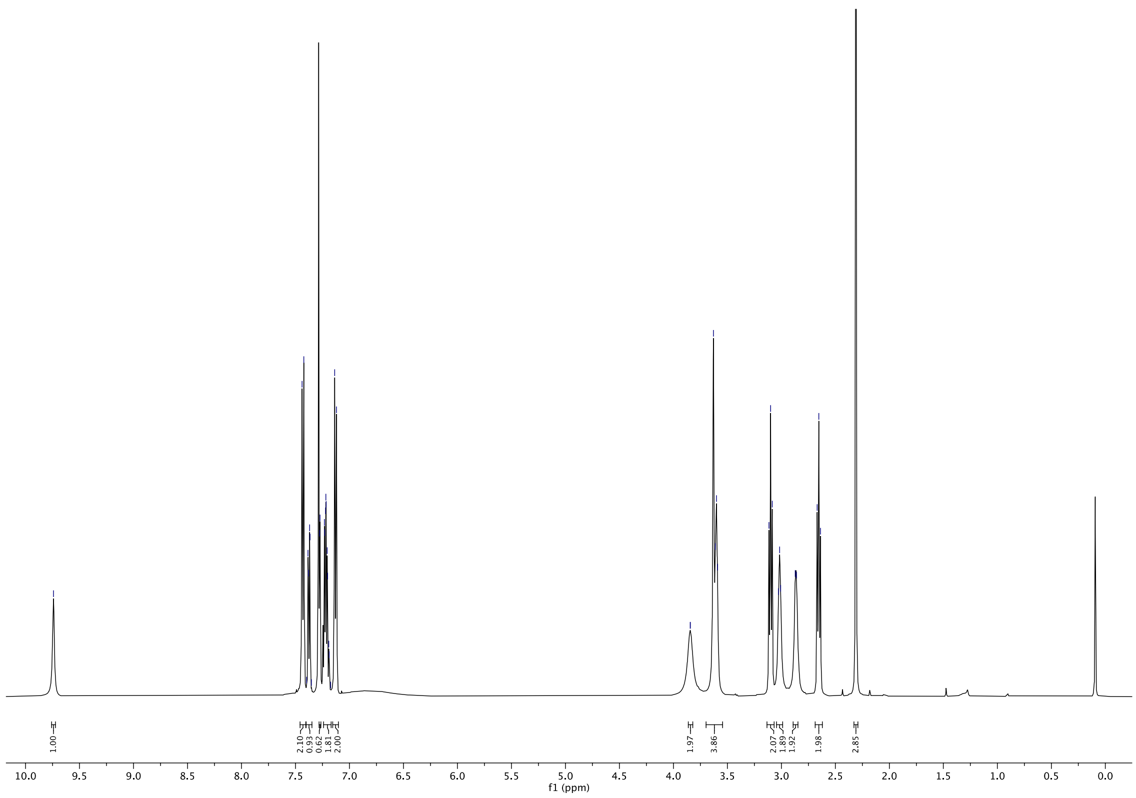

Figure S127. ^{1}H NMR spectrum (500 MHz, $CDCl_3$) of compound **117**.

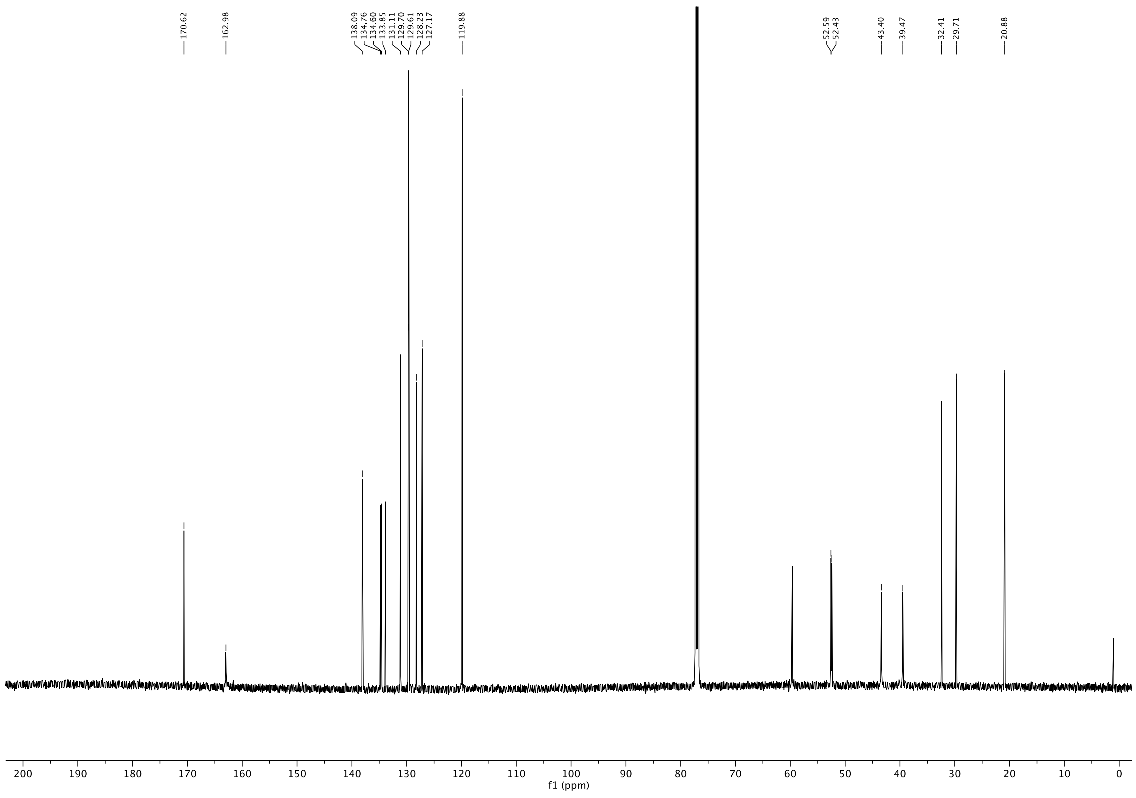

Figure S128. ^{13}C NMR spectrum (126 MHz, $CDCl_3$) of compound **117**.

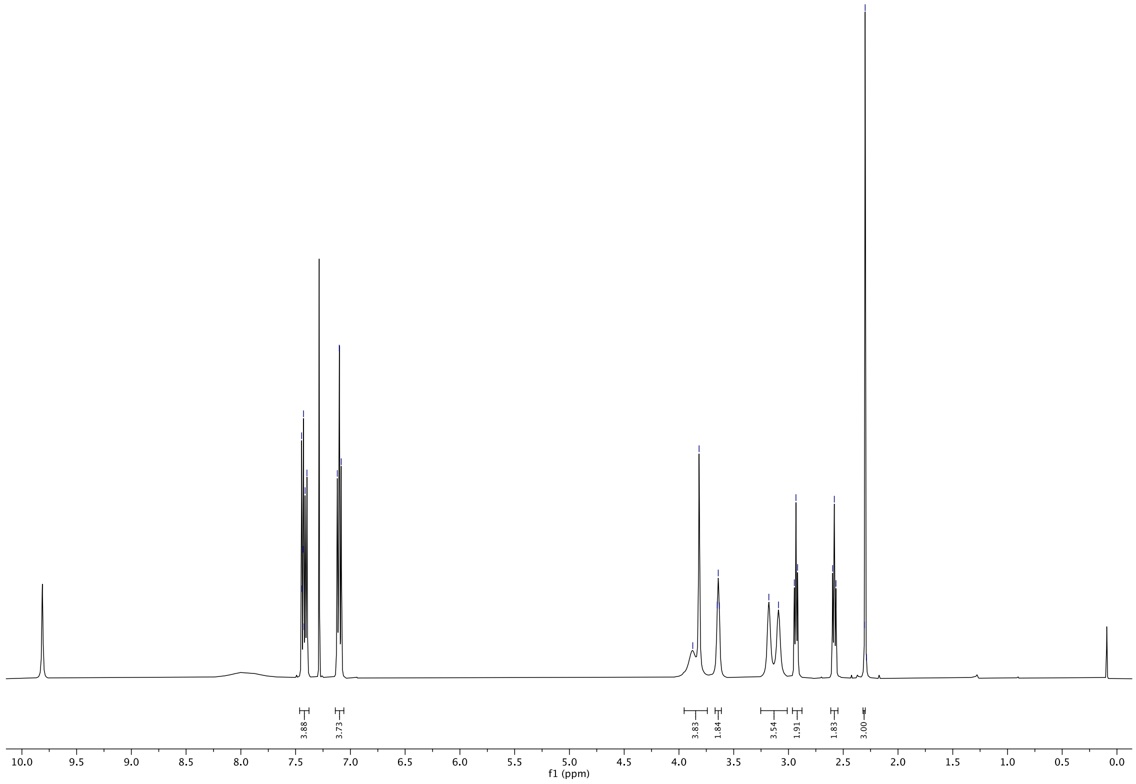

Figure S129. ^{1}H NMR spectrum (500 MHz, $CDCl_3$) of compound **118**.

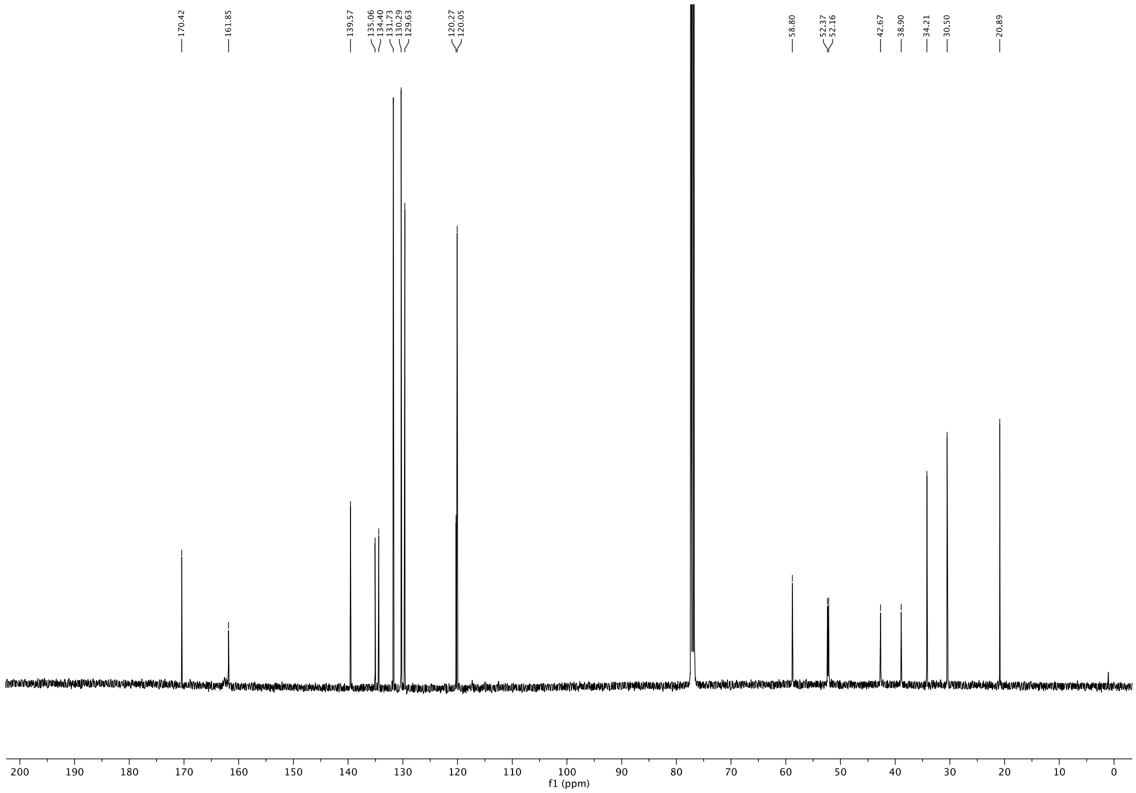

Figure S130. ^{13}C NMR spectrum (126 MHz, $CDCl_3$) of compound **118**.

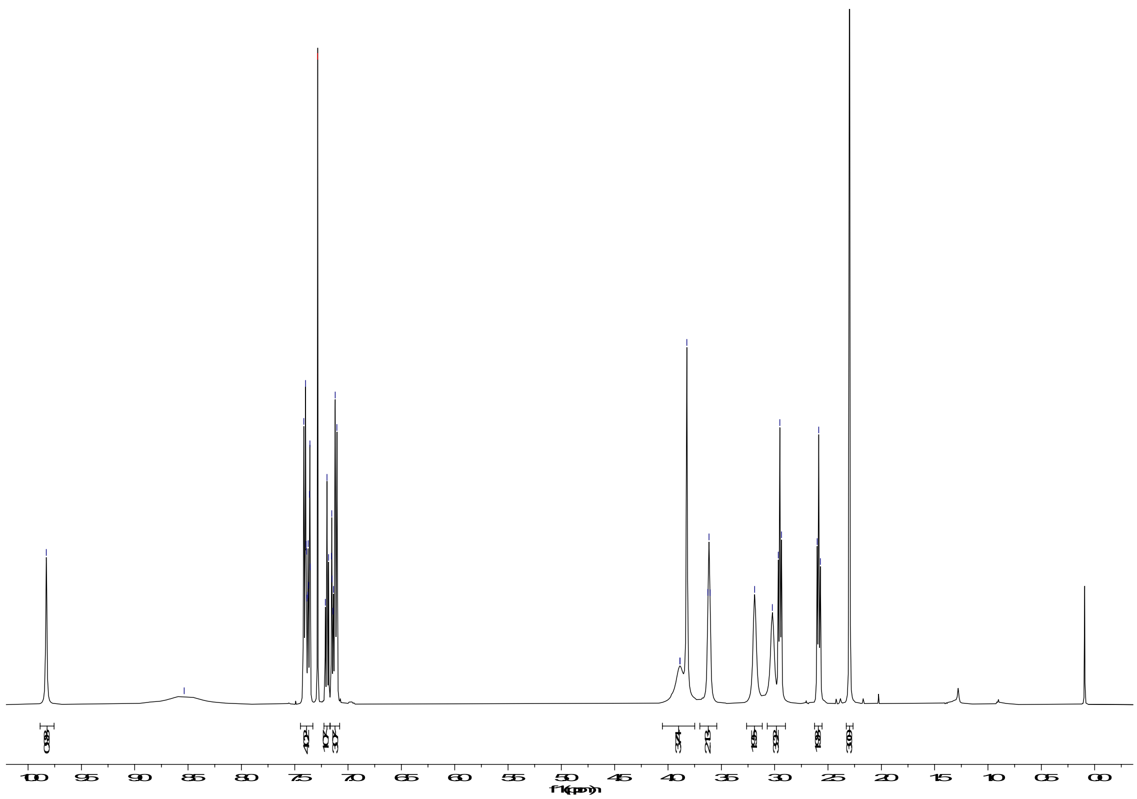

Figure S131. ^{1}H NMR spectrum (500 MHz, $CDCl_3$) of compound **119**.

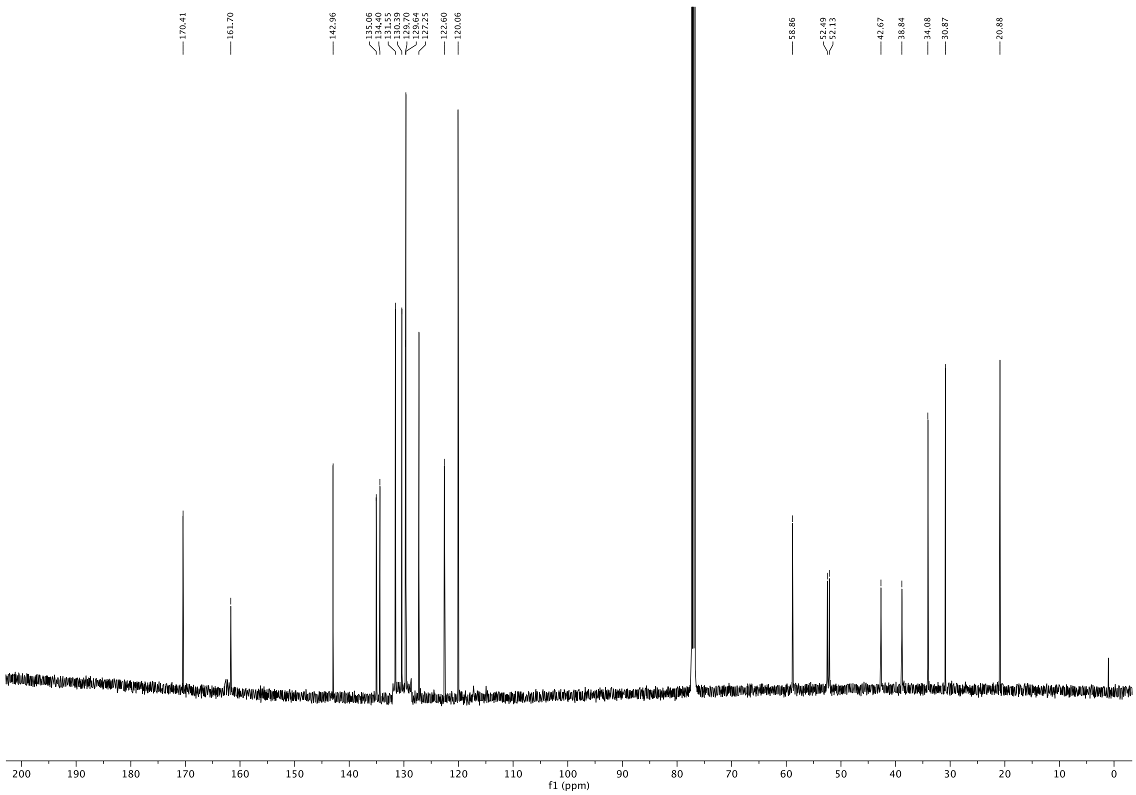

Figure S132. ^{13}C NMR spectrum (126 MHz, $CDCl_3$) of compound **119**.

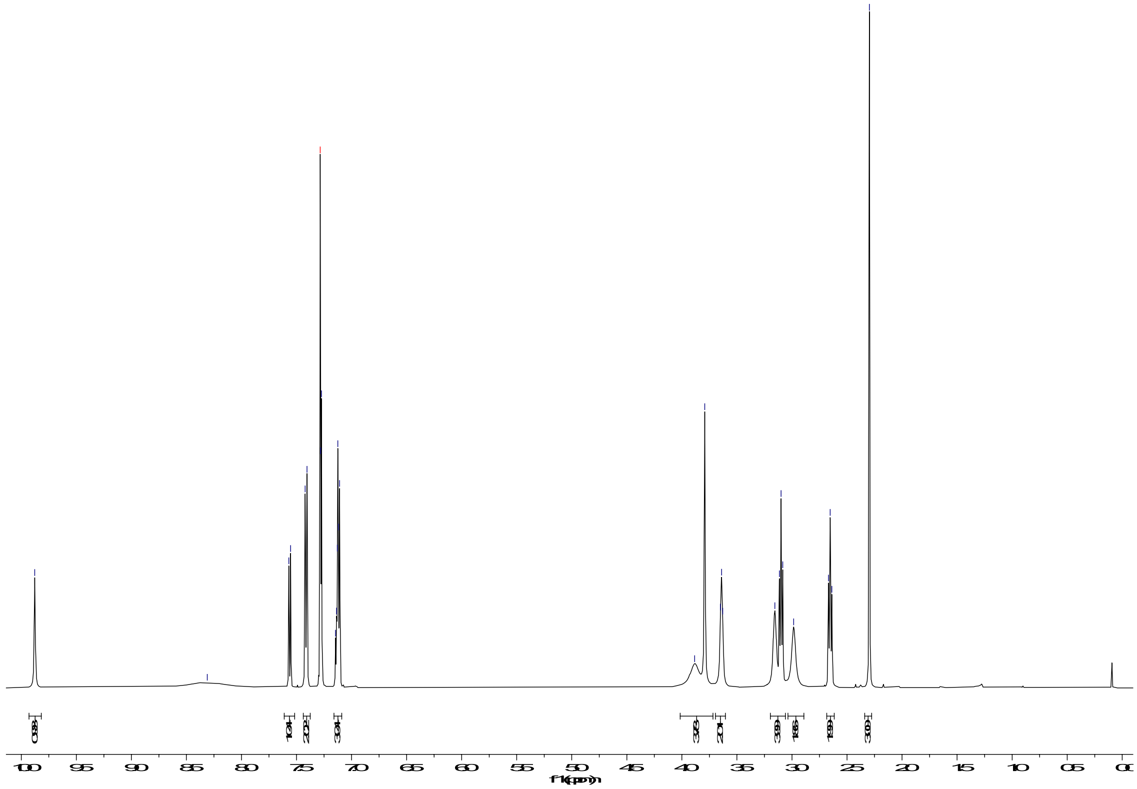

Figure S133. ^{1}H NMR spectrum (500 MHz, $CDCl_3$) of compound **120**.

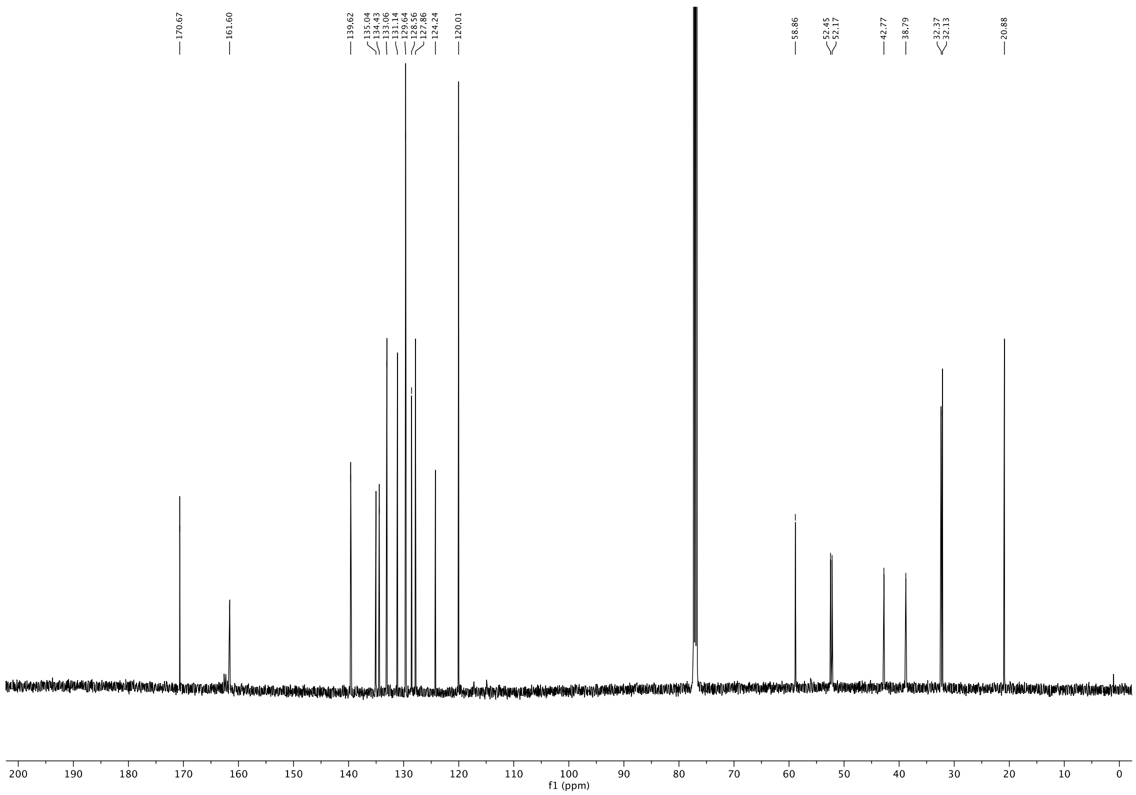

Figure S134. ^{13}C NMR spectrum (126 MHz, $CDCl_3$) of compound **120**.

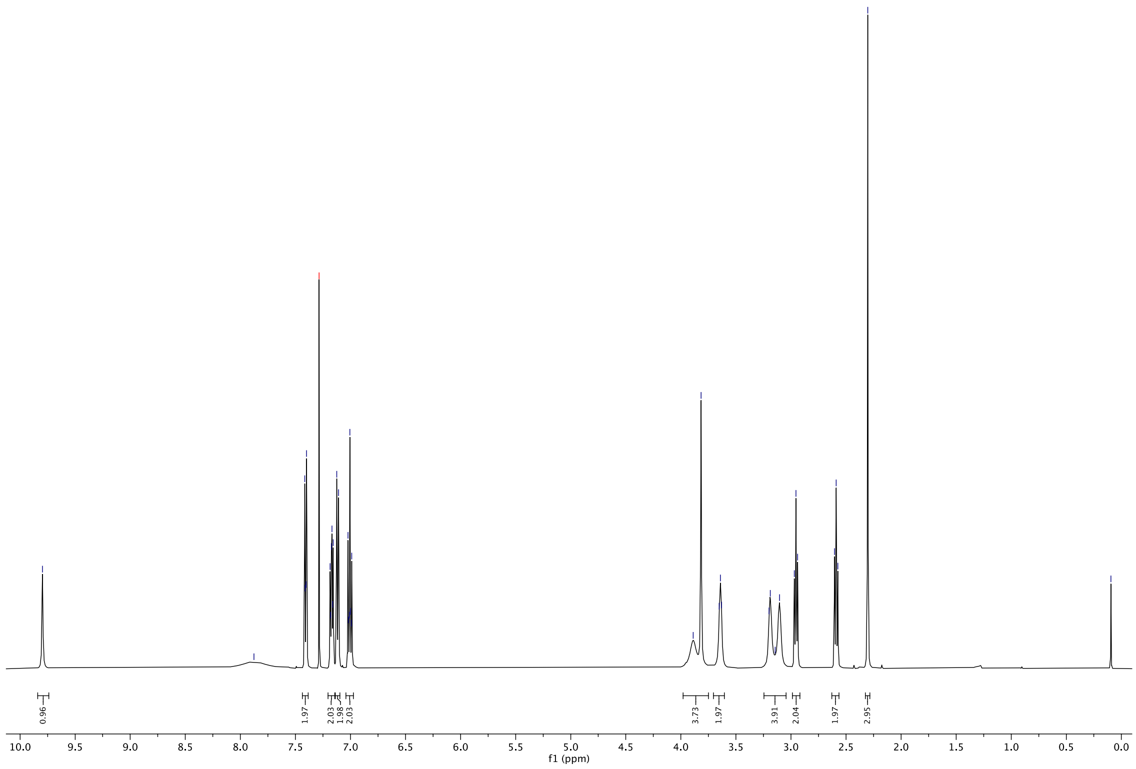

Figure S135. ^{1}H NMR spectrum (500 MHz, $CDCl_3$) of compound **121**.

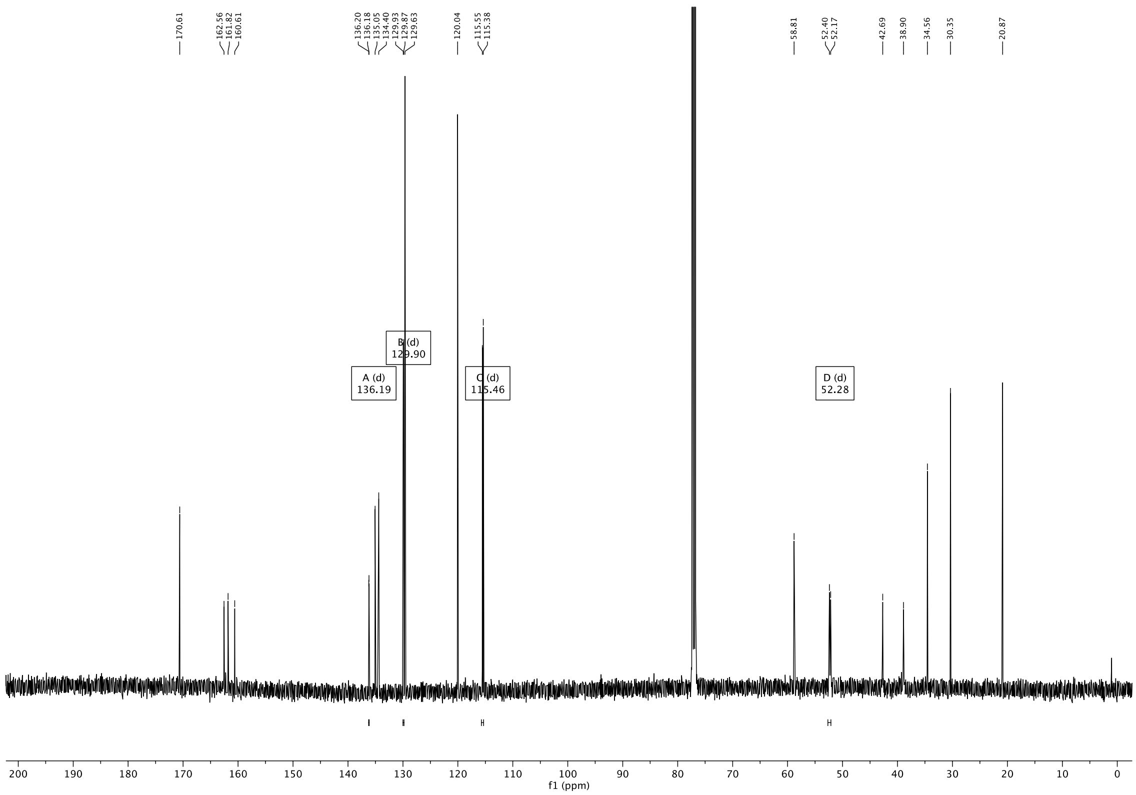

Figure S136. ^{13}C NMR spectrum (126 MHz, $CDCl_3$) of compound **121**.

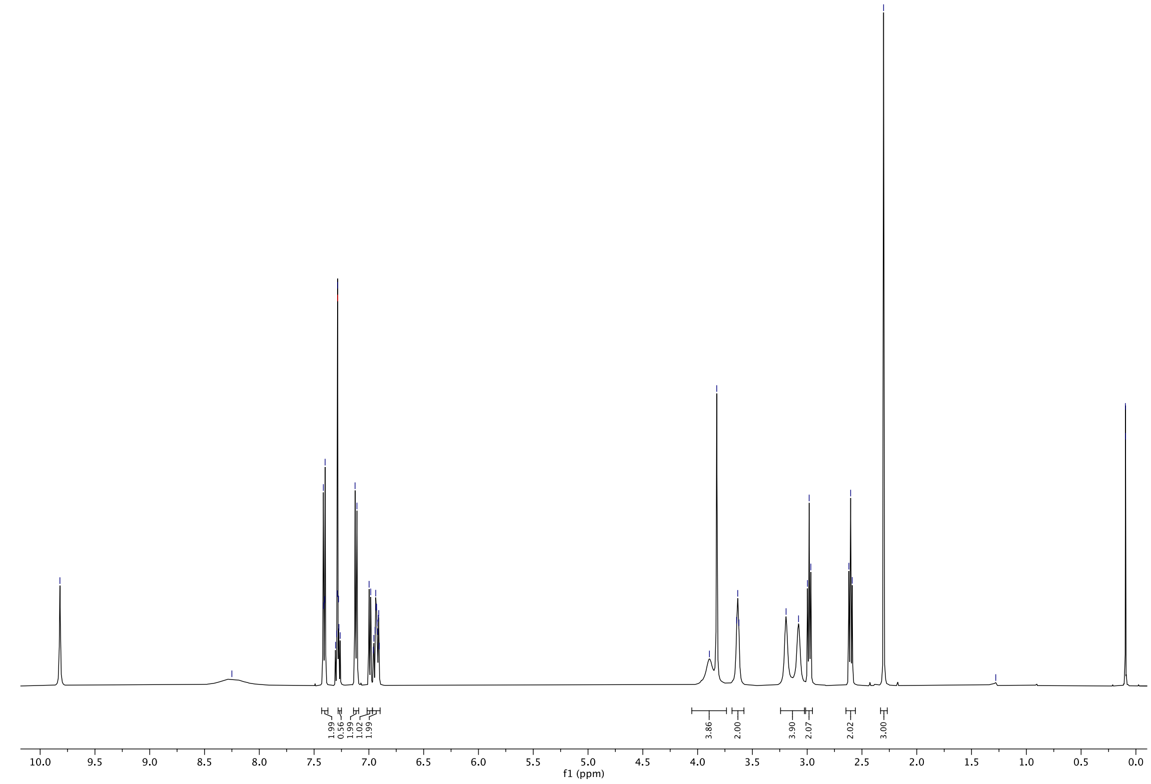

Figure S137. ^{1}H NMR spectrum (500 MHz, $CDCl_3$) of compound **122**.

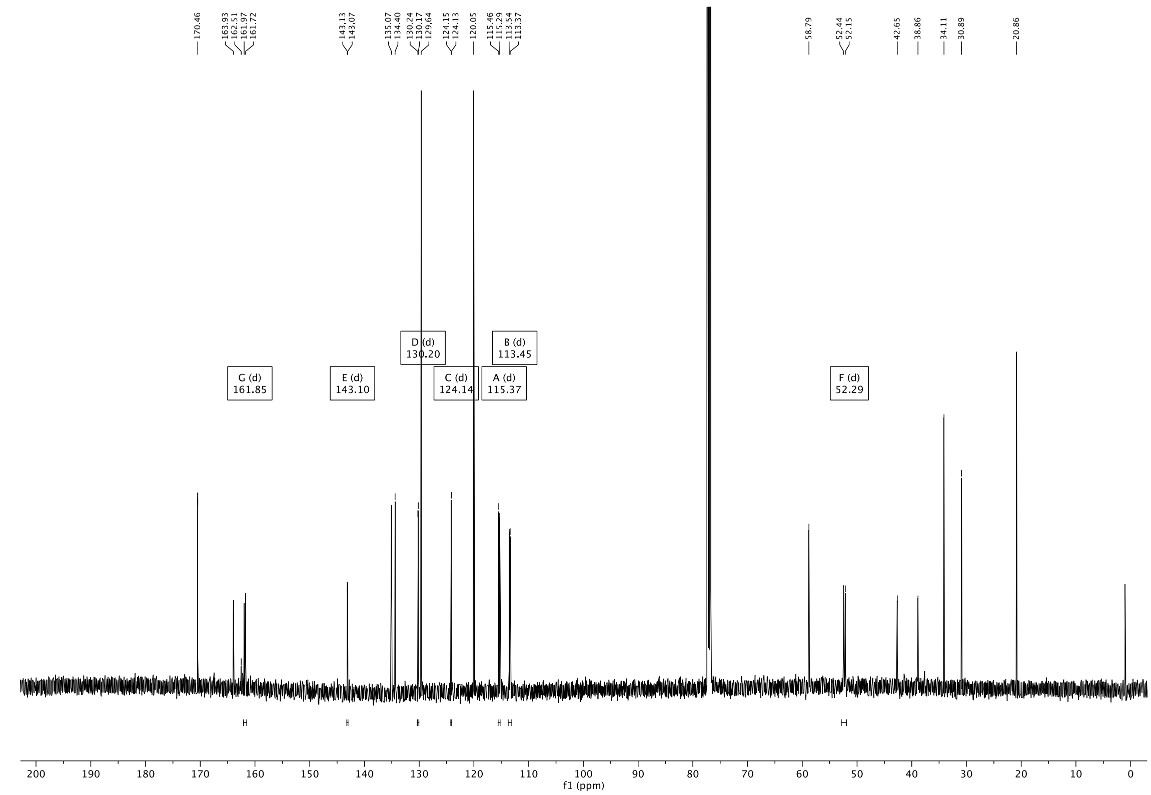

Figure S138. ^{13}C NMR spectrum (126 MHz, $CDCl_3$) of compound **122**.

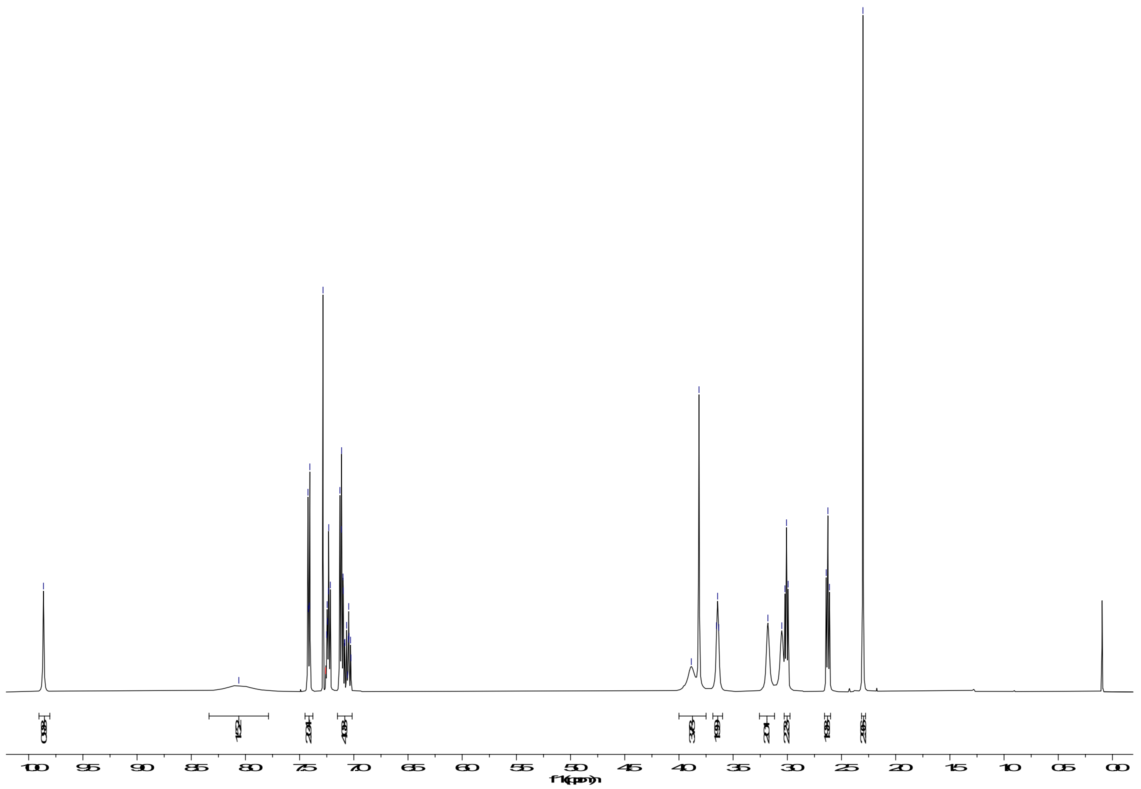

Figure S139. ^{1}H NMR spectrum (500 MHz, $CDCl_3$) of compound **123**.

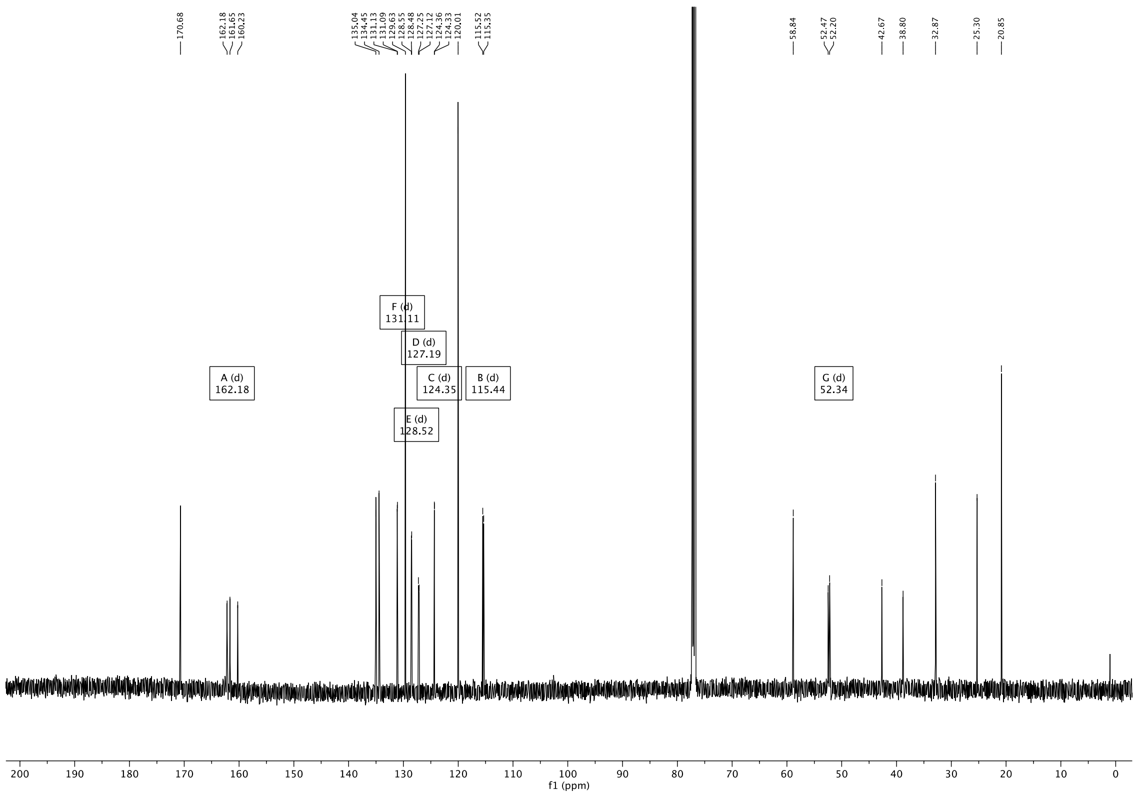

Figure S140. ^{13}C NMR spectrum (126 MHz, $CDCl_3$) of compound **123**.

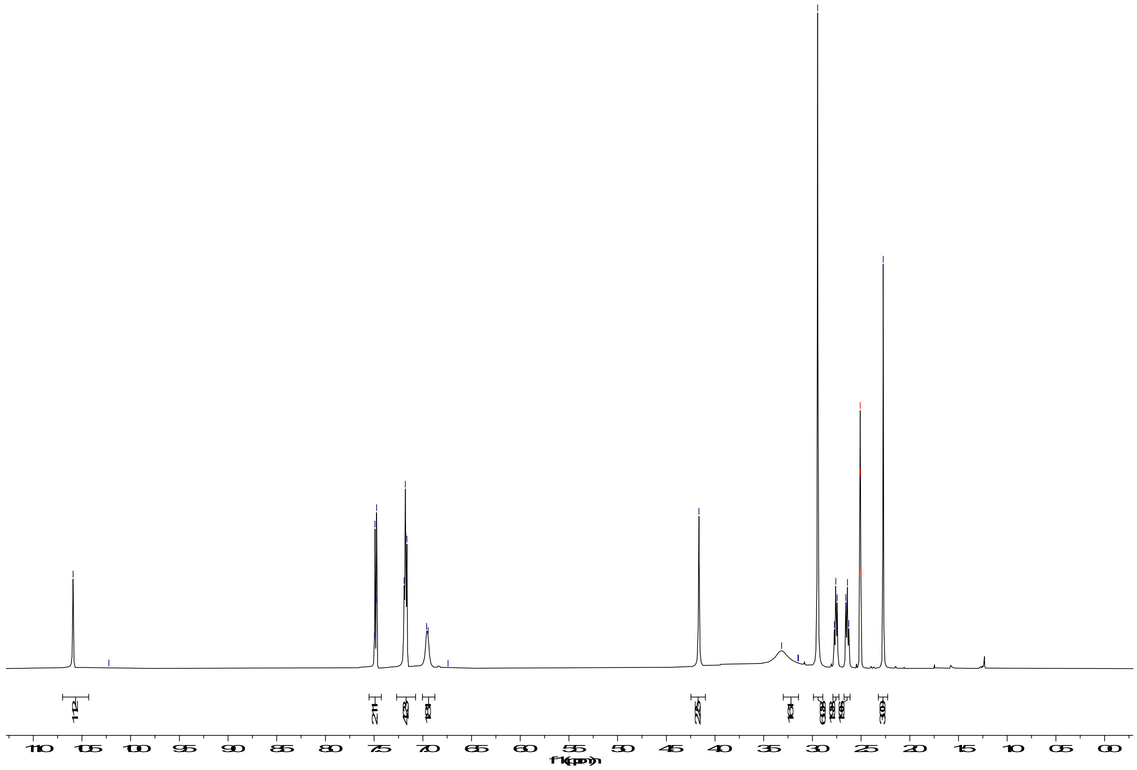

Figure S141. ^{1}H NMR spectrum (500 MHz, DMSO-d_6) of compound **124**.

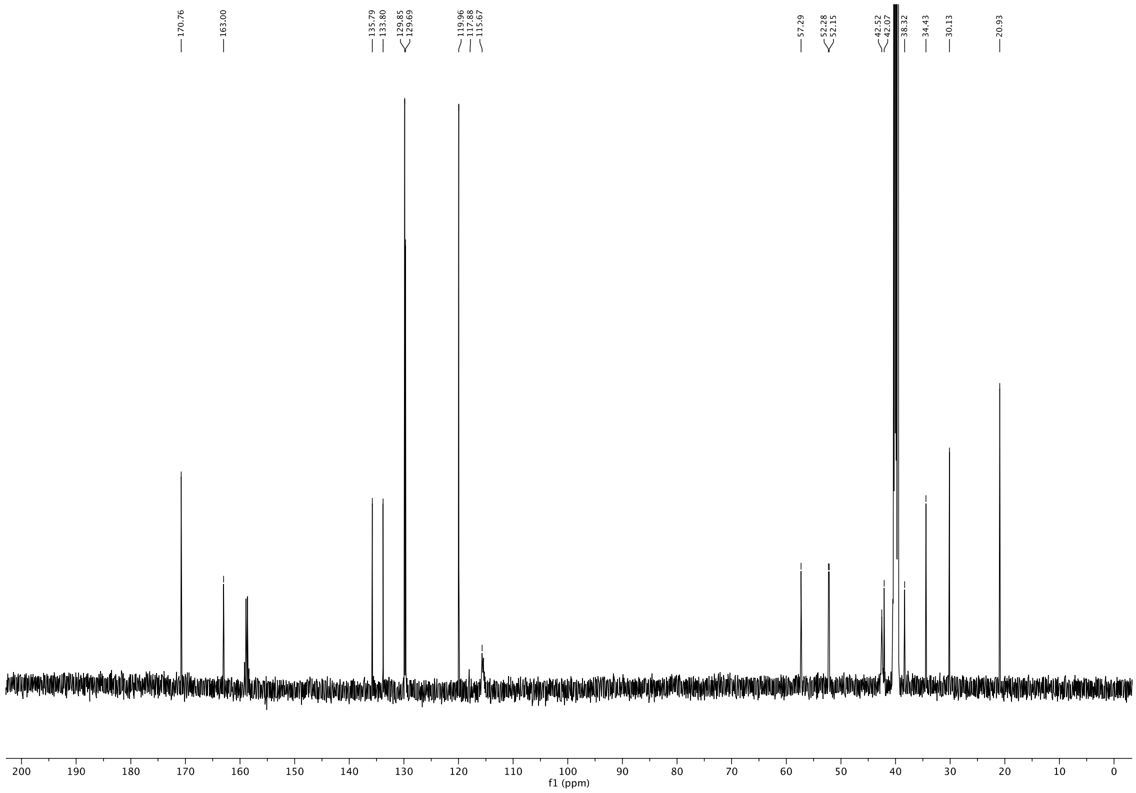

Figure S142. ^{13}C NMR spectrum (126 MHz, DMSO-d_6) of compound **124**.

RP-HPLC chromatograms of 21-37, 52-77, 80-84, 93-110, and 115-124

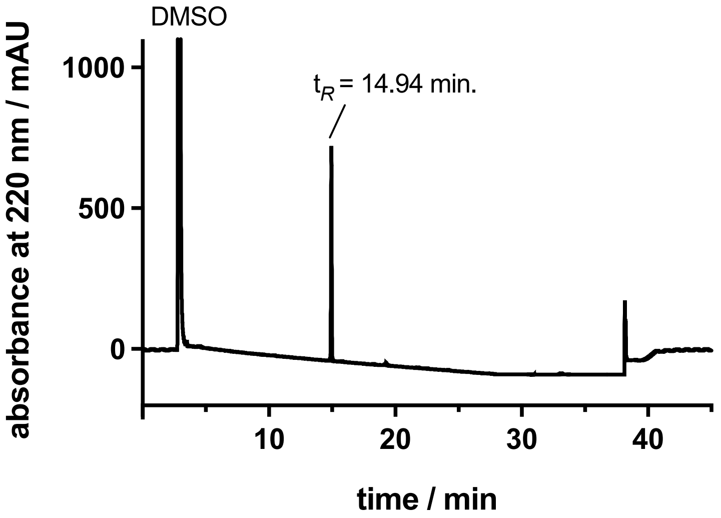

Figure S143. RP-HPLC analysis (purity control) of compound **21** (97 %, 220 nm).

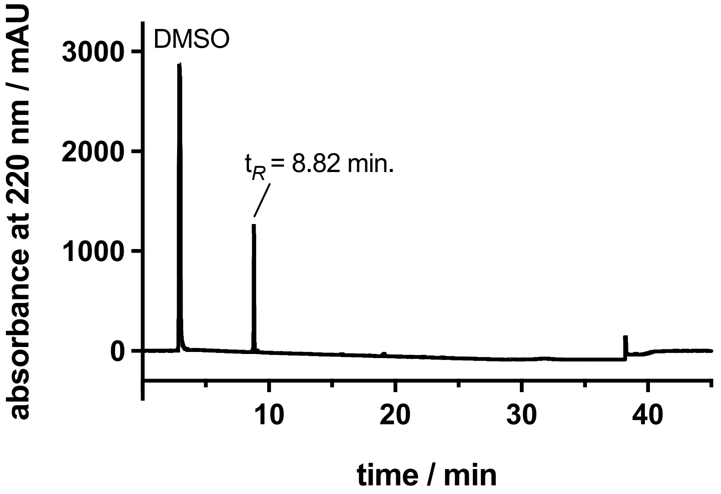

Figure S144. RP-HPLC analysis (purity control) of compound **22** (97 %, 220 nm).

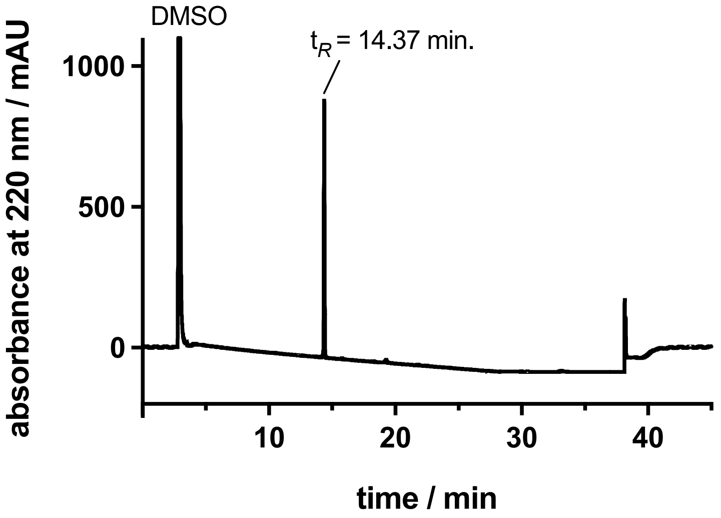

Figure S145. RP-HPLC analysis (purity control) of compound **23** (> 99 %, 220 nm).

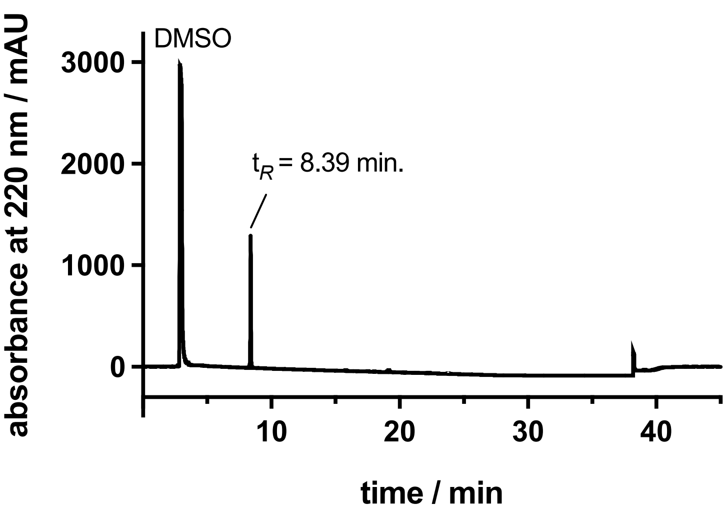

Figure S146. RP-HPLC analysis (purity control) of compound **24** (> 99 %, 220 nm).

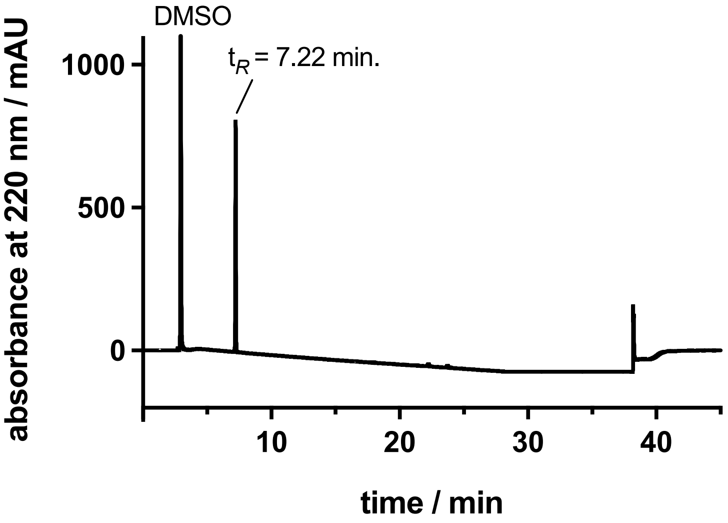

Figure S147. RP-HPLC analysis (purity control) of compound **25** (> 99 %, 220 nm).

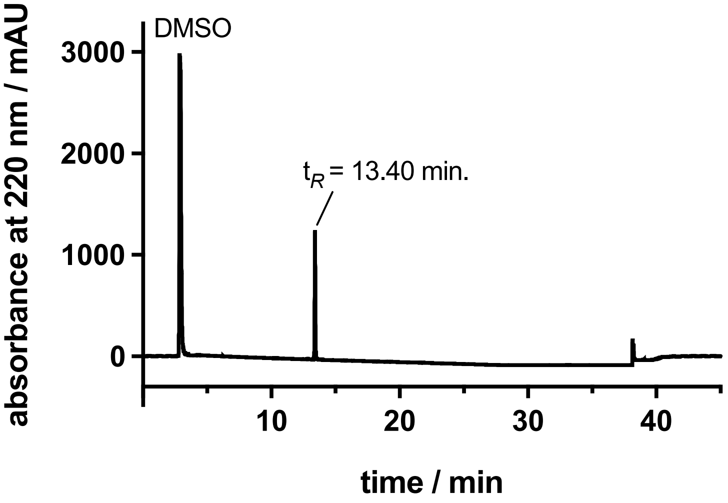

Figure S148. RP-HPLC analysis (purity control) of compound **26** (98 %, 220 nm).

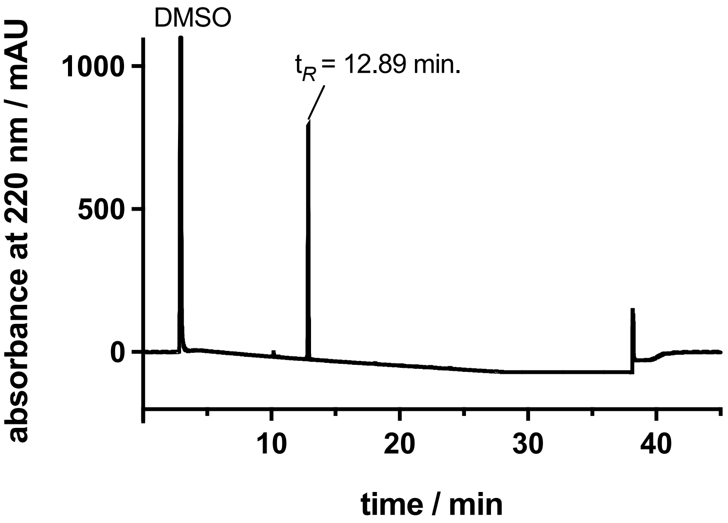

Figure S149. RP-HPLC analysis (purity control) of compound **27** (98 %, 220 nm).

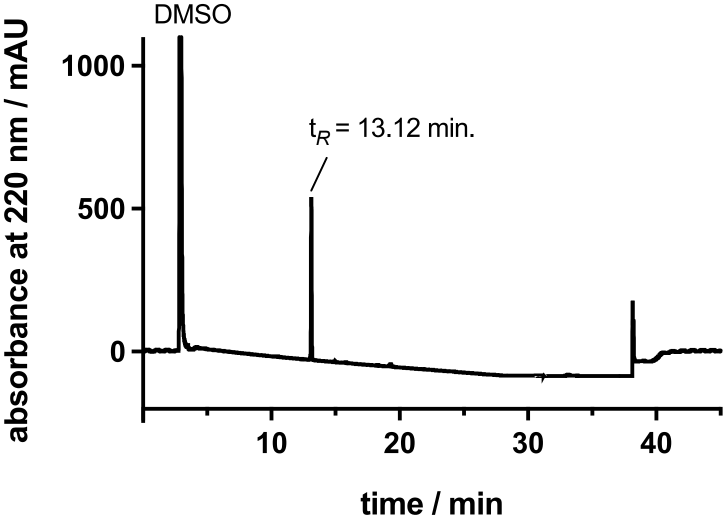

Figure S150. RP-HPLC analysis (purity control) of compound **28** (> 99 %, 220 nm).

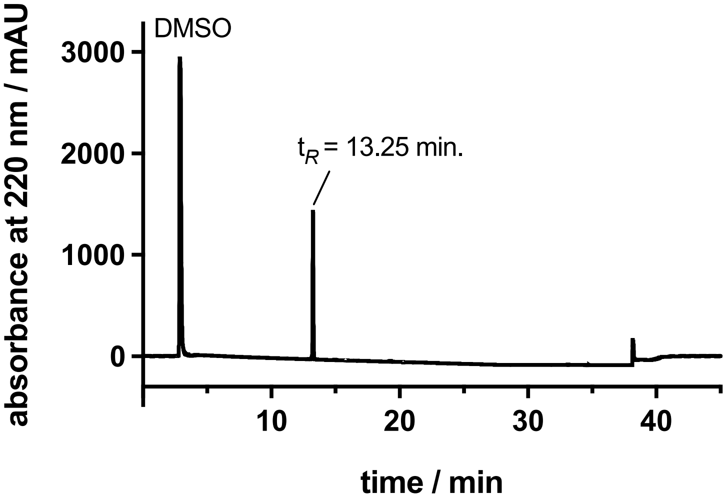

Figure S151. RP-HPLC analysis (purity control) of compound **29** (> 99 %, 220 nm).

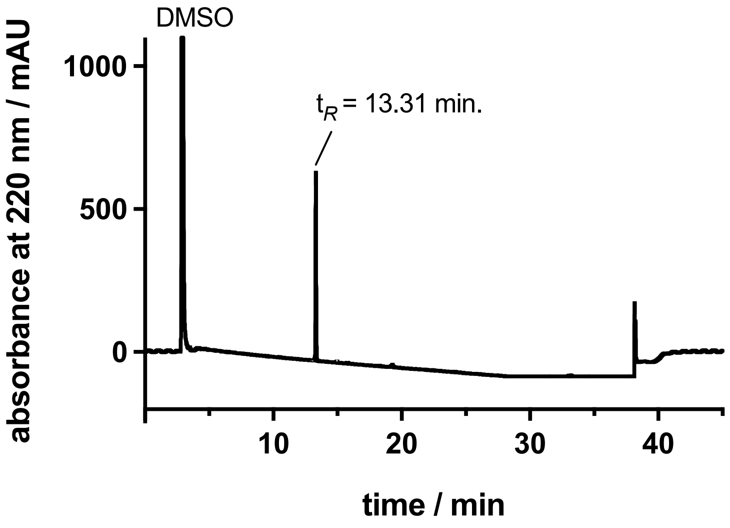

Figure S152. RP-HPLC analysis (purity control) of compound **30** (> 99 %, 220 nm).

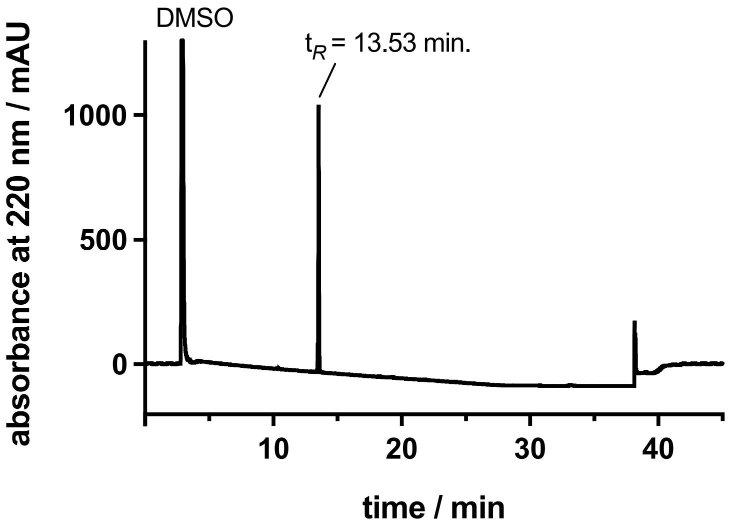

Figure S153. RP-HPLC analysis (purity control) of compound **31** (97 %, 220 nm).

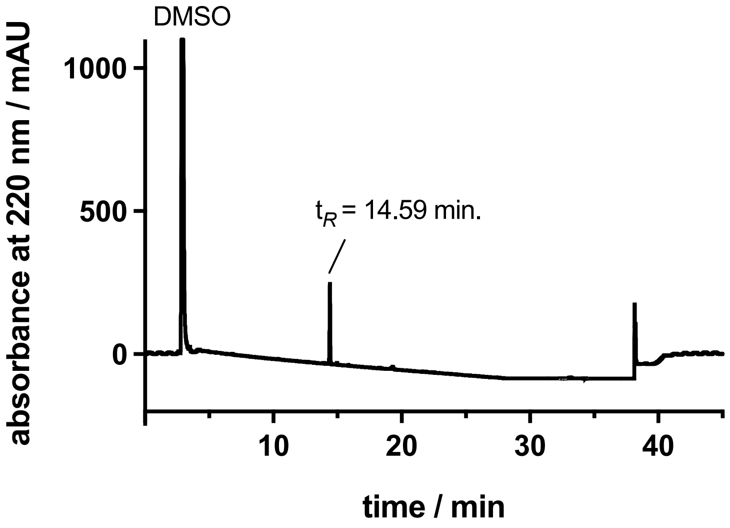

Figure S154. RP-HPLC analysis (purity control) of compound **32** (97 %, 220 nm).

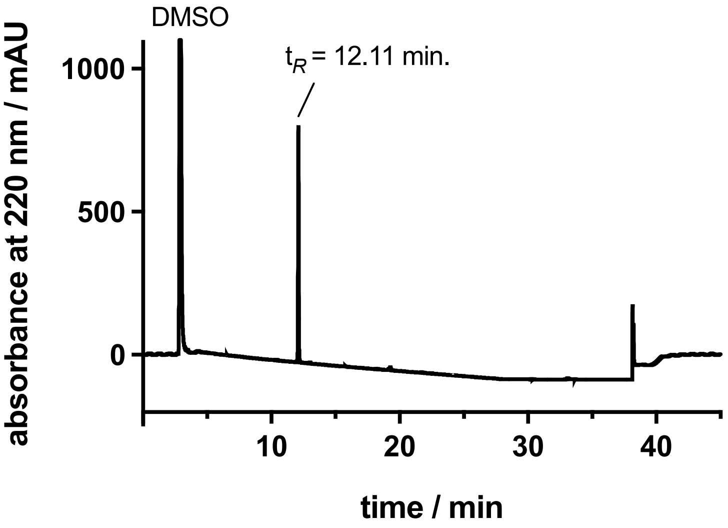

Figure S155. RP-HPLC analysis (purity control) of compound **33** (99 %, 220 nm).

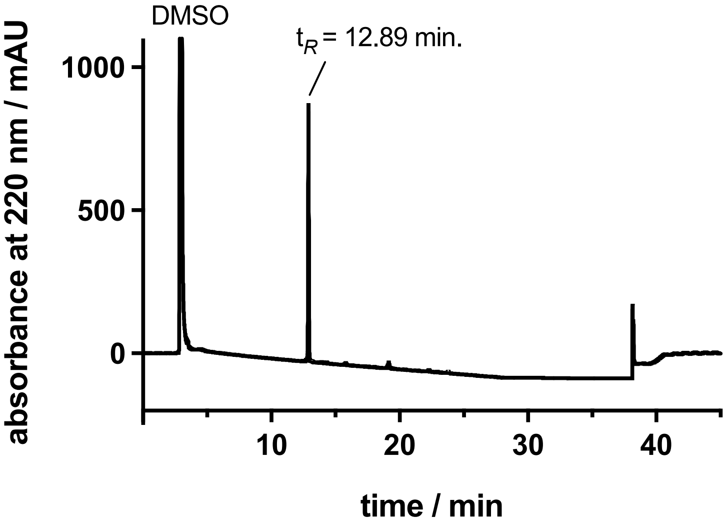

Figure S156. RP-HPLC analysis (purity control) of compound **34** (97 %, 220 nm).

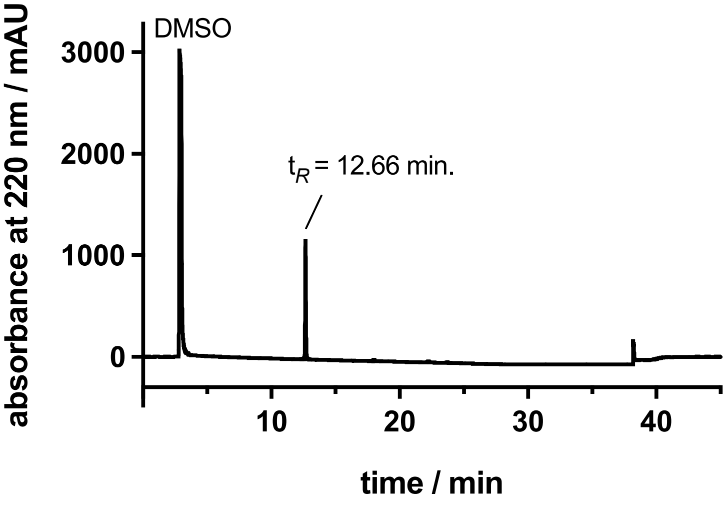

Figure S157. RP-HPLC analysis (purity control) of compound **35** (97 %, 220 nm).

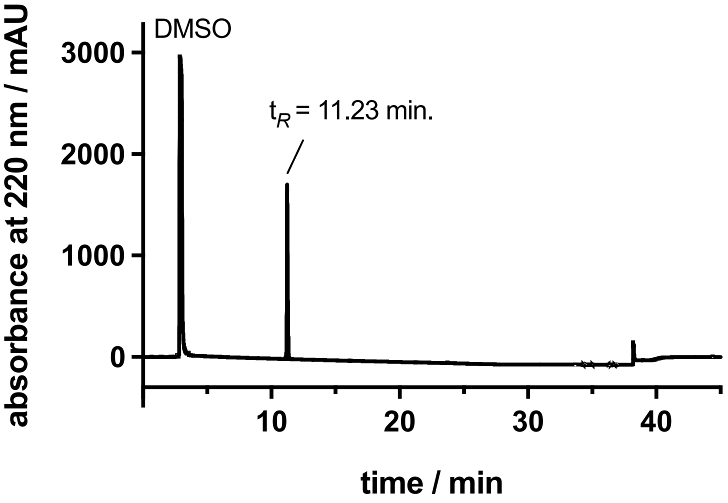

Figure S158. RP-HPLC analysis (purity control) of compound **36** (> 99 %, 220 nm).

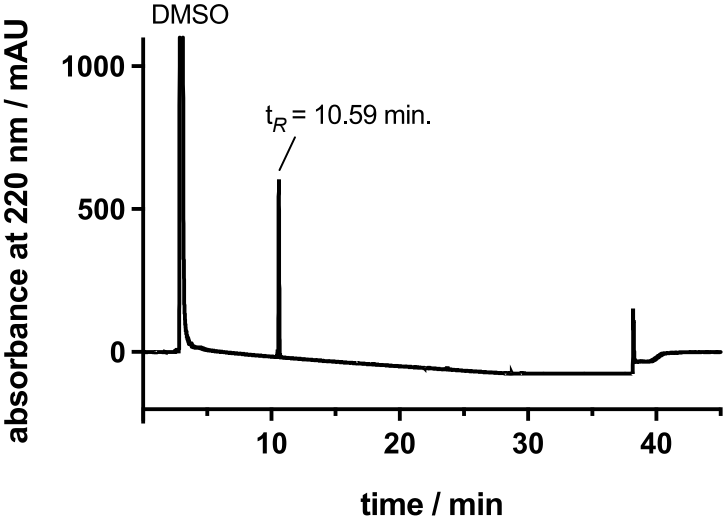

Figure S159. RP-HPLC analysis (purity control) of compound **37** (97 %, 220 nm).

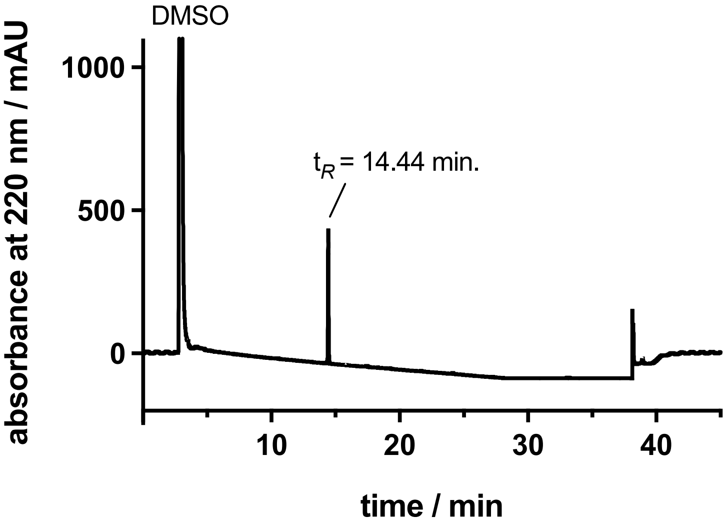

Figure S160. RP-HPLC analysis (purity control) of compound **52** (> 99 %, 220 nm).

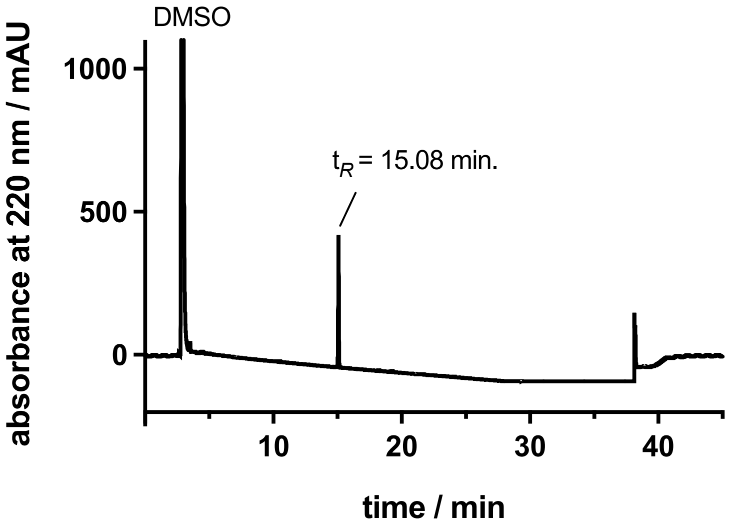

Figure S161. RP-HPLC analysis (purity control) of compound **53** (> 99 %, 220 nm).

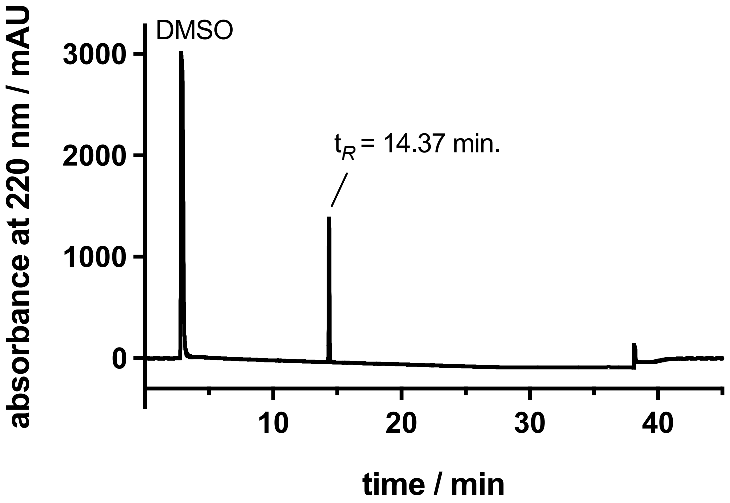

Figure S162. RP-HPLC analysis (purity control) of compound **54** (> 99 %, 220 nm).

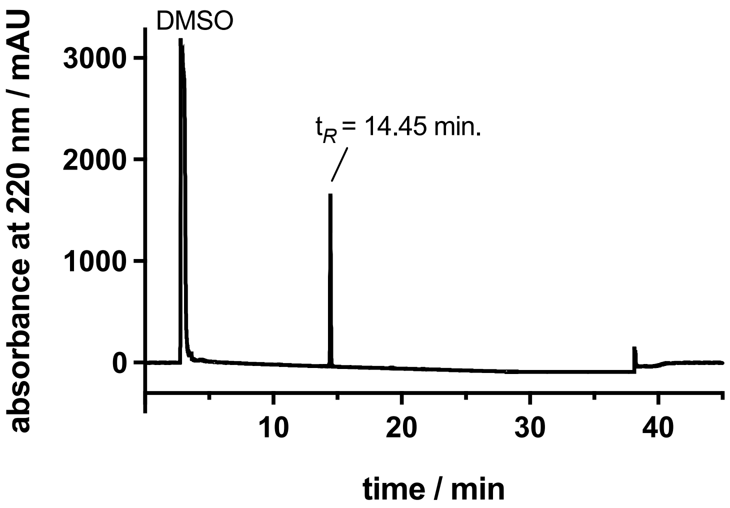

Figure S163. RP-HPLC analysis (purity control) of compound **55** (> 99 %, 220 nm).

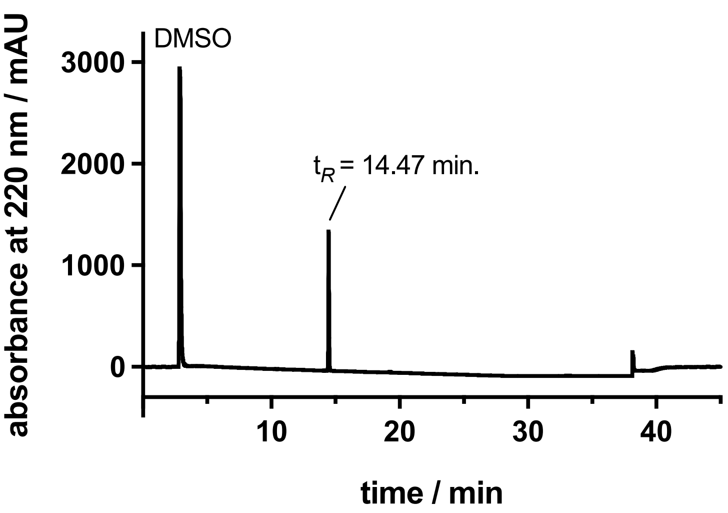

Figure S164. RP-HPLC analysis (purity control) of compound **56** (> 99 %, 220 nm).

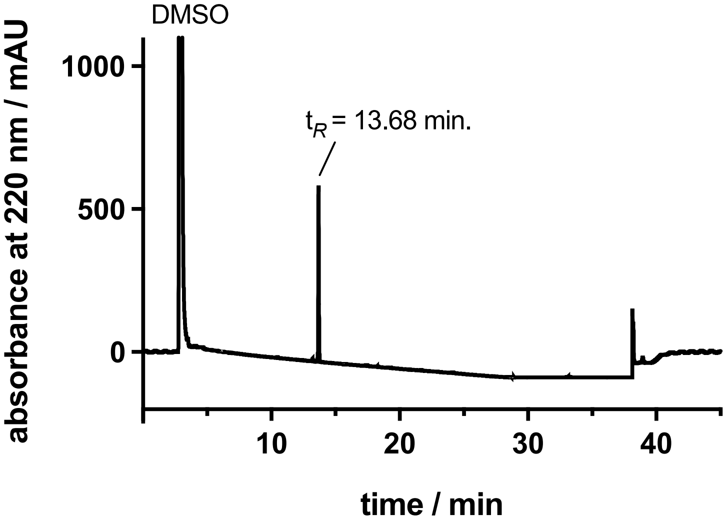

Figure S165. RP-HPLC analysis (purity control) of compound **57** (98 %, 220 nm).

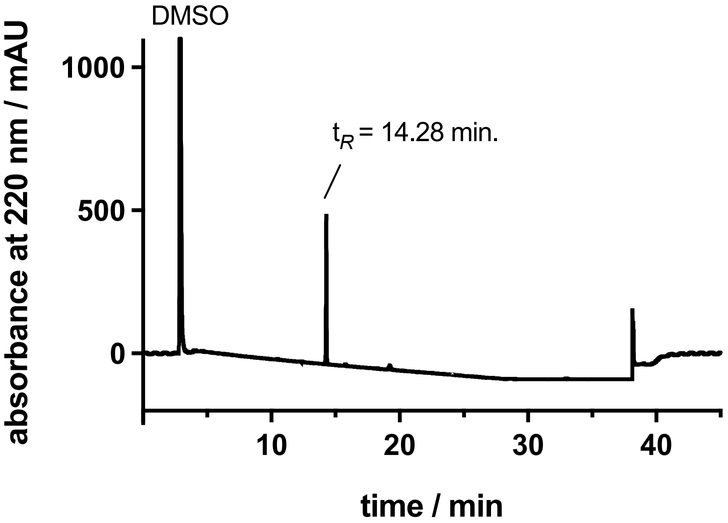

Figure S166. RP-HPLC analysis (purity control) of compound **58** (> 99 %, 220 nm).

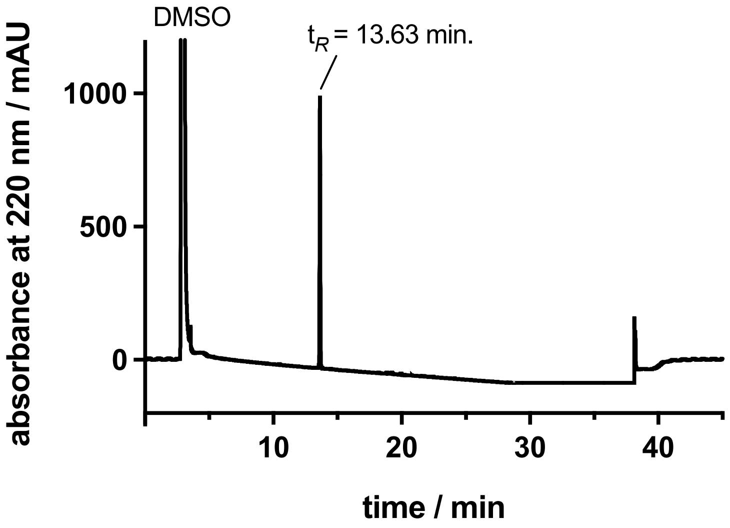

Figure S167. RP-HPLC analysis (purity control) of compound **59** (> 99 %, 220 nm).

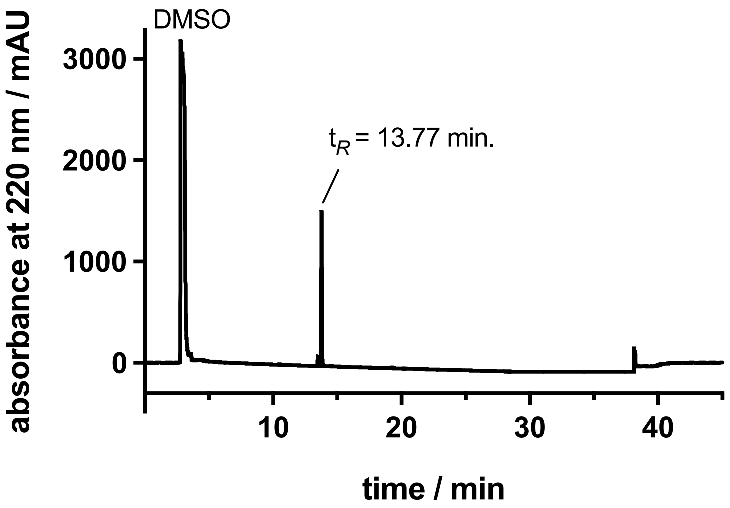

Figure S168. RP-HPLC analysis (purity control) of compound **60** (95 %, 220 nm).

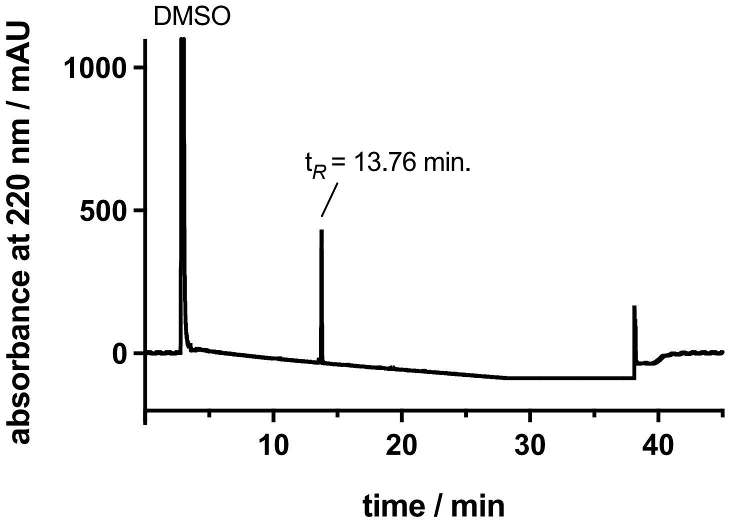

Figure S169. RP-HPLC analysis (purity control) of compound **61** (97 %, 220 nm).

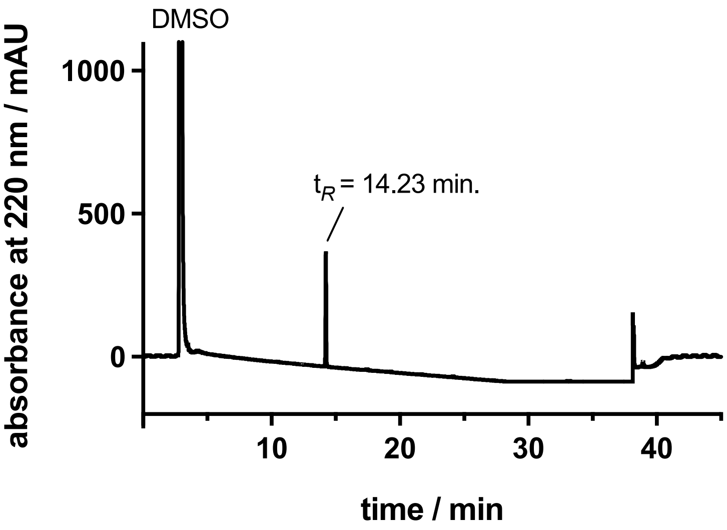

Figure S170. RP-HPLC analysis (purity control) of compound **62** (> 99 %, 220 nm).

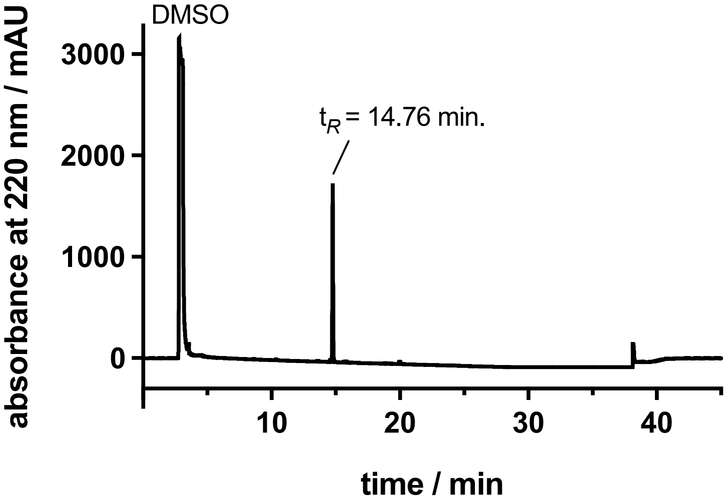

Figure S171. RP-HPLC analysis (purity control) of compound **63** (95 %, 220 nm).

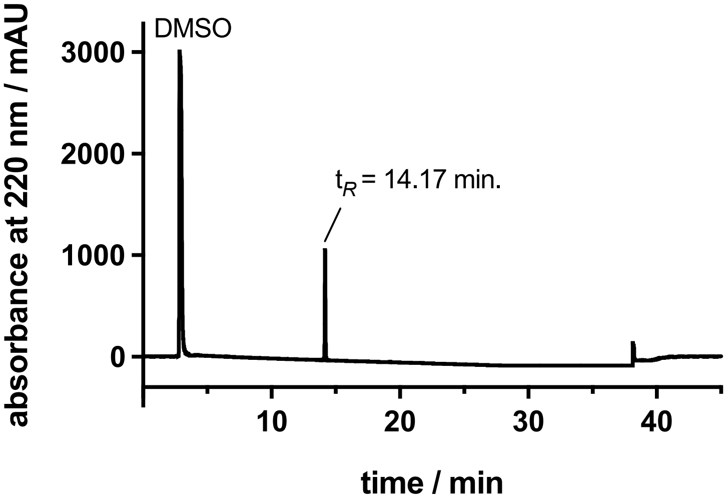

Figure S172. RP-HPLC analysis (purity control) of compound **64** (> 99 %, 220 nm).

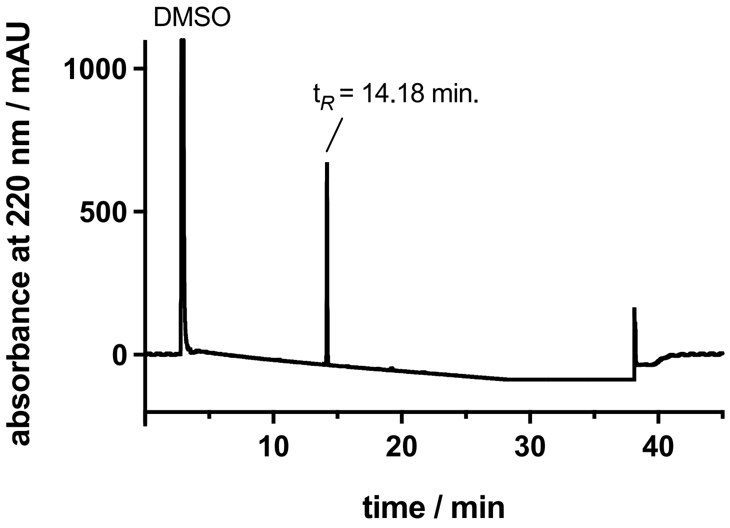

Figure S173. RP-HPLC analysis (purity control) of compound **65** (> 99 %, 220 nm).

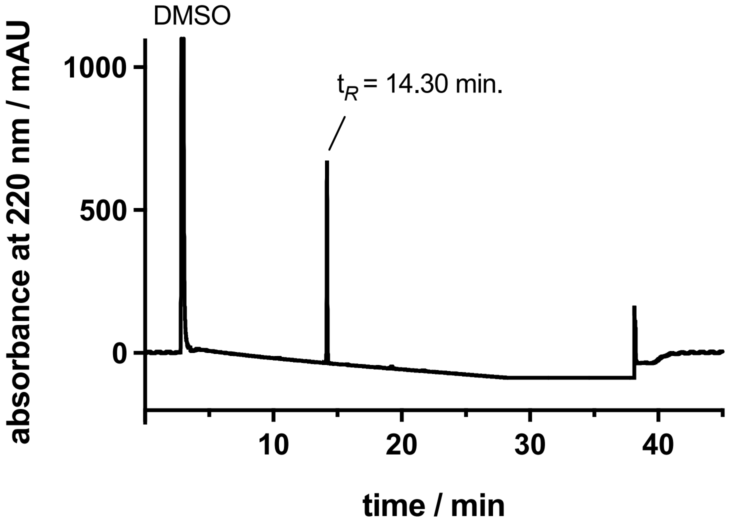

Figure S174. RP-HPLC analysis (purity control) of compound **66** (> 99 %, 220 nm).

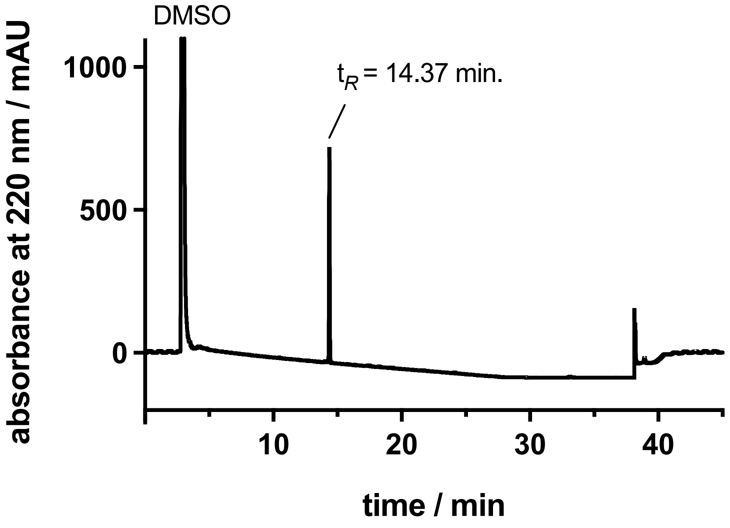

Figure S175. RP-HPLC analysis (purity control) of compound **67** (> 99 %, 220 nm).

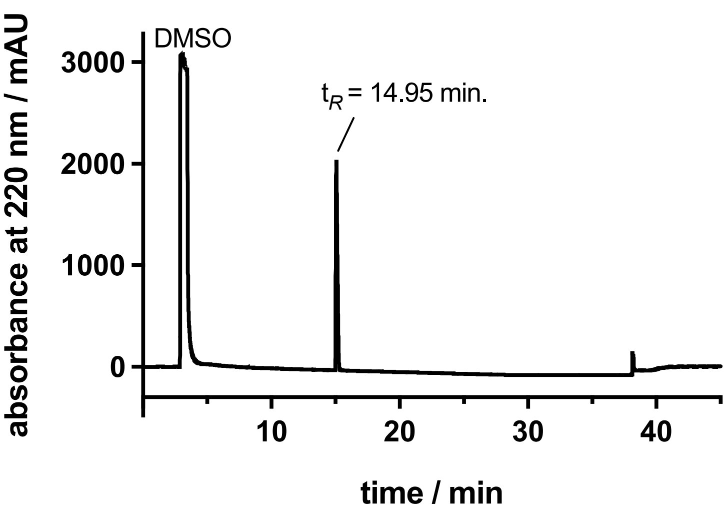

Figure S176. RP-HPLC analysis (purity control) of compound **68** (> 99 %, 220 nm).

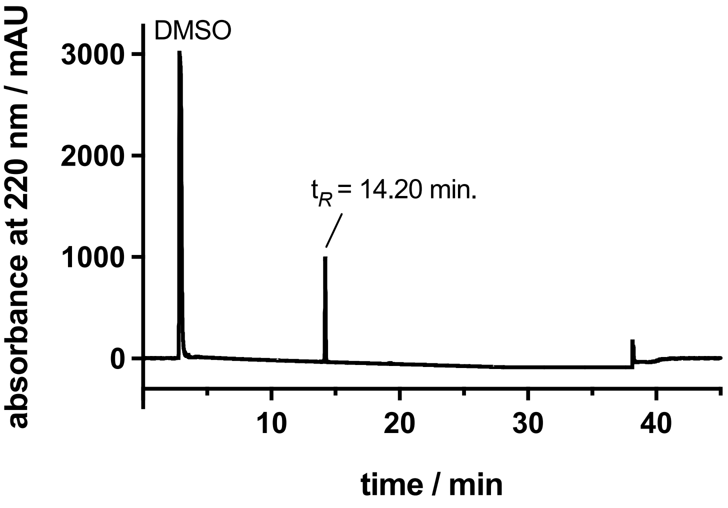

Figure S177. RP-HPLC analysis (purity control) of compound **69** (> 99 %, 220 nm).

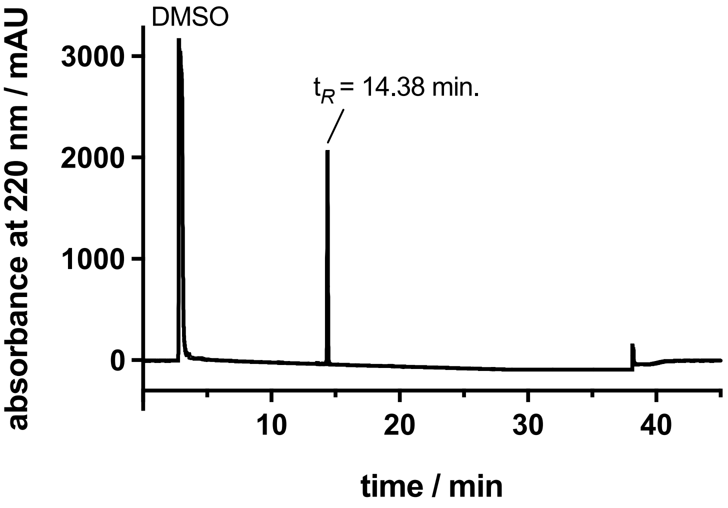

Figure S178. RP-HPLC analysis (purity control) of compound **70** (> 99 %, 220 nm).

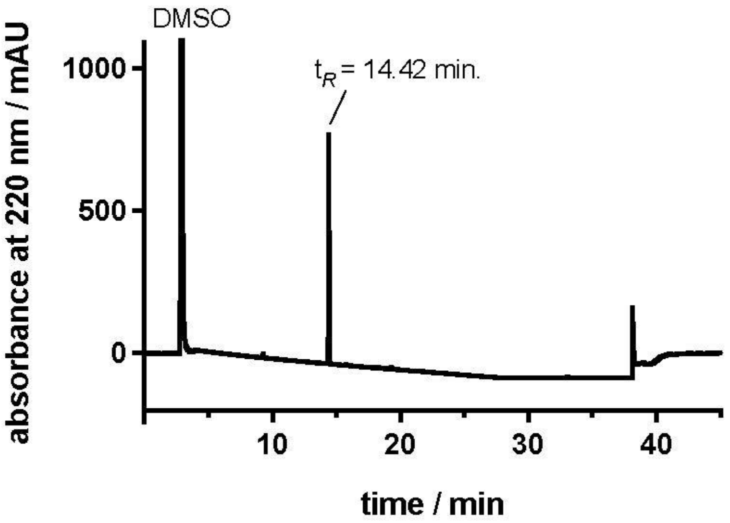

Figure S179. RP-HPLC analysis (purity control) of compound **71** (> 99 %, 220 nm).

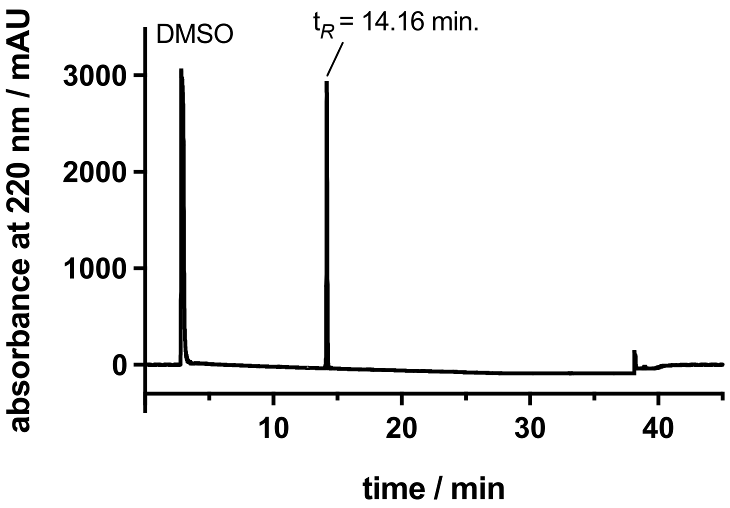

Figure S180. RP-HPLC analysis (purity control) of compound **72** (> 99 %, 220 nm).

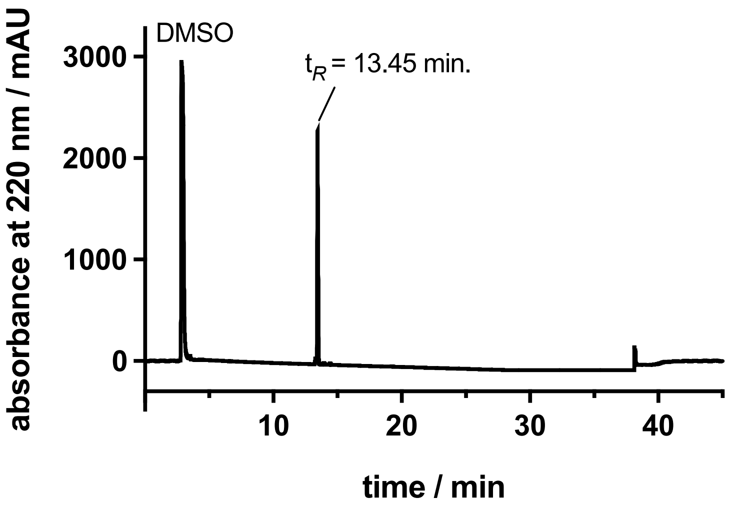

Figure S181. RP-HPLC analysis (purity control) of compound **73** (96 %, 220 nm).

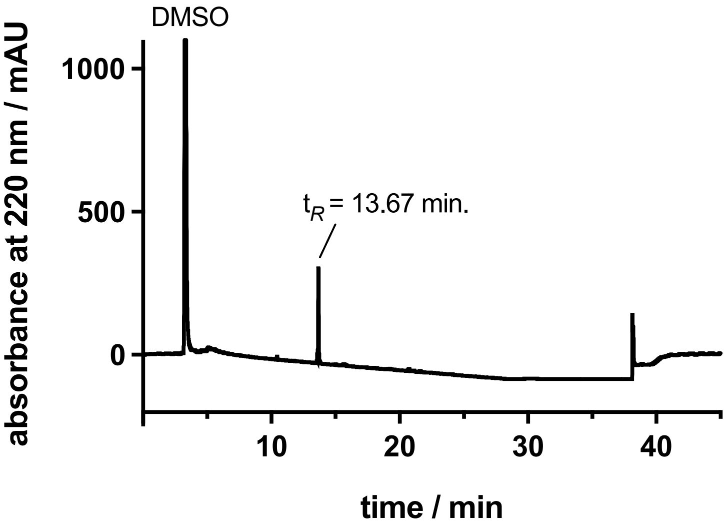

Figure S1852. RP-HPLC analysis (purity control) of compound **74** (95 %, 220 nm).

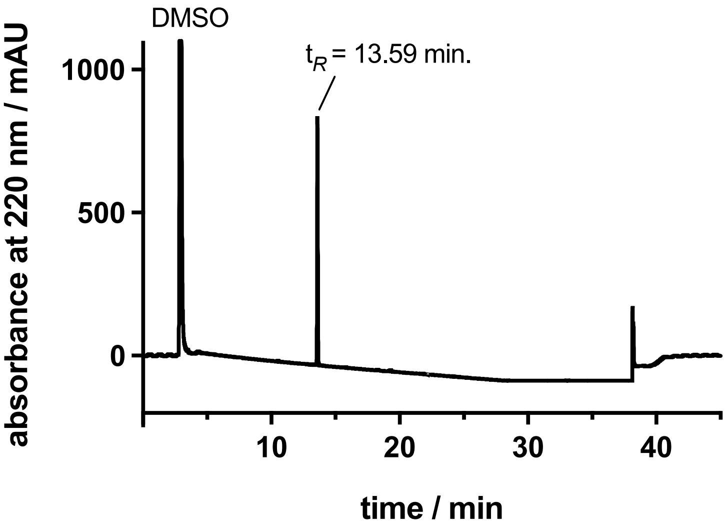

Figure S183. RP-HPLC analysis (purity control) of compound **75** (> 99 %, 220 nm).

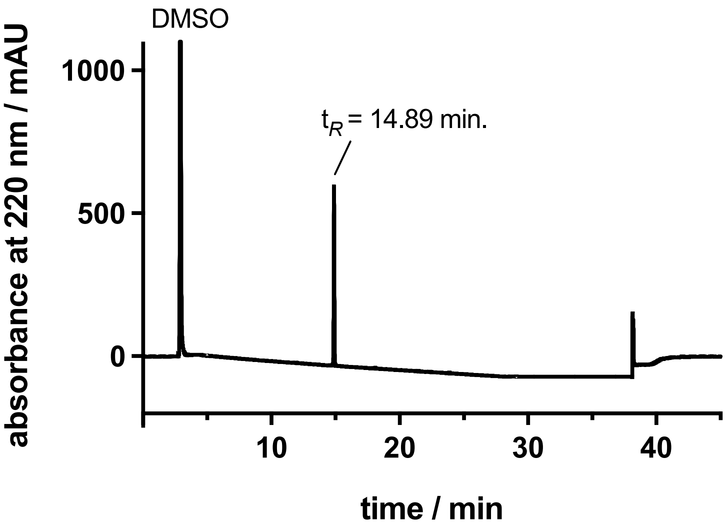

Figure S184. RP-HPLC analysis (purity control) of compound **76** (99 %, 220 nm).

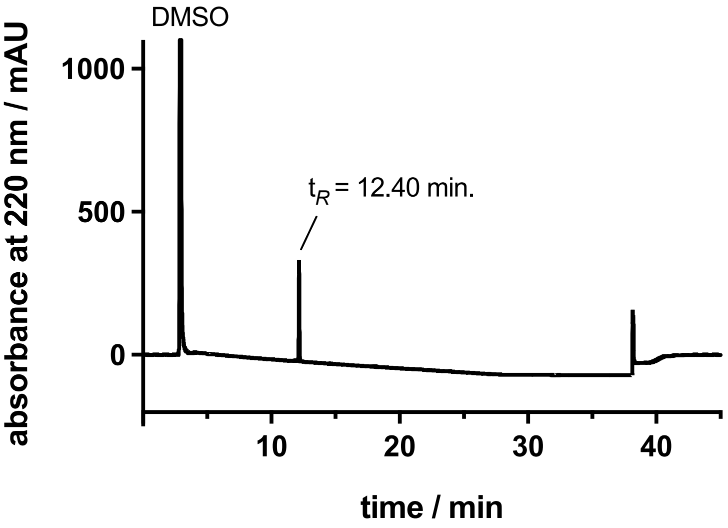

Figure S185. RP-HPLC analysis (purity control) of compound **77** (97 %, 220 nm).

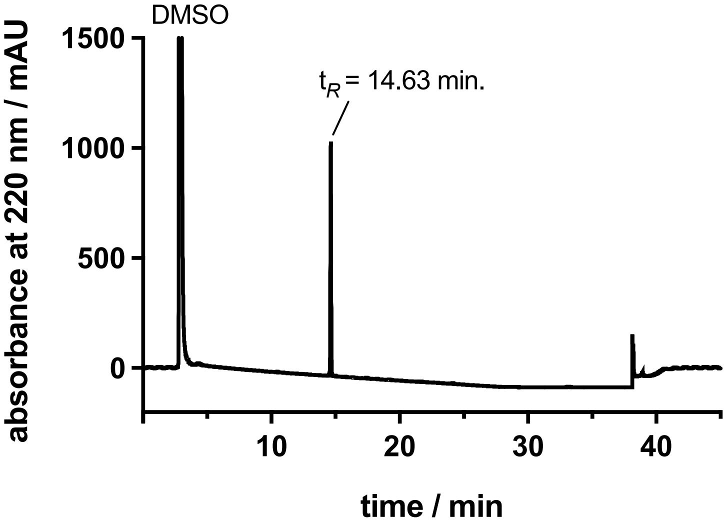

Figure S186. RP-HPLC analysis (purity control) of compound **80** (> 99 %, 220 nm).

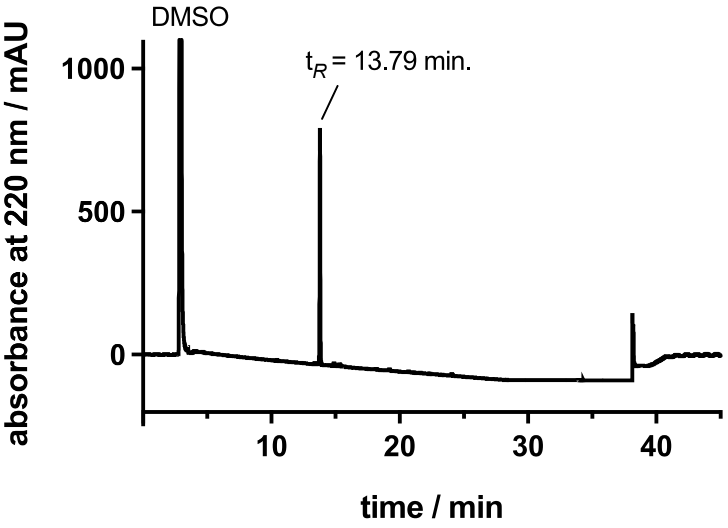

Figure S187. RP-HPLC analysis (purity control) of compound **81** (96 %, 220 nm).

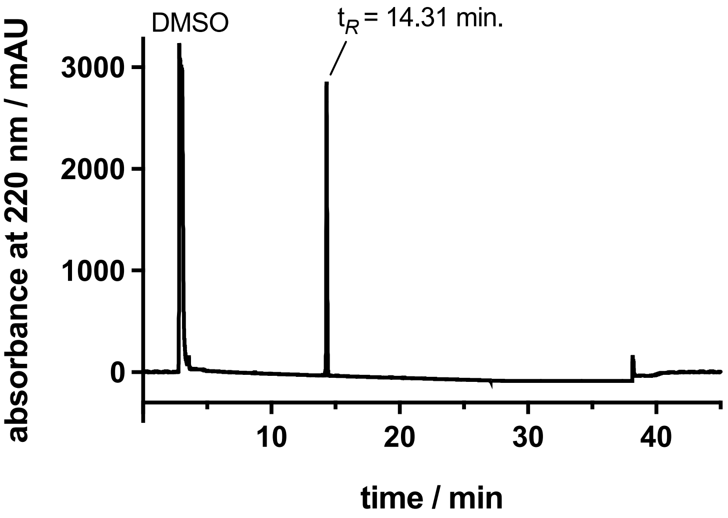

Figure S188. RP-HPLC analysis (purity control) of compound **82** (> 99 %, 220 nm).

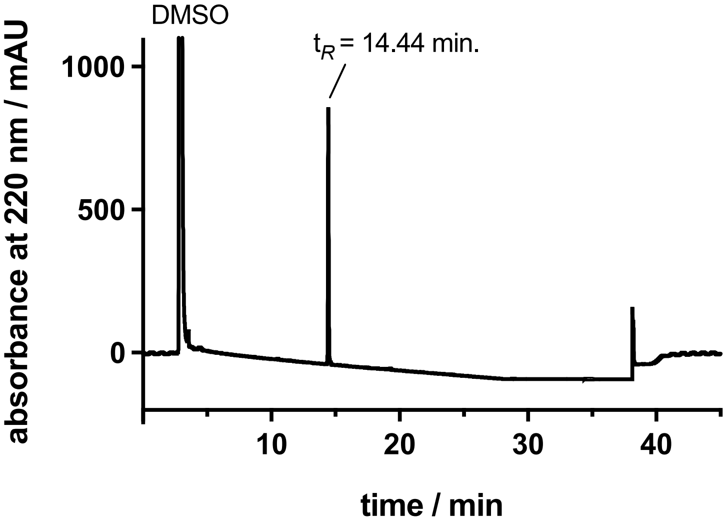

Figure S189. RP-HPLC analysis (purity control) of compound **83** (98 %, 220 nm).

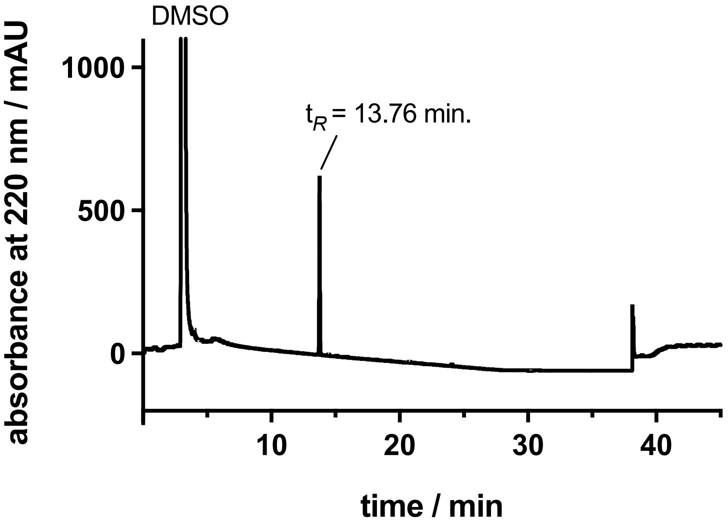

Figure S190. RP-HPLC analysis (purity control) of compound **84** (99 %, 220 nm).

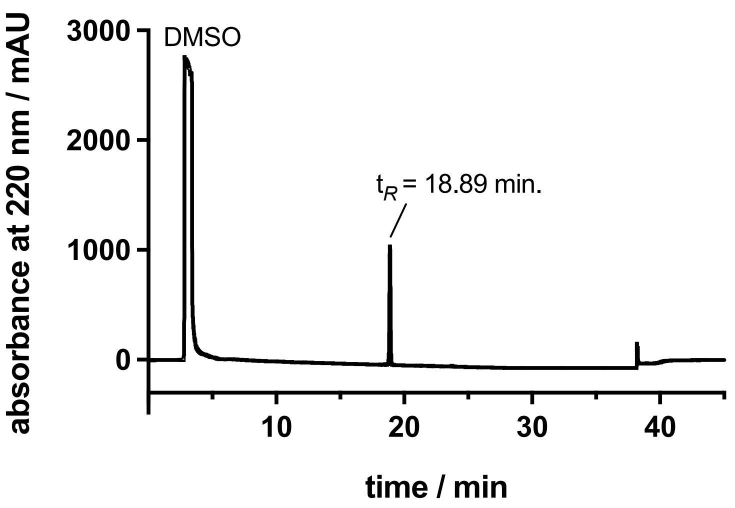

Figure S191. RP-HPLC analysis (purity control) of compound **93** (98 %, 220 nm).

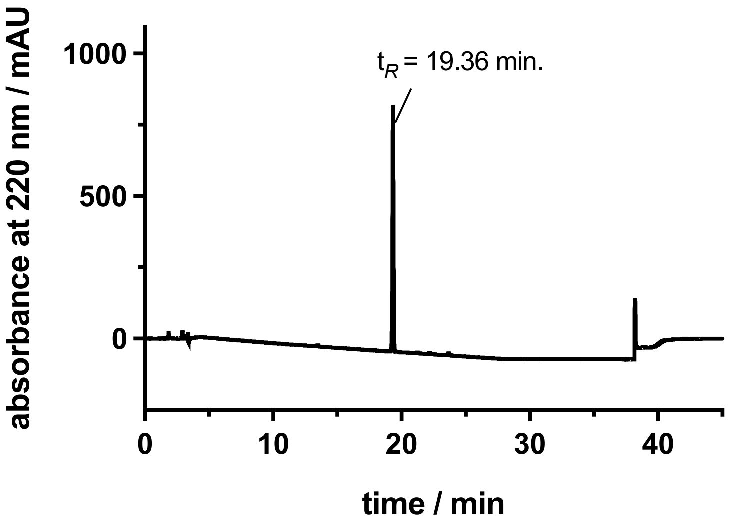

Figure S192. RP-HPLC analysis (purity control) of compound **94** (97 %, 220 nm).

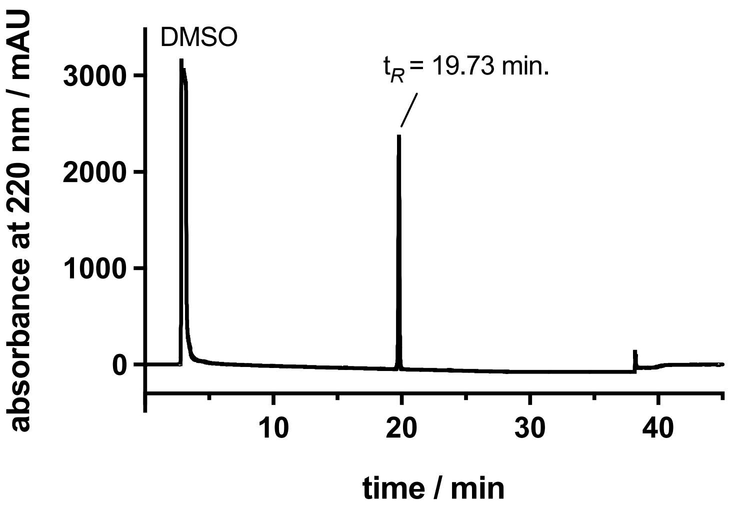

Figure S193. RP-HPLC analysis (purity control) of compound **95** (99 %, 220 nm).

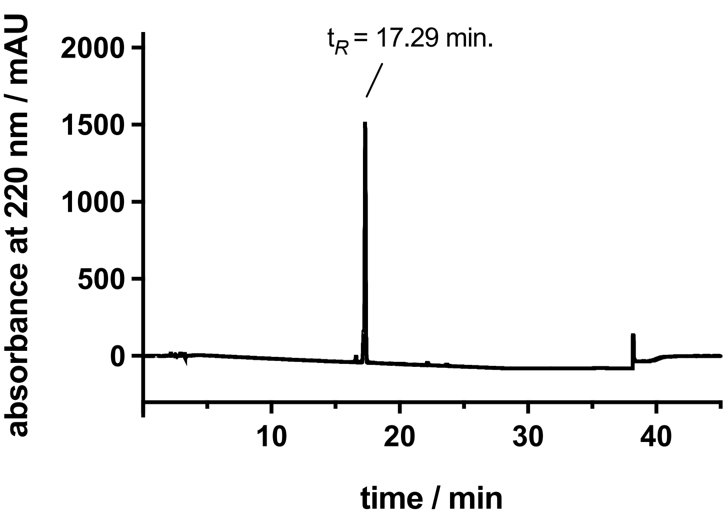

Figure S194. RP-HPLC analysis (purity control) of compound **96** (95 %, 220 nm).

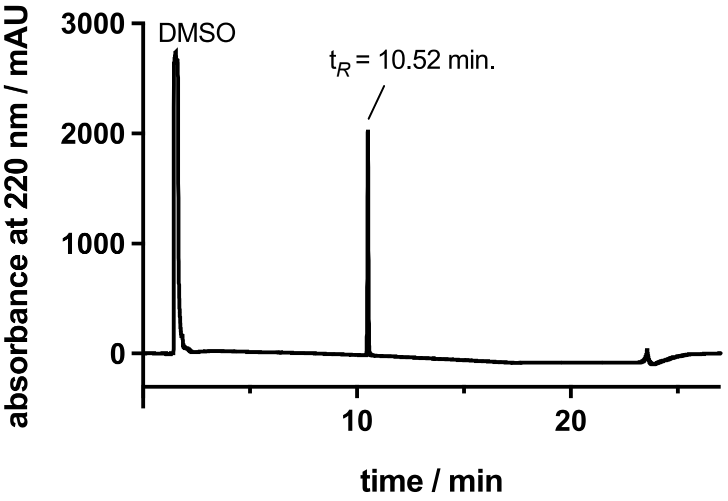

Figure S195. RP-HPLC analysis (purity control) of compound **97** (99 %, 220 nm).

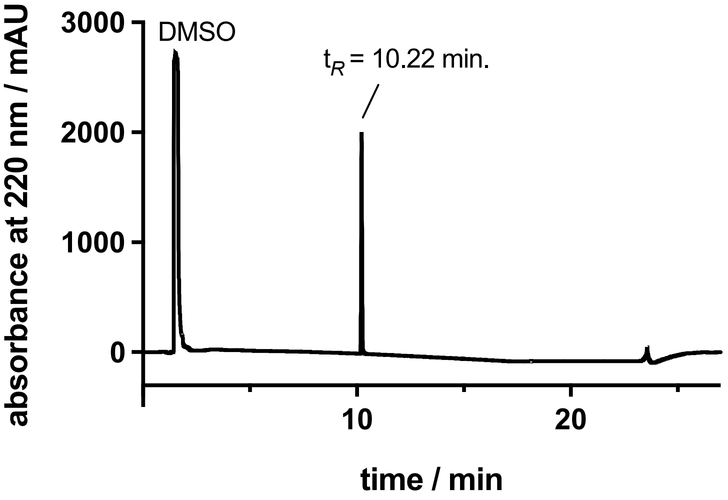

Figure S196. RP-HPLC analysis (purity control) of compound **98** (99 %, 220 nm).

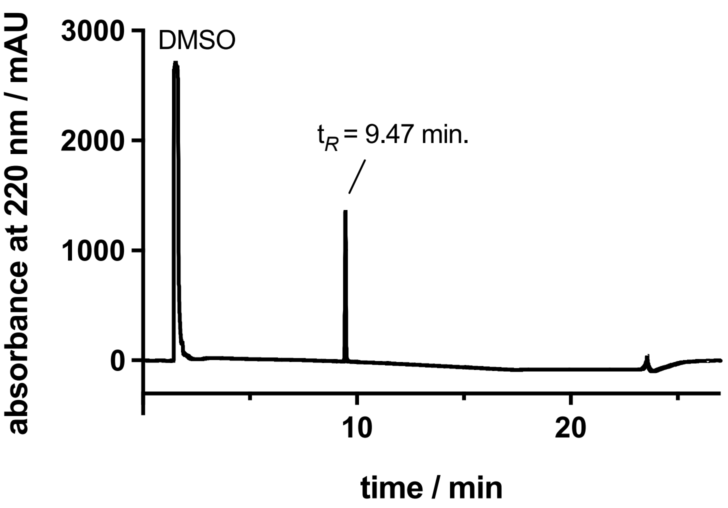

Figure S197. RP-HPLC analysis (purity control) of compound **99** (99 %, 220 nm).

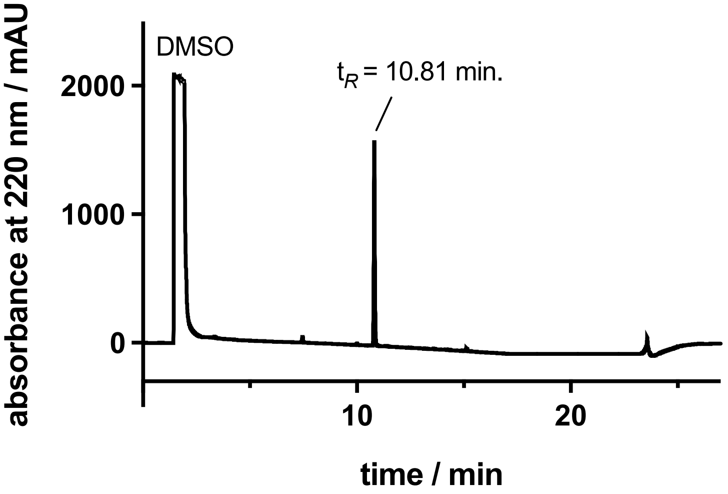

Figure S198. RP-HPLC analysis (purity control) of compound **100** (96 %, 220 nm).

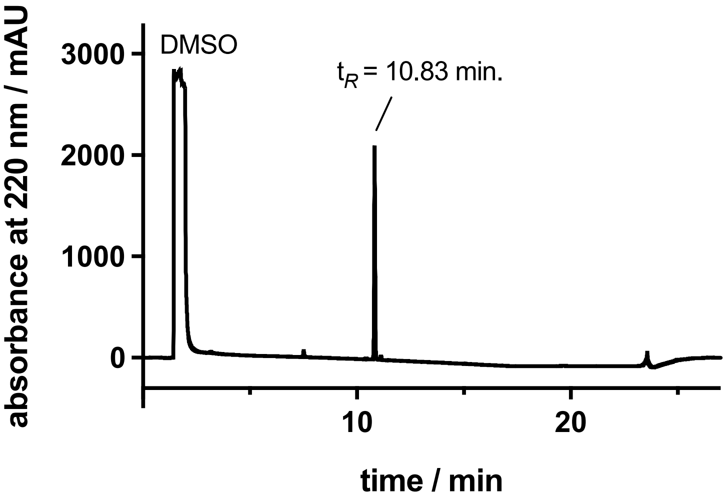

Figure S199. RP-HPLC analysis (purity control) of compound **101** (95 %, 220 nm).

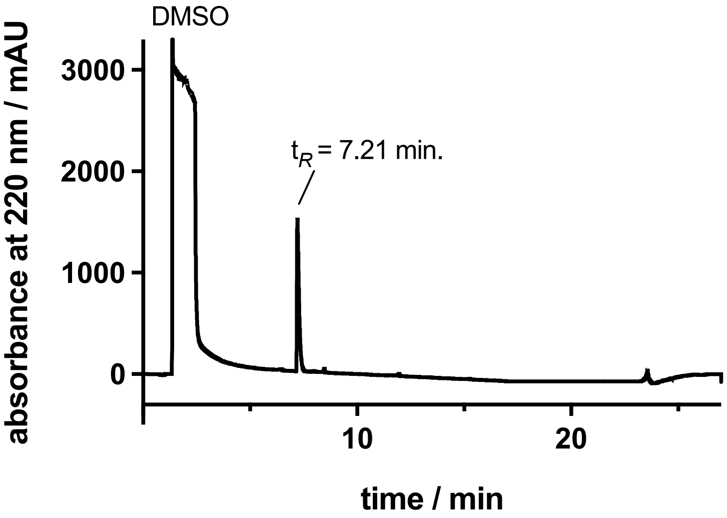

Figure S200. RP-HPLC analysis (purity control) of compound **102** (97 %, 220 nm).

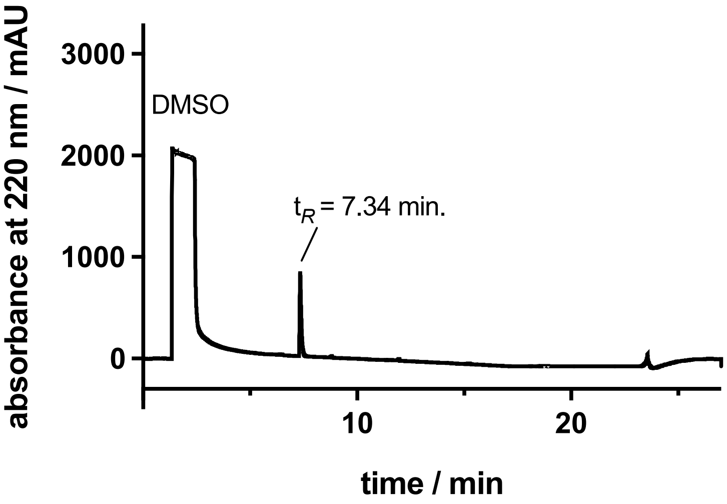

Figure S201. RP-HPLC analysis (purity control) of compound **103** (97 %, 220 nm).

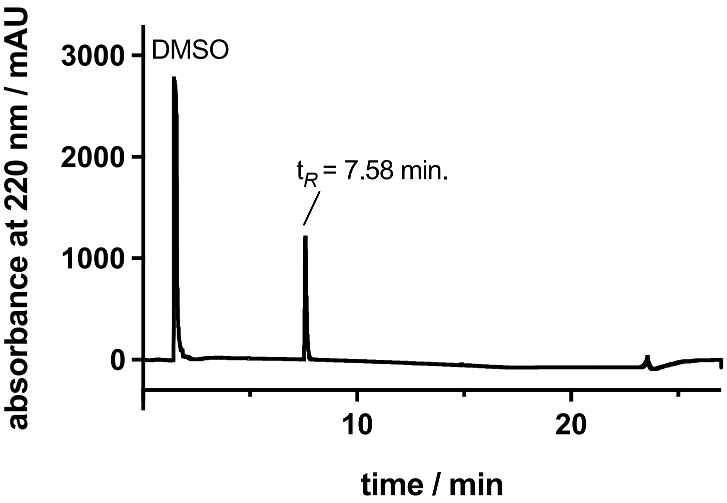

Figure S202. RP-HPLC analysis (purity control) of compound **104** (99 %, 220 nm).

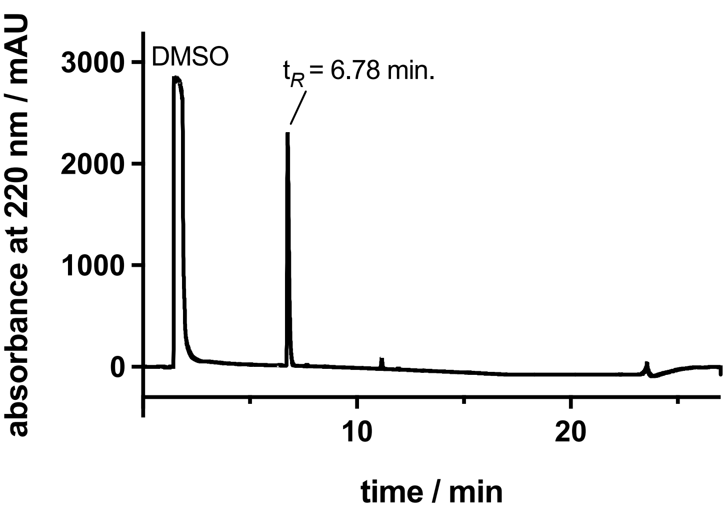

Figure S203. RP-HPLC analysis (purity control) of compound **105** (96 %, 220 nm).

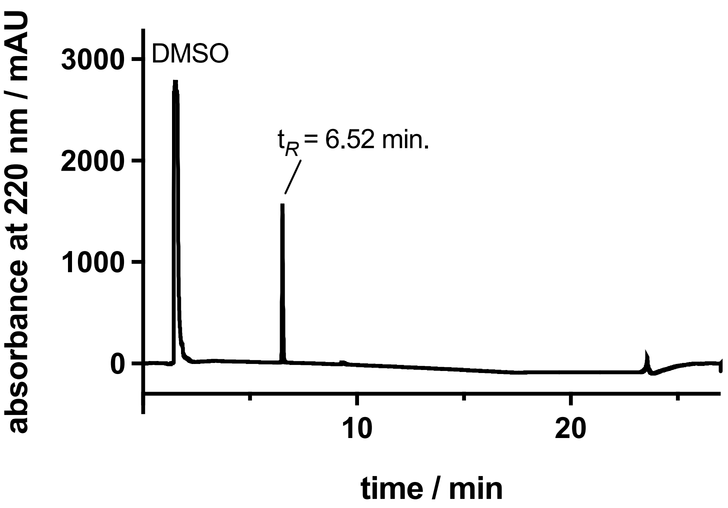

Figure S204. RP-HPLC analysis (purity control) of compound **106** (96 %, 220 nm).

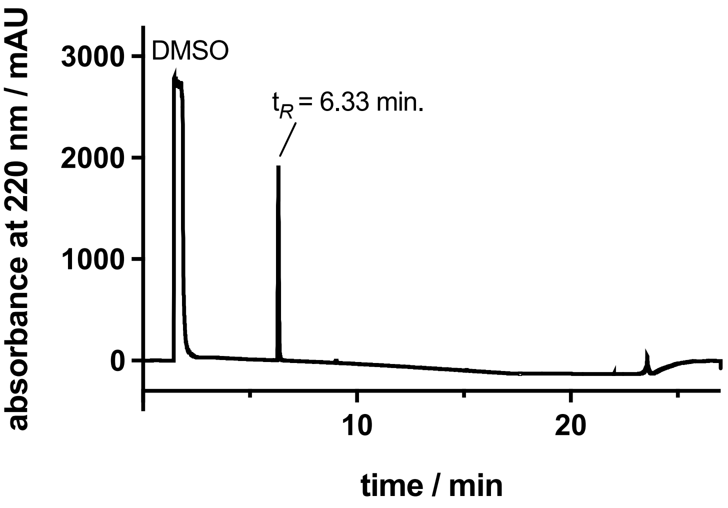

Figure S205. RP-HPLC analysis (purity control) of compound **107** (99 %, 220 nm).

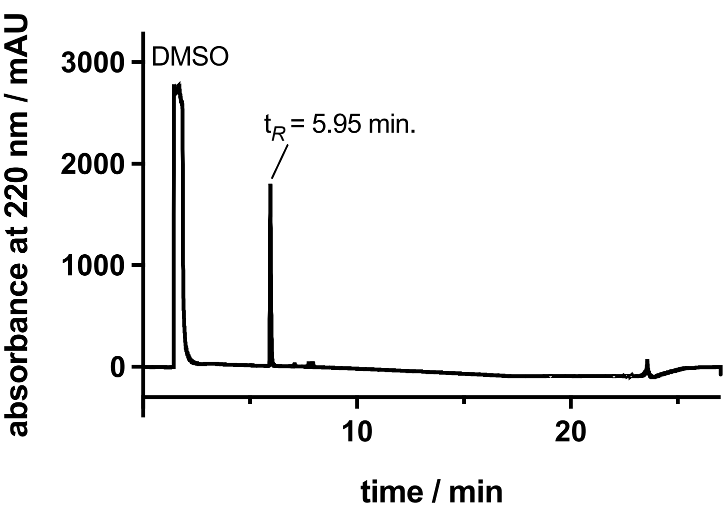

Figure S206. RP-HPLC analysis (purity control) of compound **108** (95 %, 220 nm).

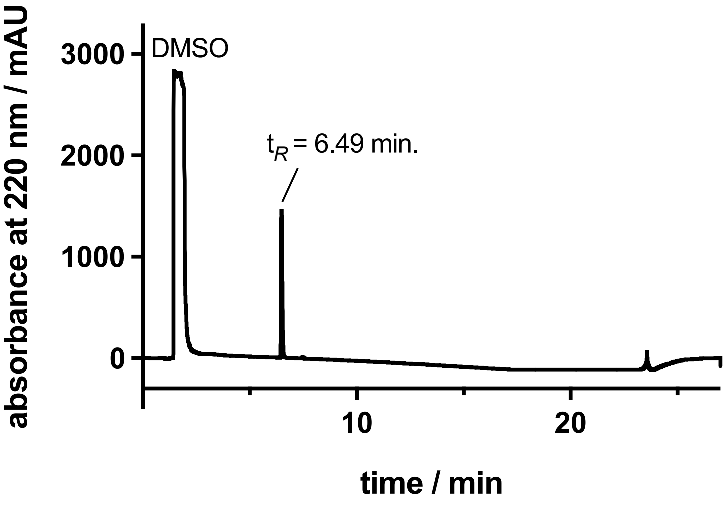

Figure S207. RP-HPLC analysis (purity control) of compound **109** (99 %, 220 nm).

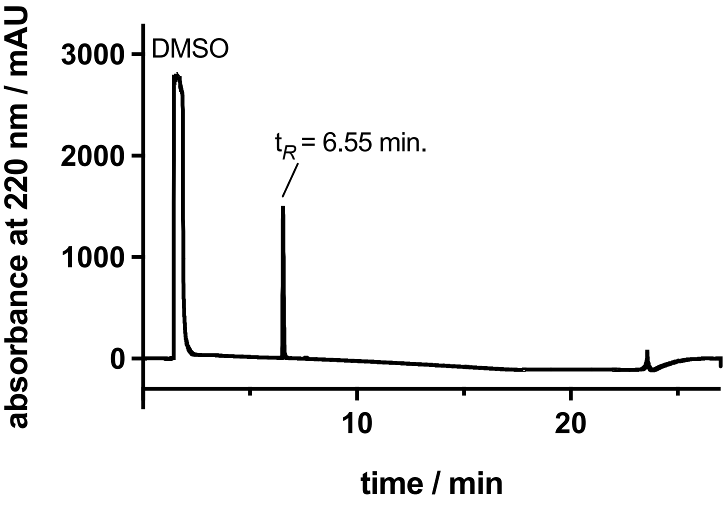

Figure S208. RP-HPLC analysis (purity control) of compound **110** (99 %, 220 nm).

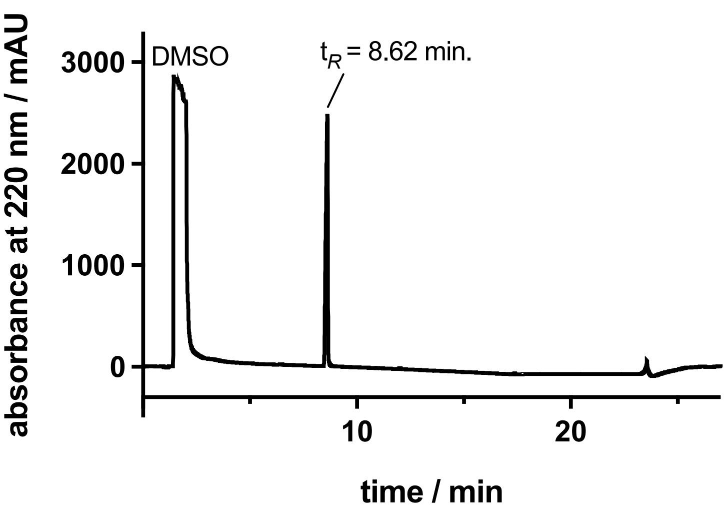

Figure S209. RP-HPLC analysis (purity control) of compound **115** (99 %, 220 nm).

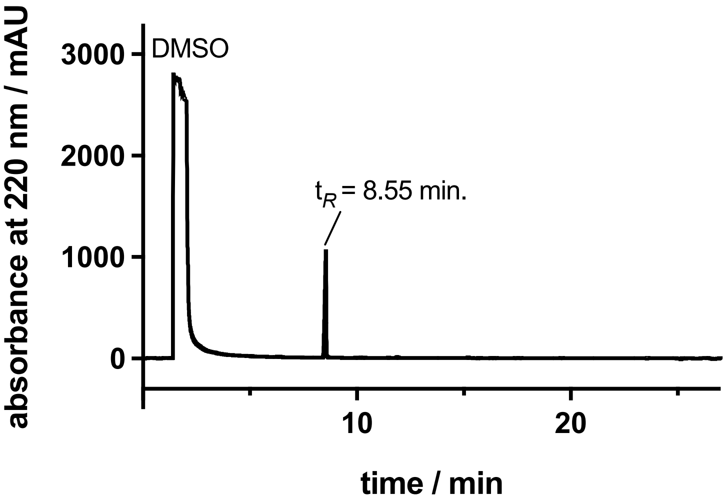

Figure S210. RP-HPLC analysis (purity control) of compound **116** (96 %, 220 nm).

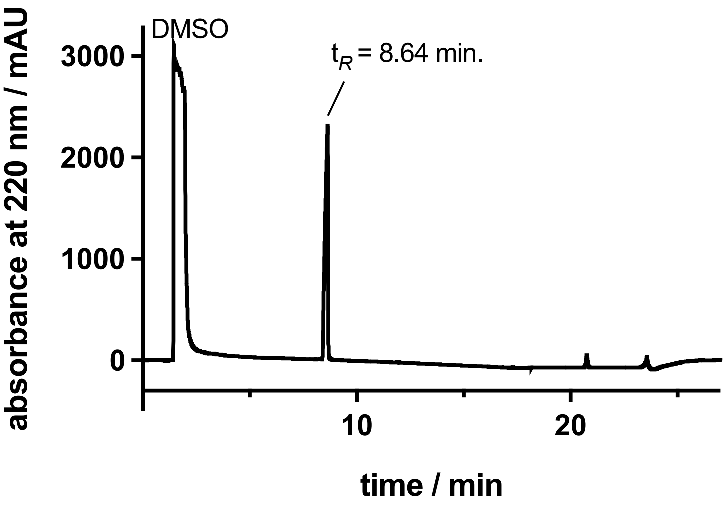

Figure S211. RP-HPLC analysis (purity control) of compound **117** (96 %, 220 nm).

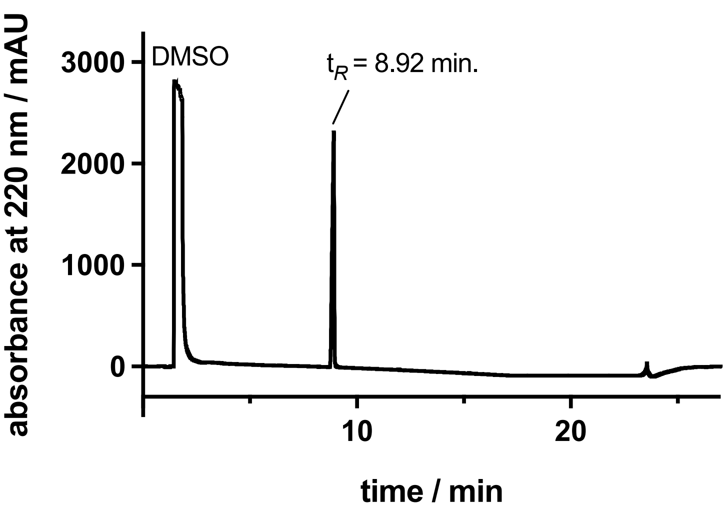

Figure S212. RP-HPLC analysis (purity control) of compound **118** (98 %, 220 nm).

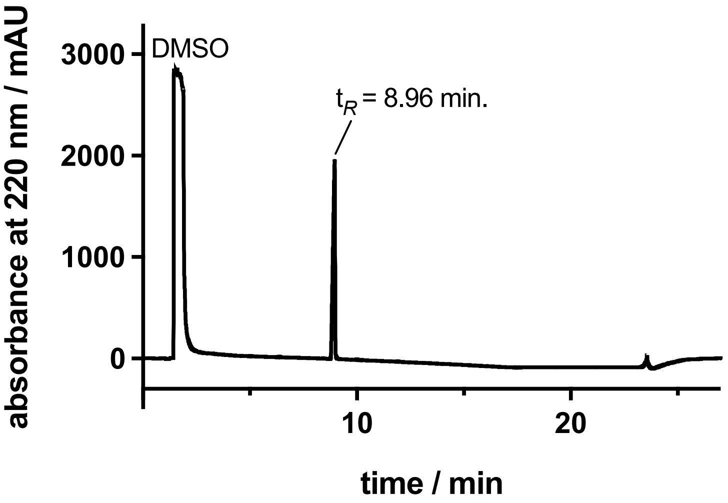

Figure S213. RP-HPLC analysis (purity control) of compound **119** (99 %, 220 nm).

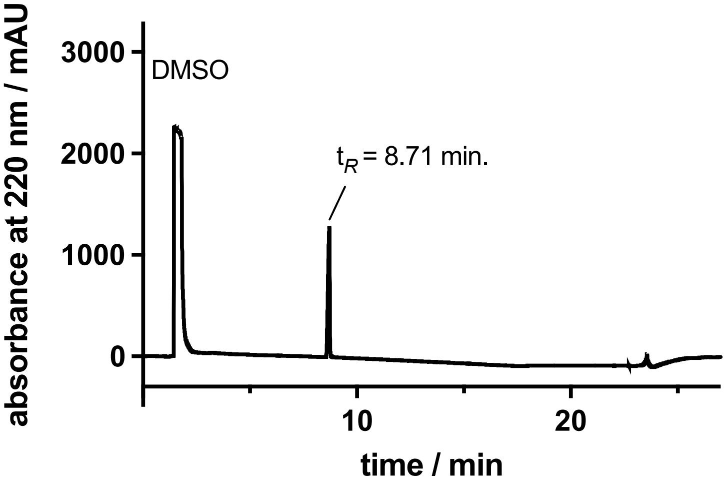

Figure S214. RP-HPLC analysis (purity control) of compound **120** (98 %, 220 nm).

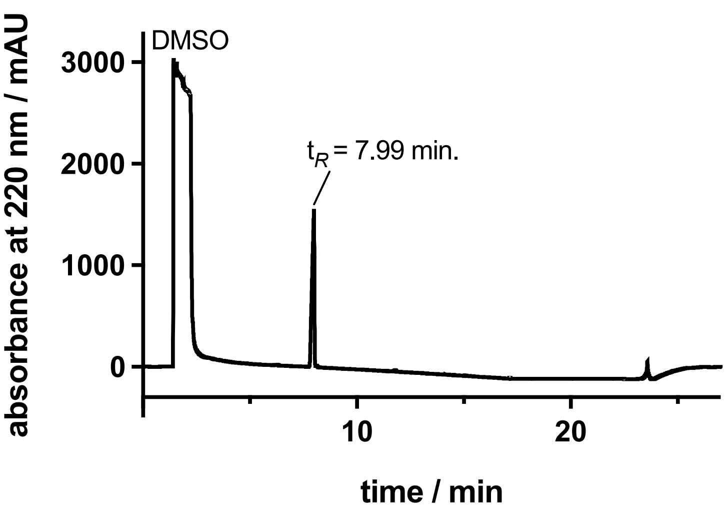

Figure S215. RP-HPLC analysis (purity control) of compound **121** (97 %, 220 nm).

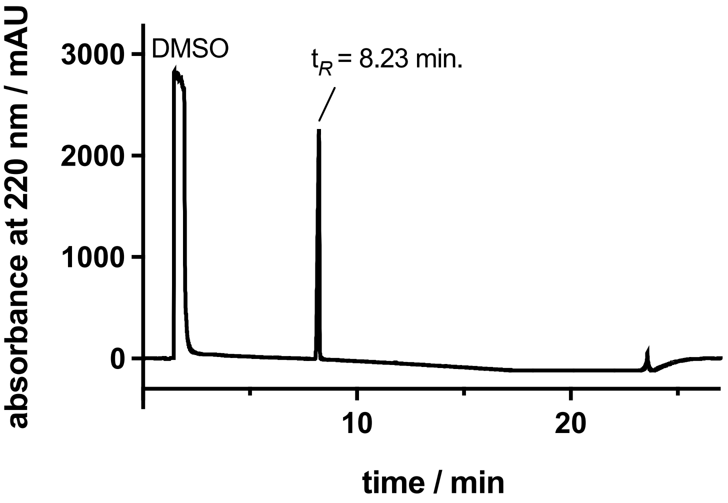

Figure S216. RP-HPLC analysis (purity control) of compound **122** (99 %, 220 nm).

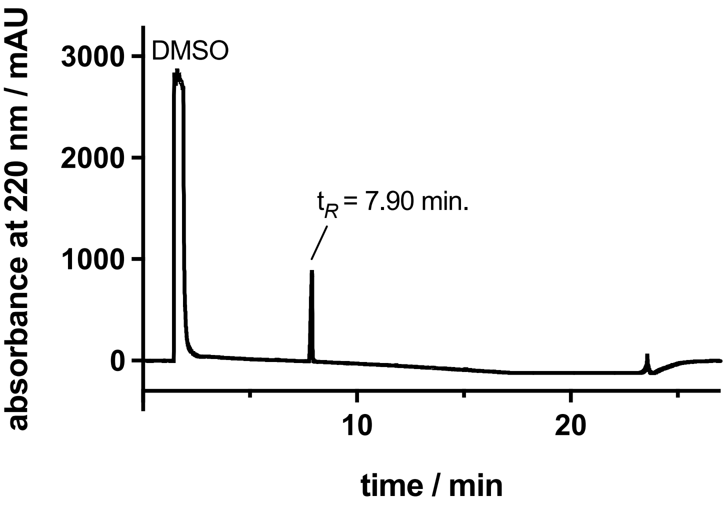

Figure S217. RP-HPLC analysis (purity control) of compound **123** (95 %, 220 nm).

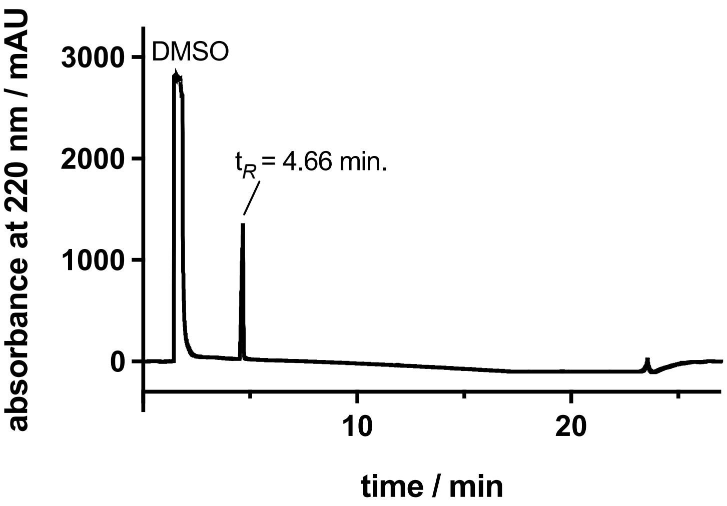

Figure S218. RP-HPLC analysis (purity control) of compound **124** (99 %, 220 nm).

Figure S219: Ligand-based virtual screening for advanced VH1 analogs.

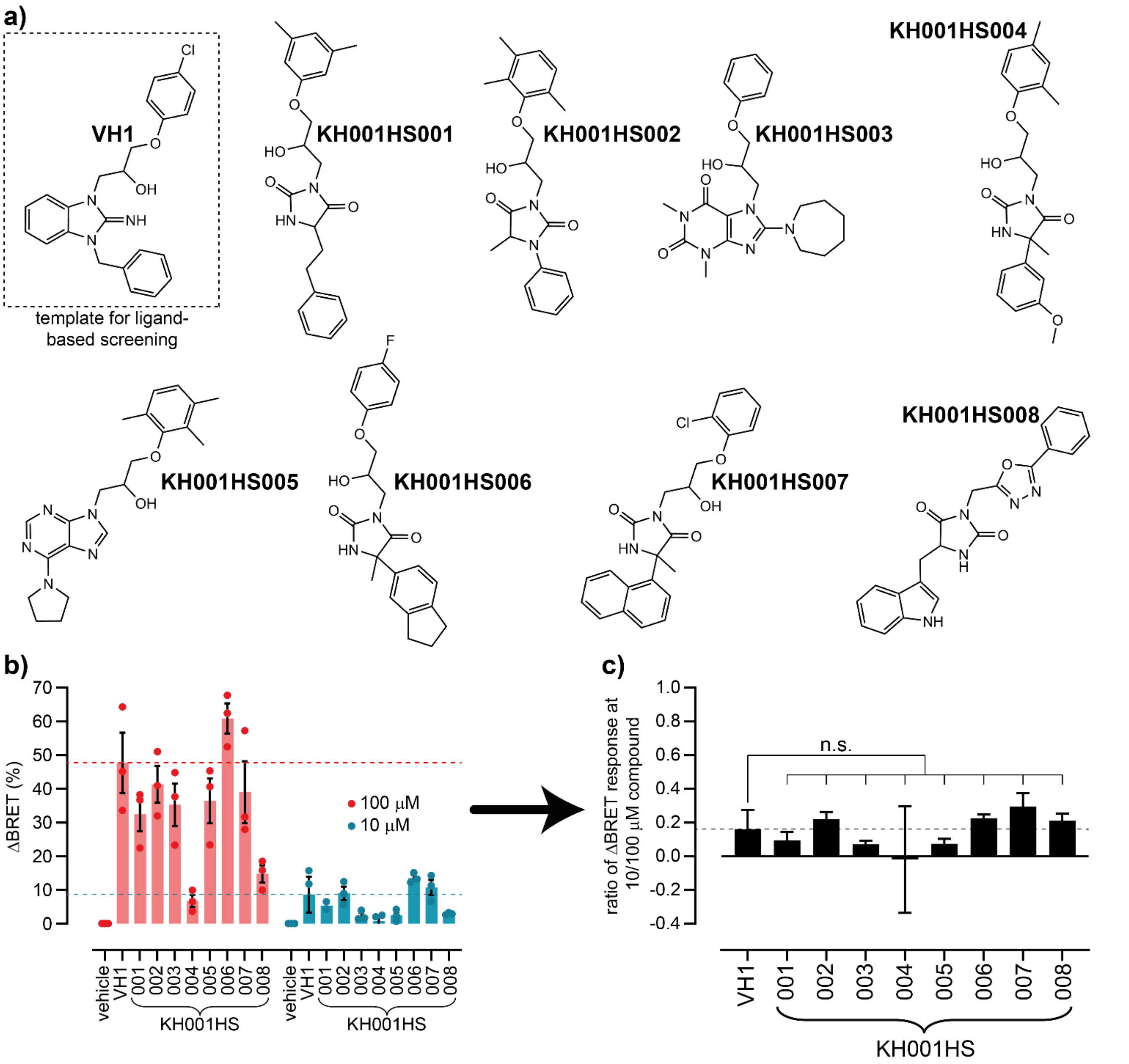

Ligand-based screening for GPR3 ligands. a) Chemical structures of the template molecule, VH1, used for the ligand-based screening of GPR3 ligands and eight purchased chemical analogs, KH001HS001 – KH001HS008. b) BRET responses measured in HEK293A cells stably expressing the GPR3-HaloTag/Nluc biosensor and treated with 100 μM or 10 μM of the indicated compounds. c) Response ratios (10 μM over 100 μM) obtained from the data shown in (b). Data show mean ± SEM of three independent experiments. Statistical difference to VH1 in (c) was calculated using One-Way ANOVA followed by Dunnett's multiple comparison.

Figure S220: Ligand-based virtual screening for advanced VH2 analogs.

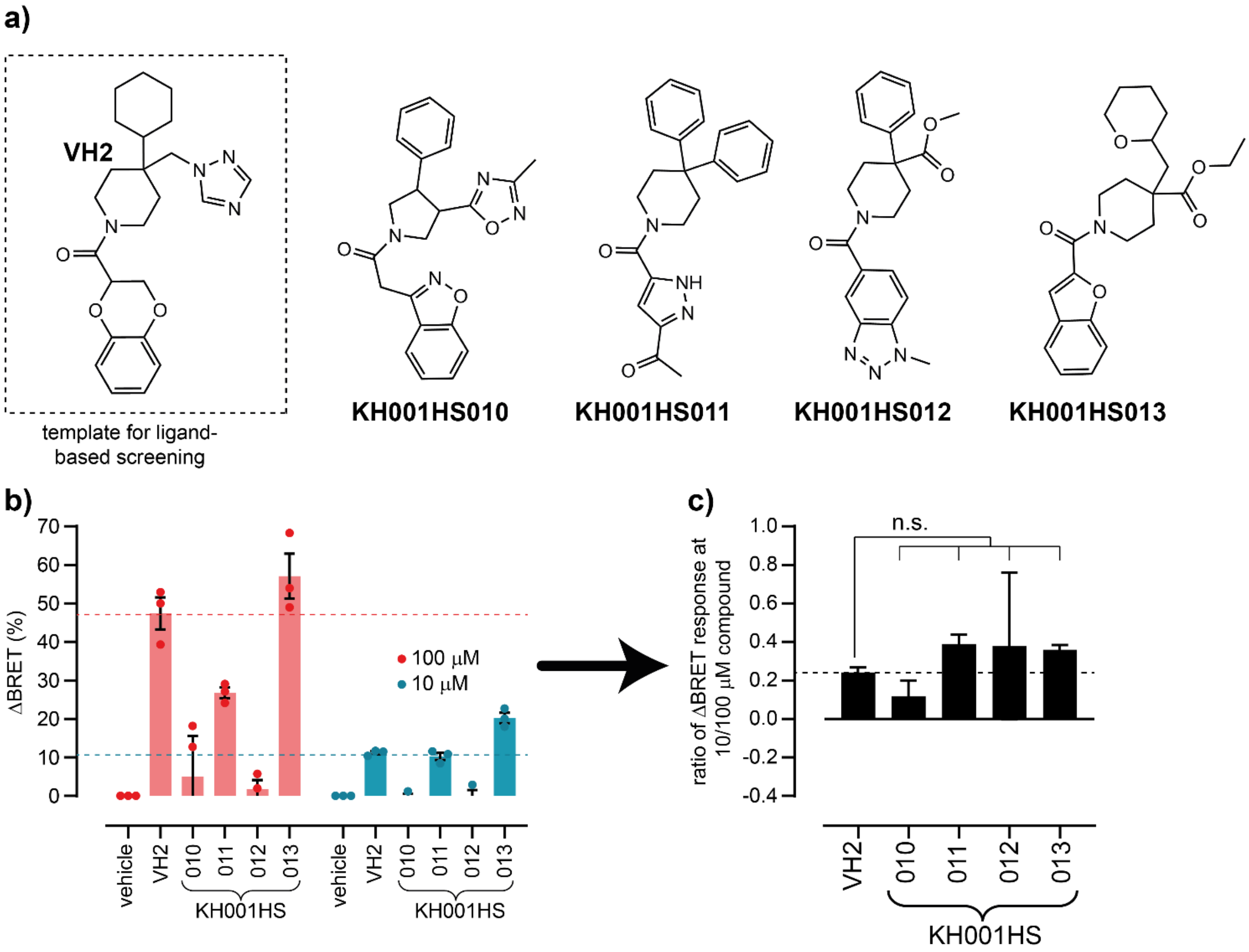

Ligand-based screening for GPR3 ligands. a) Chemical structures of the template molecule, VH2, used for the ligand-based screening of GPR3 ligands and four purchased chemical analogs, KH001HS010 – KH001HS013. b) BRET responses measured in HEK293A cells stably expressing the GPR3-HaloTag/Nluc biosensor and treated with 100 µM or 10 µM of the indicated compounds. c) Response ratios (10 µM over 100 µM) obtained from the data shown in (b). Data show mean ± SEM of three independent experiments. Statistical difference to VH2 in (c) was calculated using One-Way ANOVA followed by Dunnett's multiple comparison.

Figure S221: Ligand-based virtual screening for advanced VH3 analogs.

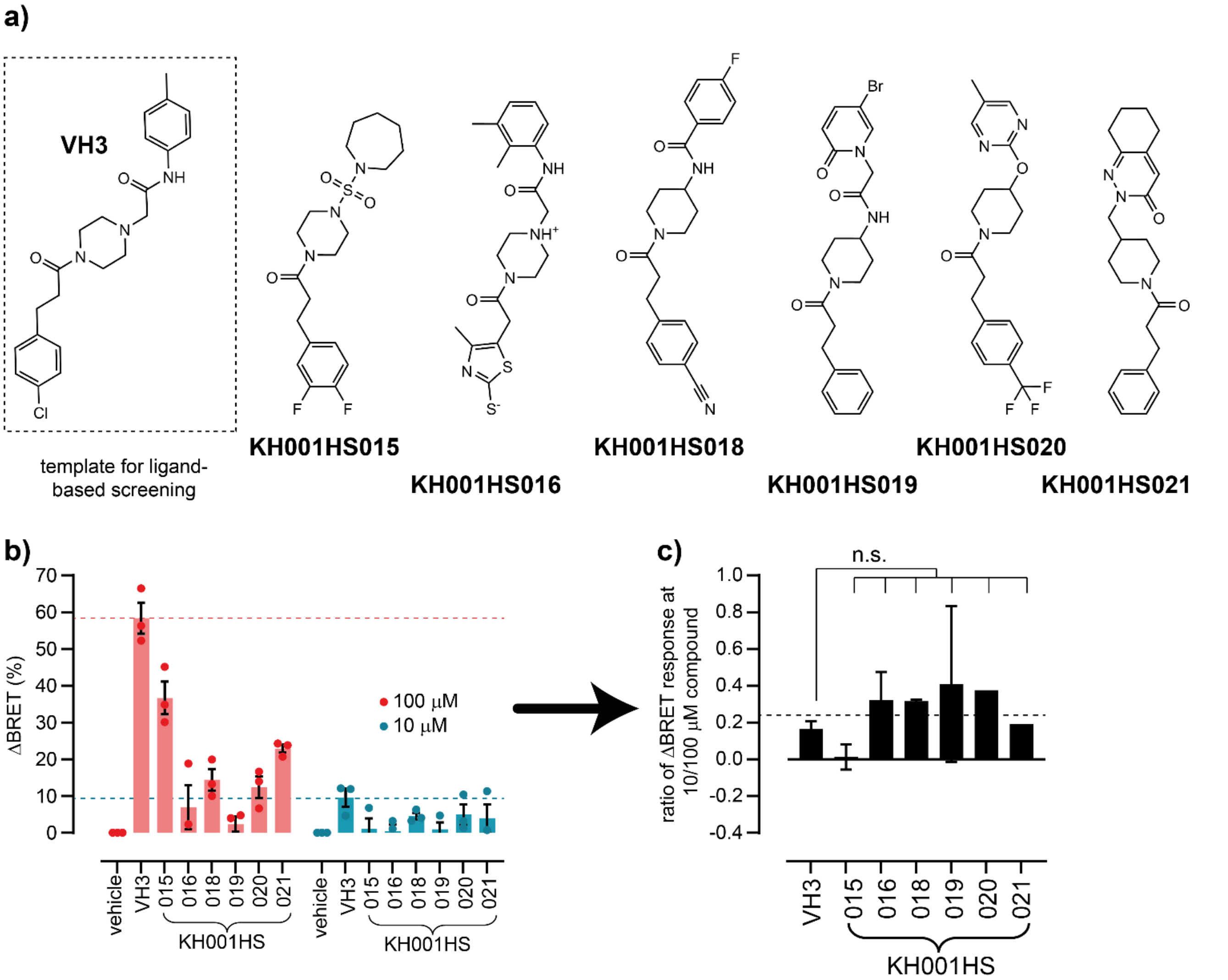

Ligand-based screening for GPR3 ligands. a) Chemical structures of the template molecule, VH3, used for the ligand-based screening of GPR3 ligands and six purchased chemical analogs, KH001HS015 – KH001HS021. b) BRET responses measured in HEK293A cells stably expressing the GPR3-HaloTag/Nluc biosensor and treated with 100 μM or 10 μM of the indicated compounds. c) Response ratios (10 μM over 100 μM) obtained from the data shown in (b). Data show mean ± SEM of three independent experiments. Statistical difference to VH3 in (c) was calculated using One-Way ANOVA followed by Dunnett's multiple comparison.

Figure S222: All atom pair Tanimoto similarity of VH1 analogs tested.

smiles	name	cmpd # in manuscript	set	AP Tanimoto
Cc1cc(OCC(O)CN2C(=O)C(CCc3ccccc3)NC2=O)cc(C)c1	K001HS001		ligand-based virtual screening for VH1 analogs	0.28
Cc1ccc(C)c(C)c1OCC(CN1C(=O)N(C(C1=O)C)c1ccccc1)O	K001HS002			0.28
Cn1c2nc(N3CCCCCC3)n(CC(O)COc3ccccc3)c2c(=O)n(C)c1=O	K001HS003			0.22
Cc1cc(C)c(OCC(O)CN2C(=O)C(C)(c3cc(OC)ccc3)NC2=O)cc1	K001HS004			0.26
Cc1c(C)c(OCC(O)Cn2c3ncnc(N4CCCC4)c3nc2)c(C)cc1	K001HS005			0.18
CC1(c2cc3CCCc3cc2)C(=O)N(CC(O)COc2ccc(F)cc2)C(=O)N1	K001HS006			0.26
CC1(c2c3ccccc3ccc2)C(=O)N(CC(O)COc2c(Cl)cccc2)C(=O)N1	K001HS007			0.37
O=C1C(Cc2c3ccccc3[nH]c2)NC(=O)N1Cc1oc(c2ccccc2)nn1	K001HS008			0.28
N=c1n(CCc2ccccc2)c2ccccc2n1Cc1ccccc1	MR16	26	SAR study with VH1 analogs	0.47
CC(n1c2ccccc2n(Cc2ccccc2)c1=N)c1ccccc1	MR17	31		0.38
N=c1n(CC2CC2)c2ccccc2n1Cc1ccccc1	MR20	33		0.48
N=c1n(CCCc2ccccc2)c2ccccc2n1Cc1ccccc1	MR23	23		0.55
N=c1n(CCCCc2ccccc2)c2ccccc2n1Cc1ccccc1	MR24	21		0.58
N=c1n(CC2CCCC2)c2ccccc2n1Cc1ccccc1	MR27	32		0.42
N=c1n(Cc2ccccc2F)c2ccccc2n1Cc1ccccc1	MR28	28		0.43
N=c1n(Cc2cccc(F)c2)c2ccccc2n1Cc1ccccc1	MR29	29		0.42
N=c1n(Cc2ccc(F)cc2)c2ccccc2n1Cc1ccccc1	MR30	30		0.45
N=c1n(Cc2c(F)cccc2)c2ccccc2n1CC(O)COc1c(C)cccc1	MR35	69		0.64
N=c1n(Cc2ccc(F)cc2)c2ccccc2n1CC(O)COc1ccc(Cl)cc1	MR37	56		0.82
N=c1n(Cc2ccc(F)cc2)c2ccccc2n1CC(O)COc1c(C)cccc1	MR38	71		0.67
N=c1n(Cc2ccc(F)cc2)c2ccccc2n1CC(O)COc1ccc(C)cc1	MR39	66		0.72
N=c1n(Cc2ccc(F)cc2)c2ccccc2n1CC(O)COc1ccccc1	MR40	75		0.75
N=c1n(Cc2ccc(F)cc2)c2ccccc2n1CC(O)COc1ccc(F)cc1	MR41	61		0.72
N=c1n(Cc2cc(F)ccc2)c2ccccc2n1CC(O)COc1ccccc1	MR43	74		0.73
N=c1n(Cc2cc(F)ccc2)c2ccccc2n1CC(O)COc1ccc(C)cc1	MR44	65		0.72
N=c1n(Cc2cc(F)ccc2)c2ccccc2n1CC(O)COc1c(C)cccc1	MR45	70		0.66
N=c1n(Cc2cc(F)ccc2)c2ccccc2n1CC(O)COc1ccc(Cl)cc1	MR46	55		0.82
N=c1n(Cc2cc(F)ccc2)c2ccccc2n1CC(O)COc1ccc(F)cc1	MR47	60		0.72
N=c1n(Cc2ccc(C)cc2)c2ccccc2n1CC(O)COc1ccccc1	MR49	72		0.75
N=c1n(Cc2ccc(C)cc2)c2ccccc2n1CC(O)COc1ccc(C)cc1	MR50	63		0.72
N=c1n(Cc2ccc(C)cc2)c2ccccc2n1CC(O)COc1c(C)cccc1	MR51	68		0.67
N=c1n(Cc2ccc(C)cc2)c2ccccc2n1CC(O)COc1ccc(Cl)cc1	MR52	53		0.82
N=c1n(Cc2ccc(C)cc2)c2ccccc2n1CC(O)COc1ccc(F)cc1	MR53	58		0.72
N=c1n(Cc2ccccc2)c2cc(F)ccc2n1CC(O)COc1ccccc1	MR55	84		0.76
N=c1n(Cc2ccccc2)c2cc(F)ccc2n1CC(O)COc1ccc(C)cc1	MR56	82		0.72
N=c1n(Cc2ccccc2)c2cc(F)ccc2n1CC(O)COc1c(C)cccc1	MR57	83		0.67
N=c1n(Cc2ccccc2)c2cc(F)ccc2n1CC(O)COc1ccc(Cl)cc1	MR58	80		0.82
N=c1n(Cc2ccccc2)c2cc(F)ccc2n1CC(O)COc1ccc(F)cc1	MR59	81		0.72
N=c1n(Cc2c(F)cccc2)c2ccccc2n1CC(O)COc1ccccc1	MR60	73		0.72
N=c1n(Cc2c(F)cccc2)c2ccccc2n1CC(O)COc1ccc(C)cc1	MR61	64		0.72
N=c1n(Cc2c(F)cccc2)c2ccccc2n1CC(O)COc1ccc(F)cc1	MR62	59		0.72
N=c1n(Cc2c(F)cccc2)c2ccccc2n1CC(O)COc1ccc(Cl)cc1	MR63	54		0.82
N=c1n(Cc2ccccc2)c2ccccc2n1CC(O)COc1ccc(C)cc1	MR69	62		0.87
N=c1n(Cc2ccccc2)c2ccccc2n1CC(O)COc1c(C)cccc1	MR70	67		0.75
N=c1n(Cc2ccccc2)c2ccccc2n1CC(O)COc1ccc(Cl)cc1	MR71	52		1
N=c1n(Cc2ccccc2)c2ccccc2n1CC(O)COc1ccc(F)cc1	MR72	57		0.87
N=c1n(Cc2ccccc2)c2ccccc2n1CC	MR73	36		0.36
N=c1n(Cc2ccccc2)c2ccccc2n1CC(C)(C)C	MR74	34		0.32
N=c1n(Cc2ccccc2)c2ccccc2n1CC(C)C	MR75	35		0.38
N=c1n(Cc2ccccc2)c2ccccc2n1C	MR76	37		0.32
N=c1n(Cc2ccccc2)c2ccccc2n1Cc1ccccc1	MR77	27		0.43
N=c1n(Cc2ccc(F)cc2)c2ccccc2n1CCCCc1ccccc1	MR78	76		0.56
N=c1n(Cc2ccc(F)cc2)c2ccccc2n1CC1CC1	MR79	77		0.44
N=c1n(Cc2ccccc2)c2ccccc2n1CCCCc1cnccc1	MR83	22		0.55
N=c1n(Cc2ccccc2)c2ccccc2n1CCCc1ccncc1	MR84	24		0.51
N=c1n(Cc2ccccc2)c2ccccc2n1CCCN1CCNCC1	MR85	25		0.35

Figure S223: BRET changes of VH2 and VH3 analogs at two concentrations in independent experiments with the GPR3 biosensors.

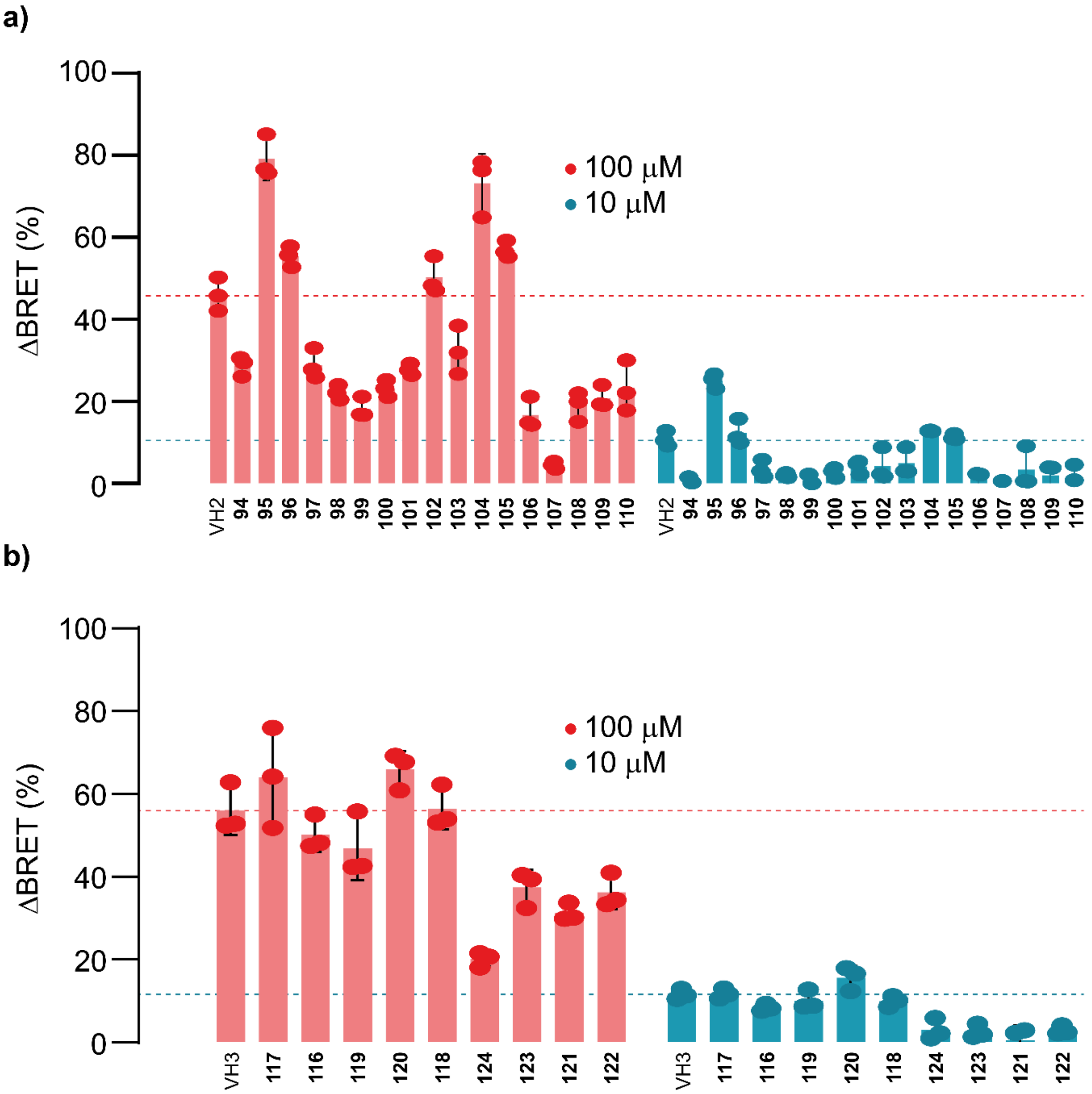

Vehicle-corrected BRET changes of GPR3-HaloTag(618)/Nluc induced by VH2 and 17 chemical analogs (a) and by VH3 and 9 chemical analogs (b). Data show mean ± SEM of three independent experiments conducted in HEK293A cells stably expressing the indicated biosensor.

Figure S224: Concentrations response curves of **33** at independent experiments with the GPR3 biosensors.

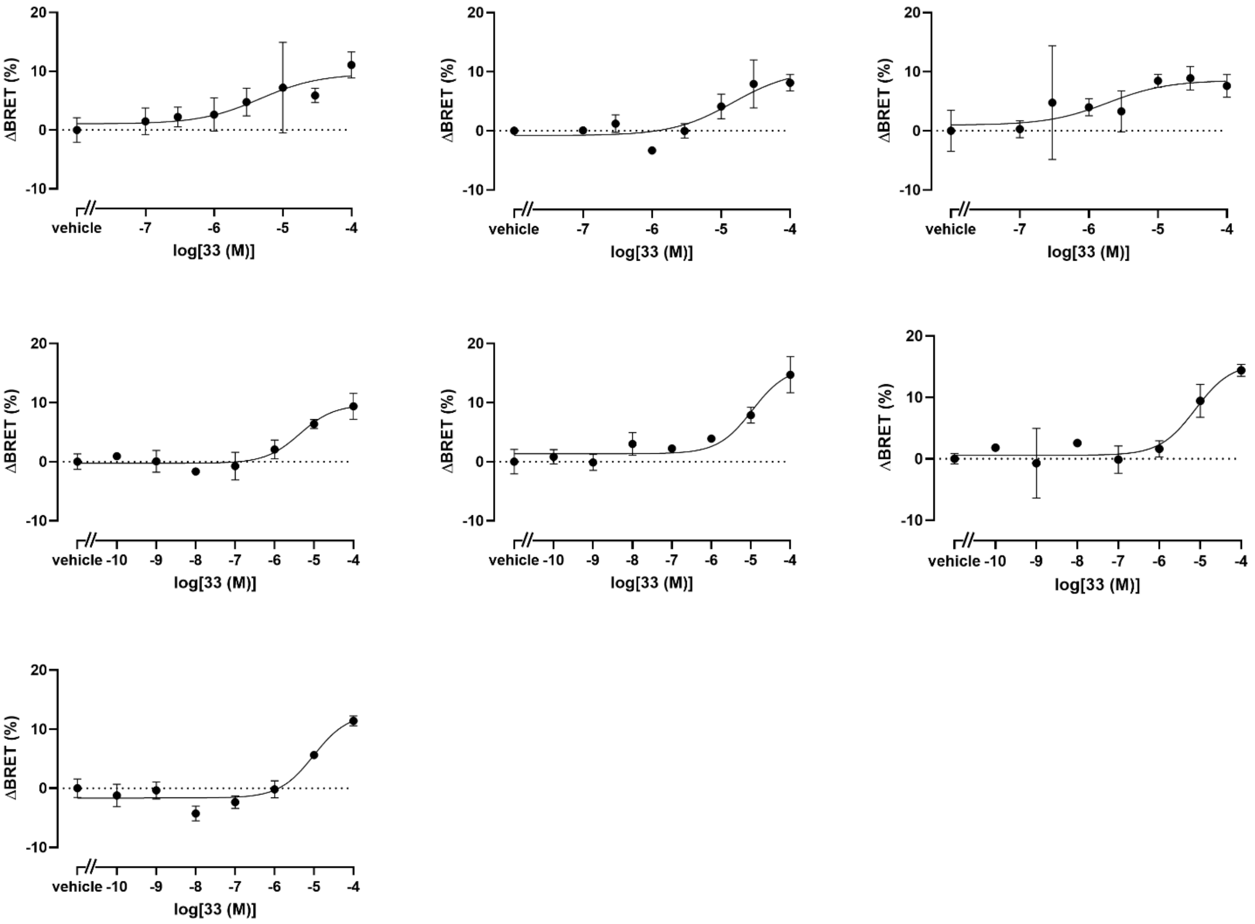

Concentration-dependent BRET changes induced by **33** at GPR3-HaloTag(618)/Nluc in seven independent experiments. Data show mean ± SD of independent biological replicates conducted in HEK293A cells stably expressing the GPR3 conformational biosensor.

Figure S225: Energy-minimized, proposed binding poses of **33** and VH1/**52** in GPR3

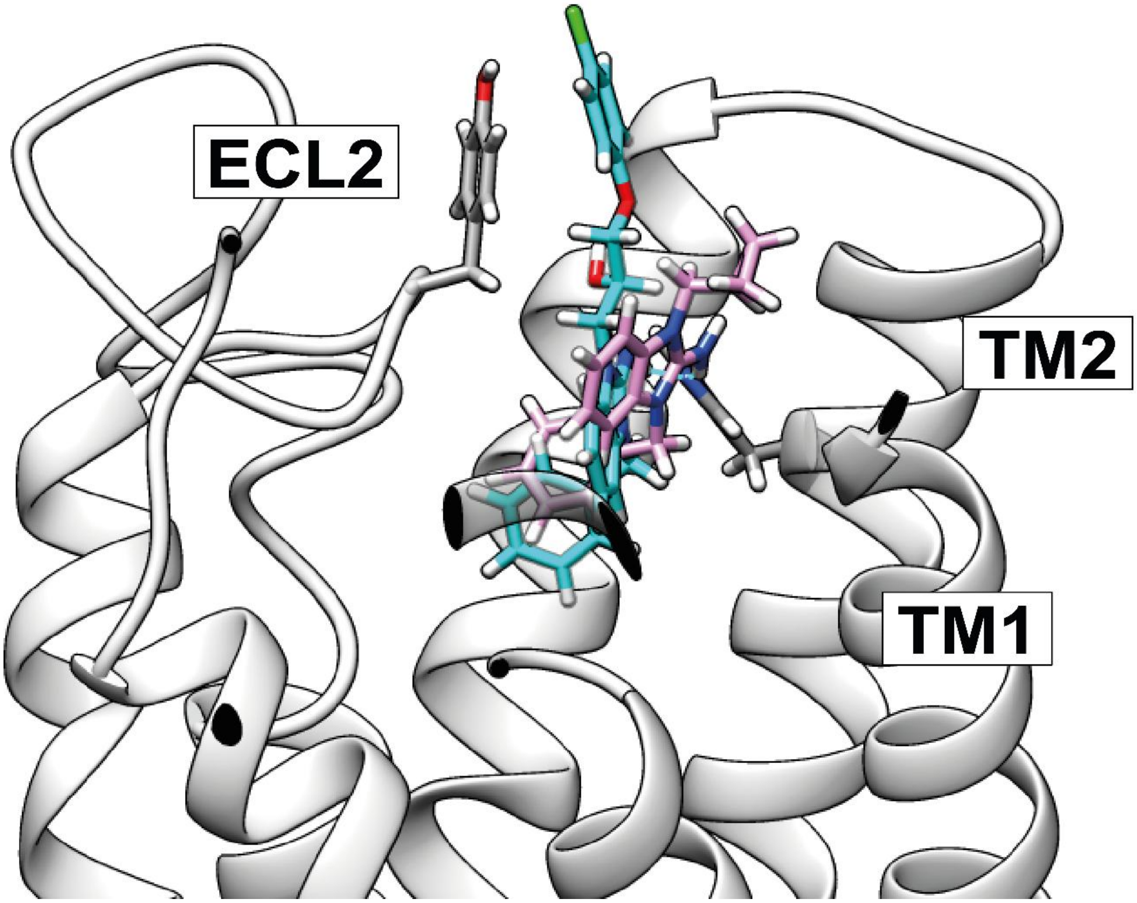

Overlay of energy-minimized, proposed binding poses of **33** (pink) and VH1/**52** (cyan) in GPR3 indicate a potential shift of **33** toward transmembrane helix 2 (TM2) (model based on PDB 8X2K).

Figure S226: Alternatively proposed binding pose of **33** in GPR3.

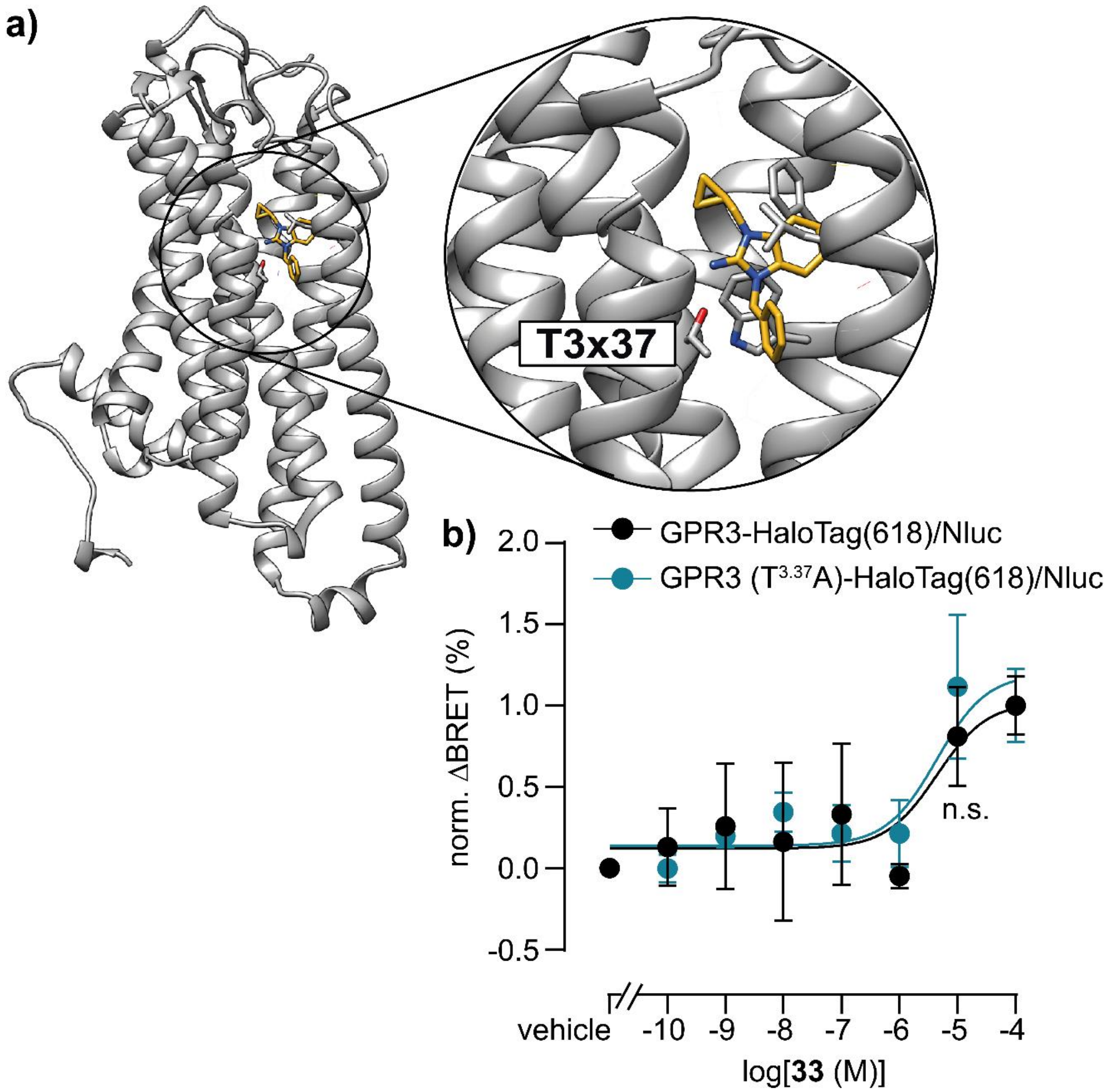

a) Computationally predicted binding poses of **33** (pink) in GPR3 (model based on PDB 8X2K). b) Concentration response curve of **33** obtained with wildtype and mutant GPR3 conformational sensors. Data show mean ± SEM of three independent experiments conducted in transiently transfected HEK293A cells. Statistical difference of the pEC_{50} values was tested using extra-sum-of-squares F-test (n.s. > 0.05).

Figure S227: Effect of **33** preincubation on oleamide- and OEA-induced GPR3 conformational changes.

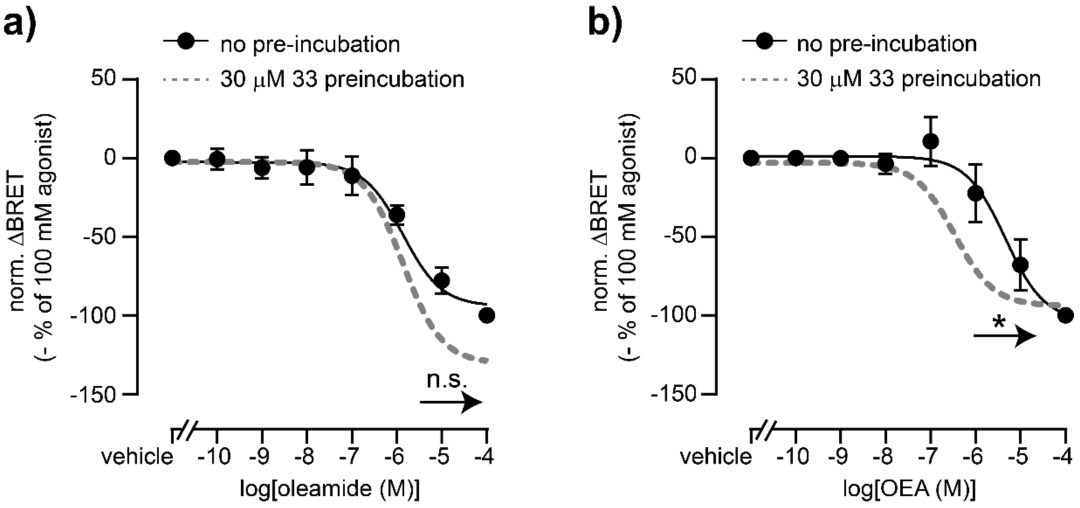

a, b) Concentration-response curves of oleamide- (a) and OEA-induced GPR3 conformational changes (b) with and without preincubation with 30 μM **33**. All vehicle-corrected ΔBRET responses have been divided by the negative 100 μM agonist-induced signal of each dataset and multiplied with a factor of 100. The grey dotted line represents the agonist response without 33 and is derived from the data represented in main Figure 1f. All experiments have been conducted in HEK294A cells expressing the GPR3 conformational biosensor. Data show the mean SEM of four to five biological replicates. Statistical difference of the EC_{50} values was tested using the extra-sum-of-squares F-test (n.s. > 0.05).

Figure S228: Activity of **33** at lipid-binding GPCRs phylogenetically related to GPR3.

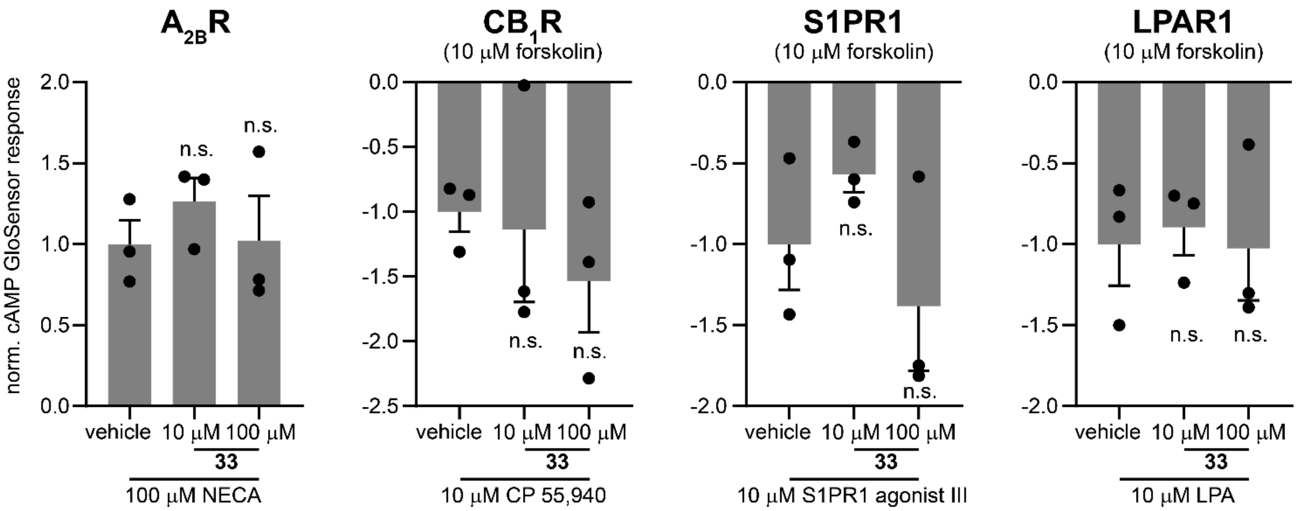

GPCR agonist induced luminescence changes of the cAMP GloSensor™ 22F after preincubation with 100 μM or 10 μM **33**, or vehicle control. Cells expressing $G_{i/o}$-coupled CB1R, S1PR1 and LPAR1 receptors were pre-stimulated with 10 μM forskolin to induce cAMP production prior to the baseline cAMP read and receptor stimulation with agonists. Data show mean ± SEM of three independent experiments conducted in transiently transfected HEK293A cells. Statistical difference of the luminescence response to vehicle control was tested using one-way ANOVA followed by Dunnett's multiple comparison (n.s. > 0.05).
